# DNA methylation profiling identifies long-range epigenetic silencing of clustered protocadherins as a key determinant of meningioma progression

**DOI:** 10.1038/s41467-026-77170-3

**Published:** 2026-08-29

**Authors:** Daniel J. Merk, Peter Paßlack, Surender Surender, Foteini Tsiami, Lara A. Haeusser, Vanessa Arnold, Vijayasarathy Sampath-Kumar, Lisa Sevenich, Andrea D. Maier, Tiit Mathiesen, Marcos Tatagiba, Hanna Gött, Jonas Tellermann, Felix Behling, Jens Schittenhelm, Hannes Becker, Ghazaleh Tabatabai

**Affiliations:** 1 Department of Neurology and Interdisciplinary Neuro-Oncology, Hertie Institute for Clinical Brain Research, University Hospital Tübingen, Eberhard Karls University Tübingen, Tübingen, Germany; 2 Cluster of Excellence iFIT (EXC 2180) “Image Guided and Functionally Instructed Tumour Therapies”, Eberhard Karls University Tübingen, Tübingen, Germany; 3 Department of Pediatric Oncology, Dana-Farber/Boston Children’s Cancer and Blood Disorders Center, Boston, MA USA; 4 M3 Research Center for Malignome, Metabolome, and Microbiome, University Hospital Tübingen, Eberhard Karls University Tübingen, Tübingen, Germany; 5 Graduate Training Center for Neuroscience, Eberhard Karls University Tübingen, Tübingen, Germany; 6 German Consortium for Translational Cancer Research (DKTK), Partner Site Tübingen, German Cancer Research Center (DKFZ), Heidelberg, Germany; 7 Department of Neurosurgery, Copenhagen University Hospital, Rigshospitalet, Copenhagen, Denmark; 8 Department of Neurosurgery and Neurotechnology, University Hospital Tübingen, Eberhard Karls University Tübingen, Tübingen, Germany; 9 Institute of Neuropathology, Department of Pathology and Neuropathology, University Hospital Tübingen, Eberhard Karls University Tübingen, Tübingen, Germany; 10 Comprehensive Cancer Center Tübingen Stuttgart, University Hospital Tübingen, Eberhard Karls University Tübingen, Tübingen, Germany

**Keywords:** CNS cancer, CNS cancer, CNS cancer, DNA methylation, Cancer epigenetics

## Abstract

Meningioma is the most common primary brain tumour in adults. However, molecular drivers of progression occurring in a subset of meningiomas are poorly understood. We hypothesise that epigenomic variations are causal for the clinical heterogeneity of meningiomas and may be functionally relevant for disease progression. To test this hypothesis, we perform global DNA methylation profiling of a large cross-sectional cohort and a longitudinal cohort of human meningiomas. Our analysis identifies a DNA hypermethylation signature that is correlated with clinical outcomes and enables more accurate prognostication for meningiomas than previous classification systems. Analyses of longitudinal high-grade meningioma samples in comparison to clinically benign meningiomas and normal meningeal tissue show convergent contributions but differing plasticity of copy number variations and DNA hypermethylation along the trajectory of meningioma progression. Systematic analysis of DNA hypermethylation in high-grade meningiomas unravels a tumour suppressive role of clustered protocadherins by restricting β-catenin nuclear localisation, consistent with the association between nuclear β-catenin staining and meningioma progression. Together, our study provides fundamental insights into the molecular mechanisms underlying the heterogeneity of clinically benign and aggressive meningiomas and the microevolutionary adaptation during disease progression.

## Abstract (web summary)

The molecular drivers of meningioma progression remain poorly understood. Here, the authors profile DNA methylation in cross-sectional and longitudinal meningioma cohorts and identify molecular features of benign and aggressive disease and microevolutionary adaptation during progression.

## Introduction

Meningiomas might arise from prostaglandin D2 synthase-positive meningeal precursor cells and account for approximately 40% of all primary intracranial tumours^1,2^. While the majority of these tumours can be cured by resection alone, about 20% of meningiomas display an aggressive clinical behaviour with a tendency to recur, requiring post-operative treatment approaches. The main therapeutic modality after surgery is photon or proton radiation therapy^3^. Recent clinical trials have investigated chemotherapies^4^, and therapeutic approaches employing radioligands^5^. Gaining deeper insights into the molecular drivers of clinically aggressive or high-grade meningiomas will be essential for developing better therapeutic strategies.

In current clinical practice, meningiomas are primarily classified based on histopathological features assigned to one of three WHO grades (grades 1–3) which are intended to reflect biological aggressiveness and risk of recurrence. The 2021 World Health Organization (WHO) Classification of Tumours of the Central Nervous System integrates histological subtype, other histological atypia features such as brain invasion, mitotic activity or necrosis, and selected molecular alterations into this grading framework^6^. CNS WHO grade 1 meningiomas are considered benign, grade 2 tumours are classified as atypical, and grade 3 tumours as anaplastic or malignant. Importantly, recent updates to the WHO classification incorporate molecular criteria such as homozygous deletion of *CDKN2A/B* or oncogenic *TERT* promoter mutations, which can independently define CNS WHO grade 3 disease irrespective of histological appearance.

The most common genetic alteration in meningiomas is functional loss of the tumour suppressor gene *NF2*, which might occur in low-grade meningiomas (37%), but is seen more frequently in higher grade tumours (63%)^7^. *NF2* mutations occur both in the germline, resulting in NF2-related Schwannomatosis, or as somatic variants. Meningiomas lacking *NF2* alterations may harbour other recurrent mutations in genes such as *TRAF7*, *KLF4*, *AKT1*, or *SMO*, but these tumours generally show better clinical outcomes than *NF2*-mutated meningiomas^8,9^. While high-grade meningiomas have a higher mutational burden than low-grade meningiomas, no recurrent genetic coding mutations other than *NF2* have been identified thus far in high-grade meningiomas, and numbers of targetable alterations in high-grade tumours tend to be lower than in low-grade meningiomas^10^. High-grade meningiomas demonstrate significantly higher levels of genome disruption, and broad copy number variations (CNVs) affecting distinct chromosome arms such as 1p, 6q, 14q, and 22q have been clearly associated with intermediate or poor prognosis^11,12^. Focal CNVs may also be prognostic in meningioma, and homozygous loss of the cell cycle inhibitor *CDKN2A/B* was recently incorporated in the 2021 WHO classification of tumours of the CNS, defining CNS WHO grade 3 meningioma^6^.

Despite the suggested histo-molecular classification framework, clinical outcomes demonstrate that patients with meningiomas of identical histological diagnosis and identical CNS WHO grade may exhibit markedly heterogeneous disease trajectories, highlighting the limitations of morphology-based grading alone and underscoring the need for improved prognostic markers. Single-gene alterations such as oncogenic variants in the *TERT* promoter, *CDKN2A/B* deletions, or *BAP1*-inactivating alterations, all of which are associated with a high risk of recurrence, might account for this heterogeneity^13–17^. In addition, molecular features such as DNA methylation profiling alone or in combination with gene expression and CNV data provide a more accurate prognostication than CNS WHO grading alone^18–22^. While a unified taxonomy for groups based on molecular markers is still pending, molecular groups defined by different studies share common underlying biology. Since all these studies relied on global DNA methylation profiling, alone or in combination with gene expression and CNV data, DNA methylation is likely the most robust molecular marker for molecular groups of meningioma. However, it remains to be determined whether distinct methylation profiles are merely remnants of differing lineage or development-associated processes within molecular groups, or whether changes in DNA methylation are functionally relevant for meningioma progression.

In this work, we perform comprehensive DNA methylation profiling using the MethylationEPIC v2.0 array in a cohort of 231 meningioma samples to assess the relationship of methylation patterns and disease progression. We further investigate the contributions of CNVs and DNA methylation differences to the microevolutionary process of meningioma development and progression, considering a hypothetical trajectory from normal meningeal tissue, over clinically benign tumours, to the longitudinal development of high-grade meningiomas. Finally, we comprehensively annotate and validate a DNA hypermethylation signature in high-grade meningiomas and provide insights into the mechanisms downstream of this epigenomic pattern.

## Results

### A DNA hypermethylation signature underlies meningioma progression independent of molecular groups

**Fig. 1:**
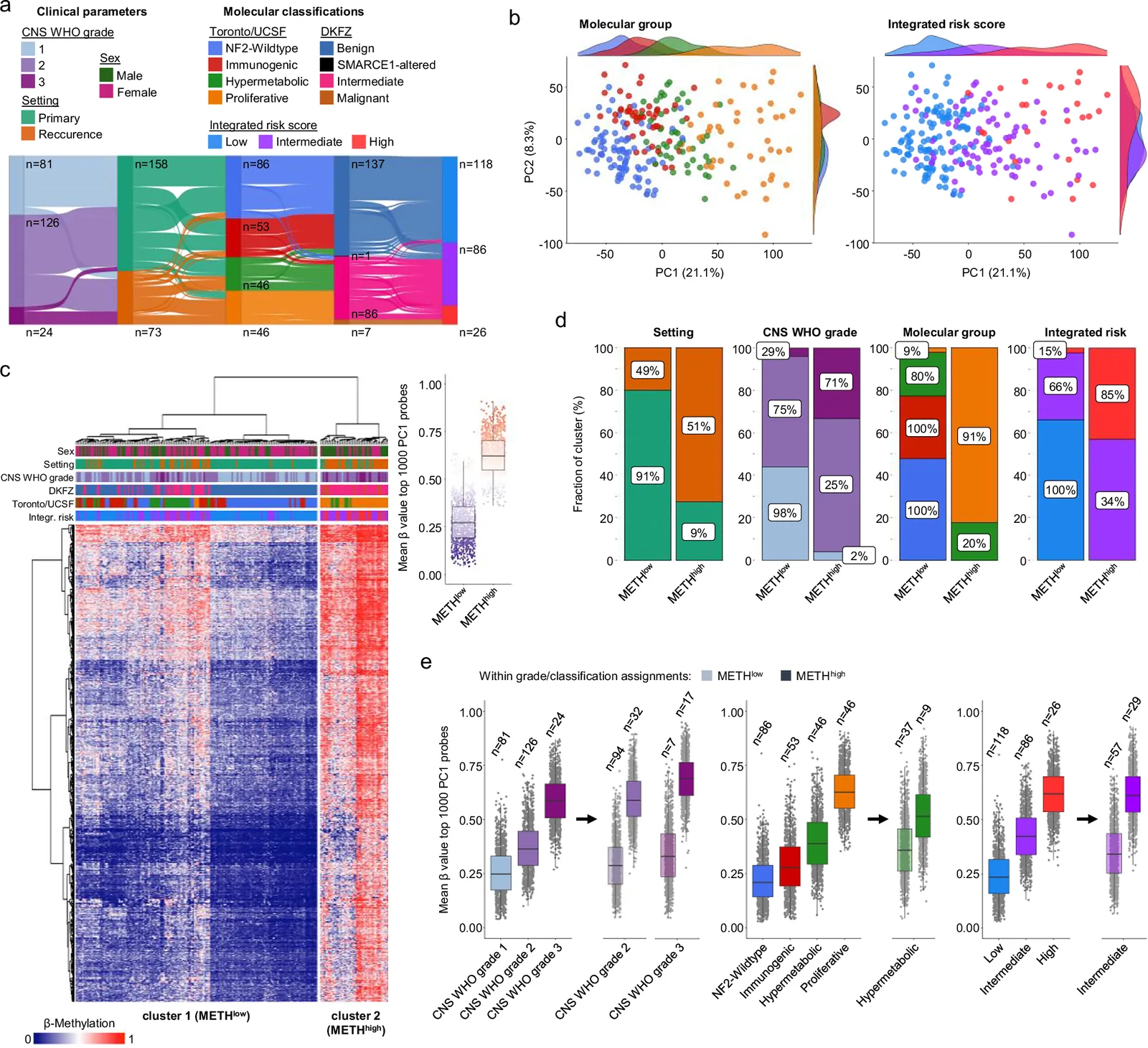
A DNA hypermethylation signature correlates with aggressive clinical behaviour of meningioma. **a** Sankey plot showing the relationship of selected clinical and molecular parameters for the Discovery cohort. The number of tumour samples is indicated inside the figure panel. **b** PCA analyses of methylation data in relation to molecular groups and integrated risk scores of meningioma. Density margins illustrate the distribution of corresponding subclassifications along the first two principal components. **c** Unsupervised hierarchical clustering using the top 1000 probes contributing to PC1 separates the Discovery meningioma cohort into a hypomethylated (METH^low^; *n* = 180) and a hypermethylated cluster (METH^high^; *n* = 51). Boxplot illustrates preferential hypermethylation of CpG sites in cluster 2 (METH^high^). **d** Relative assignments of meningioma samples to METH^low^ and METH^high^ clusters depending on classification by clinical setting, CNS WHO grading, molecular groups, and integrated risk score. Percentages on the y-axis show the fraction within each methylation cluster associated with a given condition, while percentages in boxes represent the partition of each condition to each methylation cluster. **e** Mean β values for PC1-associated probes across meningioma samples in relation to CNS WHO grading, molecular groups, and integrated risk score within the Discovery cohort. Differential methylation between METH^low^ and METH^high^ cases within CNS WHO grade 2, Hypermetabolic, and intermediate integrated risk meningiomas is shown. The number of tumour samples per condition is indicated inside the figure panel. For all boxplots, boxes represent the interquartile range (IQR) with the median indicated by the centre line and whiskers extending to extreme outliers within 1.5× IQR. Source data are provided as a Source Data file.

To investigate a functional role of DNA methylation changes during meningioma progression, we performed DNA methylation profiling on 231 meningiomas from patients treated at the University Hospital Tübingen (Discovery cohort). Our cohort included 158 primary and 73 recurrent tumour samples that passed stringent quality controls, with no patient overlap between primary and recurrent tumours (Fig. 1a, Supplementary Fig. 1, and Supplementary Data 1). Compared to the normal epidemiological distribution^1,6^, this cohort is substantially enriched for high-grade meningiomas, with 55% CNS WHO grade 2 and 10% CNS WHO grade 3 cases. DNA methylation profiling was performed using the MethylationEPIC v2.0 arrays, and data were analysed using the SeSAMe workflow that controls for the influence of genomic copy-number variants (CNVs) on β-methylation values^23^. The initial dataset was analysed for technical batch effects and corrected accordingly using linear modelling (Supplementary Fig. 2). Molecular groups were predicted using the publicly available CNS tumour classifier^24^, or by constructing an in-house support vector machine classifier based on previous literature (Supplementary Fig. 3a-c)^19^. An integrated risk score^25^ was calculated based on DNA methylation profiles, CNS WHO grading, and CNVs derived from methylation array data^26^. Of note, stratification of the Discovery cohort by molecular groups or risk score assignment was significantly prognostic for disease progression and consistent with current classification schemes (Supplementary Fig. 4).

While we did not detect any global changes in DNA methylation associated with grading or molecular groups (Supplementary Fig. 5a), we hypothesised that focal changes in DNA methylation patterns may contribute to disease progression in meningioma. To catalogue DNA methylation marks which are potentially implicated in disease progression, we performed principal component analyses (PCA) and assessed individual components for their ability to separate tumour samples based on current classification schemes used for meningioma stratification, including CNS WHO grade, molecular group as defined by DNA methylation patterns^19^, and integrated risk score. We found PC1 (21.1% of total variance) to be most associated with meningioma progression risk groups (Fig. 1b, Supplementary Fig. 6, and Supplementary Fig. 7), with a clear gradual separation of low-risk, intermediate-risk, and high-risk meningiomas along PC1. Clustering based on the top methylation probes contributing to PC1 robustly suggested the presence of two divergent clusters, which we termed METH^low^ and METH^high^ based on their disparate methylation patterns (Fig. 1c and Supplementary Fig. 8a-d). We noted preferential assignment of low-risk (CNS WHO grade 1, NF2-Wildtype/Immunogenic, low integrated risk score) and high-risk (CNS WHO grade 3, Proliferative, high integrated risk) tumours to METH^low^ and METH^high^ clusters, respectively (Fig. 1d and Supplementary Fig. 8e). For example, while all NF2-Wildtype/Immunogenic meningiomas formed part of the METH^low^ cluster (48% and 29% of that cluster, respectively), 91% of Proliferative meningiomas were associated with the METH^high^ cluster (82% of that cluster). In line with this, we found a moderate correlation of the average methylation of cluster probes and the Ki67-positive cell fraction in meningioma tissue (Supplementary Fig. 8f). In contrast, meningiomas with intermediate-risk prognostication (CNS WHO grade 2, Hypermetabolic, intermediate integrated risk) showed a more balanced allocation to methylation clusters, with 25–34% of samples being distributed to the high-risk METH^high^ cluster, and corresponding differences in methylation of marker CpG sites (Fig. 1e).

Validating our findings in an independent, publicly available cohort of 565 meningiomas (UCSF cohort)^20^, we find that global methylation levels are not associated with tumour aggressiveness (Supplementary Fig. 5b). We used a support vector machine classifier with high accuracy and good performance in receiver operating curve analysis to assign samples from the UCSF cohort to methylation clusters (Supplementary Fig. 3d-f). Of note, all NF2-Wildtype/Immunogenic meningiomas with a favourable outcome were classified as METH^low^ and showed hypomethylation of associated probes, while Hypermetabolic/Proliferative meningiomas were assigned to both clusters (Supplementary Fig. 9).

**Fig. 2:**
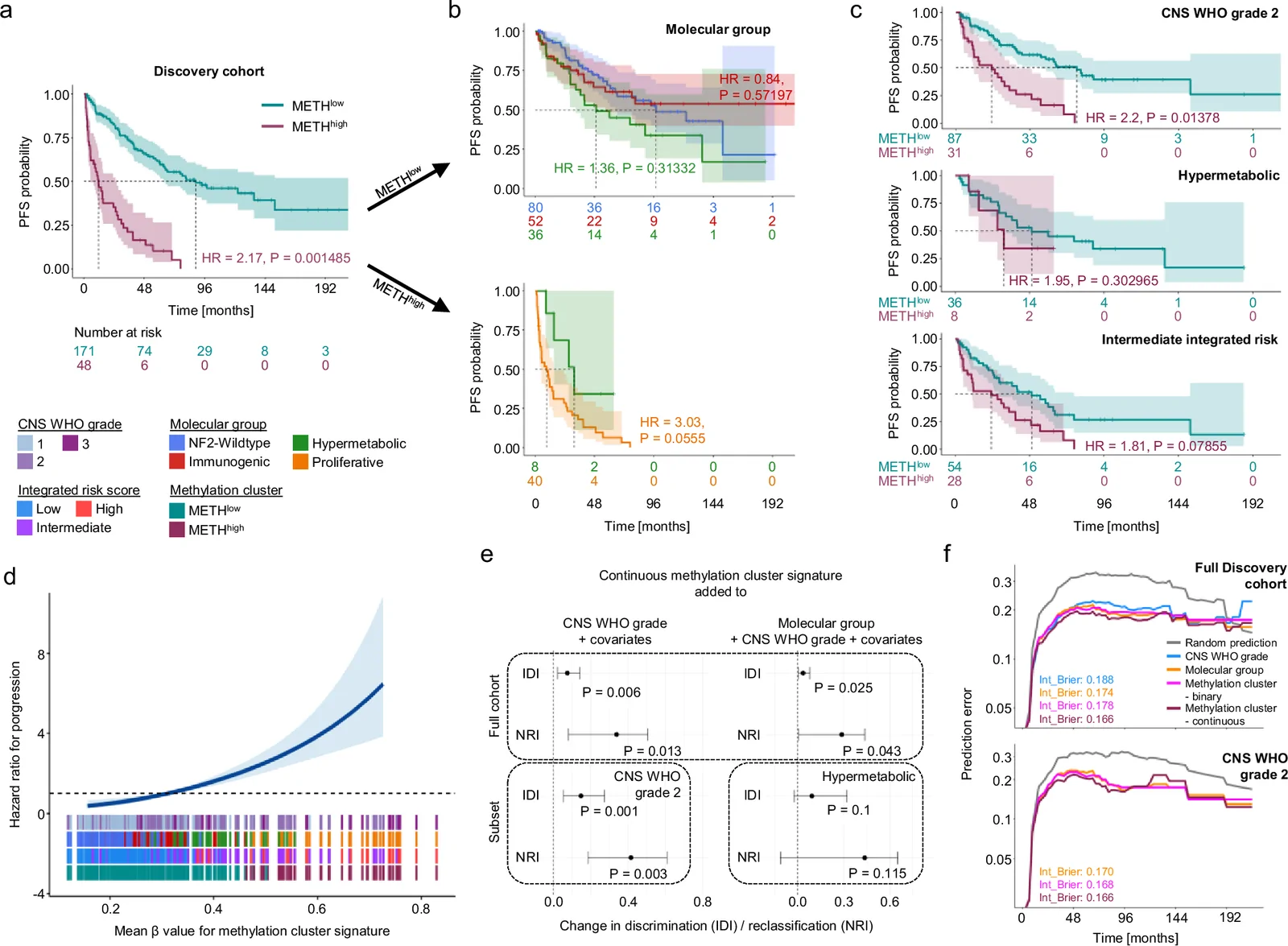
The methylation cluster signature improves risk stratification across histopathological grades and molecular groups. **a** PFS outcomes within the Discovery cohort stratified by methylation cluster affiliation (METH^low^: *n* = 171; METH^high^: *n* = 48). 95% confidence intervals are indicated for all survival curves. Hazard ratios and *P* values from two-sided Cox proportional regression modelling are shown for all comparisons. **b** PFS outcomes for further subcategorization by molecular group in METH^low^ (NF2-Wildtype: *n* = 80; Immunogenic: *n* = 52; Hypermetabolic: *n* = 36) and METH^high^ (Hypermetabolic: *n* = 8; Proliferative: *n* = 49) meningiomas. Categories with less than five cases were omitted from analyses. **c** PFS outcomes for meningiomas with intermediate prognosis (CNS WHO grade 2, Hypermetabolic, and Intermediate integrated risk) stratified by METH^low^ and METH^high^ assignment. CNS WHO grade 2: *n* = 87 METH^low^ and *n *= 31 METH^high^; Hypermetabolic: *n* = 36 METH^low^ and *n* = 8 METH^high^; Intermediate integrated risk: *n* = 54 METH^low^ and *n* = 28 METH^high^. **d** Restricted cubic spline modelling across the entire cohort (*n* = 219 patients) showing the nonlinear association between a continuous cluster methylation signature (sample-wise mean *β* values for cluster probes) and progression risk, with rugs indicating CNS WHO grade, molecular group, integrated risk scores, and methylation cluster affiliation. The tumour sample at the median of the continuous rank was assigned a hazard ratio of 1. The solid line shows the spline-estimated hazard ratio for progression, with shaded areas representing the 95% confidence interval. **e** Point estimates and 95% confidence intervals for integrated discrimination improvement (IDI) and net reclassification improvement (NRI) illustrating the change in discrimination and reclassification associated with inclusion of the continuous methylation cluster signature at 36 months. Analysis included *n* = 219 (full cohort), *n* = 118 patients (CNS WHO grade 2), or *n *= 44 patients (Hypermetabolic). *P* values and confidence intervals were estimated using 2000 perturbation iterations. **f** Brier prediction plots for indicated models using the full Discovery cohort (top, *n* = 219) or only CNS WHO grade 2 meningiomas (bottom, *n* = 118). Integrated Brier scores for the entire follow-up time are indicated (Int-Brier). Source data are provided as a Source Data file.

Meningioma methylation clusters were strongly correlated with prognostic markers such as CNS WHO grade and integrated risk score, suggesting that methylation clustering might have prognostic value as well. Kaplan-Meier curve analysis clearly distinguished methylation clusters with respect to progression-free survival (PFS), and clusters were independently significant for meningioma progression when controlling for competing variables of aggressive meningioma behaviour including sex, tumour setting, CNS WHO grade, extent of resection, and adjuvant radiotherapy in a Cox proportional hazard regression model (HR, 2.17; 95% CI, 1.34-3.46; *P* = 0.001485) (Fig. 2a, Supplementary Fig. 10a, and Supplementary Data 2). Separating METH^low^ meningiomas into a low-level and mid-level methylation group did not show any significant prognostic differences within this methylation cluster, further supporting the clinical relevance of our previous separation into two clusters (Supplementary Fig. 10b,c).

Since radiotherapy (RT) is commonly applied to meningioma patients, we also assessed the correlation of both prior RT and adjuvant RT with PFS in the context of methylation clusters. Prior RT was not associated with differences in PFS within either METH^low^ or METH^high^ tumours, including when analyses were restricted to recurrent cases only (Supplementary Fig. 10d). In contrast, adjuvant RT was associated with a significant PFS benefit in METH^low^ meningiomas, and this effect was mostly driven by tumours after subtotal resection (Supplementary Fig. 10e,f). This benefit appeared more profound than in METH^high^ tumours, but a likelihood ratio test for an interaction of adjuvant RT and methylation cluster did not support a clear sensitivity difference towards adjuvant RT. While these data have to be seen in the context of the limited cohort size, in particular for METH^high^ meningiomas, they are in line with previous reports demonstrating reduced sensitivity to adjuvant RT in biologically aggressive meningiomas^27^.

We noted that classification groups known to have different prognosis (e.g. NF2-Wildtype vs. Hypermetabolic or Hypermetabolic vs. Proliferative) were no longer significant within methylation clusters (Fig. 2b). Furthermore, methylation clusters successfully stratified CNS WHO grade 2 cases into groups with distinct risk profiles, and similar stratification was seen for molecularly-defined intermediate-risk meningiomas, although this did not reach statistical significance (Fig. 2c and Supplementary Fig. 10g). To capture potential nonlinear risk gradients beyond binary stratification, we modelled the continuous methylation cluster signature—defined as the mean methylation of cluster-defining probes—using restricted cubic splines. These analyses revealed a continuous, nonlinear relationship between methylation and risk of progression (Fig. 2d), showing that increasing methylation of cluster-related CpG sites is associated with a higher risk of progression over time. Overall, the methylation cluster signature demonstrated improved discrimination and reclassification over current CNS WHO grading, both in univariate as well as multivariate analyses, and this was particular evident when defining the methylation signature as a continuous variable (Fig. 2e and Supplementary Fig. 11). Furthermore, the continuous signature significantly improved performance beyond molecular groups and clinical covariates across the entire cohort, while this was not the case in Hypermetabolic meningioma. Accordingly, we found improved calibration and more accurate absolute risk estimation (Brier score) within the entire cohort in particular for the continuous signature as compared to molecular groups (Fig. 2f; ΔInt_Brier = 0.008, *P* = 5.15*10^−9^, two-sided Wilcoxon signed-rank test), while this effect was less pronounced in CNS WHO grade 2 meningioma (ΔInt_Brier = 0.004, *P* = 0.002627, two-sided Wilcoxon signed-rank test).

Together, we identified a DNA methylation signature indicative of disease progression and aggressive clinical behaviour in meningioma. Our results show that methylation clusters capture a biologically meaningful transition along a continuous risk gradient, highlighting the prognostic value that might thus have direct clinical implications for the management of meningioma patients (Supplementary Fig. 12).

### Independent, but convergent effects of CNVs and methylation cluster signature on meningioma progression

**Fig. 3:**
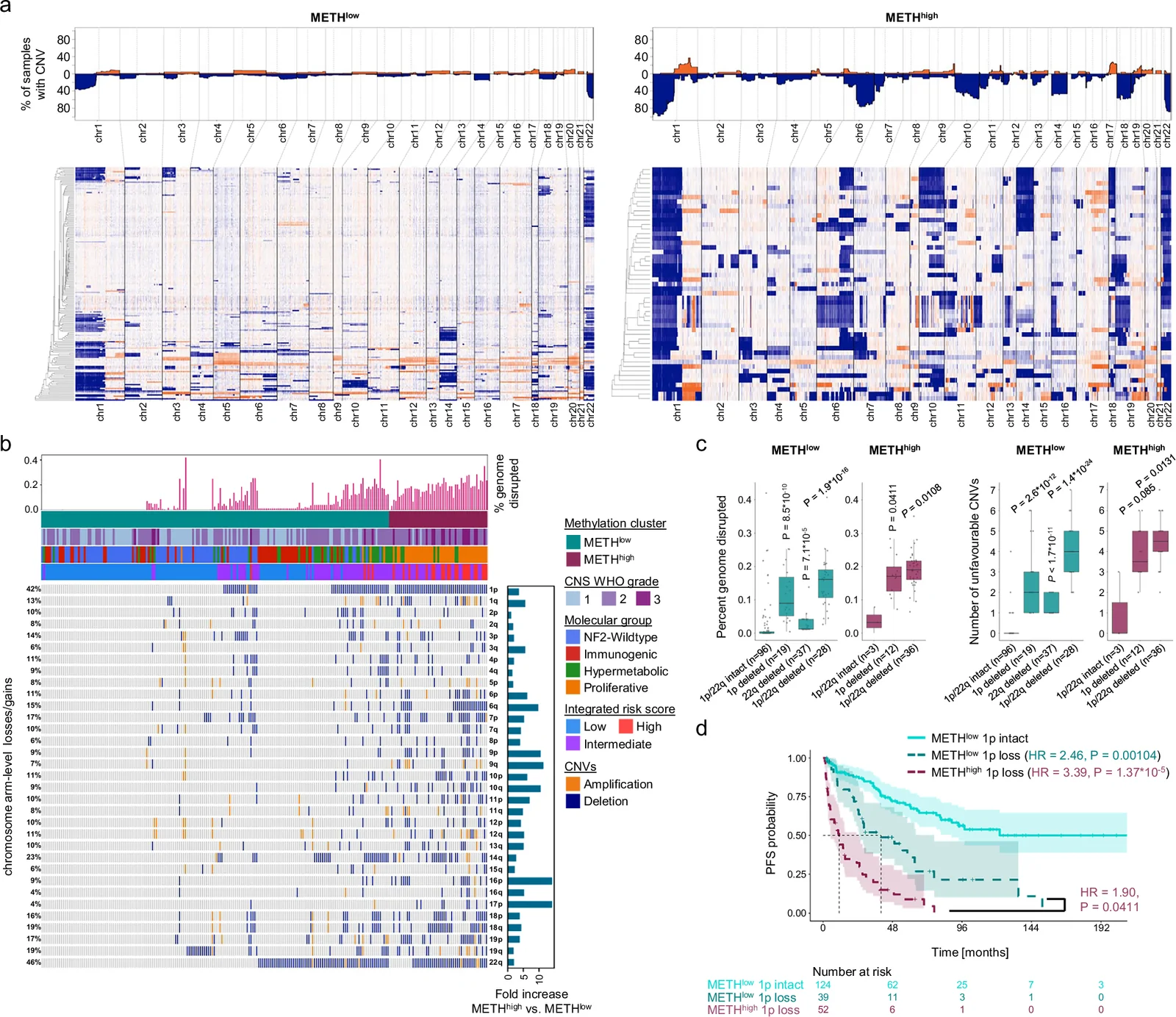
Independent contributions of DNA methylation cluster signature and CNVs on meningioma progression. **a** Summary genome plot illustrating the percentage of samples exhibiting CNVs in METH^high^ (*n* = 51) and METH^low^ (*n* = 180) meningiomas (top). Heatmap illustrates identified CNVs on a sample level (bottom). **b** Oncoprint showing recurrent chromosome arm gain or loss events (>10% of samples in either methylation cluster). Bar graph on top illustrates the overall percentage of the genome affected by CNVs for each sample. Bar graph on the right shows the fold change in incidence for each chromosome arm alteration in METH^high^ (*n *= 51) versus METH^low^ (*n* = 180) meningiomas. **c** Effect of 1p and 22q deletions on genome stability and number of unfavourable CNVs in METH^low^ and METH^high^ meningiomas. *P* values are derived from a Kruskal-Wallis test followed by two-sided Dunn’s post hoc test with Benjamini-Hochberg adjustment for multiple testing. 1p/22q intact was used as baseline reference. The sample size *n* is indicated within the panel. **d** PFS outcomes for samples from the Discovery cohort, stratified both by methylation cluster assignment and CNV status of chromosome 1p (METH^low^ 1p intact: *n* = 124; METH^low^ 1p loss: *n* = 39; METH^high^ 1p loss: *n* = 52). Hazard ratios and *P* values were calculated using two-sided Cox proportional regression modelling. For all boxplots, boxes represent the IQR with the median indicated by the centre line and whiskers extending to extreme outliers within 1.5x IQR. Source data are provided as a Source Data file.

To further analyse the relationship between methylation cluster assignment and CNVs in meningioma, we comprehensively investigated the landscape of CNVs in the Discovery cohort. In general, we found a higher incidence of CNVs in METH^high^ samples as compared to METH^low^, and this was consistent with data from the bigger UCSF cohort (Fig. 3a and Supplementary Fig. 13a). When accounting for tumour setting and methylation cluster assignment, we did not observe a significant effect of prior radiotherapy on overall CNV burden (Supplementary Fig. 13b). Across all grading schemes, we found an increasing degree of genome instability as well as an increasing number of unfavourable CNVs associated with adverse prognosis (Supplementary Fig. 13c,d). Genome instability alone or particular chromosome arm CNVs were insufficient to define METH^high^ samples (Fig. 3b). In fact, a substantial number (8.9%) of METH^low^ tumours exceeded the median genome instability found in METH^high^ tumours. In line with previous data^25,28^, loss of chromosome 1p was the best predictor for overall genome instability, even in the absence of 22q co-deletion (Fig. 3c). Upon multivariate analysis, loss of chromosome 1p remained significantly associated with disease progression even when accounting for other competing variables, and was the most prevalent chromosomal aberration in METH^high^ tumours (Supplementary Fig. 13e and Supplementary Data 3). When stratifying by both methylation cluster assignment and 1p status, we found that METH^low^ meningiomas with 1p deletion progressed significantly faster than METH^low^ meningiomas with intact chromosome 1 (Fig. 3d). However, METH^high^ meningiomas with loss of 1p, accounting for 94% of tumours in that cluster, showed a significantly higher risk of recurrence than all other 1p-deleted tumours. We did not observe a comparable stratification of 1p-deleted meningiomas by molecular groups, even though these analyses have to be seen with caution due to limited sample numbers (Supplementary Fig. 13f). Together, while CNVs are implicated in the course of meningioma progression, our data suggest that DNA hypermethylation in METH^high^ tumours is an independent mediator of meningioma progression, allowing for further prognostic sub-stratification of 1p-altered meningiomas.

### CNVs and DNA methylation shape the evolutionary trajectory of meningioma progression

Since our cross-sectional Discovery cohort lacks matched paired primary and recurrent meningioma samples, it does not allow any intra-patient and longitudinal analysis of molecular changes. To this end, we established a longitudinal cohort of 18 primaries and patient-matched first recurrences, with six patients contributing an additional second recurrence (Supplementary Data 4). Furthermore, to serve as a control population for bona fide benign meningiomas, we included primary meningiomas from the Discovery cohort with a landmark threshold of 10 years of PFS (*n* = 13), which substantially exceeded the cohort median PFS of 58.2 months (95% CI 40.1-78.3).

**Fig. 4:**
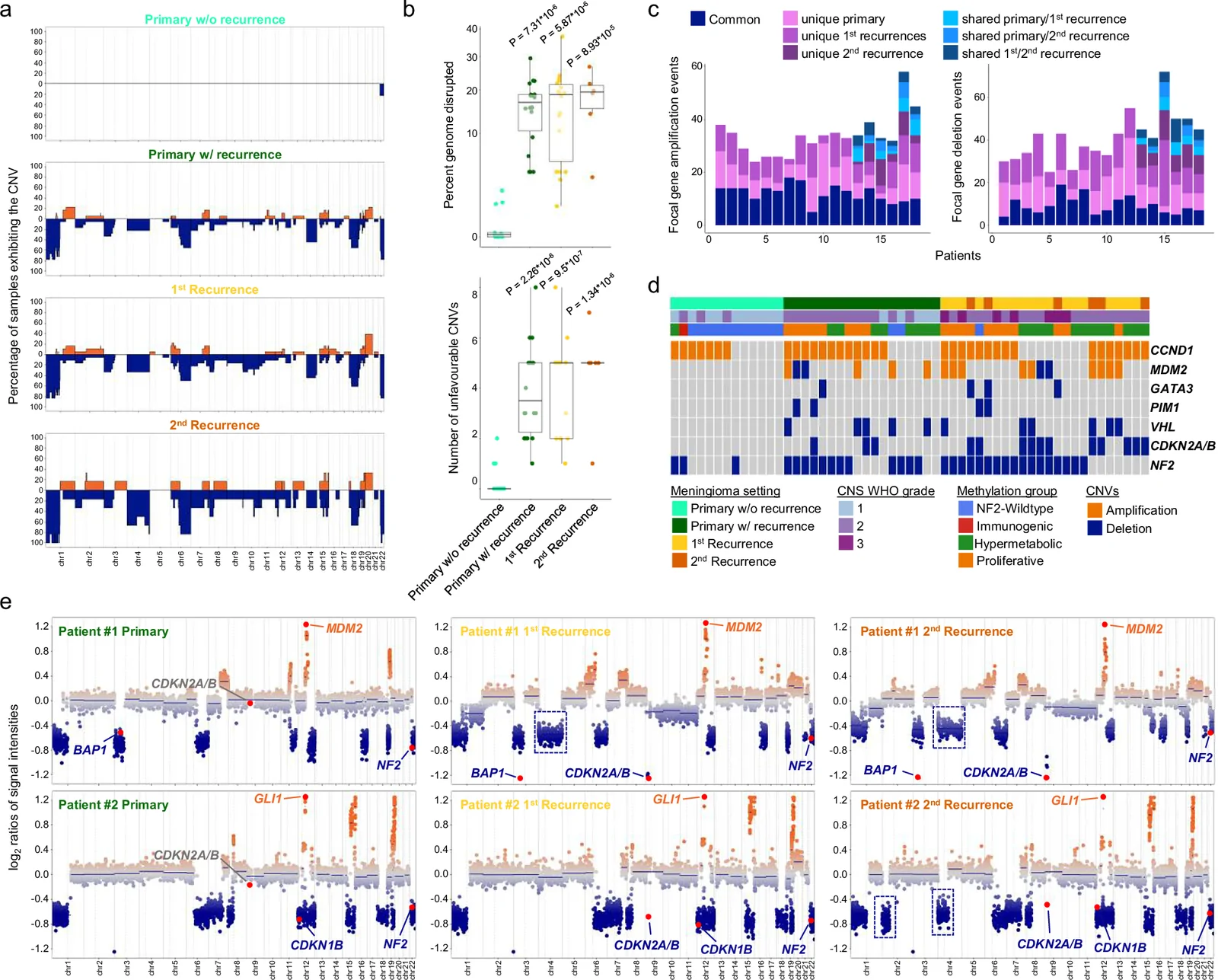
CNVs in longitudinal primary and corresponding recurrent meningioma samples. **a** Summary genome plots showing the percentage of samples exhibiting CNVs for distinct meningioma settings: control primaries without recurrence (Primary w/o recurrence, as determined by a landmark approach of minimum PFS of 10 years, *n* = 13), and patient-matched primaries (Primary w/recurrence, *n* = 18), first recurrences (1st Recurrence, *n* = 18), and second recurrences (2nd Recurrence, n = 6). **b** Differences in genome instability and number of unfavourable CNVs for meningioma samples from distinct settings given in **a** (*n* = 18 patients). Statistics are derived from a Type III F-test with Satterthwaite’s approximation, followed by two-sided Tukey-adjusted post hoc pairwise comparisons. **c** Interleaved bar plots illustrating common, unique, and shared CNVs affecting genes from the Cancer Gene Census in primary meningiomas and their recurrent counterparts. **d** Oncoprint showing arbitrarily selected genes affected by CNVs in primary and recurrent meningiomas (same sample size as in **a**). No statistical test was performed for gene selection. **e** Exemplary genome plots illustrating genomic regions affected by focal and broad CNVs in primary and recurrent meningiomas from two patients. For all boxplots, boxes represent the IQR with the median indicated by the centre line and whiskers extending to extreme outliers within 1.5× IQR. Source data are provided as a Source Data file.

Benign and progressive meningiomas displayed significant differences with regard to WHO grading (LRT *χ*^2^ = 56.99, *P* = 2.59*10^−12^) and molecular group affiliation (LRT *χ*^2^ = 34.601, *P* = 1.48*10^−7^) (Supplementary Fig. 14a,b). In particular, while clinical disease progression was associated with an increase in CNS WHO grading at first recurrence in 61% of cases, molecular group assignment stayed relatively stable with only 17% of recurrent progresses changing its assignment (Supplementary Fig. 14c). We found that primaries without disease progression are genomically stable without any major broad chromosomal aberrations, while recurrent meningiomas, both at first presentation as well as at recurrence, display a wide array of broad CNVs, consequently leading to greater genome instability and a higher number of unfavourable CNVs (Fig. 4a,b). Correlating samples based on their global CNV profiles suggested divergent development of recurrent tumours governed by their patient origin (Supplementary Fig. 14d). However, while the types of broad CNVs seemed largely similar between primaries and their recurrent counterparts, we detected a profound heterogeneity in focal CNVs affecting cancer-driving genes^29^, showing unique aberrations in both primaries and recurrent tumours (Fig. 4c). Screening for divergent CNVs between benign and progressive meningiomas, we identified several candidates to exclusively occur in recurrent tumours or their primary counterparts, but not in primaries that did not progress. These included gene amplifications of *MDM2*, a negative regulator of p53 signalling, and gene deletion events for tumour suppressors such as *VHL* and *CDKN2A/B* (Fig. 4d,e). Together, our data highlight the plasticity of focal and broad CNVs shaping the meningioma recurrence trajectory.

**Fig. 5:**
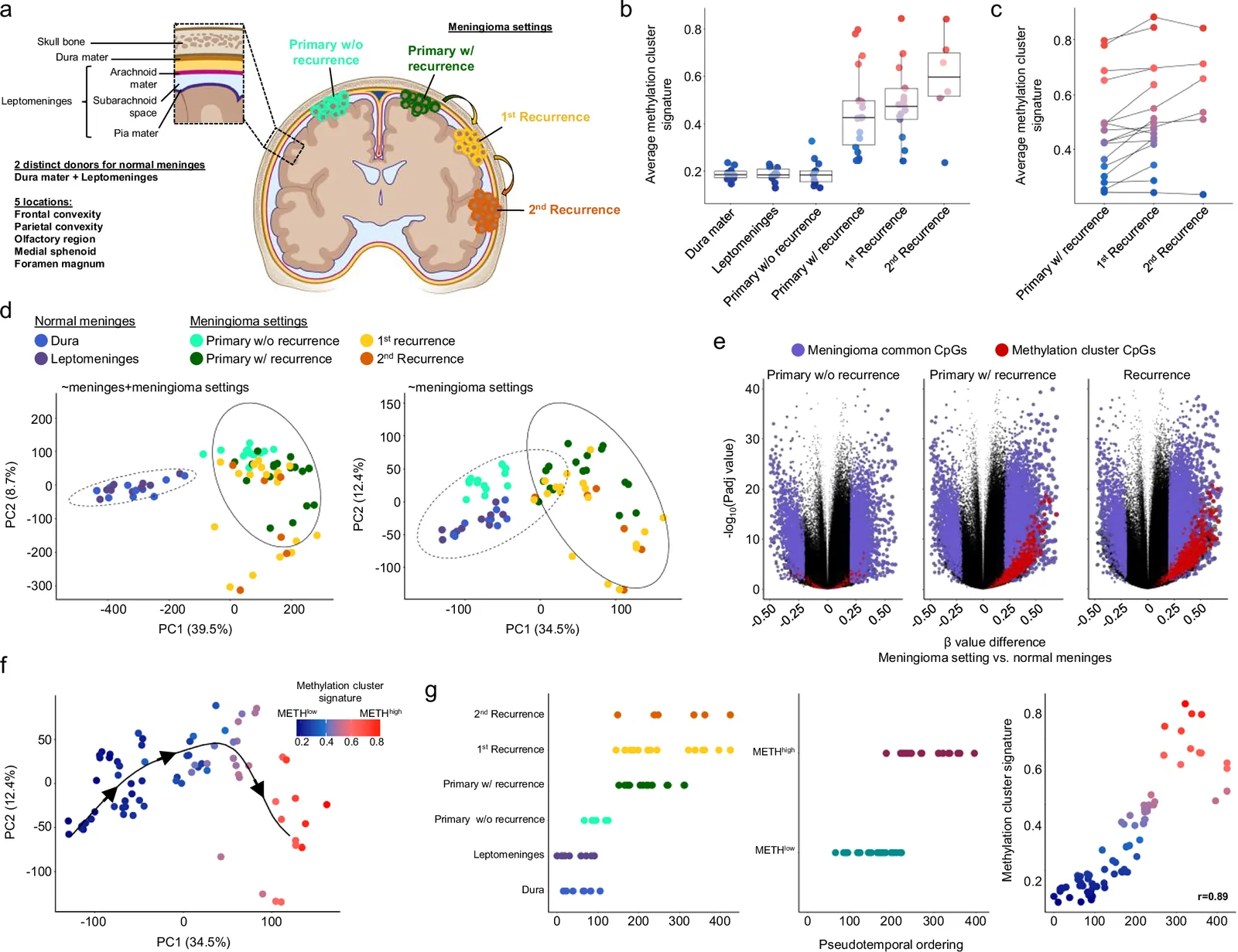
DNA methylation cluster signature recapitulates the trajectory of meningioma progression and discriminates between low and high-risk primary meningiomas. **a** Schematic overview of the longitudinal meningioma cohort incorporating normal meningeal tissue and benign meningiomas. Created in BioRender. Merk, D. (2026) https://BioRender.com/k5633fi. **b** Average methylation of CpG sites used to determine methylation cluster assignments (see Fig. 1c) for normal meninges (2 distinct donors, 5 different locations, total of *n *= 10 samples from both Dura mater and Leptomeninges) and different meningioma settings (Primary w/o recurrence, *n* = 13; Primary w/recurrence, *n* = 18; 1st Recurrence, *n* = 18; 2nd Recurrence, *n* = 6). **c** Lineplot illustrating the average methylation cluster signature in longitudinal meningioma samples from 18 patients. Each line corresponds to one patient. **d** PCA analyses of methylation data from normal meninges and different meningioma settings (same sample size as in **b**). Probes used for analysis were predefined by an F-test to significantly contribute to methylation differences between either any level of normal meninges and distinct meningioma settings (~meninges+meningioma settings, left) or only any level of meningioma settings (~meningioma settings, right). Ellipses illustrate the 95% confidence region of samples as determined by k-means clustering. **e** Volcano plots showing DNA methylation differences of distinct meningioma settings as compared to normal meninges. Probes determined to be differentially methylated across all meningioma settings as compared to meninges are shown in purple (Meningioma common CpGs). Probes defining methylation cluster assignment are indicated in red (Methylation cluster CpGs). *P* values are derived from Wald tests with Benjamini-Hochberg adjustment for multiple testing. **f** Trajectory analysis for normal meninges, benign, and progressive meningiomas based on DNA methylation profiles using *slingshot*. The activity of the methylation cluster signature along the trajectory is indicated by colour. **g** Order of samples along the trajectory based on cell identity or methylation cluster assignment, and its correlation with the activity of the methylation cluster signature. For all boxplots, boxes represent the IQR with the median indicated by the centre line and whiskers extending to extreme outliers within 1.5x IQR. Source data are provided as a Source Data file.

To properly mirror the entire evolutionary process of meningioma progression, we additionally gathered DNA methylation profiles of healthy human meninges^30^ to serve as a reference background for different meningioma settings (i.e. benign meningiomas or progressive primary meningiomas and their recurrences) (Fig. 5a). We found profound hypomethylation of methylation cluster-associated CpG sites in normal meninges and benign meningiomas, whereas malignant meningiomas were characterised by significant hypermethylation (Fig. 5b; Type III *F*-test with Satterthwaite’s approximation: *F*(5, 35.25) = 12.72, *P* = 4.27 × 10⁻⁷). Within longitudinal samples from individual patients, we did not see a clear pattern of increase in the degree of methylation for these probes (Fig. 5c).

We next assessed differential methylation along the developmental course of meningioma progression. With regard to global DNA methylation patterns, the dominant source of variance (39.5%) separated normal meninges from all meningiomas, suggesting major common changes in methylation patterns during meningioma development independent from benign or malignant meningioma biology (Fig. 5d,e). In contrast, differentially methylated probes within different meningioma settings clearly separated normal meninges and benign from aggressive meningiomas, with no clear separation of progressive primaries and their recurrences. Together, while common changes in DNA methylation discriminate between normal meninges and meningioma, we here provide further evidence for a heterogeneous, meningioma-specific hypermethylation pattern that defines METH^high^ cluster samples.

To further substantiate the correlation of the methylation cluster signature with disease progression, we applied trajectory inference analysis^31^ to characterise meningioma progression as a function of DNA methylation patterns, defining normal meninges as the starting point of the trajectory (Fig. 5f and Supplementary Fig. 15). When aligning meningeal tissue and meningioma samples to the inferred trajectory by assigning a pseudotime to each sample, we found that benign meningiomas localised to the early phase of the trajectory, overlapping with samples from normal human meninges (Fig. 5g). Progressive primaries and their recurrences were localized further downstream the trajectory, with no clear separation of these meningioma settings. Concordantly, the trajectory clearly separated meningioma samples according to their methylation cluster assignment, and the position on the trajectory correlated well with the methylation degree of the cluster signature probes.

### Long-range epigenetic silencing of clustered protocadherins drives meningioma progression

**Fig. 6:**
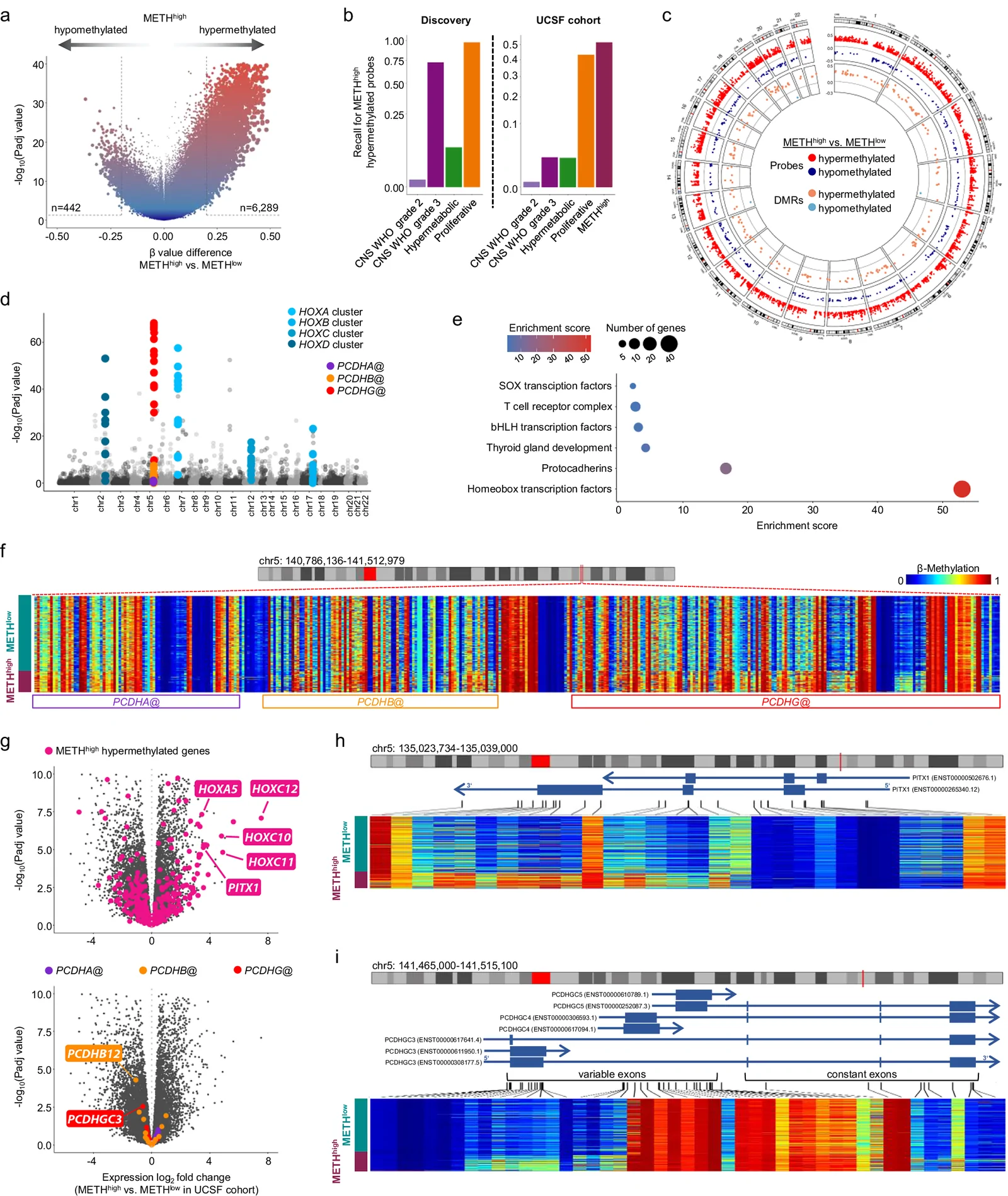
Meningioma progression is associated with long-range epigenetic silencing of protocadherin genes. **a** Volcano plot illustrating differentially methylated CpG sites when comparing METH^high^ (*n* = 51) versus METH^low^ (*n* = 180) meningiomas from the Discovery cohort. Two-sided t-tests were performed for the group coefficient, and *P* values were adjusted for multiple testing using the Benjamini-Hochberg false discovery rate procedure. **b** Recall of hypermethylated probes from METH^high^ meningiomas across various grading and molecular group contrasts associated with meningioma progression. Reference baseline for CNS WHO grade 2 (Discovery: *n* = 126; UCSF: *n* = 142) and grade 3 (Discovery: *n* = 24; UCSF: *n* = 35) were CNS WHO grade 1 (Discovery: *n* = 81; UCSF: *n* = 388) meningiomas. Reference baseline for Hypermetabolic (Discovery: *n* = 46; UCSF: *n* = 172) and Proliferative (Discovery: *n* = 46; UCSF: *n* = 95) cases were all NF2-Wildtype/Immunogenic (Discovery: *n *= 139; UCSF: *n* = 298) meningiomas. **c** Circos plot showing the genomic distribution of differentially methylated probes and regions (DMRs) in METH^high^ meningioma. **d** Manhattan plot illustrating the genomic location of significantly enriched genes targeted by hypermethylated probes in METH^high^ meningiomas. Enrichment was assessed using one-sided Fisher’s exact tests, and *P* values were adjusted using the Benjamini-Hochberg FDR procedure. **e** Top scoring enrichments from functional annotation of 744 hypermethylated genes in METH^high^ meningiomas. **f** Track view of methylation probes targeting the genomic locus encompassing *PCDHA@*, *PCDHB@*, and *PCDHG@* gene clusters on chromosome 5q31 in the Discovery cohort. **g** Volcano plots illustrating the log_2_ fold changes in gene expression between METH^high^ (*n* = 23) versus METH^low^ (*n* = 162) tumour samples in the UCSF cohort. Coloured genes have been found to be hypermethylated in METH^high^ samples from the Discovery cohort (top), or denote clustered protocadherins (bottom). Statistics are derived from two-sided Wald tests with Benjamini-Hochberg adjustments for multiple testing. **h** Track view of methylation probes targeting the *PITX1* gene locus for the Discovery cohort. Selected transcript positions are indicated. Note exclusive hypermethylation of 3’ exons in METH^high^ meningiomas. **i** Track view of methylation probes targeting the genomic locus encompassing subfamily C members of the *PCDHG@* gene cluster for the Discovery cohort. Selected transcripts illustrate variable positions for first exons of *PCDHGC3*, *PCDHGC4*, and *PCDHGC5*. Note hypermethylation of first 5’ exon of *PCDHGC3*. Source data are provided as a Source Data file.

To elucidate the mechanisms downstream of the DNA methylation cluster signature, we next determined all differentially methylated CpG sites in the METH^high^ samples as compared to METH^low^. In line with our previous clustering results, we found the vast majority (93%) of differentially methylated probes to be hypermethylated in METH^high^ samples (Fig. 6a and Supplementary Data 5). Hypermethylation of these probes was generally associated with aggressive meningioma behaviour as assessed by CNS WHO grade or molecular group, and this was also true for the larger UCSF validation cohort (Fig. 6b). Additionally, we found that this opposing methylation pattern was well recapitulated in individual molecular groups with clear low-risk or high-risk profiles, while in particular CNS WHO grade 1 tumours did not present the typical low-risk profile of our signature (Supplementary Fig. 16a). From a genomic point of view, differentially methylated probes seemed to be randomly distributed across the genome, with no particularly evident hotspot (Fig. 6c). We next assessed a potential over-representation of differentially methylated probes at specific gene loci. We identified a total of 23 and 744 genes that showed a significant over-representation of hypo- or hypermethylated CpG sites, respectively (Supplementary Fig. 16b,c and Supplementary Data 6). We noted that a substantial number of genes associated with DNA hypermethylation formed part of several gene clusters, including HOX genes and protocadherin clusters (Fig. 6d and Supplementary Fig. 16d). Further functional annotation supported a preferential mechanism involving dysregulation of several classes of transcription factors and protocadherins via DNA hypermethylation during the development of aggressive meningioma (Fig. 6e).

Long-range epigenetic silencing of protocadherin gene clusters (*PCDHA@*, *PCDHB@*, *PCDHG@*) has been shown for several solid tumours^32^, and our own data suggested that methylation alterations of clustered protocadherins might also contribute to disease progression in meningiomas (Fig. 6f). Thus, to further investigate the relationship of gene cluster methylation and expression of corresponding genes, we first carried out RNA sequencing of a small cohort of METH^high^ and METH^low^ meningiomas (*n* = 10; Supplementary Data 7). While the majority of hypermethylated genes did not show gene expression changes between METH^high^ and METH^low^ meningiomas (Supplementary Fig. 16e), we found distinct genes with a hypermethylation phenotype to be differentially expressed, including the homeobox transcription factor *PITX1* and several protocadherins such as *PCDHGC3*. Validating these initial findings in meningioma samples from the larger UCSF cohort with available expression and DNA methylation data (*n* = 185), we found a preferential upregulation of homeobox transcription factors including several HOX genes and *PITX1*, as well as robust downregulation of protocadherins such as *PCDHB12* and *PCDHGC3* in METH^high^ (*n* = 23) as compared to METH^low^ meningiomas (*n* = 162) (Fig. 6g, Supplementary Fig. 16f, and Supplementary Data 8).

We noted distinct methylation patterns for genes that were differentially expressed in between METH^high^ and METH^low^ meningiomas. Expression of the homeobox transcription factor *PITX1* was robustly enhanced in METH^high^ tumours, and this gene exhibited particular hypermethylation of gene body exons downstream of the 5’ transcription start site in these tumours (Fig. 6h). In contrast, several protocadherins such as *PCDHGC3* were strongly downregulated in METH^high^ tumours, and this gene displayed significant hypermethylation of the first exon only in the METH^high^ cluster (Fig. 6i). Closely related protocadherins from the C subfamily (*PCDHGC4* and *PCDHGC5*), sharing constant exons with *PCDHGC3* but presenting alternative first exons, presented robust hypermethylation in both METH^high^ and METH^low^ clusters. These findings suggested that the genomic context of DNA methylation may differentially influence transcriptional regulation in meningioma, and that selected protocadherins may act as tumour suppressors in meningioma.

**Fig. 7:**
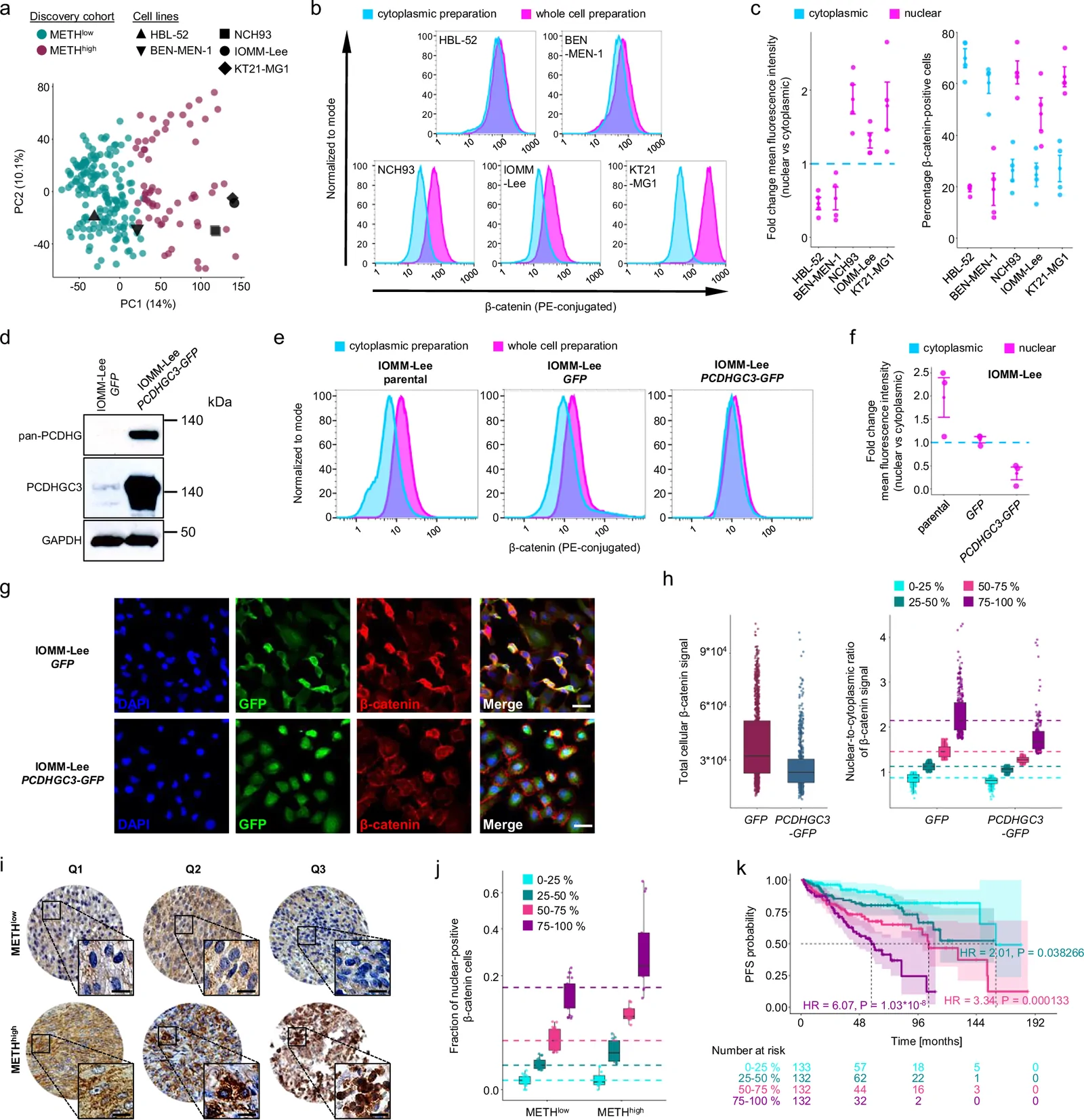
Tumour-suppressive functions of protocadherins associate with modulation of sub-cellular localisation of β-catenin. **a** PCA analyses of methylation data from the Discovery cohort with methylation cluster assignment indicated. Meningioma cell lines were plotted as supplementary variables. **b** Normalised density distributions of β-catenin signal from cytoplasmic or whole cell (nuclear + cytoplasmic) preparations of HBL-52, BEN-MEN-1, NCH93, IOMM-Lee, and KT21-MG1 cell lines using fluorescence associated cell analysis. Data shown are representative results from *n* = 4 experimental replicates. **c** Quantification of sub-cellular β-catenin signal (cytoplasmic or nuclear), measured as mean fluorescence intensity or expressed as percentage of β-catenin-positive cells. Dashed blue line illustrates the cytoplasmic reference intensity for fold change calculation. Error bars represent mean ± SEM (*n* = 4 experimental replicates per cell line). **d** Western blot analysis validating overexpression of PCDHGC3 in IOMM-Lee meningioma cells. Blots illustrate representative results from *n* = 3 experimental replicates. **e** Normalized density distributions of β-catenin signal from cytoplasmic or whole cell preparations of parental, control, and PCDHGC3-GFP-expressing IOMM-Lee cells. Data shown are representative results from *n* = 3 experimental replicates. **f** Quantification of cytoplasmic and nuclear β-catenin signal in parental, control, and PCDHGC3-GFP-expressing IOMM-Lee cells. Dashed blue line illustrates the cytoplasmic reference intensity for fold change calculation. Error bars represent mean ± SEM (*n* = 3 experimental replicates per condition). **g** Representative images from confocal microscopy of IOMM-Lee cells (GFP or PCDHGC3-GFP) illustrating a shift from nuclear to cytoplasmic β-catenin localisation upon overexpression of PCDHGC3 (*n *= 3 experimental replicates). Scale bars: 25 µm. **h** Quantification of total levels of β-catenin in control and PCDHGC3-overexpressing IOMM-Lee cells (left). Comparison of the nuclear-to-cytoplasmic ratio of β-catenin signal binned by quartile (right). Dashed lines represent the median of quartiles in the GFP condition. Data are derived from *n* = 3 experimental replicates. **i** Immunohistochemistry was performed on 80 METH^low^ and 49 METH^high^ human meningiomas on tissue microarrays (TMAs). Exemplary pictures show cases at the 25th, 50th, and 75th percentile with respect to β-catenin immunoreactivity ranking within each cluster. Scale bars: 25 µm. **j** Distribution of β-catenin immunoreactivity in METH^low^ (*n* = 80) and METH^high^ (*n* = 49) meningiomas based on the fraction of nuclear-positive β-catenin tumour cells. Dashed lines represent the median of quartiles in METH^low^ tumours. **k** PFS outcomes for 529 human meningiomas on TMAs. For stratification, meningiomas were binned by quartile according to the percentage of nuclear-positive β-catenin cells. The number of tumours per condition can be retrieved from the number at risk table. Hazard ratios and *P* values were calculated using two-sided Cox proportional regression modelling. For all boxplots, boxes represent the IQR with the median indicated by the centre line and whiskers extending to extreme outliers within 1.5x IQR. Source data are provided as a Source Data file.

Protocadherins interact with β-catenin via their intracellular protein domain and might regulate β-catenin indirectly as well^33,34^. Dysregulated WNT/β-catenin signalling is implicated in meningioma^35,36^, and long-range epigenetic silencing of protocadherins has been associated with dysregulation of β-catenin signalling in colorectal cancer^37^. To investigate and modulate a potential mechanism of protocadherin downregulation during meningioma progression, we first aimed to recapitulate our findings in a series of human meningioma cell lines, including models generated from both benign (HBL-52, BEN-MEN-1)^38,39^ as well as malignant meningiomas (NCH93, IOMM-Lee, KT21-MG1)^40–42^. In line with this clinical gradient, benign and malignant in vitro models showed divergent clustering with METH^low^ and METH^high^ tumours, respectively, based on global DNA methylation profiles (Fig. 7a and Supplementary Fig. 17a), and progressive hypermethylation of methylation cluster probes (Supplementary Fig. 17b-d).

Since β-catenin can act as a transcriptional co-activator within the nucleus, we first determined the cellular localisation of β-catenin in meningioma cell models employing differential cell permeabilization and flow cytometry (Supplementary Fig. 18a-c). In cell lines derived from benign meningiomas, we found significantly higher protein levels of β-catenin in the cytoplasm as compared to the nucleus (Fig. 7b,c). In contrast, cell lines derived from high-grade meningiomas showed preferential localisation of β-catenin in the nucleus.

Next, we overexpressed *GFP* alone or *PCDHGC3-GFP* in the malignant cell line IOMM-Lee, based on our previous results that this protocadherin is silenced by hypermethylation in high-grade meningiomas (Fig. 7d, Supplementary Fig. 18d, and Supplementary Information). Flow cytometry analyses suggested a profound change in localisation and a shift towards the cytoplasmic compartment for β-catenin upon overexpression of *PCDHGC3* in meningioma cells (Fig. 7e,f). In parallel, we also analysed β-catenin localisation in these cells using confocal microscopy (Fig. 7g). We found that overexpression of *PCDHGC3* significantly decreased total levels of cellular β-catenin (two-sided Mann-Whitney test, *P* < 2.2*10^−16^; Fig. 7h). In addition, our data revealed that the nuclear-to-cytoplasmic ratio of β-catenin was significantly lowered in meningioma cells with protocadherin overexpression (two-sided Mann-Whitney test, *P* = 5.97*10^−10^). In contrast, we did not detect any changes in the nuclear-to-cytoplasmic ratio of β-catenin when overexpressing *PCDHGC3* in the benign cell line BEN-MEN-1 (two-sided Mann-Whitney test, *P* = 0.9976) (Supplementary Fig. 19 and Supplementary Information). Together, these data suggested that protocadherins regulate the sub-cellular localisation of β-catenin.

To further determine whether differential β-catenin signalling was associated with methylation clusters and thus poor meningioma outcome, we first assayed β-catenin localisation using IHC on tissue microarrays containing 80 METH^low^ and 49 METH^high^ meningiomas from the Discovery cohort. While we found abundant cytoplasmic β-catenin staining in most meningiomas (Fig. 7i), nuclear staining was variable and significantly different between methylation clusters (two-sided Mann-Whitney test, *P* = 0.04557). METH^low^ tumours on average presented 5.1% nuclear-positive cells, with only 13.4% of tumours displaying nuclear β-catenin staining in more than 10% of tumour cells (Fig. 7j). In contrast, while METH^high^ tumours also had a considerable fraction of samples with low nuclear staining, an average of 11.4% of cells presented nuclear-positive for β-catenin across this methylation cluster, with 34.7% of tumours displaying nuclear β-catenin staining in more than 10% of tumour cells. Expanding on this analysis, we performed β-catenin IHC for an additional cohort of 529 meningiomas with clinical follow-up data. In line with previous results^35^, meningiomas with a high fraction of nuclear-positive β-catenin tumour cells had a significantly worse outcome as measured by progression-free survival (Fig. 7k). Together, we find that hypermethylation of clustered protocadherins facilitates epigenetic silencing and translocation of β-catenin to the nucleus, both of which are associated with poor clinical outcome for meningioma.

## Discussion

Characteristic DNA methylation patterns in cancer are a combination of both somatically acquired changes and an inheritance reflecting the cell of origin^43,44^. DNA methylation profiles segregate human meningiomas with distinct clinical outcomes into molecular groups with different underlying biology^18–20,22^. Similar usage of DNA methylation for sub-categorisation of umbrella tumour entities previously defined by histomorphological characteristics has been pioneered by studies on tumours such as medulloblastoma^45^, and has led to a methylation-based reference map for central nervous system tumours^24^. While being useful for classification purposes, it remains unclear to what extent distinct DNA methylation patterns are functionally relevant for the aggressive clinical behaviour of meningiomas.

Our study provides evidence for a DNA methylation signature that strongly correlates with poor clinical outcomes in meningioma patients and separates meningiomas in METH^low^ and METH^high^ cases using a binary cluster solution. Accordingly, methylation cluster assignment was largely in agreement with assignments to CNS WHO grades or molecular groups associated with bona fide low-risk or high-risk meningiomas. Furthermore, our data show that the binary cluster classification represents a surrogate for an underlying methylation gradient that is directly associated with a continuous, nonlinear risk gradient. In light of these findings, it is worth noting that the paradigm of previous assignments to distinct meningioma molecular groups has been recently challenged toward a tumour microenvironment-determined risk continuum^46^, and our data might present evidence for a tumour-intrinsic contribution to this biologically relevant gradient. As such, models including the continuous methylation signature significantly improved risk prediction beyond both CNS WHO grade and molecular group across our entire cohort. In contrast to clinically well-defined low-risk or high-risk meningiomas, we show that classifications with a considerable heterogeneity in their clinical course (CNS WHO grade 2, Hypermetabolic, intermediate integrated risk)^19,25,47^ showed a more balanced assignment to either METH^low^ and METH^high^ clusters. While we found that methylation cluster assignment successfully stratified outcomes of CNS WHO grade 2 tumours, it was unable to stratify Hypermetabolic or intermediate integrated risk meningiomas into groups with different clinical behaviour. However, these findings have to be seen in light of limited sample sizes in our cohort, in particular for METH^high^ tumours in these intermediate-risk meningiomas. Future studies in larger, multicenter cohorts are needed to improve resolution for these tumours.

We assumed that factors driving meningioma progression might either I) be driven by acquired changes along the trajectory of disease progression, or II) be present ab initio and might therefore discern benign and malignant meningiomas already at first presentation. Since no particular recurrent genetic alterations have been identified in high-grade meningiomas^10^, we aimed at investigating both CNVs as well as DNA methylation patterns in the context of longitudinal meningioma evolution. We found a high level of plasticity with regard to both broad chromosomal and gene-level CNVs during the progression of meningiomas. These included several alterations previously associated with clinically aggressive meningiomas, including deletions of chromosome 4^48^, *BAP1*^49^, and *CDKN2A/B*^16^. In contrast, cytogenetics and DNA methylation clearly separated malignant meningiomas from both normal meninges as well as meningiomas proven to present clinically benign behaviour, with malignant meningiomas characterised by the presence of several co-occurring broad CNVs and hypermethylation of METH^high^-associated probes. Thus, a combination of cytogenetic and DNA methylation aberrations is likely providing a suitable genetic background for the development of high-grade meningiomas, with further clonal selection of CNVs underlying the longitudinal progression of these tumours.

We further investigated the downstream mechanisms driven by focal hypermethylation in METH^high^ meningiomas. Among the 744 genes found to be significantly hypermethylated in the METH^high^ cluster, protocadherins, in particular those organised in gene clusters on chromosome 5, and several classes of transcription factors were among the top enriched functional categories. Our data suggest distinct modalities of hypermethylation resulting in either epigenetic silencing, as in the case of protocadherins, or gene activation, as shown for several transcription factors. In line with this, HOX genes have previously shown to be hypermethylated and concurrently upregulated in aggressive meningioma^50–54^. In contrast, we found hypermethylation of protocadherins to be associated with reduced gene expression, and this relationship has been demonstrated for several solid tumours^32^, supporting a tumour suppressive role for protocadherins across tumour entities. Tumour suppressive functions of family members of the *PCDHG@* gene cluster have been associated with negative regulation of WNT/β-catenin signalling^37,55^. This is of particular interest as deregulated WNT/β-catenin signalling has been implicated in the development and progression of meningiomas^28,35,56^. Of note, hypermethylation of all three protocadherin gene clusters has already been implicated as a potential proliferative biomarker associated with meningioma progression^57^, and we expand on these findings using a larger cohort. In particular, we now provide mechanistic insights showing that protocadherins from the *PCDHG@* gene cluster can regulate the subcellular localisation of β-catenin in meningioma cells. Furthermore, high levels of nuclear β-catenin staining correlated with poor clinical outcome in primary meningiomas. Thus, our data suggest a model where protocadherin expression conveys tumour suppressive functions by preventing translocation of β-catenin to the nucleus, and this mechanism can be attenuated by DNA hypermethylation and subsequent downregulation of protocadherin gene expression. While we focused here on the regulation of β-catenin signalling, it is tempting to speculate that loss of protocadherins, which have been shown to associate with several cytoskeletal and cell adhesion molecules^33^, might affect meningioma behaviour in other ways, e.g. by modulating contact inhibition of tumour cells. Thus, further research is needed to comprehensively dissect the role of protocadherins in meningioma development, potentially including further validation studies in suitable model system.

Our study reveals a prognostically relevant DNA methylation signature that defines a continuous, nonlinear risk gradient in meningioma. While methylation clusters defined by this signature are largely in line with previous classifications for bona fide low-grade and high-grade tumours, it may be particularly useful to predict clinical outcome for meningiomas currently classified as CNS WHO grade 2 and thereby inform clinical observation strategies in these patients accordingly. Within the framework of previous recommendations from the cIMPACT-NOW consortium^12^, the methylation cluster classification should be seen as a complementary dichotomised molecular risk label providing additional prognostic resolution. Accordingly, we recommend extended molecular testing using DNA methylation arrays for meningiomas with unclear prognostication, including histomorphologically borderline CNS WHO grade 1 and 2, CNS WHO grade 2 confirmation, unexpected rapid growth of low-grade tumours, brain-invasive meningioma otherwise benign, and tumours with known high-risk histology. While sparing patients at low risk of progression from adverse side effects of therapy, additional testing will also identify potentially underdiagnosed high-risk meningiomas which need closed-meshed surveillance time frames. In order to facilitate fast clinical translational in a real-world setting, we provide a ready-to-use web application that both predicts meningioma methylation cluster assignment and performs CNV analysis from Illumina DNA methylation arrays (https://mmcc.neurologie.uni-tuebingen.de).

## Methods

### Meningiomas and clinical data

The Independent Review Board of the University Hospital Tübingen and the Medical Faculty of the Eberhard Karls University of Tübingen, Germany, approved the collection and use of tumour material and clinical data for research purposes (application numbers 191/2021BO2, 116/2018BO2 and 456/2009BO2). Patients provided written informed consent to the use of tumour material and clinical data for research purposes. This consent includes publication of the associated data. Meningioma samples from the Discovery cohort were selected from patients treated at the University Hospital Tübingen. This cohort includes a total of 231 primary or recurrent meningioma samples, including 81 CNS WHO grade 1, 126 CNS WHO grade 2, and 24 CNS WHO grade 3 cases. The following clinical parameters were available for all samples: sex, age, clinical setting (primary vs. recurrent), 2021 CNS WHO grade, classification by histology, brain invasion status, extent of resection (EOR), status on prior and adjuvant radiotherapy, and H3K27me3 status. Information on sex was available based on self-report. Analysis of tissue histology, including estimation of tumour content as well as grading based on morphological features, has been performed by an experienced neuropathologist. Clinical follow-up was available for 219 meningiomas with a median follow-up of 35 months. Progression-free survival (PFS) was defined as the period between surgical resection and the time of progression (date of MRI indicating progression). The same definition of PFS was applied to both primary and recurrent tumours. Patients without progression were censored at the last follow-up. Across the Discovery cohort, 111 patients experienced disease progression during follow-up, either restricted to the local surgical bed (94.6%), both local and intracranially distant (3.6%), or only intracranially distant (1.8%). The Longitudinal cohort comprised a total of 42 meningioma samples from 18 patients. Each patient contributed a meningioma sample from first presentation (primary), and from first recurrence. For a total of six patients, a sample from the second recurrence was available. For all meningiomas included in our study, progression was verified by MRI imaging. A total of 129 samples included in the Discovery cohort were available as tissue microarrays used for immunohistochemistry. Furthermore, an additional 529 meningioma samples on tissue microarrays as part of the Tübingen meningioma cohort with clinical follow-up data were used for immunohistochemistry.

### Analyses of progression-free survival

Clinical follow-up was available for 219 out of 231 meningiomas from the Discovery cohort. Univariate and multivariate survival analyses were performed using the *survival* R package (v3.8-3) using Cox proportional hazards regression modelling. We performed survival analysis for each coefficient individually, and all variables scoring as significant in univariate analysis were included in a multivariate Cox proportional model. Cox proportional hazards assumption was tested using the *ggcoxzph* function implemented in the *survminer* R package (v0.5.0). The following covariates were included for modelling: age, sex, tumour setting, CNS WHO grade, extent of resection (EOR), brain invasion, adjuvant radiotherapy (RT), H3K27me3, and methylation cluster. Sex violated the assumption, and Cox models were accordingly stratified by sex to account for this violation. Sex was evaluated as a clinical covariate in survival analyses. However, the study was not designed or powered to assess sex-specific effects of the methylation signature, and no formal interaction analyses were performed. Adjuvant RT administered after baseline was modelled as a time-dependent covariate using counting-process methods (tstart-tstop methods) to properly account for the timing of treatment during follow-up. Furthermore, we stratified by tumour setting (primary vs. recurrent) in multivariate Cox regression models, assuming different baseline hazards for each group while estimating the effects of all other covariates across the full cohort. Nonlinear associations between the continuous DNA methylation cluster signature and risk of progression were modelled using restricted cubic splines with three knots, allowing flexible modelling of potential nonlinear relationships while avoiding overfitting. To assess the prediction performance of individual models, we employed both the concordance index (c-index) and the Brier score as implemented in the *survival* package. Additionally, we assessed the integrated discrimination improvement (IDI) and net reclassification improvement (NRI) using the *survIDINRI* R package (v1.1-2)^58^.

### DNA methylation profiling and analysis

For both the Discovery and the Longitudinal cohort, genomic DNA was extracted from FFPE material using the blackPREP FFPE DNA Kit (Innuscreen), and DNA was processed on the MethylationEPIC v2.0 BeadChip (Illumina) according to the manufacturer’s instructions at the Microarray Core Facility of the DKFZ in Heidelberg. Downstream analyses were performed in R (v4.4.2) with the *SeSAMe package* (v1.24.0). Samples with inferior quality, as assessed by abnormal median methylation intensities, unusual β value distribution and mean detection *P* value < 0.05, were excluded. Probes were filtered using the standard *SeSAMe* preprocessing pipeline consisting of normal-exponential out-of-band background normalisation, nonlinear dye bias correction, detection *P* value assessment using pOOBAH, and standard β methylation value calculation. All probes with missing data in any sample after probes masking were excluded from further analysis, generating a complete case dataset. Methylation probes targeting sex chromosomes were disregarded. After preprocessing and filtering, a total of 527,352 probes were retained for further analysis in the Discovery cohort.

We considered time of genomic DNA isolation, EPICv2 array runs, tumour content on tissue sections, and age of the paraffin block as potentially confounding technical artifacts. These potential technical confounders were evaluated using PERMANOVA on multivariate methylation distances, with PERMDISP used to assess homogeneity of dispersion. All factors identified to represent a real confounding factor were corrected using the *removeBatchEffect* function from the limma package in R.

Principal component analysis (PCA) was performed using the *prcomp* function in R with parameters centre = TRUE, and scale. = TRUE. We found that the total number of most meaningful components was not majorly affected by the number of top variable DNA methylation probes. In order to select for probes most associated with adverse clinical outcome, we investigated which principal component was best able to discriminate meningioma samples based on previous classification schemes predictive of clinical outcome, including CNS WHO grading^6^, molecular groups as defined by colleagues from Toronto^19^, and integrated risk score^25^. As a measure of discrimination alongside a given principal component, we calculated the weighted pairwise mean overlap of distributions of distinct grades, groups, and risk scores. This was done for the top 10,000, 50,000, and 200,000 most variable probes. The best separation of clinically relevant subgroups was seen alongside PC1 in the 10,000 probes dataset, which was used for all downstream analyses.

Probes contributing to PC1 were extracted and used for unsupervised hierarchical clustering. For several top-scoring probe subsets, the optimal number of clusters *k* was evaluated using both the Elbow method, based on within-cluster sum of squares, and the Silhouette method, based on average silhouette width across samples. Following cluster number selection, consensus clustering was applied to each probe subset using a resampling-based framework to evaluate clustering stability. ConsensusClusterPlus^59^ was used to generate consensus matrices and final cluster assignments, from which consensus cluster prediction (CCP) scores were calculated to quantify within-cluster stability. In addition, concordance of tumour cluster assignments across different probe subsets was assessed using the adjusted rand index. Findings on methylation clusters from the Discovery cohort were validated in a larger meningioma cohort (*n* = 565) from UCSF^20^, comprising 456 primary and 109 recurrent tumours, with 388, 142, and 35 cases being CNS WHO grade 1, 2, or 3, respectively.

Global DNA methylation profiles were used for the prediction of molecular-based classification of meningiomas as defined by previous groups. DKFZ groups^18^ were assigned using the Heidelberg CNS Tumour Methylation classifier (v12.8) previously available at www.MolecularNeuropathology.org. DKFZ groups and other classification schemes derived from DNA methylation data alone or from an integration of various data levels are referred to as molecular classifications within our manuscript^11^. When referring to molecular groups derived from DNA methylation patterns within our manuscript, we stick to the proposed nomenclature employed by colleagues from Toronto^19^ unless otherwise stated. We used a machine learning approach to assign meningiomas from our cohorts to Immunogenic, NF2-Wildtype, Hypermetabolic, and Proliferative molecular groups based on a previous cohort^19^. As a default, we selected the top 10,000 methylation probes to set up a classifier with the following algorithms available within the caret R package (v7.0-1): random forest, support vector machine, k-nearest neighbours, nearest shrunken centroids and stochastic gradient boosting. The final model was selected based on the highest accuracy. The selected classifier was then applied to our human meningiomas. Methylation cluster assignment for meningioma samples outside of our Discovery cohort was assigned accordingly. Models were trained and evaluated using 10-fold cross-validation implemented in the caret package, with accuracy used as the primary tuning metric and Cohen’s kappa reported as a complementary measure of agreement. For binary outcomes, discriminative performance was further assessed using standard receiver operating characteristic (ROC) analysis. For multiclass outcomes, ROC analysis was performed using a one-vs-rest strategy with micro-averaging, based on cross-validated hold-out predictions. Integrated risk scores were calculated as previously described^25^.

For integrated analyses of normal meninges and meningiomas, data from meningeal tissue of both dura and leptomeninges covering five different intracranial locations from two healthy donors was retrieved^30^. Only probes present on the EPIC (meninges) and EPIC v2.0 array (meningiomas) were used. Prior to PCA analyses, we defined probes that significantly contribute to the variance between normal meninges and meningioma settings (Primary w/o recurrence, Primary w/ recurrence, and recurrence) (*n* = 113,047), or only in between meningioma settings (*n* = 19,142), using F-test global statistics. K-means clustering of samples was performed using the *fviz_nbclust* function of the *factoextra* R package (v1.0.7). Differential DNA methylation in meningioma methylation clusters, or in between meningiomas, as compared to normal meninges, was assessed using the *DML* function implemented in the *SeSAMe* package. These analyses were conducted at the single-CpG level using previously calculated β-values. Statistical significance was assessed with *P* values adjusted for multiple testing using the Benjamini-Hochberg false discovery rate. CpG sites with an absolute estimated β-value difference of 0.2 for a given contrast and adjusted *P* value of <0.05 were considered differentially methylated. Trajectory analysis in PCA plots defined by DNA methylation probes varying between meningioma settings and corresponding pseudotime analyses were performed using the *slingshot* package (v2.14.0)^31,60^. As an additional sensitivity analysis, we computed patient-level centroids of samples in PCA space and used those for trajectory inference to account for non-independence of samples from the same patient.

### Copy number variant analysis

CNV profiles from DNA methylation data were generated using the conumee2 (v2.1.2)^26^ package in R. CNVs were estimated by comparing samples from the Discovery or the Longitudinal cohort to a series of 121 normal CNS tissue samples^24^ and calculation of log_2_ ratios of signal intensities. We considered all absolute ratios >0.3 a potential CNV. For broad chromosomal aberrations, we considered CNVs covering at least 5% of the corresponding chromosome arm^61^. Unfavourable broad CNVs were defined as previously described^11^. CNVs in large cohorts were summarised using the *CNV.summarplot* and *CNV.heatmap* functions from conumee2. To correlate CNV profiles with clinical behaviour, we considered overall genome instability or the presence of several unfavourable CNVs previously described^11^. We used the *CNV.focal* function to reveal genes affected by CNVs in each meningioma sample. Due to the high number of genes affected in most malignant meningioma samples, we focused the analysis of gene-specific CNVs on genes from the Cancer Gene Census^29^. For correlation analyses, genomes were binned by 300,000 bp segments, and log_2_ signal intensity ratios were calculated for each bin of each sample as compared to normal CNS tissue samples. Pairwise pearson correlation coefficients were calculated based on intensity ratios.

### TERT promoter sequencing

To analyse *TERT* promoter hotspots (c.−124C>T (C228T), c.−146C>T (C250T)), targeted multigene mutation screening was performed by Next Generation Sequencing (Ion GeneStudio S5, Thermo Fisher Scientific, Waltham, MA, USA) using an AmpliSeq Custom panel (hotspot regions in *BRAF*, *CTNNB1*, *GNA11*, *GNAQ*, *KIT*, *KRAS*, *MAP2K1*, *NRAS*, *PDGFRA*, and *TERT* (promoter region)). To analyse *TERT* hotspots solitarily, only pool 1 of the panel was used. Amplicon library preparation and semiconductor sequencing were done according to the manufacturers’ manuals using the Ion AmpliSeq Library Kit v2.0, the Ion Library TaqMan Quantitation Kit on the QuantStudio 5 (Thermo Fisher Scientific), the Ion 540 Kit – Chef on the Ion Chef, and the Ion 540 Chip Kit (Thermo Fisher Scientific). Output files were generated with Torrent Suite 5.18. Variants were visualised using the Integrative Genomics Viewer (IGV; Broad Institute, Cambridge, MA; Version 2.19.7) to detect *TERT* mutations.

### Gene expression profiling

Gene expression profiling was performed for a total of 10 meningioma samples, five METH^low^ and five METH^high^ cases. Total RNA was isolated from FFPE meningioma tumour material using the RNeasy FFPE Kit (Qiagen). For RNA sequencing, mRNA fraction was enriched using polyA capture from 200 ng of total RNA using the NEBNext Poly(A) mRNA Magnetic Isolation Module (NEB). Next, mRNA libraries were prepared using the NEB Next Ultra II Directional RNA Library Prep Kit for Illumina (NEB) according to the manufacturer’s instructions. The libraries were sequenced as paired-end 50 bp reads on an Illumina NovaSeq6000 (Illumina) with a sequencing depth of approximately 25 million clusters per sample. RNA raw data QC and processing were performed using megSAP (version 0.2-135-gd002274) combined with ngs-bits package (version 2019_11-42-gflb98e63). Reads were aligned using STAR v2.7.3a to the GRCh37, and alignment quality was analysed using ngs-bits. Normalized read counts for all genes were obtained using Subread (v2.0.0) and edgeR (v3.26.6). Further details can be found under https://nf-core.re/rnaseq. Raw count data was analysed using the DESeq2 (v1.46.0)^62^ pipeline using standard protocols. For validation of gene expression findings, meningioma samples from the UCSF cohort^20^ with available gene expression data and methylation data to predict methylation cluster assignment (*n* = 185) were used and analysed using DESeq2.

### Immunohistochemistry of tissue microarrays (TMAs)

Immunohistochemical staining for beta catenin (clone 14, BD Transduction Laboratories #610153, 1:500) on TMA FFPE samples was carried out on a Ventana BenchMark immunostainer (Ventana Medical Systems, Tucson, Arizona, USA) using the OptiView immunohistochemical staining methodology. Heat Induced Epitope Retrieval consisted of cell conditioner CC1 pretreatment for 32 min. The primary antibody was incubated at 37 °C for 32 min. Antigen-antibody reaction was visualised using the Ventana OptiView Universal DAB Detection kit (OptiView Linker 8 min, HRP Multimer 8 min, H202/DAB 8 min, Copper 4 min). All slides were then counterstained with haematoxylin for 2 min. Histology and immunohistochemistry slides were scanned with a Philips Pathology SG60 scanner and exported as stitched TIFF files for further processing. Scans were further analysed in QuPath (v0.5.1). TMAs were de-arrayed using a grid and annotated. DAB signal was further quantified using default settings of QuPath’s positive cell detection mode. This includes nucleus detection using hematoxylin counterstain, cell expansion from the nucleus to estimate whole cell area, and compartment measurement to quantify nuclear and cytoplasmic intensities.

### Cell culture studies

Stable cell lines generated from benign (HBL-52, BEN-MEN-1) and malignant meningiomas (NCH93, IOMM-Lee, KT21-MG1) were used in the study. All cell lines were authenticated by STR profiling. All cell lines were regularly tested negative for mycoplasma. IOMM-Lee cells were purchased from ATCC, BEN-MEN-1 cells were purchased from DSMZ, HBL-52 cells were purchased from Cytion. NCH93 and KT21-MG1 cells were provided by Christel Herold-Mende (University of Heidelberg, Germany) and Christian Mawrin (University of Magdeburg, Germany), respectively. The following media formulation were used: DMEM, 10% FCS, 0.1% gentamycin (NCH93, IOMM-Lee, KT21-MG1), DMEM, 20% FCS, 0.1% gentamycin (BEN-MEN-1), and DMEM/F12, 1x ITS, 10% FCS, 0.1% gentamycin. For overexpression studies, we generated lentiviral particles in HEK293FT cells coding for GFP alone (Addgene #2211811) or in combination with the *PCDHG@* member PCDHGC3 (HorizonDiscovery #OHS5897-202619779) and transduced IOMM-Lee and BEN-MEN-1 cells. GFP-positive cells were selected for using fluorescence-associated cell sorting using a BD FACS Diva 9.0.1 machine. For all in vitro cell culture studies, genetic alteration experiments were performed in *n* = 3 experimental replicates based on different cell stocks and experiments repeated at different time points.

### Flow cytometry

The cells were harvested using trypsin and washed in cold PBS by centrifuging at 4 °C at 1200 rpm for 5 min. In order to preserve the cellular structure, the cells were fixed with 4% paraformaldehyde (PFA) in PBS for 15 min at RT, followed by two washing steps in PBS. Next, the cells were divided into two equal proportions for cytoplasmic or whole cell staining. For cytoplasmic staining, we used a mild detergent, 0.1% saponin, that permeabilizes the plasma membrane, for 15 min at RT. For whole cell staining, we resuspended the cells in 0.5% Triton X-100 in 1x FOXP3 permeabilization buffer (Invitrogen, Thermo Fisher Scientific) for 10 min at RT. The cells were then washed and incubated with β-catenin antibody (15B3, PE-conjugated, Invitrogen, 1:50) for 30 min at RT. Finally, the cells were washed and resuspended in 200 µL of FACS buffer (PBS containing 2% FCS and 0.5 M EDTA) and analysed using a MACSQuant Analyser 10 (Miltenyi Biotec). All flow cytometry data were analysed using FlowJo version 10.10.0. FMOs (fluorescence minus one) were used as negative controls for proper gating. Exclusive nuclear-only staining as mean fluorescent intensity (MFI) or percentage of positive cells was calculated on the basis of average values from each replicate by subtracting cytoplasmic from whole cell values.

### Confocal microscopy

IOMM-Lee and BEN-MEN-1 cells expressing either GFP or PCDHGC3-GFP were seeded in Lab-Tek 4 chamber slide systems. After 24 h, cells were fixed with PFA for 30 min, permeabilized with 0.1% Triton X-100 in PBS, and blocked in 2% BSA in PBS for 1 h. Primary antibodies against GFP (ab13970, Abcam, 1:500, chicken) and β-catenin (D10A8, Cell Signaling, 1:100, rabbit) were incubated overnight. Cells were incubated with coupled secondary antibodies (donkey anti-rabbit IgG (H + L) Alexa Fluor 555, Invitrogen, 1:500; goat anti-chicken IgY (H + L) Alexa Fluor 647, Jackson ImmunoResearch, 1:500) for 1 h, washed, stained with DAPI, and mounted. Images of fluorescently-labelled cells were acquired using a spinning disk confocal microscope (Cytation C10, BioTek) and quantified with the cellular analysis mode of Gen5 software (version 3.16, BioTek). To analyse region-specific fluorescence signal intensities, a primary mask was generated based on DAPI staining to define cell nuclei, and a secondary mask was applied to encompass the surrounding cytoplasmic region. Mean fluorescence intensity was measured separately within the nuclear (primary) and cytoplasmic (secondary) masks to assess spatial differences in signal distribution. Fluorescence intensity values were exported and further analysed using R for statistical evaluation.

### Western blot analysis

Whole cell lysates were prepared using either RIPA (25 mM Tris-HCl pH 7.6, 150 mM NaCl, 1% NP-40, 1% sodium deoxycholate, 0.1% SDS). Protein gels were run using 4-12% NuPAGE Bis-Tris polyacrylamide precast gels. Proteins were blotted onto PVDF or nitrocellulose membranes and detected using the indicated antibodies as per standard methods. Visualisation was done using HRP-coupled, species-specific secondary antibodies. Primary antibodies used in this study for western blotting were the following: anti-PCDHG-pan-gamma (S159-5, Thermo Fisher, 1:500), anti-PCDHGC3 (MAB8364, R&D, 1:250), and anti-GAPDH (D4C6R, Cell Signaling, 1:1000).

### Statistics and reproducibility

No statistical methods were used to predetermine sample sizes, but our Discovery and Longitudinal cohort sizes are similar to or larger than those reported in previous publications^19,22^. Data distributions were tested for normality using the Shapiro-Wilk normality test, and parametric or non-parametric tests were used accordingly. Results were compared using Student’s *t* test, *χ*^2^ test, Mann-Whitney test, Wilcoxon signed-rank test for paired data, or Wald test. For comparisons involving repeated measures, differences between conditions were tested using a linear mixed-effects model for continuous data types, with condition as a fixed effect and subject as a random effect, using a Type III F-test to assess the statistical significance of the condition effect. Pairwise comparisons between conditions were performed using estimated marginal means with Tukey adjustment for multiple testing. For categorical data such as WHO grades involving repeated measures, we used an ordinal mixed-effects model and used a likelihood ratio test to test significance. Investigators for functional studies on β-catenin were blinded to conditions during analysis. All box plots show distributions and underlying data points. Boxes are defined as follows: centre line, median; box limits, upper and lower quartiles; whiskers, extremes within 1.5× interquartile range.

### Reporting summary

Further information on research design is available in the Nature Portfolio Reporting Summary linked to this article.

## Supplementary information


Supplementary Information
Description of Additional Supplementary Information
Supplementary Data
Reporting Summary
Transparent Peer Review file


## Source data


Source Data


## Data Availability

The raw DNA methylation data and RNAseq data generated in this study have been deposited in the GEO database under accession code GSE304097. Publicly available datasets used in this study include DNA methylation array and RNAseq data (https://www.ncbi.nlm.nih.gov/geo/query/acc.cgi?acc=GSE180061 and https://www.ncbi.nlm.nih.gov/geo/query/acc.cgi?acc=GSE183656)^19,20^. Raw DNA methylation data from the normal meningeal samples^30^ cannot be deposited in a public or third-party controlled-access repository because the data derive from only two individuals who donated their bodies through the University of Copenhagen Body Donation Programme. Their use is governed by the donor framework, institutional stewardship requirements, and project-specific data-sharing agreements that do not permit onward transfer or repository deposition without separate authorisation. The data are available through a controlled-access procedure administered directly by Andrea Daniela Maier and Tiit Mathiesen from the University of Copenhagen, Denmark. Access may be granted to qualified researchers at recognised academic or non-profit research institutions when the proposed use is compatible with the donor and institutional framework and the applicant agrees to a project-specific Data Use Agreement. Requests should be submitted in writing to and andrea. maier@regionh.dktiit.illimar. mathiesen@regionh.dk. Requests will be acknowledged within five working days and decisions will be provided within 20 working days after receiving all required documentation. Approved access will normally be granted for 12 months and may be renewed following review. Additional preprocessed data of DNA methylation arrays and a folder structure compatible with our submitted code are available on figshare (https://doi.org/10.6084/m9.figshare.32043249)^63^. The remaining data are available within the Article, Supplementary Information or Source Data file. Source data are provided with this paper.

## Code availability

All code used in this study is publicly available at GitHub (https://github.com/MerkLabHIH/Meningioma_DNA_methylation_project).
