# Supplementary material for: DNA methylation profiling identifies long-range epigenetic silencing of clustered protocadherins as a key determinant of meningioma progression
Source: Nat Commun. 2026 Aug 29;17:9236. doi: 10.1038/s41467-026-77170-3 (PMC13526022; doi:10.1038/s41467-026-77170-3)
Supplement: Supplementary file 1 [file 41467_2026_77170_MOESM1_ESM.pdf]

Supplementary Information

DNA methylation profiling identifies long-range epigenetic silencing of clustered protocadherins as a key determinant of meningioma progression

Daniel J. Merk[1,2], Peter Paßlack[1,2], Surender Surender[1,2], Foteini Tsiami[1,2,3], Lara A. Haeusser[1,2], Vanessa Arnold[1,4,5], Vijayasarathy Sampath-Kumar[1,4,5], Lisa Sevenich[1,2,4,6], Andrea D. Maier[7], Tiit Mathiesen[7], Marcos Tatagiba[8], Hanna Gött[8], Jonas Tellermann[8], Felix Behling[8], Jens Schittenhelm[9,10], Hannes Becker[1,2,8], and Ghazaleh Tabatabai[1,2,6,10*]

[1]Department of Neurology and Interdisciplinary Neuro-Oncology, Hertie Institute for Clinical Brain Research, University Hospital Tübingen, Eberhard Karls University Tübingen, Tübingen, Germany

[2]Cluster of Excellence iFIT (EXC 2180) "Image Guided and Functionally Instructed Tumour Therapies", Eberhard Karls University Tübingen, Tübingen, Germany

[3]Department of Pediatric Oncology, Dana-Farber/Boston Children's Cancer and Blood Disorders Center, Boston, MA, USA

[4]M3 Research Center for Malignome, Metabolome, and Microbiome, University Hospital Tübingen, Eberhard Karls University Tübingen, Tübingen, Germany

[5]Graduate Training Center for Neuroscience, Eberhard Karls University Tübingen, Tübingen, Germany

[6]German Consortium for Translational Cancer Research (DKTK), Partner Site Tübingen, German Cancer Research Center (DKFZ), Heidelberg, Germany

[7]Department of Neurosurgery, Copenhagen University Hospital, Rigshospitalet, Copenhagen, Denmark

[8]Department of Neurosurgery and Neurotechnology, University Hospital Tübingen, Eberhard Karls University Tübingen, Tübingen, Germany

[9]Institute of Neuropathology, Department of Pathology and Neuropathology, University Hospital Tübingen, Eberhard Karls University Tübingen, Tübingen, Germany

[10]Comprehensive Cancer Center Tübingen Stuttgart, University Hospital Tübingen, Eberhard Karls University Tübingen, Tübingen, Germany

*Corresponding author:

Ghazaleh Tabatabai, MD PhD, Department of Neurology and Interdisciplinary Neuro-Oncology, Hertie Institute for Clinical Brain Research, University Hospital Tübingen, Eberhard Karls University Tübingen, Tübingen, Germany, Hoppe-Seyler-Str 3, 72076 Tübingen, E-mail: ghazaleh.tabatabai@uni-tuebingen.de

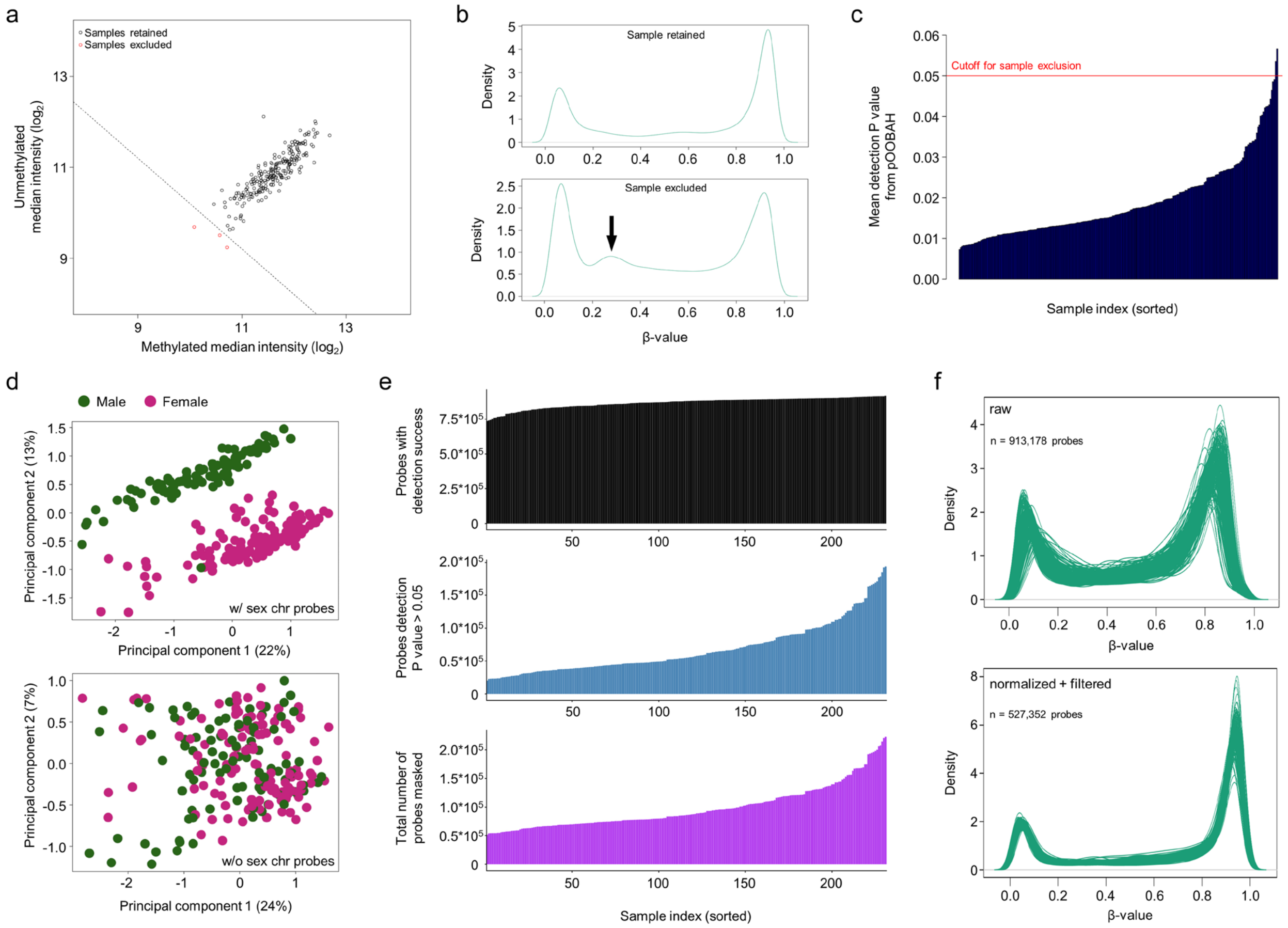

Supplementary Fig. 1: Quality control of DNA methylation arrays. a-c Sample-level quality assurance of methylation arrays from meningioma samples in a provisional Discovery cohort (n=237). Samples were excluded based on abnormal median methylation intensities (n=3) (**a**), unusual β methylation value distribution (n=1) (**b**), or insufficient signal quality as judged by detection *P* values (n=2) (**c**). The final Discovery cohort contains a total of n=231 meningioma samples. **d** Multidimensional scaling analyses illustrating the effect of methylation probes targeting sex chromosomes on sample clustering. As a default, all sex chromosome probes were masked from further analyses. **e** Probe-level preprocessing and masking of probes was performed in batch for all meningioma samples using the *openSesame* pipeline[1], employing normalization/background correction using Noob and detection *P* value estimation using out-of-band array hybridization (pOOBAH). Bar charts illustrate for all samples: total number of successfully detected probes (top), number of probes with detection P value > 0.05 (middle), and total number of probes masked (bottom). **f** Density distributions of β values for the raw dataset and after pre-processing and filtering. Source data are provided as a Source Data file.

a

PERMANOVA results before correction

Factor	Df	SumOfSqs	R^2	F	P value
DNA isolation	19	12,250,436	0.26891	4.0848	0.001
Array run	34	17,268,996	0.37908	3.5194	0.001
Tumor content	8	1,390,016	0.03051	0.8734	0.612
FFPE age	1	4,164,800	0.09142	23.043	0.001

b

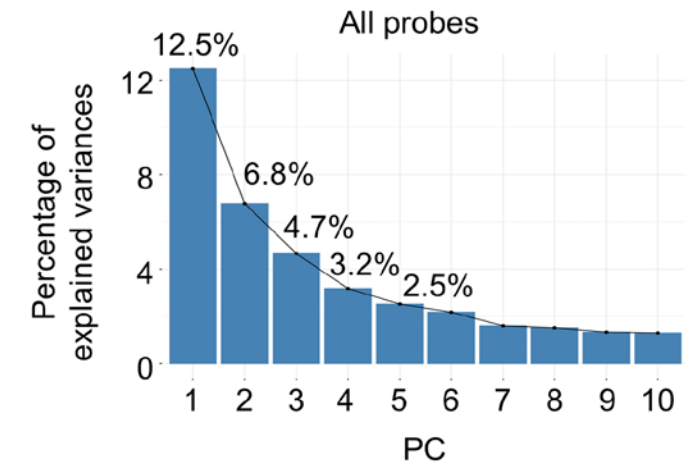

c

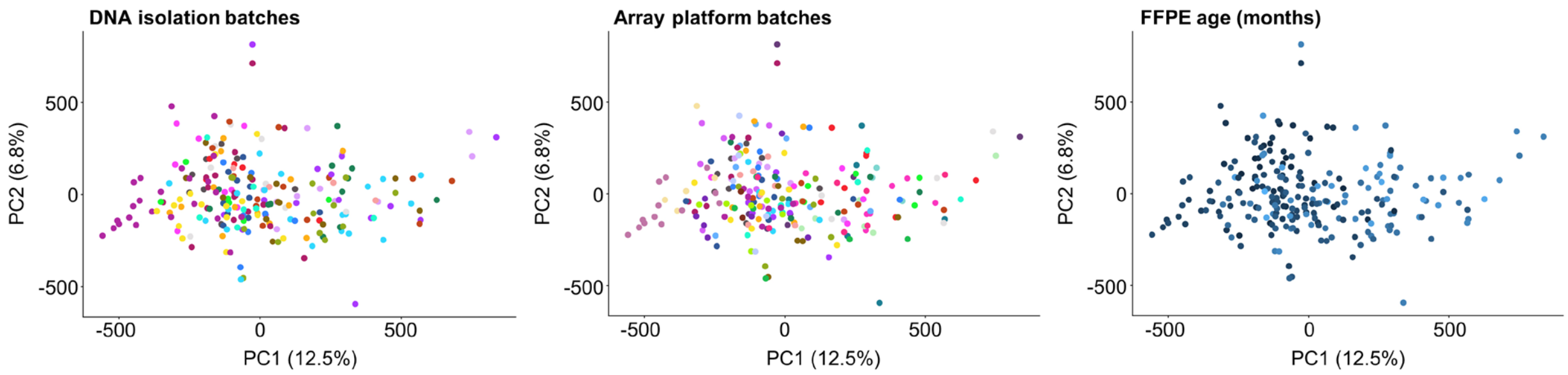

d

PERMANOVA results after limma::*removeBatchEffect*

Factor	Df	SumOfSqs	R^2	F	P value
DNA isolation	19	2,077,732	0.05	0.5845	1
Array run	34	2,795,265	0.06727	0.4158	1
Tumor content	8	1,673,111	0.04026	1.1642	0.192
FFPE age	1	76,520	0.00184	0.4225	0.896

e

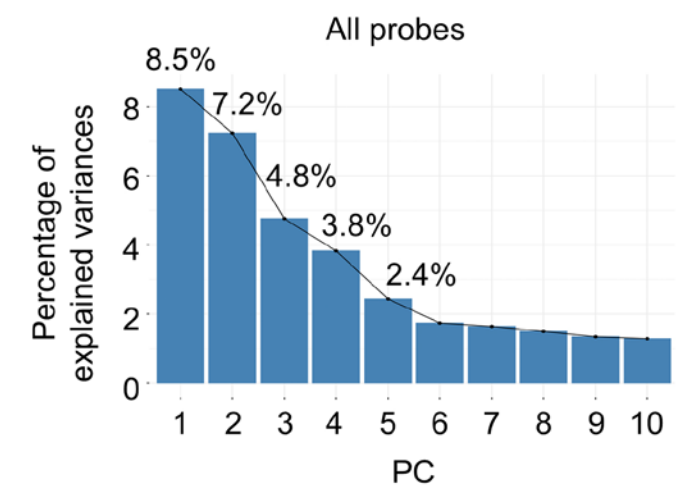

f

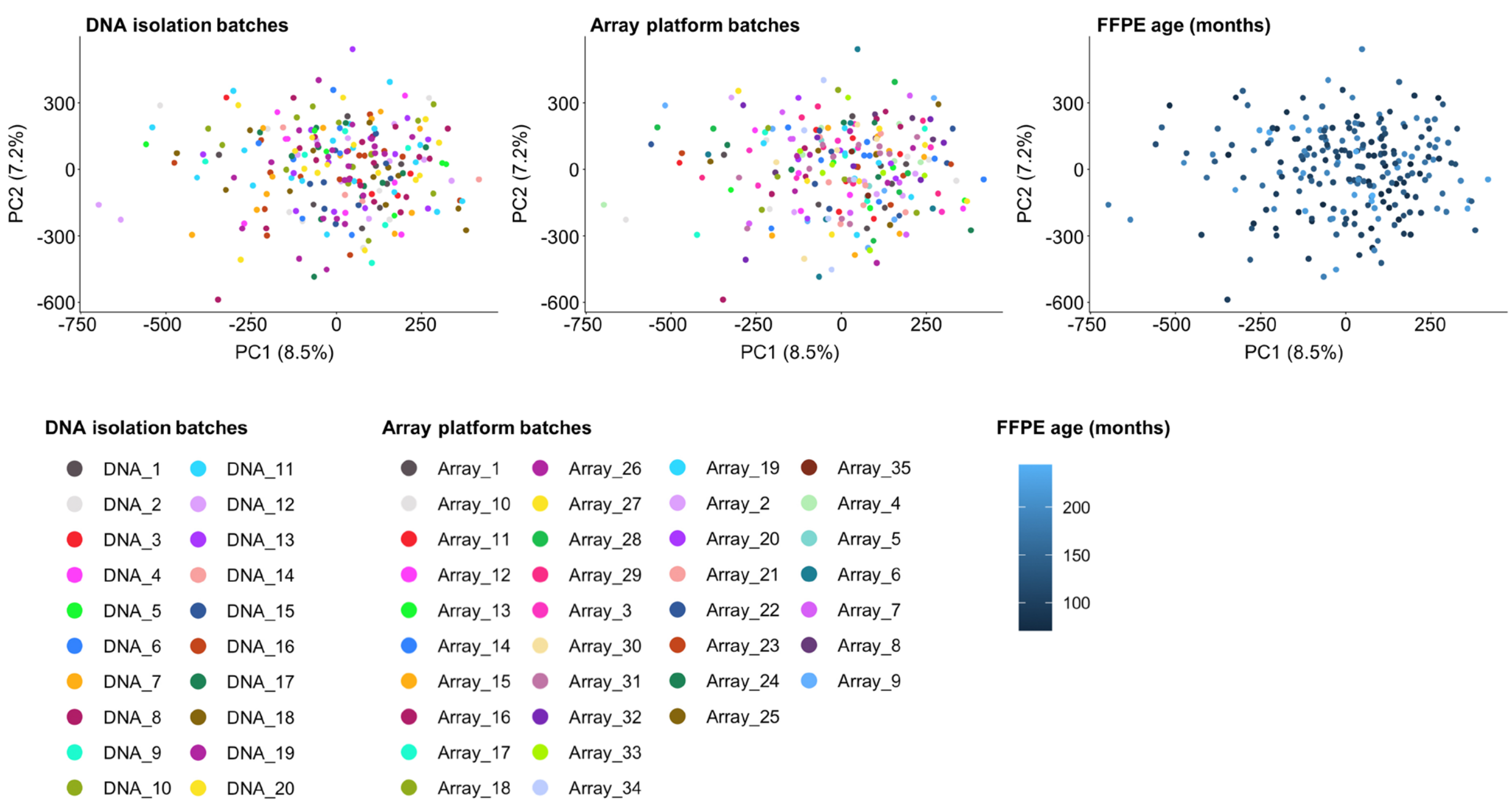

Supplementary Fig. 2: Batch effect correction in the Discovery cohort. a-c Results from permutational multivariate analysis of variance on all data from the Discovery cohort (n=231 tumour samples) (**a**). Scree plot illustrating results from principal component analysis on all data before correction (**b**). Scatter plots associating principal component 1 and 2 in terms of individual tumour sample position, colour-coding by DNA isolation batches, array platform batches, and age of the FFPE material (**c**). **d-f** Same data illustrations

as in **a-c**, but after correction using the limma::removeBatchEffect function. Source data are provided as a Source Data file.

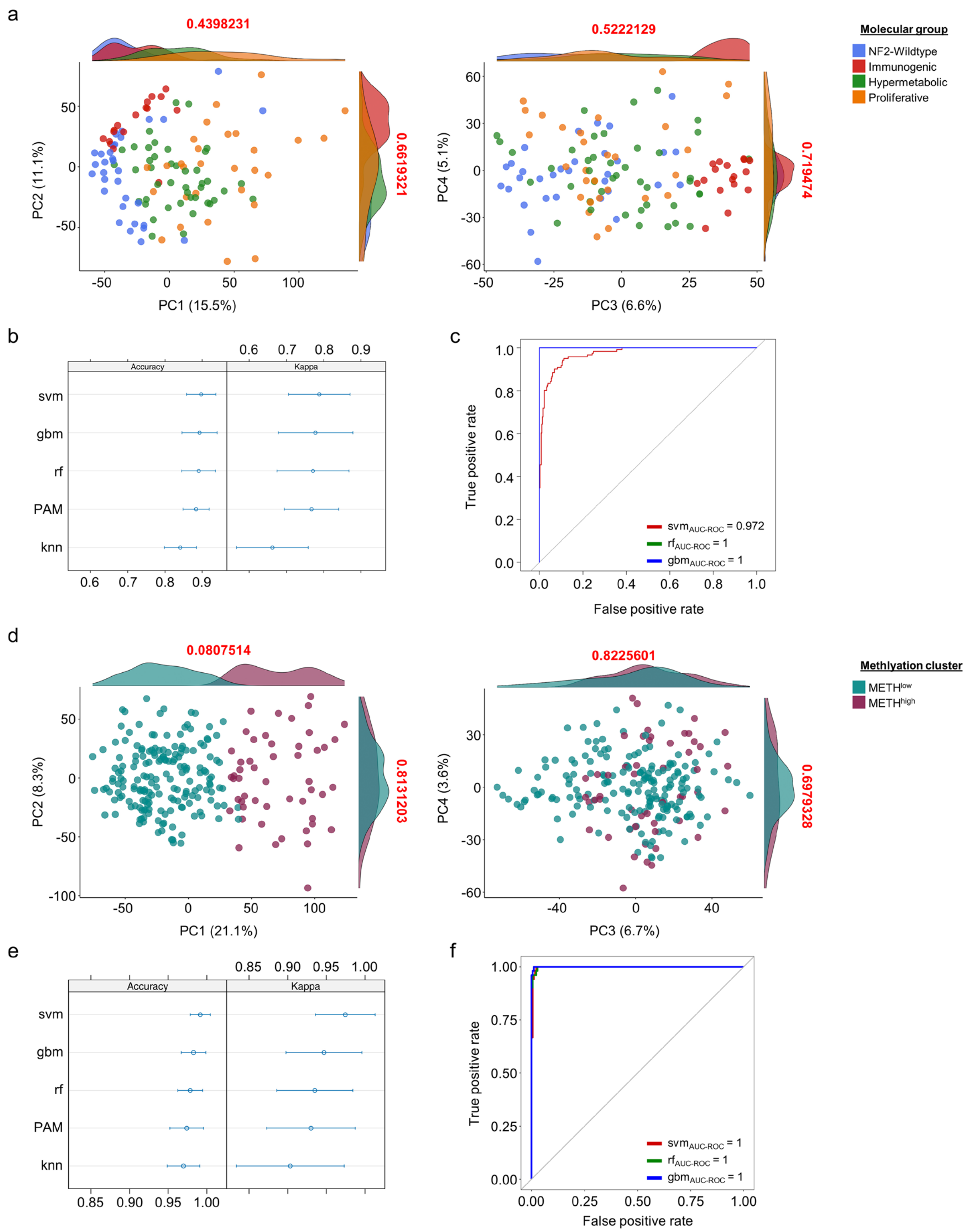

Supplementary Fig. 3: Data structure and classification performance metrics for classification models. a Data structure in the meningioma cohort from Nassiri et al. 2021 (n=121 meningioma samples)[2] with respect to molecular groups in form of a PCA plot. **b** Performance of models to predict molecular groups as judged by accuracy and Cohen's kappa. Employed algorithms were: support vector machine (svm), stochastic gradient boosting (gbm), random forest (rf), nearest shrunken centroids (PAM), and k-nearest neighbors (knn). **c** Micro-averaged ROC curve for multiclass classification performance regarding molecular groups for selected models. **d** PCA plots for the Discovery cohort separating $METH^{low}$ (n=51) and $METH^{high}$ (n=180) meningiomas. **e** Accuracy and Cohen's kappa for several models trained on the contrast of $METH^{low}$ and $METH^{high}$ meningiomas. **f** ROC curve for binary classification for selected models. Source data are provided as a Source Data file.

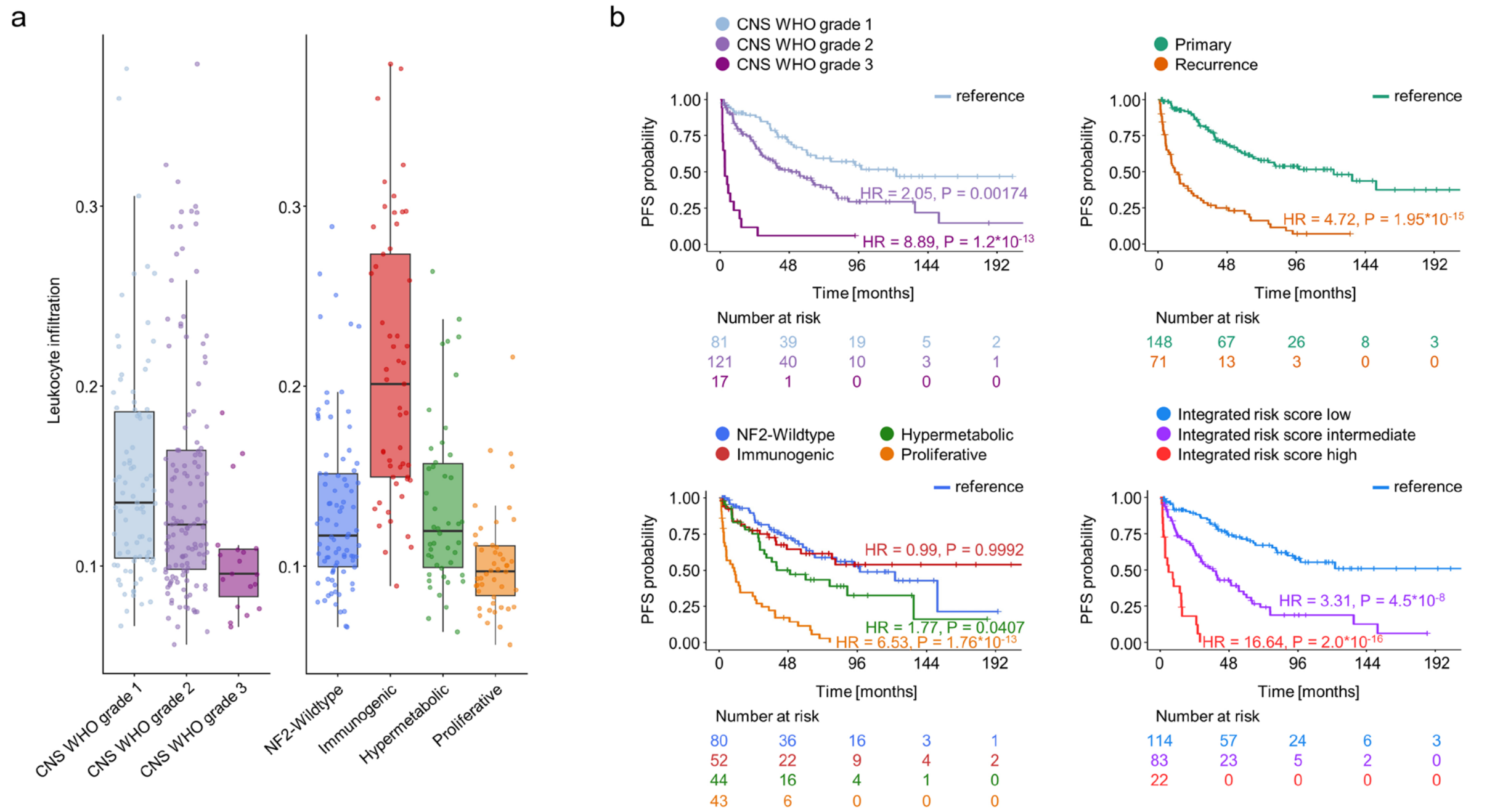

Supplementary Fig. 4: Benchmarking of the Discovery cohort. a Estimation of meningioma immune cell infiltration from methylation arrays. Immune infiltration decreases with disease aggressiveness, as judged by CNS WHO grading (grade 1: n=81; grade 2: n=126; grade 3: n=24) and DNA methylation family assignment (NF2-Wildtype: n=86; Immunogenic: n=53; Hypermetabolic: n=46; Proliferative: n=46), and the previously described Immunogenic methylation group of meningiomas shows the highest rate of infiltrations. **b** Kaplan-Meier analyses of progression-free survival for the Discovery cohort, stratified by either CNS WHO grading (grade 1: n=81; grade 2: n=121; grade 3: n=17), disease setting (Primary: n=148; Recurrence: n=71), molecular group (NF2-Wildtype: n=80; Immunogenic: n=52; Hypermetabolic: n=44; Proliferative: n=43), or Integrated risk score (low: n=114; intermediate: n=83; high: n=22). Hazard ratios and *P* values were generated using Cox proportional hazard regression with two-sided Wald tests. For all boxplots, boxes represent the interquartile range (IQR) with the median indicated by the centre line and whiskers extending to extreme outliers within 1.5x IQR. Source data are provided as a Source Data file.

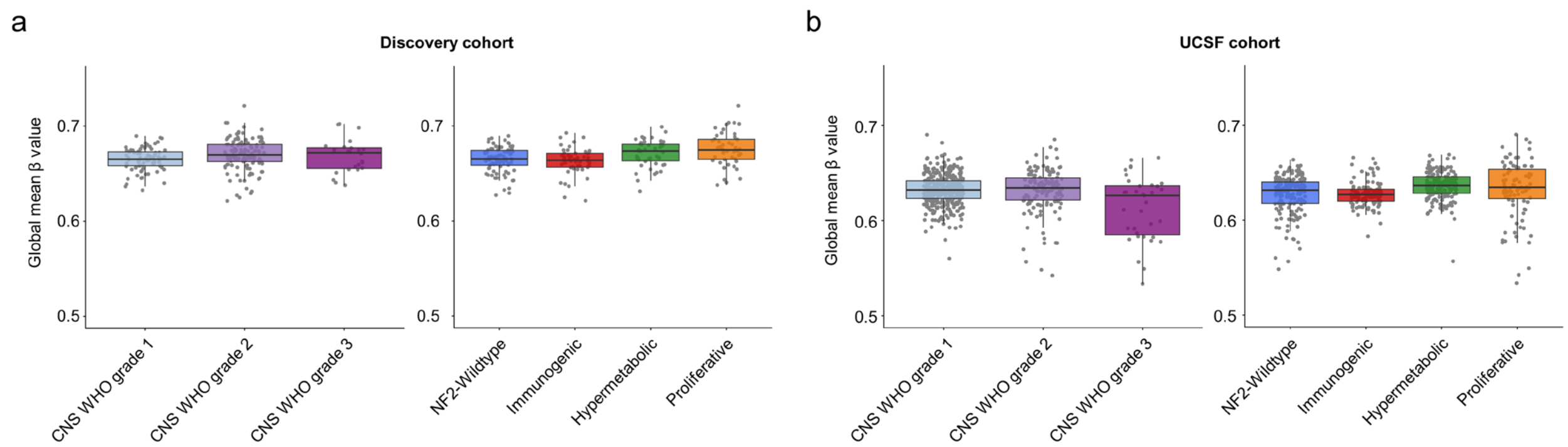

Supplementary Fig. 5: Association of tumor classifications and global DNA methylation in distinct meningioma cohorts. a-b Global mean methylation values and their association with CNS WHO grading and methylation groups in the Discovery (**a**) and the UCSF cohort[3] (**b**). Discovery cohort: grade 1 (n=81), grade 2 (n=126), grade 3 (n=24), NF2-Wildtype: (n=86), Immunogenic (n=53), Hypermetabolic (n=46), Proliferative (n=46). UCSF cohort: grade 1 (n=388), grade 2 (n=142), grade 3 (n=35), NF2-Wildtype: (n=187), Immunogenic (n=111), Hypermetabolic (n=172), Proliferative (n=95). For all boxplots, boxes represent the IQR with the median indicated by the centre line and whiskers extending to extreme outliers within 1.5x IQR. Source data are provided as a Source Data file.

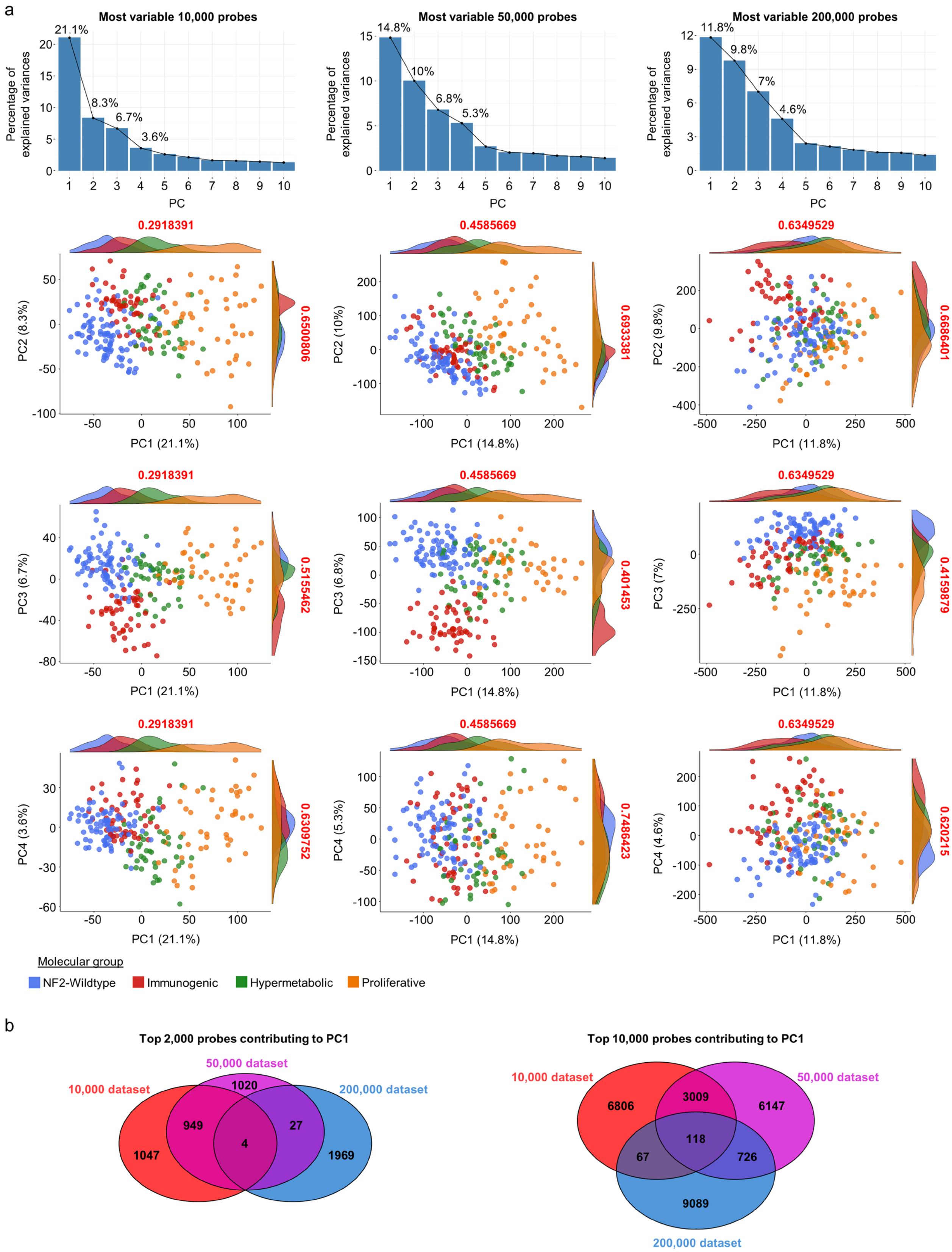

Supplementary Fig. 6: Principal component analysis identifies recurrence-associated methylation probes based on molecular group classification. a Scree plots for the first 10 principal components

(PCs) based on varying subsets of most variable methylation probes within the Discovery cohort (top). The first three to four components explain a major part of variance in the dataset. Plots illustrating clustering of meningiomas according to the first four components with molecular groups indicated (bottom). Density margins illustrate the distribution of corresponding subclassifications along individual components. Weighted pairwise mean overlaps indicated in red were calculated as a measure of separation. Best separation of low grade/good prognosis cases (NF2-Wildtype/Immunogenic groups) and higher grade/intermediate/bad prognosis cases (Hypermetabolic/Proliferative groups) is seen on PC1. PC3 separates immunogenic meningiomas from all other cases. **b** Venn diagrams illustrate the overlap of probes contributing most to PC1 (top 2,000 and 10,000) in distinct subsets of most variable probe datasets. Source data are provided as a Source Data file.

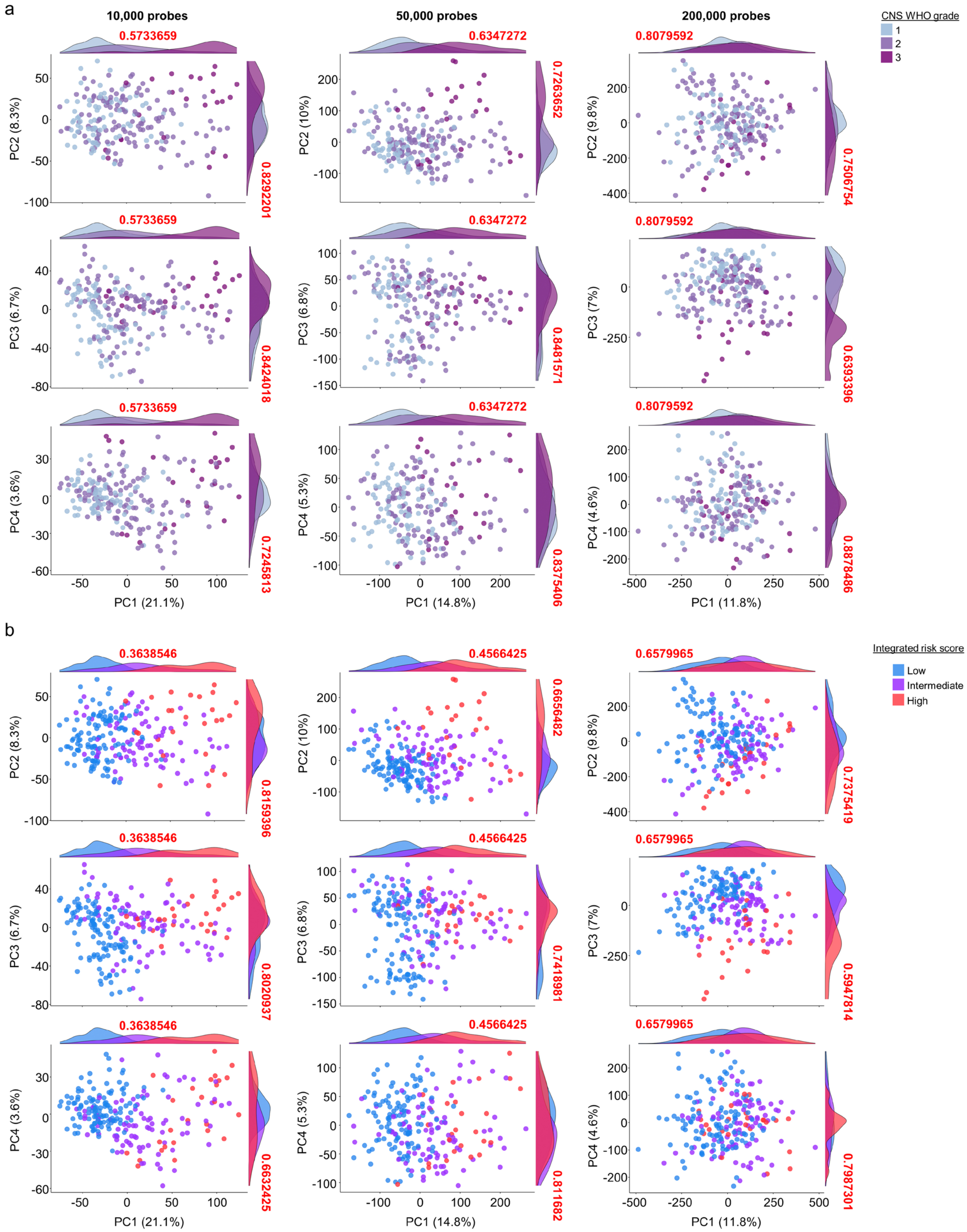

Supplementary Fig. 7: Principal component analysis using additional classification schemes. a-b Plots illustrating clustering of meningiomas according to the first four components with CNS WHO grade (**a**) or integrated risk score (**b**) indicated. Density margins illustrate the distribution of corresponding

subclassifications along individual components. Weighted pairwise mean overlaps indicated in red were calculated as a measure of separation. Source data are provided as a Source Data file.

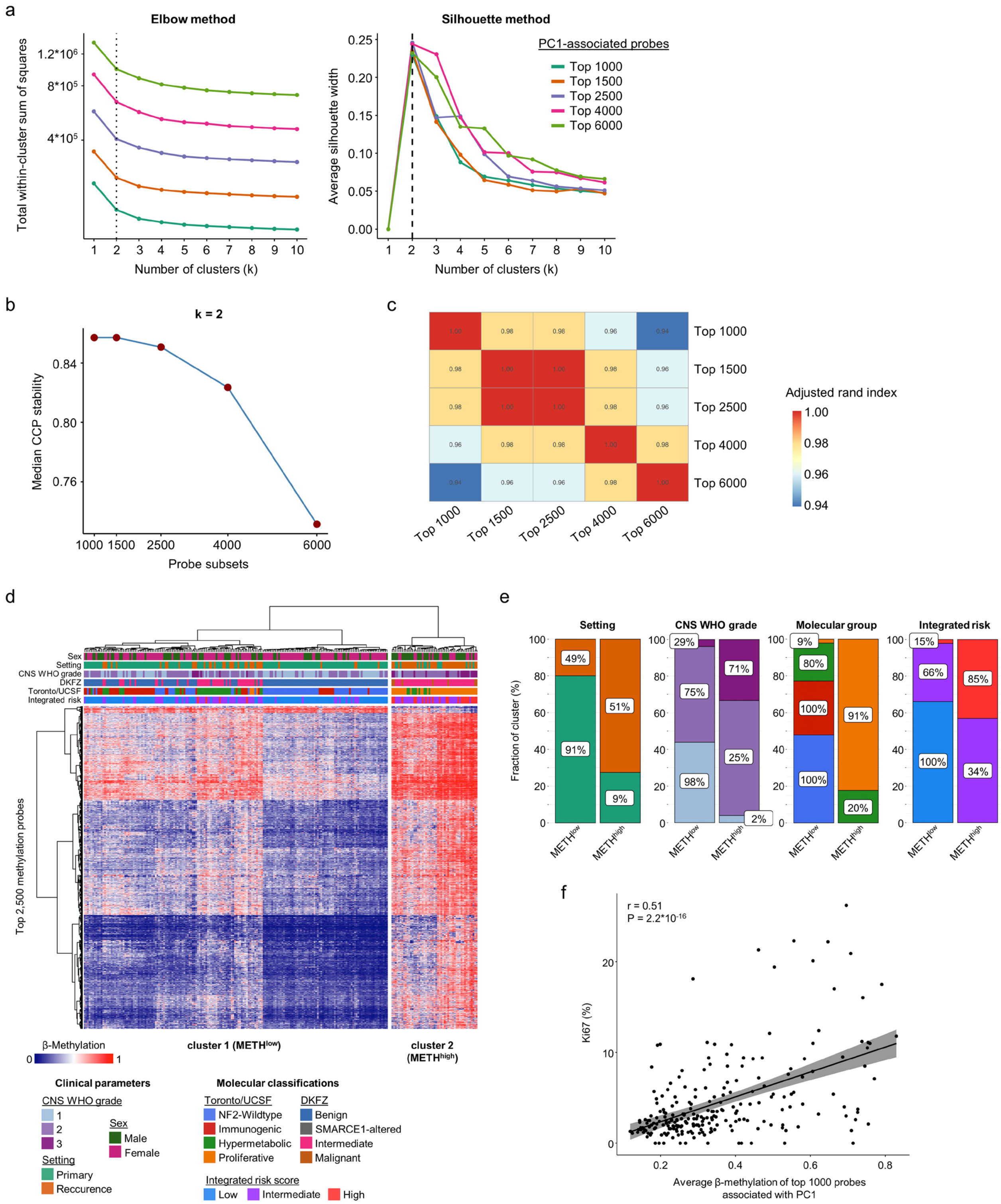

Supplementary Fig. 8: Cluster consistency for hypermethylation signature separating benign from aggressive meningiomas. a Elbow and Silhouette methods to determine optimal number of clusters for top PC1-asssociated methylation probes from principal component analysis using n=231 meningioma samples from the Discover cohort. Analyses were iteratively performed for several subsets of top associated probes. **b** Consensus clustering prediction (CCP) scores were defined as the mean consensus value between a given sample and all other samples assigned to the same cluster. Consensus clustering only

assumed the presence of two clusters. The median CCP scores across samples were used as a summary measure of cluster stability for each probe subset. **c** The adjusted rand index was used to quantify the concordance of cluster assignments for different probe subsets according to consensus clustering. **d** Unsupervised hierarchical clustering of meningioma samples (n=231) using the top 2500 probes contributing to PC1. Note that cluster assignment for all samples was identical to the clustering using the top 1000 probes seen in Fig. 1. **e** Relative assignments of meningioma samples to $METH^{low}$ (n=51) and $METH^{high}$ (n=180) clusters depending on classification by clinical setting, CNS WHO grade, and molecular group, and integrated risk score. Percentages on the y-axis show the fraction within each methylation cluster associated with a given condition, while percentages in boxes represent the partition of each condition to each methylation cluster. **f** Correlation of methylation cluster signature and fraction of Ki67-positive cells in tumor tissue from n=231 human meningiomas. Correlation coefficient r and a regression line with 95% CI are shown. Source data are provided as a Source Data file.

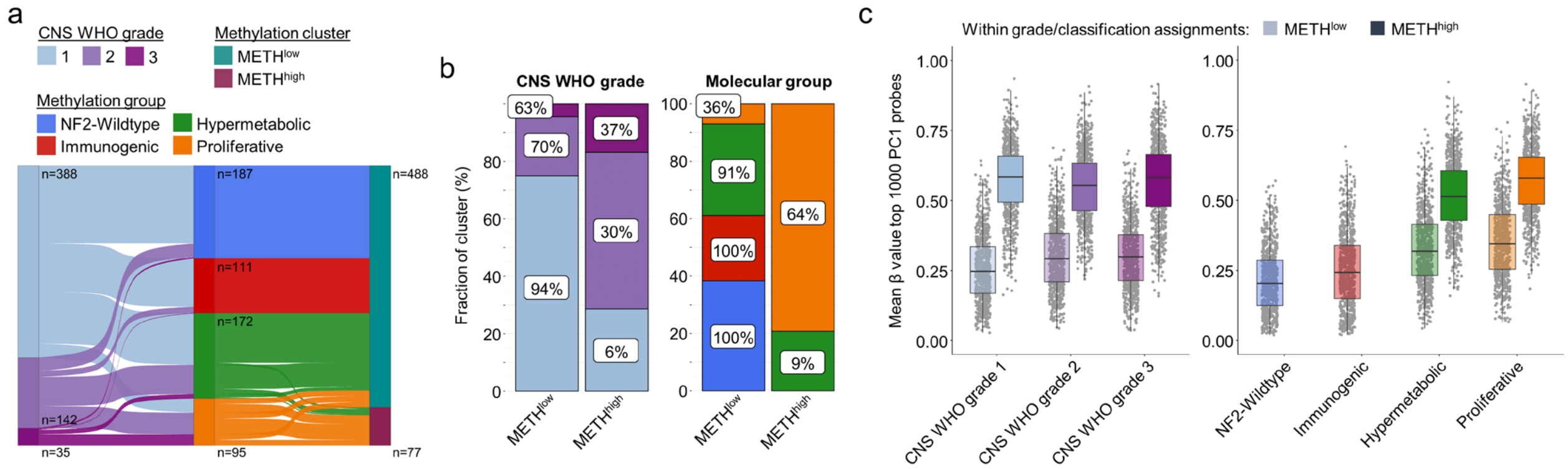

Supplementary Fig. 9: Cross-validation of METH^{low}/METH^{high} signatures in the UCSF meningioma cohort. **a** Sankey plot showing the relationship between CNS WHO grade, molecular groups, and METH^{low}/METH^{high} clusters in the UCSF cohort[3]. The number of tumours in each group is indicated within the figure panel. **b** Relative assignments of meningioma samples to METH^{low} (n=488) and METH^{high} (n=77) clusters according to classification by CNS WHO grading and molecular groups. Percentages on the y-axis show the fraction within each methylation cluster associated with a given condition, while percentages in boxes represent the partition of each condition to each methylation cluster. **c** Mean β values of the top 1000 PC1-associated probes across meningioma samples in relation to CNS WHO grading and molecular groups within the UCSF cohort, stratified by methylation cluster assignment. CNS WHO grade 1 (METH^{low}: n=366; METH^{high}: n=22), CNS WHO grade 2 (METH^{low}: n=100; METH^{high}: n=42), CNS WHO grade 3 (METH^{low}: n=22; METH^{high}: n=13), NF2-Wildtype (METH^{low}: n=187; METH^{high}: n=0), Immunogenic (METH^{low}: n=111; METH^{high}: n=0), Hypermetabolic (METH^{low}: n=156; METH^{high}: n=16), Proliferative (METH^{low}: n=34; METH^{high}: n=61). For all boxplots, boxes represent the IQR with the median indicated by the centre line and whiskers extending to extreme outliers within 1.5x IQR. Source data are provided as a Source Data file.

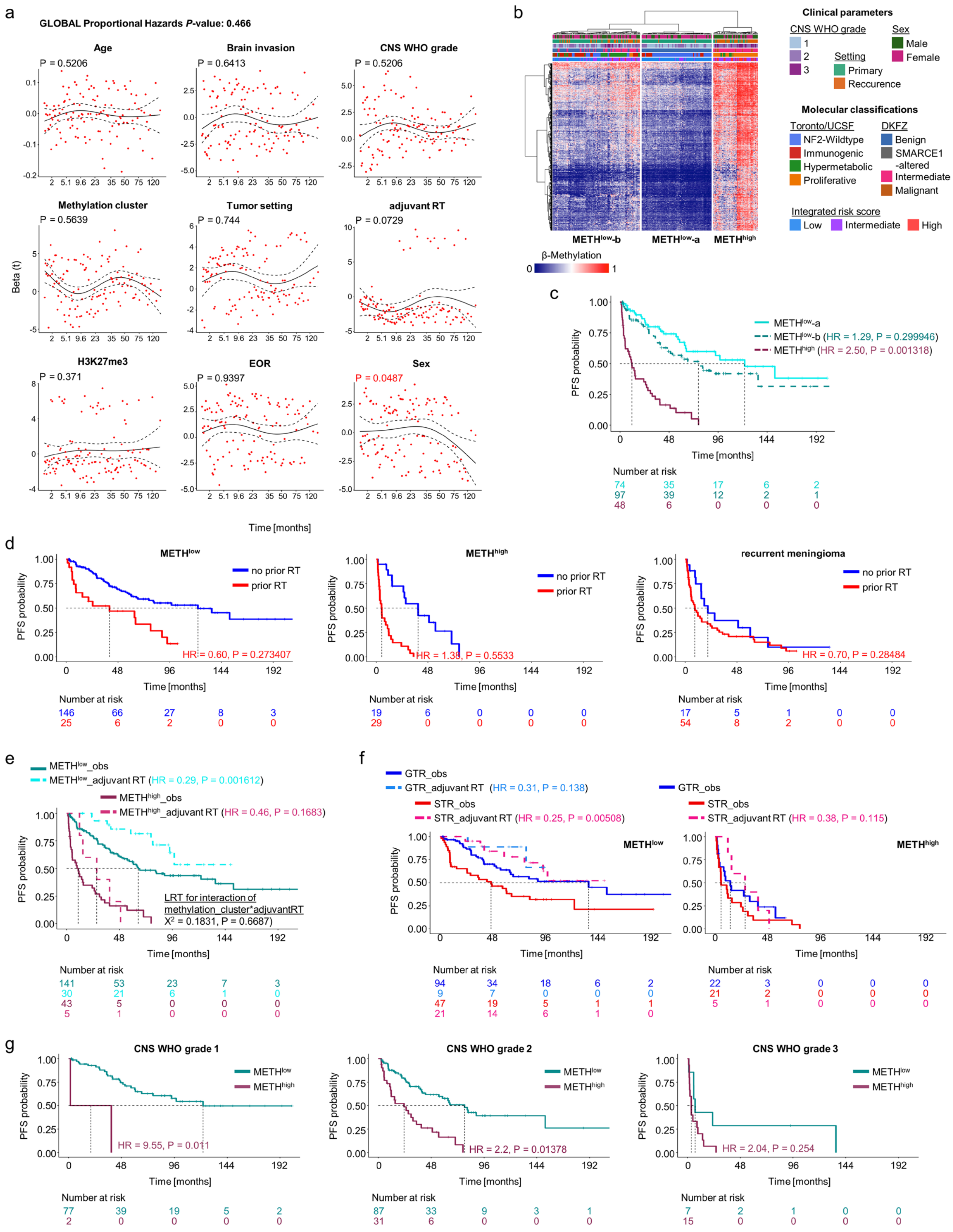

Supplementary Fig. 10: Survival analyses within the Discovery cohort. a Schoenfeld residual analyses to test the proportional hazards assumption for all coefficients used in modelling including age, sex, tumour setting (Primary vs. Recurrence), CNS WHO grading, EOR (extent of resection; GTR: gross total resection;

STR: subtotal resection), brain invasion, adjuvant RT, H3K27me3, and methylation cluster assignment for the Discovery cohort with n=231 human meningiomas. The proportional hazards assumption was assessed using the Grambsch and Therneau test based on scaled Schoenfeld residuals. The proportional hazard assumption is violated for sex, as evidenced by a significant relationship of residuals and time. **b** Clustering of the discovery cohort by the top 1000 PC1-asssociated probes into three clusters splits $METH^{low}$ meningiomas into low-level ($METH^{low}$-a; n=79) and mid-level ($METH^{low}$-b; n=101) groups. **c** Survival analysis shows a moderate, but not significant difference in PFS for $METH^{low}$-a and $METH^{low}$-b tumors ($METH^{low}$-a: n=74; $METH^{low}$-b: n=97; $METH^{high}$: n=48). **d** Survival analyses for $METH^{low}$ and $METH^{high}$ meningiomas, stratified by whether patients received radiotherapy before the follow-up start or not ($METH^{low}$: n=146 no prior RT and n=25 prior RT; $METH^{high}$: n=19 no prior RT and n=29 prior RT). Since only recurrent cases had received prior RT, this analysis was also performed in recurrent-only cases (n=17 no prior RT and n=54 prior RT). **e** PFS outcomes within the Discovery cohort stratified by methylation cluster and receipt of adjuvant RT ($METH^{low}$_obs: n=141; $METH^{low}$_adjuvant RT: n=30; $METH^{high}$_obs: n=43; $METH^{high}$_adjuvant RT: n=5). In this scenario, only $METH^{low}$ meningiomas seem to significantly benefit from adjuvant RT, however, a likelihood ratio test for interaction of methylation cluster and adjuvant RT is not significant. **f** PFS outcomes stratified by extent of resection and treatment modality (EOR and receipt of adjuvant RT) in $METH^{low}$ (GTR_obs: n=94; GTR_adjuvant RT: n=9; STR_obs: n=47; STR_adjuvant RT: n=21) and $METH^{high}$ (GTR_obs: n=22; STR_obs: n=21; STR_adjuvant RT: n=5) meningiomas. **g** PFS outcomes stratified by methylation cluster assignment within CNS WHO grade 1 (n=77 $METH^{low}$ and n=2 $METH^{high}$), CNS WHO grade 2 (n=87 $METH^{low}$ and n=31 $METH^{high}$), or CNS WHO grade 3 (n=7 $METH^{low}$ and n=15 $METH^{high}$). For all survival analyses, Cox proportional hazard models with two-sided Wald tests were generated while accounting for competing variables of meningioma behaviour. Source data are provided as a Source Data file.

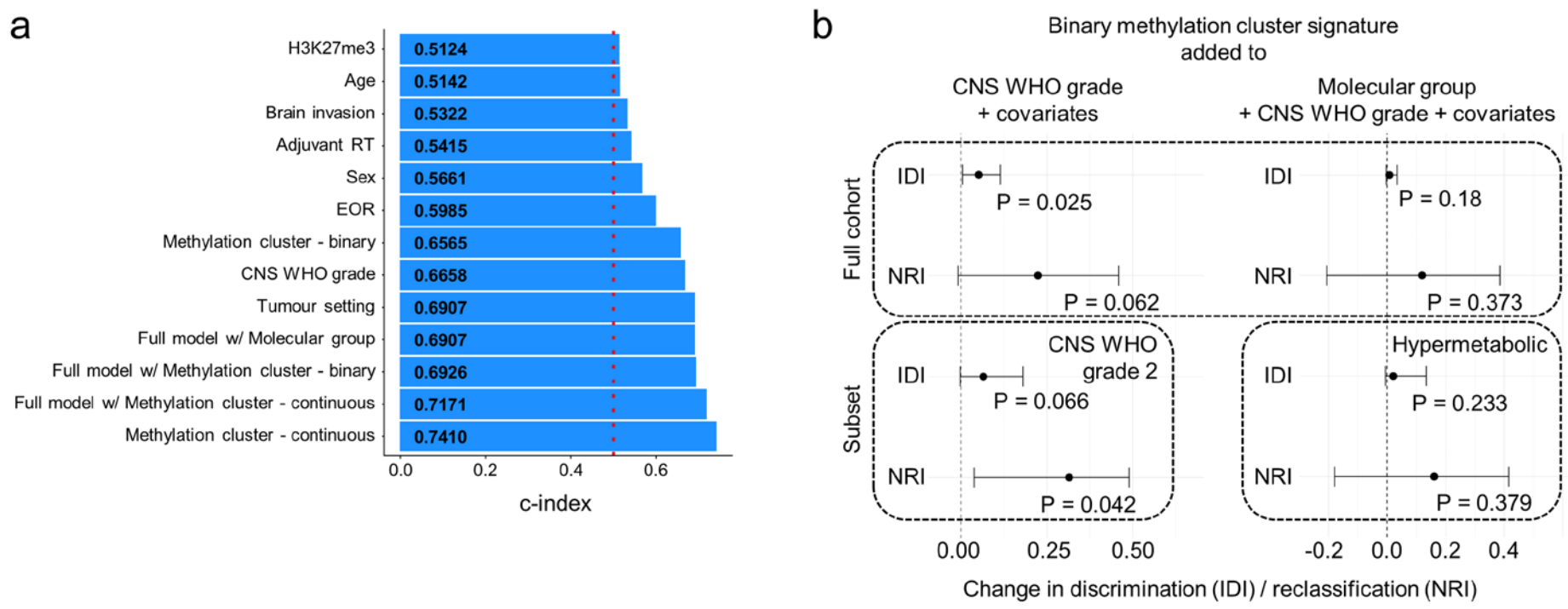

Supplementary Fig. 11: Performance metrics to compare survival models. a Bar graph illustrating concordance indices (c-index) for indicated univariate and multivariate models applied to PFS within the Discovery cohort (n=219). **b** Point estimates and 95% confidence intervals for integrated discrimination improvement (IDI) and net reclassification improvement (NRI) illustrating the change in discrimination and reclassification associated with inclusion of the binary methylation cluster signature. Analysis included n=219 (full cohort), n=118 patients (CNS WHO grade 2), or n=44 patients (Hypermetabolic). P values and confidence intervals were estimated using 2,000 perturbation iterations. Source data are provided as a Source Data file.

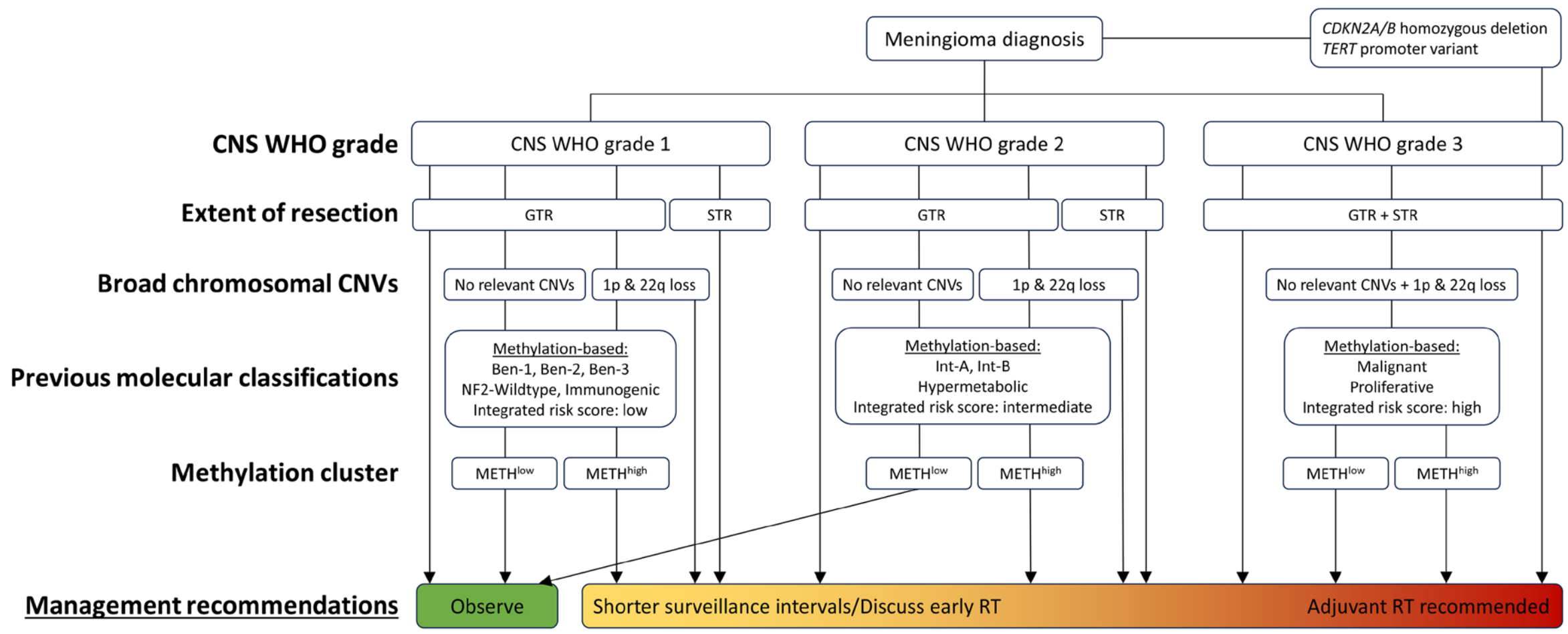

Supplementary Fig. 12: Decision tree for clinical follow-up management. Recommendations for clinical management of meningioma patients according to CNS WHO grade, EOR, and extended molecular diagnostics. $METH^{high}$ tumors otherwise classified as CNS WHO grade 1 should be subjected to shorter surveillance time frames, while $METH^{low}$ tumors with CNS WHO grade 2 and without relevant CNVs might be recommended for observation only.

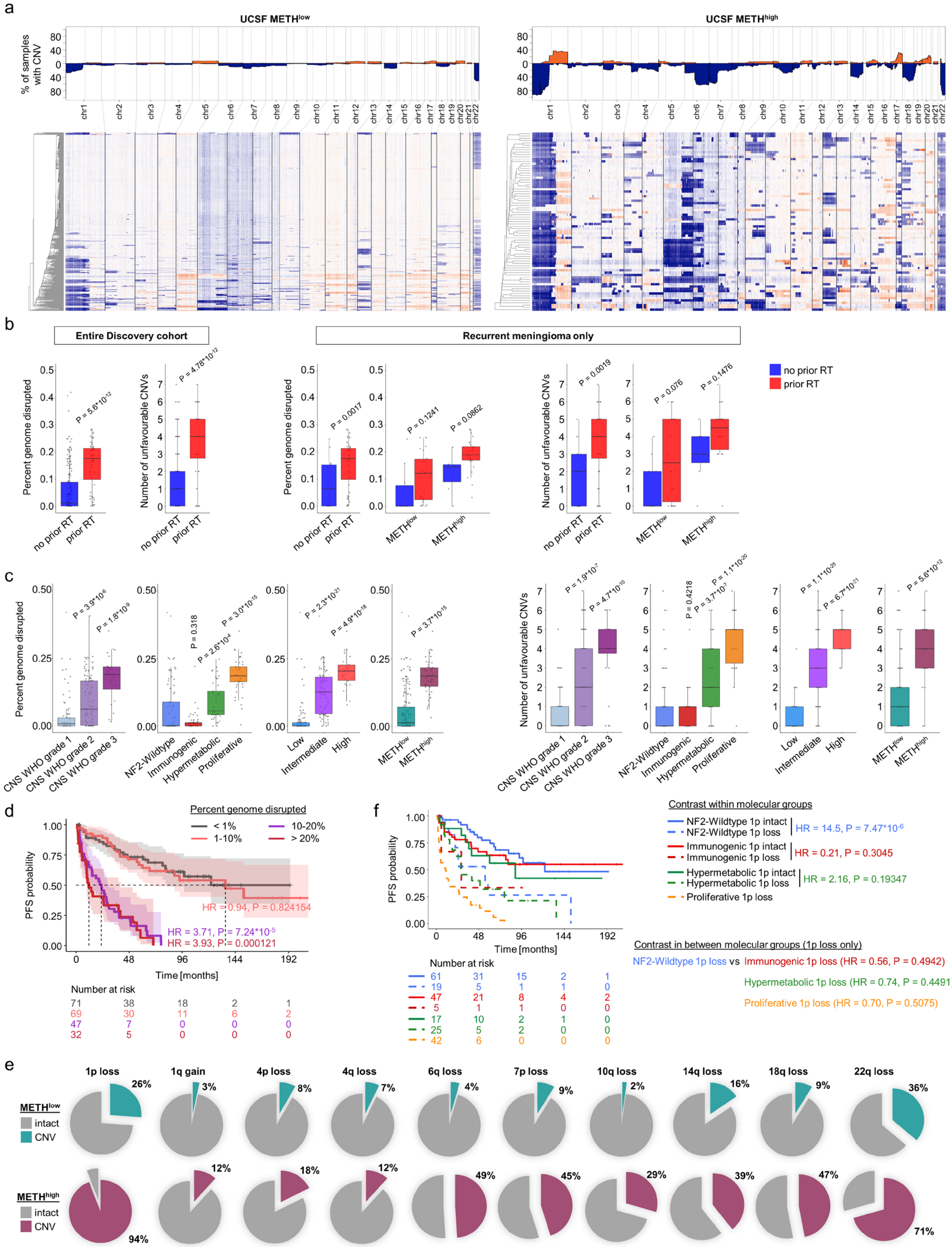

Supplementary Fig. 13: Validation of broad chromosomal CNVs in the Discovery and UCSF cohort. **a** Summary genome plot illustrating the percentage of samples exhibiting CNVs in predicted METH^{low}

(n=488) and $METH^{high}$ (n=77) meningiomas from the UCSF cohort[3] (above). Heatmap illustrates identified CNVs on a sample level (below). **b** Effect of radiotherapy prior to the follow-up time on CNVs within the entire Discovery cohort (no prior RT: n=175; prior RT: n=56; left panel) or only in recurrent meningiomas ($METH^{low}$: no prior RT n=10; prior RT n=26; $METH^{high}$: no prior RT n=7; prior RT n=30; right panel). Percentage of genome disrupted or number of unfavourable CNVs were used as metrics to define CNV burden. P values are derived from a Kruskal-Wallis test followed by two-sided Dunn's post hoc test with Benjamini-Hochberg adjustment for multiple testing (more than 2 groups) or Mann-Whitney test (2 groups). **c** Boxplots illustrating the relationship of genome instability (left) or number of unfavourable CNVs (right) with CNS WHO grading (CNS WHO grade 1: n=81; CNS WHO grade 2: n=126; CNS WHO grade 3: n=24), molecular group, (NF2-Wildtype: n=86; Immunogenic: n=53; Hypermetabolic: n=46; Proliferative: n=46), integrated risk score (low: n=118; intermediate: n=86; high: n=26), and methylation clustering ($METH^{low}$: n=180; $METH^{high}$: n=51) in the Discovery cohort. P values are derived from a Kruskal-Wallis test followed by two-sided Dunn's post hoc test with Benjamini-Hochberg adjustment for multiple testing (more than 2 groups) or Mann-Whitney test (2 groups). **d** Kaplan-Meier analyses for the Discovery cohort, stratifying samples by percentage of genome disrupted (<1%: n=71; 1-10%: n=69; 10-20%: n=47; >20%: n=32) . **e** Pie charts illustrating the penetrance of broad chromosomal CNVs associated with poor prognosis in $METH^{low}$ and $METH^{high}$ clusters. **f** Kaplan-Meier analyses for the Discovery cohort, stratifying samples both by molecular group and chromosome 1p status (NF2-Wildtype: n=61 1p intact and n=19 1p loss; Immunogenic: n=47 1p intact and n=5 1p loss; Hypermetabolic: n=17 1p intact and n=25 1p loss; Proliferative: n=42 1p loss). Hazard ratios from Cox proportional hazards modeling with two-sided Wald tests with adjustment for competing variables are shown for within molecular group comparisons, as well as for within 1p-deleted meningiomas, using NF2-Wildtype cases as a reference. For all boxplots, boxes represent the IQR with the median indicated by the centre line and whiskers extending to extreme outliers within 1.5x IQR. Source data are provided as a Source Data file.

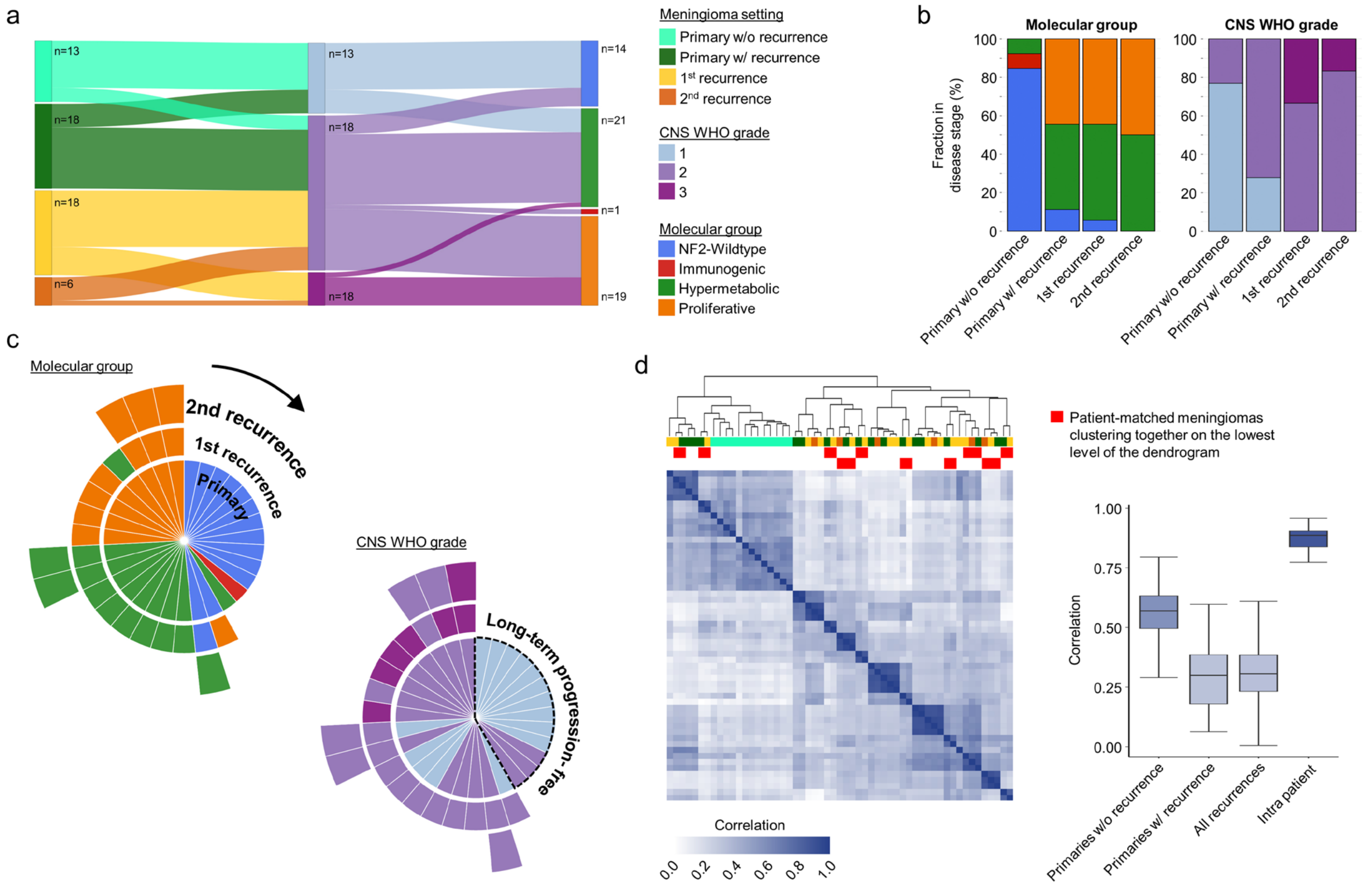

Supplementary Fig. 14: Detailed analyses of the longitudinal meningioma cohort. a Sankey plot showing the relationship of distinct meningioma settings, CNS WHO grading, and molecular group within the Longitudinal cohort. The number of tumours in each category can be seen within the panel. **b** Relative assignments of different meningioma settings to molecular group and CNS WHO grading classifications. **c** Sunburst charts illustrating the longitudinal development of molecular group and CNS WHO grade assignments. Each radial segment corresponds to one patient. Primary and recurrent samples are ordered from inside to outside. Long-term progression-free primary meningiomas (i.e. Primary w/o recurrence) are indicated. **d** Heatmap showing the pairwise correlation coefficients across distinct meningioma settings (Primary w/o recurrence: n=13; Primary w/ recurrence: n=18; 1st recurrence: n=18; 2nd recurrence: n=6). Each genome was binned into 300k bp segments, and mean log2 ratio of signal intensities of all segments were used to calculate correlation. A box plot illustrates correlation coefficients within indicated categories. Note that the majority of tumour samples clustered by patient on the lowest level of the dendrogram, and intra-patient correlations are substantially higher as compared to correlations within meningioma settings. For all boxplots, boxes represent the IQR with the median indicated by the centre line and whiskers extending to extreme outliers within 1.5x IQR. Source data are provided as a Source Data file.

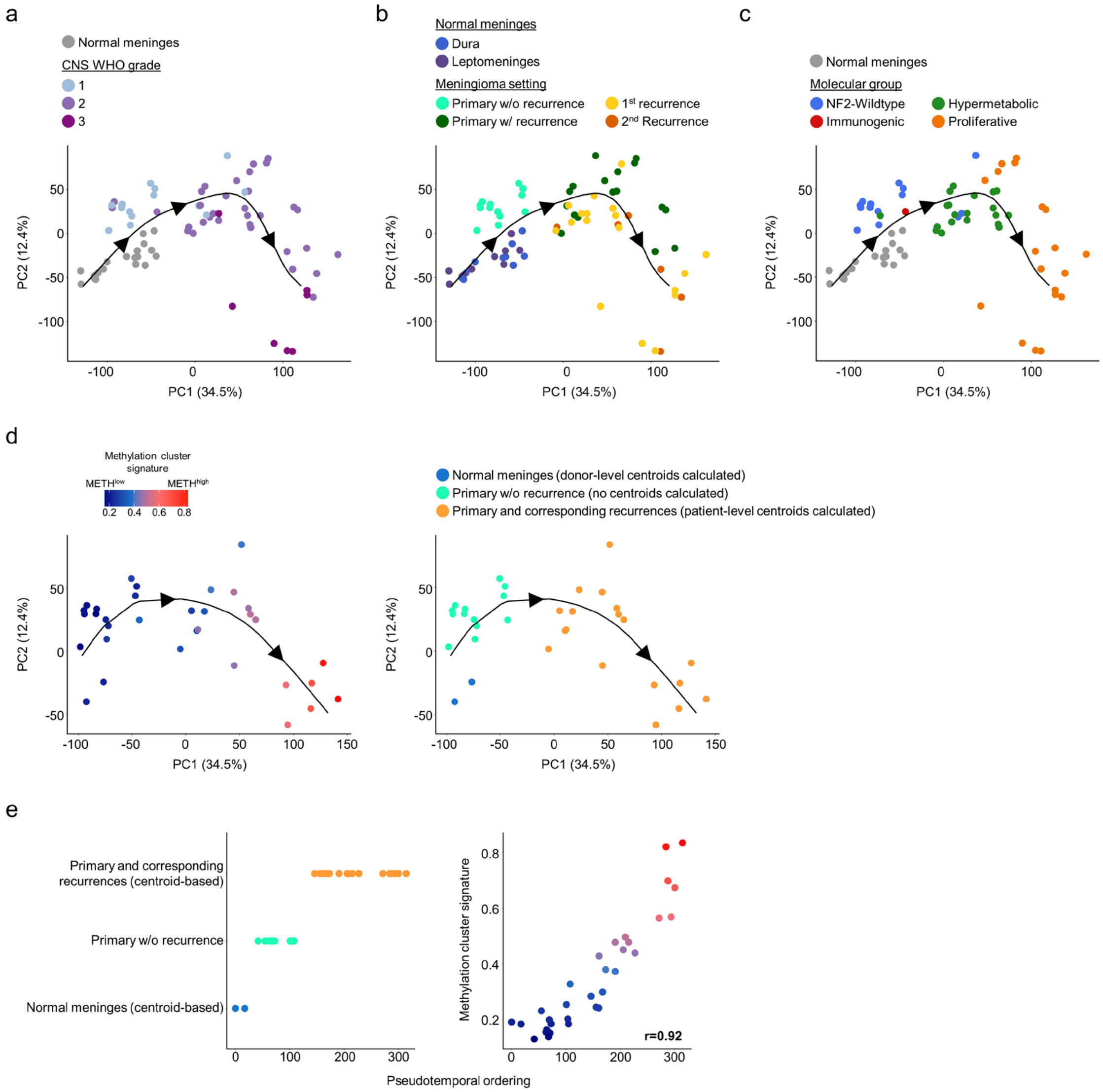

Supplementary Fig. 15: Trajectory analysis for normal meninges and meningiomas based on DNA methylation profiles. a-c Trajectory analysis for normal meninges, benign, and progressive meningiomas based on DNA methylation profiles using slingshot (compare to Fig. 4f). Colour coding indicates CNS WHO grading for meningioma samples (**a**), normal meninges and distinct meningioma settings (**b**), or molecular group assignment for meningioma samples (**c**). **d** Trajectory analysis was repeated based on donor-level (normal meninges) and patient-level centroids (recurrent meningioma) to account for non-independence of samples from the same donor/patient. **e** Pseudotime analysis for centroid-based trajectory inference. Source data are provided as a Source Data file.

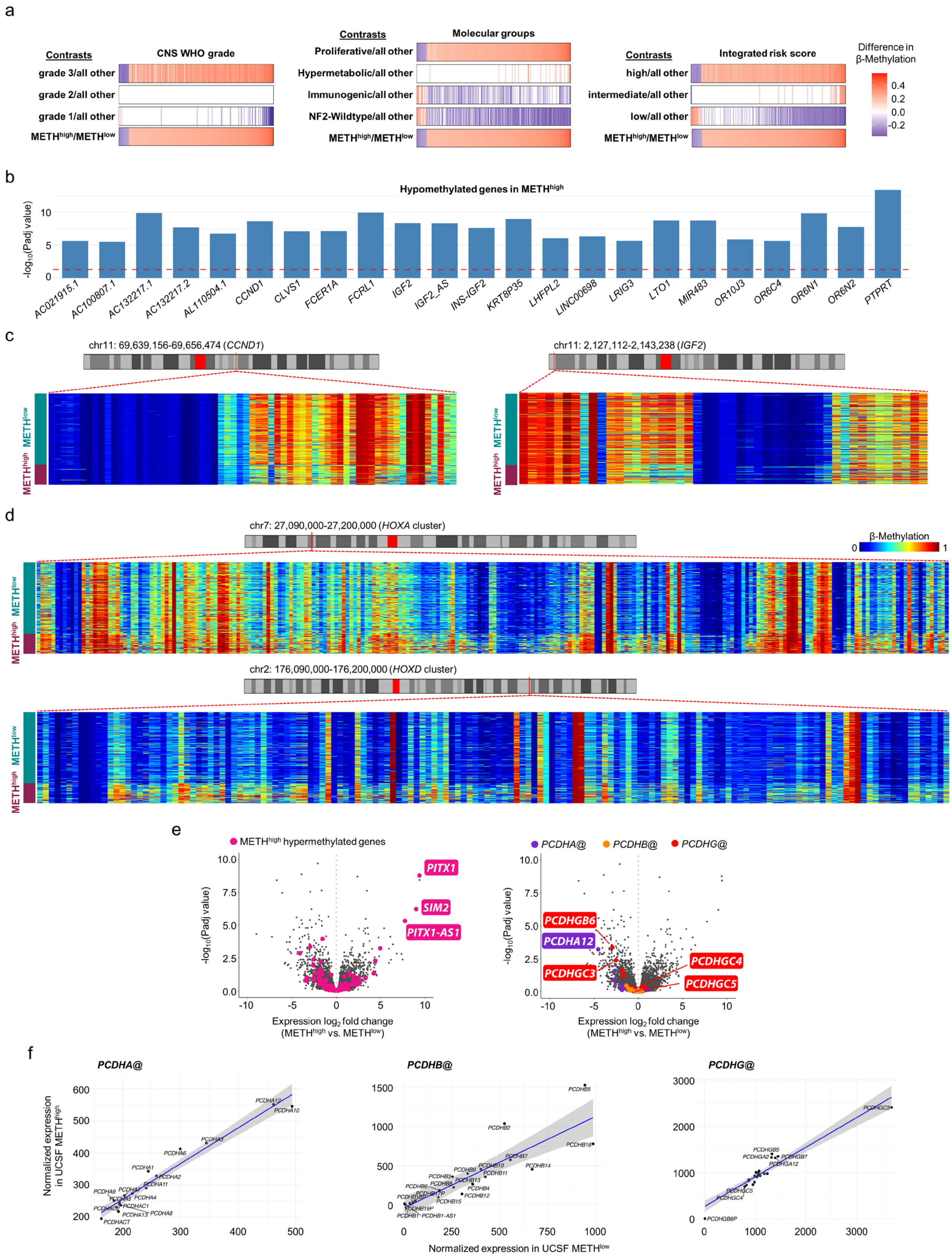

Supplementary Fig. 16: Genes predicted to be affected by differential DNA methylation in METH^{high} meningiomas and their validation in gene expression data. a Illustration of shared DNA methylation

changes between $METH^{high}$/$METH^{low}$ clusters and individual classification schemes of meningioma. Bottom bar presents all methylation probes significantly different in $METH^{high}$ vs $METH^{low}$ tumors. Top bars illustrate the methylation change of those probes within probes determined to significantly define individual classifications (e.g. NF2-Wildtype) when compared to all other meningiomas. **b** Bar plot showing all significantly enriched genes targeted by hypomethylated probes in $METH^{high}$ meningiomas. **c** Track view of two example genes (CCND1 and IGF2) determined to be hypomethylated in $METH^{high}$ meningiomas. **d** Track view of methylation probes targeting the genomic loci of gene clusters HOXA and HOXD within the Discovery cohort. Note hypermethylation of both gene clusters in $METH^{high}$ meningiomas. **e** Volcano plots illustrating the log2 fold changes in gene expression between $METH^{high}$ versus $METH^{low}$ tumour samples (n=5 each). Coloured genes have been found to be hypermethylated in $METH^{high}$ samples from the Discovery cohort (left), or denote clustered protocadherins (right). Statistics are derived from two-sided Wald tests with Benjamini-Hochberg adjustments for multiple testing. **f** Correlation of normalized gene expression values for individual protocadherin clusters in $METH^{low}$ and $METH^{high}$ meningiomas from the UCSF cohort. A regression line and the corresponding 95% confidence interval are shown. Source data are provided as a Source Data file.

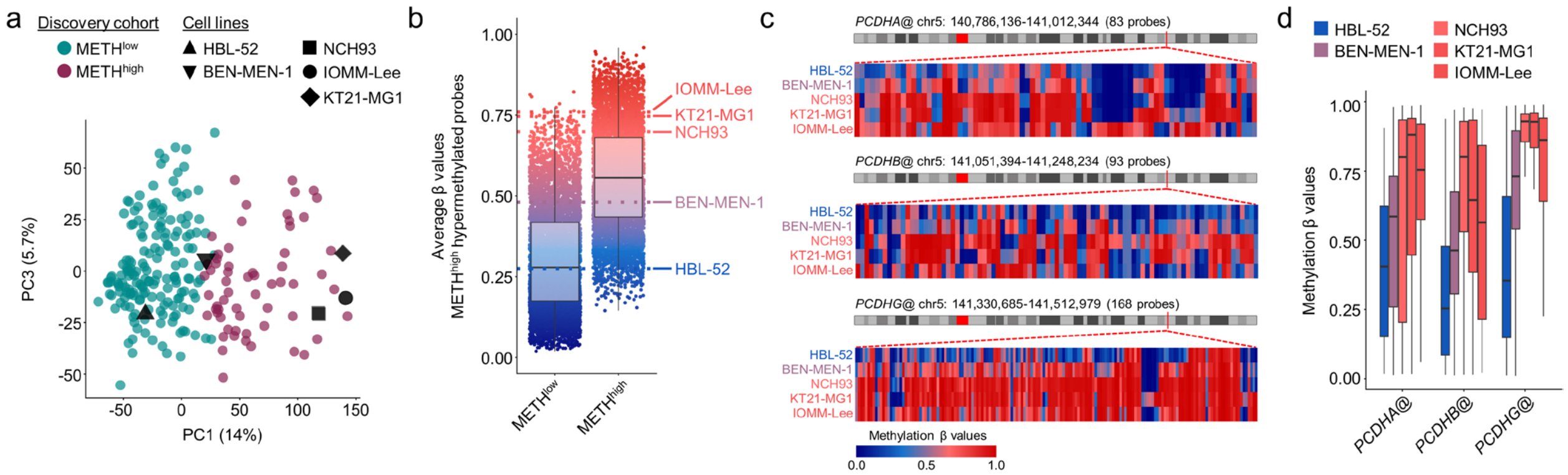

Supplementary Fig. 17: Validation of the DNA hypermethylation signature in in vitro models of meningioma. a PCA plot of meningiomas from the Discovery cohort (METH^{low}: n=180; METH^{high}: n=51) based on the 50,000 most variable methylation probes, with colour coding indicating methylation cluster assignment. Meningioma cell lines (n=5) were treated as supplementary variables. **b** Box plots showing the per probe average methylation degree for probes hypermethylated in METH^{high} meningiomas across all tumors in the Discovery cohort (METH^{low}: n=180; METH^{high}: n=51). Per cell line average β values are indicated. **c** Track view of methylation probes targeting the genomic loci encompassing *PCDHA@*, *PCDHB@*, and *PCDHG@* gene clusters in meningioma cell lines (n=5 cell lines). **d** Box plots illustrating the distribution of methylation β values for probes targeting protocadherin gene clusters in meningioma cell lines (n=5 cell lines). For all boxplots, boxes represent the IQR with the median indicated by the centre line and whiskers extending to extreme outliers within 1.5x IQR. Source data are provided as a Source Data file.

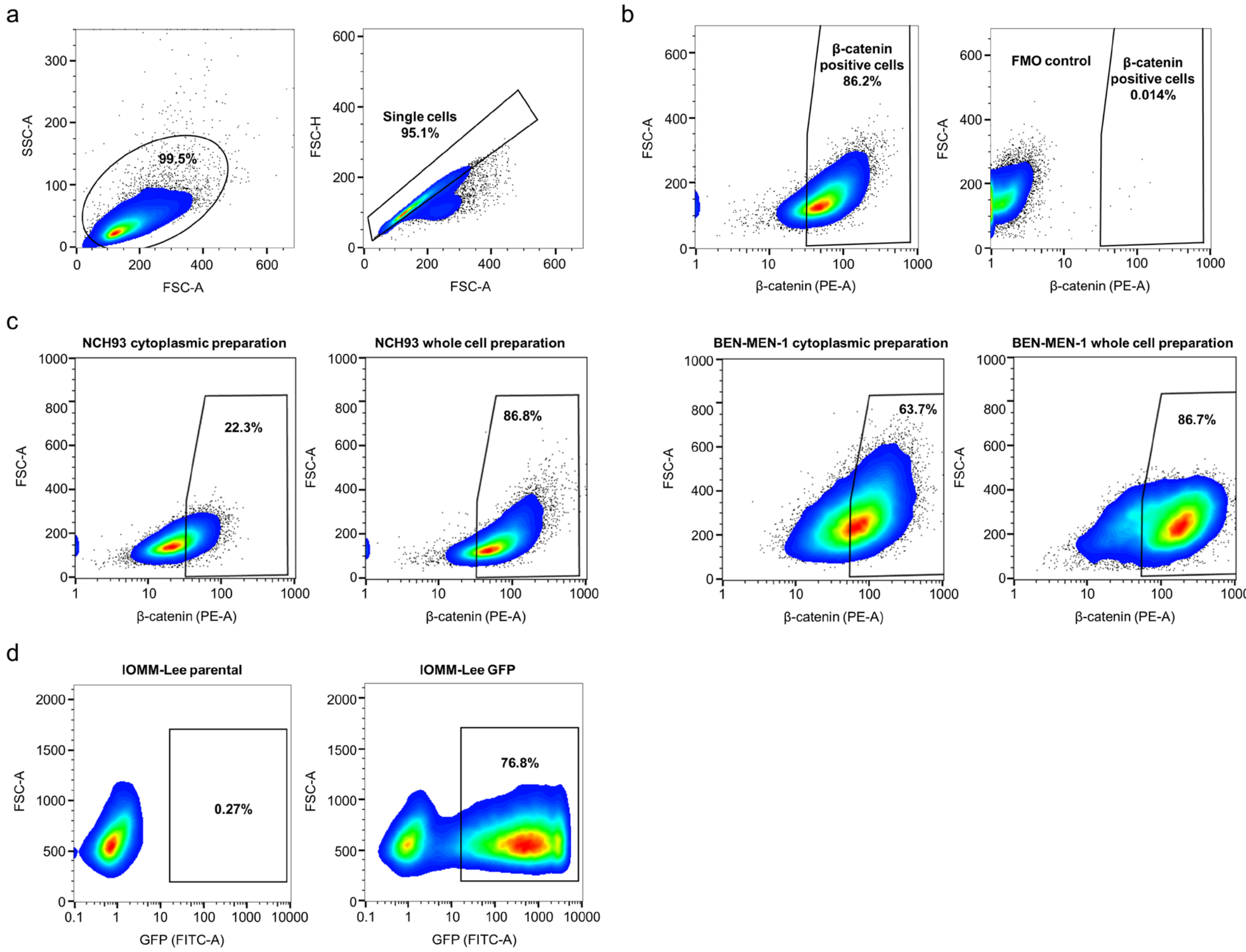

Supplementary Fig. 18: Gating strategy for fluorescence associated cell sorting. a General gating strategy to use forward and sideward scatter to identify a general cell population (left) and single cells (right), shown for BEN-MEN-1 cells. This strategy has been applied to all FACS analyses in our study. **b** Gating strategy determining the β-catenin-positive BEN-MEN-1 cells using a PE-conjugated anti-β-catenin antibody (left). The same cells, but no antibody included (right). This gating strategy refers to experiments shown in Fig. 7b and 7e. **c** Effect of differential cellular extract preparations (cytoplasmic or whole cell) on the fraction of β-catenin-positive cells detected, shown for NCH93 cells (left) and BEN-MEN-1 cells (right). This gating strategy refers to experiments shown in Fig. 7b and 7e. **d** Gating strategy for GFP-positive cells. Examples show gating from IOMM-Lee parental cells, applied to GFP-expressing IOMM-Lee cells. This gating strategy refers to experiments shown in Fig. 7e.

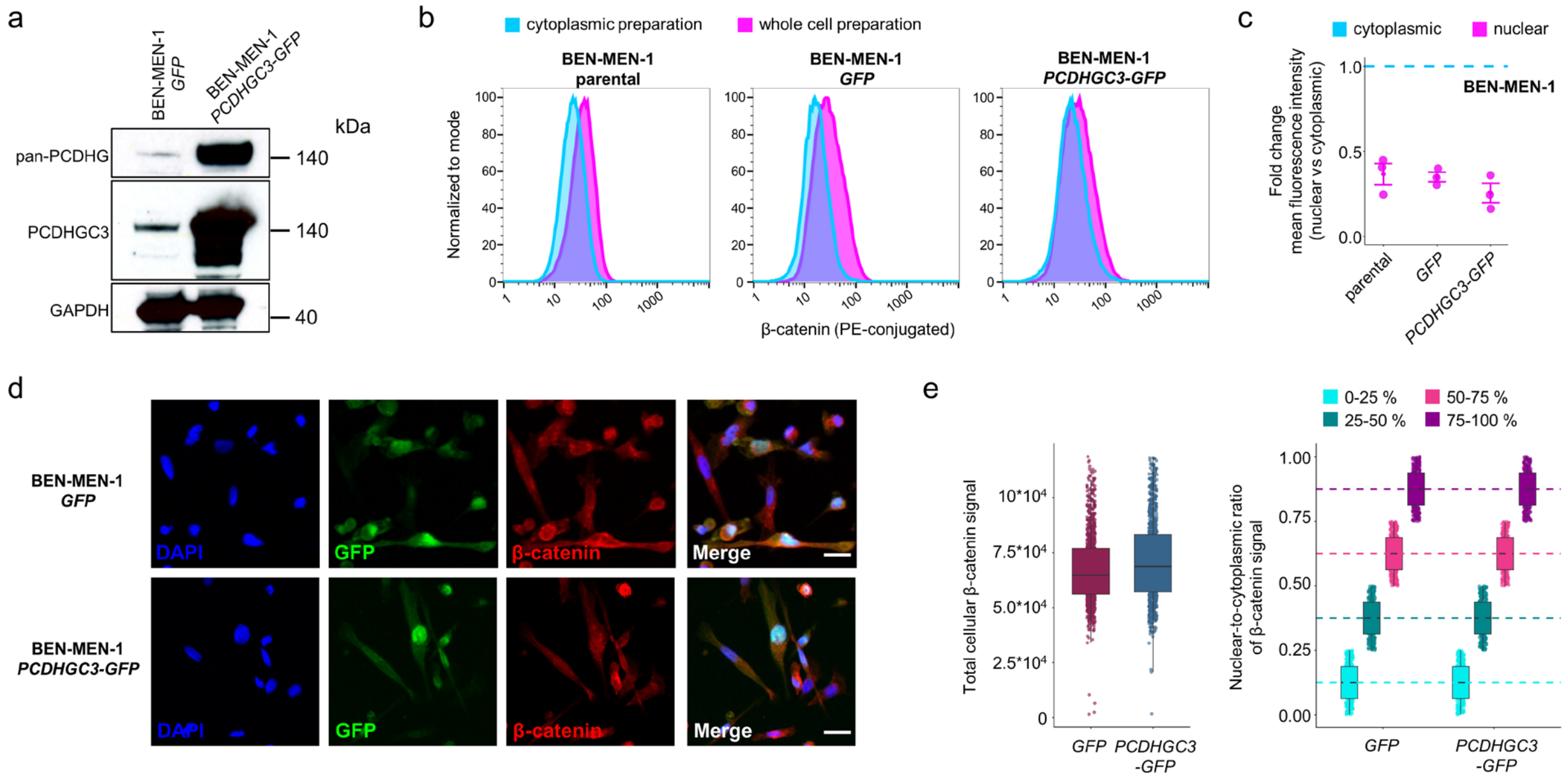

Supplementary Fig. 19: Effect of PCDHGC3 overexpression on β-catenin localization in BEN-MEN-1 cells. a Western blot analysis validating overexpression of PCDHGC3 in BEN-MEN-1 meningioma cells. Blots illustrate representative results from n=3 independent iterations. **b** Normalized density distributions of β-catenin signal from cytoplasmic or whole cell preparations of parental, control, and PCDHGC3-GFP-expressing BEN-MEN-1 cells. Data shown are representative results from n=3 experimental replicates. **c** Quantification of cytoplasmic and nuclear β-catenin signal in parental, control, and PCDHGC3-GFP-expressing BEN-MEN-1 cells. Data is shown as fold change in mean fluorescence intensity in the nuclear vs cytoplasmic compartment (n=3 experimental replicates). Dashed blue line illustrates the cytoplasmic reference intensity. Error bars represent mean ± SEM. **d** Representative images from confocal microscopy of BEN-MEN-1 cells (GFP or PCDHGC3-GFP) illustrating expression of GFP and PCDHGC3 together with DAPI stain (n=3 experimental replicates). Scale bars: 25 µm. **e** Quantification of total levels of β-catenin in control and PCDHGC3-overexpressing BEN-MEN-1 cells (left). Comparison of the nuclear-to-cytoplasmic ratio of β-catenin signal binned by quartile (right). Data are derived from n=3 experimental replicates. For all boxplots, boxes represent the IQR with the median indicated by the centre line and whiskers extending to extreme outliers within 1.5x IQR. Source data are provided as a Source Data file.

Appendix

Uncropped Western Blots

Uncropped blots corresponding to Supplementary Fig. 19a

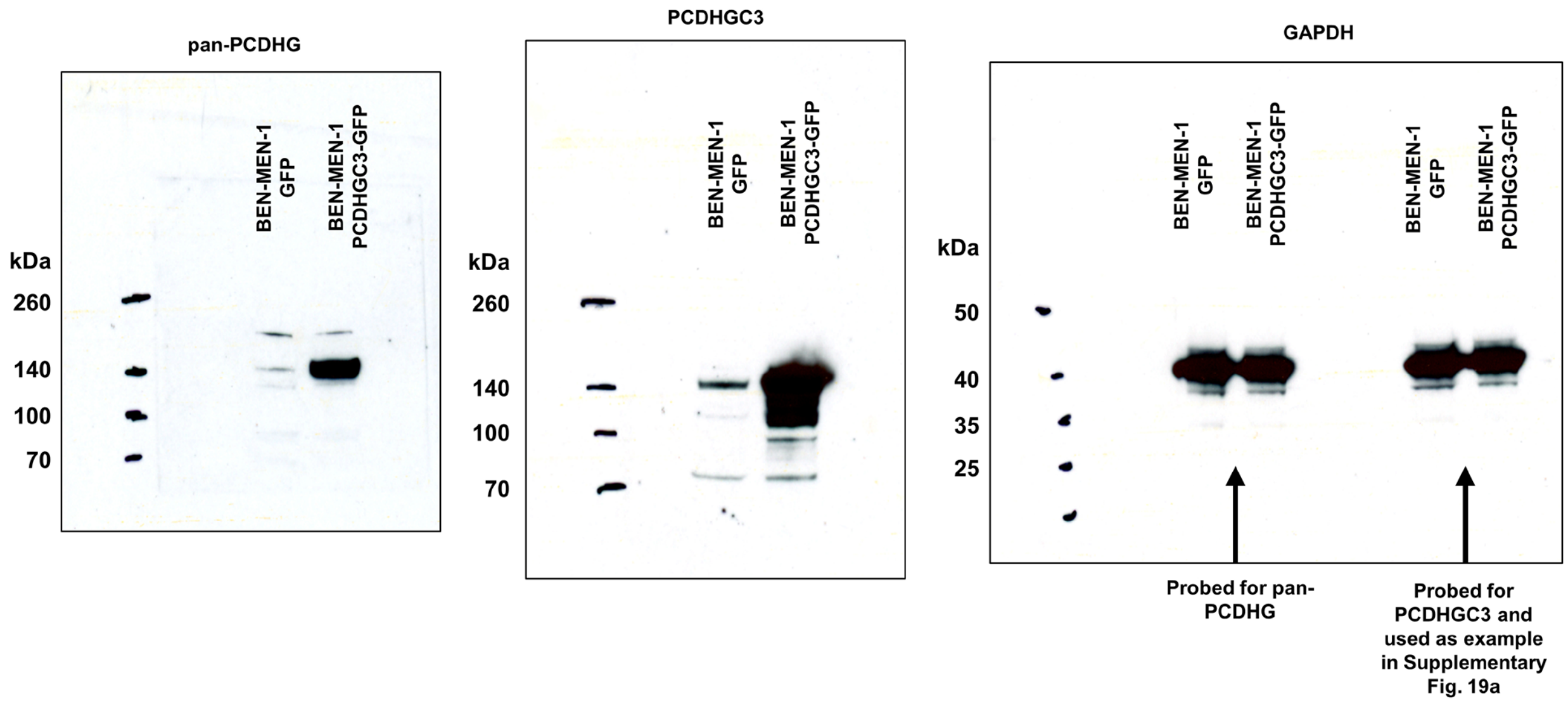

Supplementary References

1. Zhou, W., Triche, T. J., Jr., Laird, P. W. & Shen, H. SeSAMe: reducing artifactual detection of DNA methylation by Infinium BeadChips in genomic deletions. Nucleic Acids Res **46**, e123 (2018).
2. Nassiri, F., *et al.* A clinically applicable integrative molecular classification of meningiomas. Nature **597**, 119-125 (2021).
3. Choudhury, A., *et al.* Meningioma DNA methylation groups identify biological drivers and therapeutic vulnerabilities. Nat Genet **54**, 649-659 (2022).
