# Supplementary material for: DNA methylation profiling identifies long-range epigenetic silencing of clustered protocadherins as a key determinant of meningioma progression
Source: Nat Commun. 2026 Aug 29;17:9236. doi: 10.1038/s41467-026-77170-3 (PMC13526022; doi:10.1038/s41467-026-77170-3)
Supplement: Supplementary file 2 [file 41467_2026_77170_MOESM2_ESM.pdf]

Description of Additional Supplementary Information

Title: Supplementary Data 1

Description: Overview of clincial and molecular data from the Tübingen meningioma Discovery cohort (n=231).

Title: Supplementary Data 2

Description: Univariate and multivariate Cox proportional hazard regression analysis of Progression-free Survival in the discovery cohort. Hazard ratios and P values were estimated using Cox proportional hazards regression models with two-sided Wald tests.

Title: Supplementary Data 3

Description: Univariate and multivariate Cox proportional hazard regression analysis for effects of CNVs in the discovery cohort. Hazard ratios and P values were estimated using Cox proportional hazards regression models with two-sided Wald tests with Benjamin-Hochberg false discovery rate adjustment for multiple testing.

Title: Supplementary Data 4

Description: Overview of clinical and molecular data from the longitudinal cohort.

Title: Supplementary Data 5

Description: Differentially methylated probes in $METH^{high}$ (n=51) as compared to $METH^{low}$ (n=180) meningiomas in the Discovery cohort. Two-sided t-tests were performed for the group coefficient, and P values were adjusted for multiple testing using the Benjamini-Hochberg false discovery rate procedure.

Title: Supplementary Data 6

Description: Gene-centric enrichment analysis for hypermethylated probes in $METH^{high}$ meningiomas in the Discovery cohort. Enrichment was assessed using one-sided Fisher's exact tests, and P values were adjusted using the Benjamini-Hochberg FDR procedure.

Title: Supplementary Data 7

Description: Overview of patient characteristics used for RNA sequencing.

Title: Supplementary Data 8

Description: Differential gene expression analysis for $METH^{high}$ (n = 23) vs $METH^{low}$ (n = 162) meningiomas in the UCSF cohort. Statistics are derived from two-sided Wald tests with Benjamini-Hochberg adjustments for multiple testing.
