# Supplementary material for: DNA methylation profiling identifies long-range epigenetic silencing of clustered protocadherins as a key determinant of meningioma progression
Source: Nat Commun. 2026 Aug 29;17:9236. doi: 10.1038/s41467-026-77170-3 (PMC13526022; doi:10.1038/s41467-026-77170-3)
Supplement: Supplementary file 3 [file 41467_2026_77170_MOESM4_ESM.pdf]

nature portfolio

Corresponding author(s): Ghazaleh Tabatabai

Last updated by author(s): Aug 12, 2026

Reporting Summary

Nature Portfolio wishes to improve the reproducibility of the work that we publish. This form provides structure for consistency and transparency in reporting. For further information on Nature Portfolio policies, see our Editorial Policies and the Editorial Policy Checklist.

Statistics

For all statistical analyses, confirm that the following items are present in the figure legend, table legend, main text, or Methods section.

n/a	Confirmed	
☐	☒	The exact sample size (n) for each experimental group/condition, given as a discrete number and unit of measurement
☐	☒	A statement on whether measurements were taken from distinct samples or whether the same sample was measured repeatedly
☐	☒	The statistical test(s) used AND whether they are one- or two-sided<br>*Only common tests should be described solely by name; describe more complex techniques in the Methods section.*
☐	☒	A description of all covariates tested
☐	☒	A description of any assumptions or corrections, such as tests of normality and adjustment for multiple comparisons
☐	☒	A full description of the statistical parameters including central tendency (e.g. means) or other basic estimates (e.g. regression coefficient) AND variation (e.g. standard deviation) or associated estimates of uncertainty (e.g. confidence intervals)
☐	☒	For null hypothesis testing, the test statistic (e.g. F, t, r) with confidence intervals, effect sizes, degrees of freedom and P value noted<br>*Give P values as exact values whenever suitable.*
☒	☐	For Bayesian analysis, information on the choice of priors and Markov chain Monte Carlo settings
☒	☐	For hierarchical and complex designs, identification of the appropriate level for tests and full reporting of outcomes
☐	☒	Estimates of effect sizes (e.g. Cohen's d, Pearson's r), indicating how they were calculated

Our web collection on statistics for biologists contains articles on many of the points above.

Software and code

Policy information about availability of computer code

Data collection	No software was used to collect data in this study.
Data analysis	Data analysis of DNA methylation arrays was performed in R (v4.4.2) with the SeSAMe (v1.24.0). Principal component analysis (PCA) was performed using the prcomp function in R. Probes best descriminating benign from maligant meningiomas were extracted based on PCA findings and used for unsupervised hierarchical clustering. For several top scoring probes subsets, the optimal number of clusters k was evaluated using both the Elbow method, based on within-cluster sum of squares, and the Silhouette method, based on average silhouette width across samples. Following cluster number selection, consensus clustering was applied to each probe subset using a resampling-based framework to evaluate clustering stability. ConsensusClusterPlus (v1.70.0) was used to generate consensus matrices and final cluster assignments, from which consensus cluster prediction (CCP) scores were calculated to quantify within-cluster stability. In addition, concordance of tumour cluster assignments across different probe subsets was assessed using the adjusted rand index.<br>We used a machine learning approach to assign meningiomas from our cohorts to Immunogenic, NF2-Wildtype, Hypermetabolic, and Proliferative groups based on a previous cohort. As a default, we selected the top 10.000 most variable methylation probes to set up a classifier with the following algorithms available within the caret R package (v7.0-1): random forest, support vector machine, k-nearest neighbors, nearest shrunken centroids and stochastic gradient boosting. The final model was selected based on the highest accuracy and Cohen's Kappa. K-means clustering of samples was performed using the fviz_nbclust function of the factoextra R package (v1.0.7). Differential DNA methylation in meningioma methylation clusters, or in between meningiomas, as compared to normal meninges, was assessed using the DML function implemented in the SeSAMe package. Statistical significance was assessed with P values adjusted for multiple testing using the Benjamini-Hochberg false discovery rate. CpG sites with an absolute estimated beta value difference of 0.2 for a given contrast and adjusted P value of < 0.05 were considered differentially methylated. Trajectory analysis in PCA plots defined by DNA methylation probes varying between meningioma settings and corresponding pseudotime analyses were performed using the slingshot (v2.14.0). CNV profiles from DNA methylation data were generated using the conumee2 (v2.1.2).

Libraries for mRNAseq were sequenced as paired-end 50bp reads on an Illumina NovaSeq6000 (Illumina) with a sequencing depth of approximately 25 million clusters per sample. RNA raw data QC and processing were performed using megSAP (version 0.2-135-gd002274) combined with ngs-bits package (version 2019_11-42-gflb98e63). Reads were aligned using STAR v2.7.3a to the GRCh37, and alignment quality was analyzed using ngs-bits. Normalized read counts for all genes were obtained using Subread (v2.0.0) and edgeR (v3.26.6). Raw count data was analyzed using the DESeq2 (v1.46.0)
For survival analyses, Cox' proportional hazards assumption was tested using the ggcoxzph function implemented in the survminer R package (v0.5.0). Univariate and multivariate survival analyses were performed using the survival R package (v3.8-3) using Cox proportional hazards regression modeling. Integrated discrimination improvement (IDI) and net reclassification improvement (NRI) were performed using the survIDINRI R package (v1.1-2)
QuPath (v0.5.1) was used for the analysis of immunohistochemistry stainings of tissue microarrays.
Gen5 software (version 3.16, BioTek) was used for the analysis of fluorescent images from confocal microscopy.
All code has beeen deposited at GitHub (https://github.com/MerkLab/Meningioma_DNA_methylation_project).

For manuscripts utilizing custom algorithms or software that are central to the research but not yet described in published literature, software must be made available to editors and reviewers. We strongly encourage code deposition in a community repository (e.g. GitHub). See the Nature Portfolio guidelines for submitting code & software for further information.

Data

Policy information about availability of data

All manuscripts must include a data availability statement. This statement should provide the following information, where applicable:

- Accession codes, unique identifiers, or web links for publicly available datasets
- A description of any restrictions on data availability
- For clinical datasets or third party data, please ensure that the statement adheres to our policy

TThe raw DNA methylation data and RNAseq data generated in this study have been deposited in the GEO database under accession code GSE304097 (https://www.ncbi.nlm.nih.gov/geo/query/acc.cgi?acc=GSE304097). Publicly available datasets used in this study include DNA methylation array and RNAseq data (https://www.ncbi.nlm.nih.gov/geo/query/acc.cgi?acc=GSE180061 and https://www.ncbi.nlm.nih.gov/geo/query/acc.cgi?acc=GSE183656)19,20. Raw DNA methylation data from the normal meningeal samples30 cannot be deposited in a public or third-party controlled-access repository because the data derive from only two individuals who donated their bodies through the University of Copenhagen Body Donation Program. Their use is governed by the donor framework, institutional stewardship requirements, and project-specific data-sharing agreements that do not permit onward transfer or repository deposition without separate authorization. The data are available through a controlled-access procedure administered directly by Andrea Daniela Maier and Tiit Mathiesen from the University of Copenhagen, Denmark. Access may be granted to qualified researchers at recognized academic or non-profit research institutions when the proposed use is compatible with the donor and institutional framework and the applicant agrees to a project-specific Data Use Agreement. Requests should be submitted in writing to andrea.maier@regionh.dk and tiit.illimar.mathiesen@regionh.dk. Requests will be acknowledged within five working days and decisions will be provided within 20 working days after receiving all required documentation. Approved access will normally be granted for 12 months and may be renewed following review. Additional preprocessed data of DNA methylation arrays and a folder structure compatible with our submitted code are available on figshare (https://doi.org/10.6084/m9.figshare.32043249). The remaining data are available within the Article, Supplementary Information or Source Data file.

Research involving human participants, their data, or biological material

Policy information about studies with human participants or human data. See also policy information about sex, gender (identity/presentation), and sexual orientation and race, ethnicity and racism.

Reporting on sex and gender	Sex information can be retrieved from Supplementary Data 1 + 4 + 7. Sex and gender information was determined based on self-report. Sex was evaluated as a clinical covariate in survival analyses. However, the study was not designed or powered to assess sex-specific effects of the methylation signature, and no formal interaction analyses were performed. All sex chromosome-associated probes from DNA methylation arrays were excluded. All patients provided written consent to the use of tumour material and clinical data for research purposes including publication.
Reporting on race, ethnicity, or other socially relevant groupings	We did not collect data on race, ethnicity, or other socially relevant groupings on the subjects that were included in this study.
Population characteristics	A description of population statistics is included in the manuscript, including patient age, tumour location, and other tumor-associated charateristics.
Recruitment	Patients were recruited in the course of their presentiation in the clinic based on availability. All patients provided written informed consent to the use of tumor material and clinical data for research purposes. This consent includes publication of the associated data.
Ethics oversight	The local Ethics Review Board at the Medical Faculty of the Eberhard-Karls-University Tübingen, Germany, approved the collection and usage of tumour material (application number 191/2021BO2).

Note that full information on the approval of the study protocol must also be provided in the manuscript.

Field-specific reporting

Please select the one below that is the best fit for your research. If you are not sure, read the appropriate sections before making your selection.

- [x] Life sciences
- [ ] Behavioural & social sciences
- [ ] Ecological, evolutionary & environmental sciences

For a reference copy of the document with all sections, see nature.com/documents/nr-reporting-summary-flat.pdf

Life sciences study design

All studies must disclose on these points even when the disclosure is negative.

Sample size	Sample size was defined by sample availability. No sample-size calculation was performed.
Data exclusions	Tumor diagnosis of meningioma was done by a certified pathologist and confirmed by DNA methylation-bases classification (www.molecularneuropathology.org). Samples were only excluded for inferior sample quality, while blinded to any molecular or clinical characteristics.
Replication	Each experiment was performed at least in three biological replicates.
Randomization	No trials requiring randomization are included in the study.
Blinding	Investigators for functional studies on beta-catenin were blinded to conditions during analysis. Other than that, no blinding was performed.

Reporting for specific materials, systems and methods

We require information from authors about some types of materials, experimental systems and methods used in many studies. Here, indicate whether each material, system or method listed is relevant to your study. If you are not sure if a list item applies to your research, read the appropriate section before selecting a response.

Materials & experimental systems

n/a	Involved in the study	
☐	☒	Antibodies
☐	☒	Eukaryotic cell lines
☒	☐	Palaeontology and archaeology
☒	☐	Animals and other organisms
☒	☐	Clinical data
☒	☐	Dual use research of concern
☒	☐	Plants

Methods

n/a	Involved in the study	
☒	☐	ChIP-seq
☐	☒	Flow cytometry
☒	☐	MRI-based neuroimaging

Antibodies

Antibodies used	Antibodies used for western blot analyses:<br>anti-PCDHG-pan-gamma (S159-5, Thermo Fisher, 1:500), anti-PCDHGC3 (MAB8364, R&D, 1:250), and anti-GAPDH (D4C6R, Cell Signaling, 1:1000)<br>Antibodies used for flow cytometry:<br>anti-beta-catenin-PE-conjugated (12-2567-42, ThermoFisher, 1:50)<br>Antibodies used for immunocytochemistry:<br>anti-GFP (ab13970, Abcam, 1:500, chicken), anti-beta-catenin (D10A8, Cell Signaling, 1:100, rabbit)<br>Antibodies used for immunohistochemistry:<br>anti-beta-catenin (clone 14, BD Transduction Laboratories #610153, 1:500)
Validation	All antibodies used in this study were commercially available and extensively validated by the respective company. Validation data can be found on the corresponding websites.

Eukaryotic cell lines

Policy information about cell lines and Sex and Gender in Research

Cell line source(s)	IOMM-Lee cells were purchased from ATCC. BEN-MEN-1 cells were purchased from the DSMZ, Leibzig, Germany. HBL-52 cells were puchased from Cytion. NCH93 and KT21-MG1 meningioma cell lines were provided by Professor Christel Herold-Mende and Professor Christian Mawrin, respectively.
Authentication	All cell lines were authenticated by STR-profiling.
Mycoplasma contamination	All cell lines regularly tested negatve for mycoplasma.
Commonly misidentified lines (See ICLAC register)	No commonly misidentified cell lines were used.

Plants

Seed stocks

Report on the source of all seed stocks or other plant material used. If applicable, state the seed stock centre and catalogue number. If plant specimens were collected from the field, describe the collection location, date and sampling procedures.

Novel plant genotypes

Describe the methods by which all novel plant genotypes were produced. This includes those generated by transgenic approaches, gene editing, chemical/radiation-based mutagenesis and hybridization. For transgenic lines, describe the transformation method, the number of independent lines analyzed and the generation upon which experiments were performed. For gene-edited lines, describe the editor used, the endogenous sequence targeted for editing, the targeting guide RNA sequence (if applicable) and how the editor was applied.

Authentication

Describe any authentication procedures for each seed stock used or novel genotype generated. Describe any experiments used to assess the effect of a mutation and, where applicable, how potential secondary effects (e.g. second site T-DNA insertions, mosiacism, off-target gene editing) were examined.

Flow Cytometry

Plots

Confirm that:

- [x] The axis labels state the marker and fluorochrome used (e.g. CD4-FITC).
- [x] The axis scales are clearly visible. Include numbers along axes only for bottom left plot of group (a 'group' is an analysis of identical markers).
- [x] All plots are contour plots with outliers or pseudocolor plots.
- [x] A numerical value for number of cells or percentage (with statistics) is provided.

Methodology

Sample preparation

For FACS, we used parental BEN-MEN-1, HBL-52, NCH93, IOMM-Lee, and KT21-MG1 meningioma cell lines, or IOMM-LEE cell and BEN-MEN-1 overexpressing either GFP alone or in combination with the protocadherin PCDHGC3. Cell were prepared for cytoplasmic only (0.1% saponin buffer) or whole cell extracts (0.5% Triton X-100 in 1x FOXP3 permeabilization buffer (Invitrogen, Thermo Fisher Scientific)).Cells were washed and incubated with β-catenin antibody (15B3, PE-conjugated, Invitrogen, 1:50) for 30 minutes at RT. Finally, the cells were washed and resuspended in 200 μL of FACS buffer (PBS containing 2% FCS and 0.5M EDTA)

Instrument

Measurements were peformed on a MACSQuant Analyzer 10 machine.

Software

We used FloJow (v10.10.0) for the analysis of FACS data.

Cell population abundance

FACS was performed to select for viable, single cells.

Gating strategy

Live cells were identified by size (forward scatter) and granularity (back scatter), singlet gating by area to height of foward scatter, GFP or beta-catenin positive cells by fluorescence intensity to forward scatter height.

- [x] Tick this box to confirm that a figure exemplifying the gating strategy is provided in the Supplementary Information.
