# Supplementary material for: DNA methylation profiling identifies long-range epigenetic silencing of clustered protocadherins as a key determinant of meningioma progression
Source: Nat Commun. 2026 Aug 29;17:9236. doi: 10.1038/s41467-026-77170-3 (PMC13526022; doi:10.1038/s41467-026-77170-3)
Supplement: Supplementary file 4 [file 41467_2026_77170_MOESM5_ESM.pdf]

nature portfolio

Peer Review File

DNA methylation profiling identifies long-range epigenetic silencing of clustered protocadherins as a key determinant of meningioma progression

Corresponding Author: Professor Ghazaleh Tabatabai

This file contains all reviewer reports in order by version, followed by all author rebuttals in order by version.

Version 0:

Reviewer comments:

Reviewer #1

(Remarks to the Author)
The authors present a coherent and clinically relevant risk axis for meningioma based on a DNA hypermethylation program centered on clustered protocadherins. The simple two-state label (METHlow / METHhigh) may clarify prognosis across existing taxonomies. To achieve practice-changing impact, the authors may illustrate direct consequences for treatment decisions, and align explicitly with the recent cIMPACT-NOW update.

Major comments

- The authors showed that METH clusters cut across published groups and improve prognostication, with particular value in intermediate-risk meningiomas. Please indicate when testing is recommended.
- Please translate the METHhigh/METHlow label into a decision aid. The authors are encouraged to illustrate when and how METH status would alter management in terms of surveillance intervals, timing of adjuvant RT. etc.. A simple decision tree (inputs: EOR, 1p status, integrated risk score/methylation class/molecular subgroup, METH label: "observe", "discuss early RT", "adjuvant RT recommended") would help clinicians operationalize your findings.

- Please separate outcomes by pattern of failure (local bed vs. distant intracranial or spinal) and prior RT exposure. Methylation programs that predict local regrowth may not predict distant seeding, and prior RT can reshape both CNVs and genomic profiles. Provide additional information to contextualize molecular changes in recurrent meningiomas. Tie these dynamics to time-to-recurrence and interval therapies.

- TERT promoter mutations are a recognized high-risk marker in meningioma. Please report TERT status.

- The data suggest METHhigh enriches for early progression. Please provide exploratory analyses of RT benefit by METH status and whether adjuvant RT preferentially benefits METHhigh.

- Please contextualize the METH label within cIMPACT-NOW update 8 recommendations for meningioma grading and risk parameters.

Minor comments
- Extended Figure 2D (hypermetabolic subgroup): Adapt the placement of its heading.

Reviewer #2

(Remarks to the Author)
Review

The authors present a very interesting integrative study on meningioma progression, with a particular focus on global DNA methylation profiling. They provide original findings regarding the the trajectory of meningioma progression, and the

suppressive role of protocadherins, which are involved in the β-catenin/WNT signaling pathway. They also demonstrate the significant prognostic value of global methylation status in relevant subgroups, such as the intermediate subgroup and 1p-deleted meningiomas. In addition to methylome analyses, their approach highlights the prognostic relevance of β-catenin immunohistochemistry.
The results appear robust, being supported by a discovery cohort, a longitudinal cohort, and validation in a publicly available cohort. Moreover, the authors performed in-cellulo experiments to further investigate the role of protocadherins.

Minor concerns

1- Was the proportion of METH^high cases higher in the proliferative group? Was the METH^low vs. METH^high signature significantly associated with survival in this proliferative group? This should be clarified in the Results.

2- Was there a correlation between the Ki-67 labeling index (a well-established prognostic marker) and the hypermethylation signature?

3- A few words about the prognostic value of β-catenin (IHC) should be added to the Abstract.

4- In the Introduction, first sentence: should this refer to arachnoid cap cells or to the subdural inner layer (see studies from Kalamarides et al.)?

5- Fig 6i, magnification of IHC picture should be higher (focusing on nuclear staining, wich is not evident on the illustration).

Style
In the Results section, the use of present vs. past tense should be standardized.

Reviewer #3

(Remarks to the Author)
The manuscript with a title „DNA methylation profiling identifies long-range epigenetic silencing of clustered protocadherins as a key determinant of meningioma progression" provides data on DNA methylation profiles from 231 meningioma samples. It identifies clustered protocadherins as determinants of meningioma progression. Although the role of clustered protocadherins in the progression of many different cancers have been demonstrated and their methylation has been demonstrated in meningiomas before, the current study adds additional value due to its complexity and the large number of clinical samples analyzed. This research could thus contribute to the establishment of new biomarkers for the classification of meningiomas, the guidance of therapy and the development of new treatment options for affected patients. Significant differences in methylation patterns between normal meninges and those observed during meningioma development are convincingly demonstrated and evaluated using various methods. In addition to the methylation patterns and their comprehensive analysis, the authors present gene expression data from cancers with high and low methylation and demonstrate a correlation between these two data sets. They provide valuable experimental data supporting the overall hypothesis. The overexpressing one of the clustered protocadherins, PCDHGC3, in the IOMM-Lee cell line confirmed its strong effects on the regulation of Wnt-signaling. They provide evidence that epigenic silencing and the PCDHGC3-mediated regulation of b-catenin activity, known from the literature, contribute to poor clinical outcomes in meningioma patients. I have just a few comments:

It would be interesting to describe in the introduction the current classification schemes used clinically for meningiomas and to describe the potential of the presented data for defining new clinical classification markers either in the introduction or in the discussion

Additional analysis of PCDHGC3 and / or other clustered protocadherins levels in published meningioma mRNA sequencing datasets and their correlation with clinical parameters and overall survival would contribute to the establishment of PCDH genes as novel biomarkers in meningiomas.

Reviewer #4

(Remarks to the Author)
Key results and significance
This paper presents high-methylation and low-methylation subgroups for meningioma that help to stratify the mid-risk class from currently available classifiers. It proceeds to seek biological function underlying these subgroups that may relate to the differential outcome. While further understanding the prognosis of these challenging cases is of value, there is sufficient concern with the analytical methods to reduce my confidence in the presented results.

Data, methodology, analytical approach, and validity

1. Technical factors that may impact the analysis outcomes are not addressed.
a. Explain how the tumor content was verified in the FFPE tissue samples before DNA and RNA extraction (lines 476-7, 552).
b. Examine the extent to which PCA is impacted by technical artifacts – % tumor content in tissue sample, batch or run-order, age of the FFPE block.

2. Survival modeling has temporality issues that may impact conclusions
a. Define the PFS outcome for primary and for recurrent cases
b. Primary and recurrent cases likely need to be modeled separately. Simple covariate adjustment makes strong assumptions that primary and recurrent tumors have the same baseline hazard and association with predictors.
c. Adjuvant RT is not a baseline factor for primary diagnoses, and it is not clear if primary or post-recurrence adjuvant RT is modeled for recurrent diagnoses. To prevent immortal time bias, adjuvant RT must be included as a time-varying covariate. Caution should also be used since receipt of adjuvant RT is tied to grade and EOR which are in the model.
d. Caution is needed when reporting and visualizing time-to-event data, so that censoring time is addressed. Figure ED1d does not account for censoring time and cannot be interpreted.
e. Mean and SD are not appropriate summaries for survival data in this study (e.g., lines 207-208). The KM and Reverse KM methods will give appropriate median and IQR estimates, as possible.

3. Pseudoreplication in longitudinal analysis may lead to Type 1 error
a. Simple one-way ANOVA cannot be used when the groups include the repeated measure categories (e.g. Fig 3b, line 522)
b. The "nested t-test" (e.g., line 237) is not a precise description and may indicate pseudoreplication. Please clarify.
c. Clustering of primary-recurrent samples within case was not passed to the trajectory analysis algorithm

4. Methods are not fully aligned with presented text or submitted R-code
a. Sample-level QC evaluation is described (lines 483-485) but not present in the code. Please verify.
b. Clarify in the methods that p-value filtering excludes probes with any missing data, creating a complete case analysis (lines 485-488)
c. Exploration of PC1 – PC3 was conducted to select PC1, including checking association with clinical factors. This is not described in the methods.
d. The use of multiple clustering models to apply a prior classifier (lines 508-516) is unclear and is not included among the submitted code. The results indicate that gradient boosting (lines 104-5) is used for this classification task, but this method is not described and no code is provided.
e. The work conducted in the submitted code file for differential methylation analysis is not well described in the methods. Also, the data pre-processing and filtering steps in this code file are different than the steps in the code file with PCA and clustering. Please ensure that the analysis-ready data for the Discovery set is consistent or explain the rationale for the difference.
f. Multiple sample sets are introduced in the code but not described in the methods – e.g., normal meninges, characteristics of the UCSF dataset, which and how many cases were profiled with RNA seq.

5. Generation of the Meth-low vs Meth-high groups
a. PC 1 captures more variance with 10K probes, but the 50K probe subset is selected, please clarify
b. The elbow of the scree plot for the hierarchical clustering is not striking and a 3rd mid-methylation cluster appears reasonable.

Suggested improvements
It is suggested that the authors consider the impact of analyzing primary and recurrent tumors separately. Recurrent tumors tend to be molecularly distinct due to prior treatment and the mix of primary and recurrent tumors may be impacting outcomes. It is also suggested that a three-cluster solution for the generated methylation groups be considered. Figure 1c is suggestive of a mid-level methylation group that separates a fairly distinct NF2-wt subset for the Meth-low group.

Clarity and context
Figure panels are densely packed and annotation is difficult to read. There is heavy reliance on color to distinguish groups. It appears that "Molecular Group" is used to refer to the Toronto clustering, however, the DKFZ clustering is also under the header of molecular group in figure 1 and elsewhere.

Your expertise
While I read the cell experimentation, this bench work is outside of my area of comfort to review in depth.

Version 1:

Reviewer comments:

Reviewer #2

(Remarks to the Author)
The authors have responded to each of the reviewers' comments and significantly improved the manuscript.

Minor concerns:

→ Supplementary Fig. 7: Decision tree.
There are some ambiguities in different parts of this decision tree; e.g.:

GTR in grade 2: the direct pathway to “shorter surveillance time frames/discuss early RT” should be deleted if methylation clustering is recommended.

Grades 1 and 2: if 1p and 22q are present, this should probably lead directly to “shorter surveillance time frames/discuss early RT”.

→ Extended Data Fig. 4.
The HR ratio and p-values seem to be in contradiction with the well-separated survival curves. Is it a log-rank test or Cox proportional hazards models stratified by tumour setting (primary vs. recurrent)? This should be added to the legend.

→ Extended Data Fig. 5.
Replace “p <” with “p =”.

→ Fig. 7.
Nuclear staining is still not evident. I mainly observe, in METHhigh cases, cytoplasmic perinuclear staining. Is it possible to illustrate this with more evident nuclear relocalization?

→ Methods, line 732:
Was there a specific tool in QuPath to quantify cytoplasmic and nuclear staining separately?

→ Radiotherapy and PFS, lines 176–188.
In this retrospective study, because of uncontrollable biases, the term “effect” of RT is not appropriate; it should be replaced with “correlation [of both prior and adjuvant RT] with (…)”.

→ Typo:

In the Extended Data Fig. 10 title: BEN-MEN-1 instead of “BEN-MNE-1”.

Line 394: “for an additional 529 meningiomas”; suggestion to replace with “for 529 additional meningiomas” or “for an additional cohort of 529 meningiomas”.

(Remarks on code availability)

Reviewer #3

(Remarks to the Author)
The authors have revised the manuscript in accordance with the reviewers’ comments and added valuable new data and analyses. I have no further concerns.

(Remarks on code availability)

Reviewer #4

(Remarks to the Author)
Thank you for the thoughtful and complete address of the prior feedback. The following comments are minor edits for clarity.

• Line 129-130 - progression is not directly measured please change "most associated with meningioma progression" to "most associated with meningioma progression risk groups"
• Line 671 - typo: please change "war" to "was"
• Line 784-785 - The indicated R code cannot be found in the repository. Please investigate why Figure 7c and 7f do not have error for the cytoplasmic fold change values? If these are group means, then variability would be expected around the mean. If these are 1.0 by design from a paired assessment, then consider a reference line since the cytoplasmic value is built into the fold change.
• Extended figure 4 f-g -- The KM plot uses 4 groups that do not account for the immortal time bias of adjuvant RT and other factors that impact decision to add RT are missed (see code file 06_survival beginning line 850). Survival curves for RT groups are likely optimistic. However, an appropriate model was used to estimate the HR. Please indicate in the legend that HR estimates are from an adjusted model.
• Since the hypotheses for Kruskal-Wallis and ANOVA multi-group testing differ, when a set of tests are being conducted it is best practice to be consistent so that the results can be meaningfully interpreted between tests. Please consider converting the few ANOVA assessments to KW. (see code file 09_discovery_cnv, beginning line 367)

(Remarks on code availability)
Code submission - In reference to the Nature Research code submission check list, this code submission is not for a standalone product, but provides 12 scripts that accomplish most of the analysis in the manuscript. The readme file is a simple abstract for the project, but does provide the operating system and the version numbers for some of the packages that are used. The readme file references a dataset that will be available in GEO and that metadata is in the manuscript. I

assume this is in reference to the 231 cases provided by the author as the discovery set and that the manuscript in question is the one under review. This would need clarification once the study is published and unblinded. GEO archive numbers, or other access information, should also be added for all datasets used in the analysis, including the UCSF, Nassiri, normal meninges, and cell line methylation datasets and the RNA seq dataset. The readme file does not directly refer to any of the script files regarding their function or expected output. If this is not in the readme, then each of the 12 files should have a header connecting the code to the manuscript and with references for the called datasets. While I did not access the data to run each script, I was able to load the packages, and I read through the scripts. The readme or script headers would be further improved by indicating which packages are found in CRAN vs Bioconductor. While some of the packages may be obvious to an experienced reader, a novice reader may struggle.

Above what is stated, there are no concerns for files 01-05. In files 6-12, the library calls have not been consolidated to the top of the script. If the later load, or reload, is needed (e.g., masking of functions) please clarify.

06_Survival.R - line 729 - introduces a new library (stringr)
07_trajectory_analysis.R - Line 229 reloads the library (ggstatsplot); Line 715 introduces a new library (RColorBrewer); Line 784 introduces a new library (ggplot2)

09_Discovery_CNV.R -
Library installation and loading needs clean-up
• Bioconductor is indicated, however, there are redundant calls to install "BiocManager"
• Line 1-4 - the installation call for 'conumee' is missing
• Line 21 - "ComplexHeatMap" is installed, but not loaded until line 258
• Line 23 - "ggbeeswarm" is installed but not loaded
• Line 26 - Install of 'conumee2' package appears to be proprietary from a local library so this cannot be replicated
• Libraries repeatedly added - line 42 (stringr), 217 (stringr)
• Line 539 - ggplot2 reloaded
Multiple txt and csv files are loaded throughout from local directories - please indicate which other script generated these files, provide an external source for these files, or describe the contents so the user can generate a comparable file

10_longitudinal_CNV.R
Library installation and loading needs clean-up
• Conumee2 is loaded then removed then installed from a proprietary local library - it is reloaded in line 734
• Libraries are reloaded - Conumee (line 393), sesame (lines 417, 735), ggplot2 (line 1221)
• Libraries added
○ the package 'stringr' is repeatedly loaded on lines 35, 102, 152, 201, 245, 454, 560
○ lines 498 (lme4), 499 (lmerTest), line 500 (emmeans), line 604 (pheatmap), line 631 (data.table), 667 (dplyr), 767 (biomaRt), line 1316 (ComplexHeatmap)
• Etc.
Clean up unused attempts - code references using old and new conumee
• Line 394 - "important to use conumee, not conumee2, does not work"
• Line 414 - "try with old conumee package"
• Line 416 - "this is a try with the new package"
Isses with coding seem to persist -
• Line 758 - #try using CNV.define_detail for cancer census genes (should however be covered by sig_cgenes below, but somehow is not)
• Line 766 - #does not seem to work, problem with seqnames which are not in seqinfo, means that by default anno function uses seqinfo other than what is given in seqnames
• Line 1458 - ###new try
Provide a header and comments for the function created starting on line 925.

11_RNAseq.R
Lines 19, 22 - Origin of the data is not clear
New library calls - line 149-150

12_Classifier.R
At the top, indicate that the webapp is being built - beginning with line 108

Reviewer #5

(Remarks to the Author)
In this manuscript the authors perform EPIC methylation profiling on a cohort of 231 meningiomas, then propose a binary classification scheme based on 1000 probes with strongest association to PC1. They find that this scheme recapitulates previously described paradigms (Fig 1d). Intermediate risk (WHO grade 2, Hypermetabolic) were balanced between METH low/high; and Meth low/high could stratify outcomes of WHO grade 2 tumors (but not hypermetabolic or Intermediate integrated risk; Fig 2c). Analysis of longitudinal samples showed expected differences between progressive and benign cases (WHO grade, number of CNVs, etc), and between meningiomas and a small set of meninges samples previously published. They examine genes that show statistical hypo-methylation (23) or hyper-methylation (744) in the METH high subgroup, and find enrichment of HOX genes and protocadherins, which also showed differential expression in some cases.

As shown in previous studies of other cancers, the authors suggest that silencing of protocadherin in high-grade meningiomas may result in more nuclear localization of B-catenin, which is known to play a role in meningioma biology. Indeed, overexpression of PCDHGC3 in IOMM-LEE (but not BEN-MEN-1) caused increased cytoplasmic localization of B-catenin, as well as decreased overalabundance of B-catenin.

The authors have addressed the comments provided by Reviewer #1. However, included in the rebuttal is the statement “However, even when compared to purely molecular-based or integrated classification systems, we find more accurate prognostication for intermediate-risk meningiomas”, which does not seem to be supported by Fig 2c (see further discussion below).

Comments:

While the authors have demonstrated that their binary methylation classification provides additional prognostic benefit on top of WHO grading, this has now been demonstrated in many large-cohort publications (PMIDs 28314689, 34433969, 34618539, 35534562 among others). To be of added clinical value, this manuscript would need to show a direct prognostic comparison of this proposed classification to these other systems. If the claim is that this new system is only helpful for “intermediate” cases, more evidence is needed to support this (currently figure 2c seems to show that it does not provide prognostic benefit).

Line 127: "Furthermore, within classification groups with difficult estimation of prognosis commonly referred to as intermediate-risk meningiomas, methylation clustering stratified into groups with different clinical behaviour, with METHhigh cluster patients having a significantly or moderately worse prognosis (Fig. 2c and Extended Data Fig. 4d)."

Fig 2c and Extended Data Fig 4d show that Meth Low/High can stratify outcomes of WHO grade 2 tumors, while Fig 2c seems to show that Meth Low/High is unable to stratify outcomes of Hypermetabolic or Intermediate Risk (Maas) meningiomas ($p > 0.05$). This needs to be clearly and explicitly stated in the main text, as it suggests that the Meth Low/High classification does not provide any further prognostic benefit over currently proposed schemes. Unless I have mis-understood, this is in direct contradiction to how the text is currently worded.

Line 227: "our data suggest that DNA hypermethylation in METHhigh tumours is an independent mediator of meningioma progression, allowing for further prognostic sub-stratification of 1p-altered meningiomas."

Do other methylation predictors also stratify 1p loss cases, or is this a unique feature of the binary classification proposed here?

Line 258: "we identified several candidates to exclusively occur in high-grade tumours including gene amplifications of MDM2, a negative regulator of p53 signalling, and gene deletion events for tumour suppressors such as VHL and CDKN2A/B (Fig. 4d,e)."

Is there statistical testing that supports enrichment of these genes in high-grade cases? How were these specific genes selected for the figure, there are no p values shown. Contrary to the statement in the main text (“exclusively occur in high-grade”), both MDM2 amplification and VHL deletion appears to occur in a WHO grade 1 tumor.

Line 312: "We noted that a substantial number of genes associated with DNA hypermethylation formed part of several gene clusters, including HOX genes and protocadherin clusters (Fig. 6d and Extended Data Fig. 8c)."

Methylation changes in HOX genes have been well reported previously in meningiomas (PMIDs: 22735029, 23349797, 22102699, 31069492; and 30202034 as noted in the discussion); and to a lesser extent for protocadherins (PMIDs: 36551712). The authors should comment on these papers and if there are any unique findings from this new analysis.

Line 377: "In contrast, we did not detect any changes in the nuclear-to-cytoplasmic ratio of B-catenin when overexpressing PCDHGC3 in the benign cell line BEN-MEN-1."

How do you explain this? It is not clear to me why protocadherins would regulate B-catenin localization (and possibly production) only in the context of aggressive meningiomas. More broadly, I am concerned that this experiment has only shown an effect in a single meningioma cell line. It is possible that the observed effect on B-catenin localization is not due to benign vs. aggressive meningiomas (n = 1 each), but rather an effect of IOMM-LEE vs. BEN-MEN, which have many differences. To be more convincing, the authors should replicate this finding in the other cell lines that they have available (HBL-52, NCH93, KT21-MG1 are mentioned), in addition to primary cells if possible. This is particularly needed given the known hyper-aggressive phenotype associated with IOMM-LEE, which may poorly replicate meningioma biology.

Line 57: "no recurrent genetic aberrations other than NF2 have been identified thus far in high-grade meningiomas,"

This does not seem accurate, I think you mean “genetic coding mutations”. TERTp mutations and CDKN2A/B loss is sufficient for high-grade classification by the WHO, and CNVs such as 1p loss (among others) are well associated with aggressiveness. Alterations in BAP1 have also been proposed to be enriched in high-grade cases.

Line 415: "In contrast, CNS WHO grade 2 meningiomas, encompassing the majority of hypermetabolic/integrated

intermediate-risk tumours that are characterized by a considerable heterogeneity in their clinical course, showed a more balanced assignment to either METHlow and METHhigh clusters."

However, you show that there is not a statistical difference conferred by METH high/low for hypermetabolic/integrated intermediate-risk. This should be acknowledged explicitly here. Similar changes should be made to the statement “it may be particularly useful to predict clinical outcome for meningiomas currently classified as intermediate-risk and thereby inform clinical..”, which should explicitly say “Grade 2” instead of “intermediate risk”, in order to match your results.

Line 427: "Together, our data reveal a hypermethylation signature directly associated with meningioma progression, and this association is independent of previously described molecular groups defined mainly based on DNA methylation."

From my understanding this statement is not accurate, there is in fact a strong association of previously described molecular groups and your hyper-methylation signature (Fig 1d). The sentence should be clarified. Similar adjustments should be made to line 476 “Our study reveals a prognostically relevant DNA hypermethylation signature that acts independently of molecular groups defined by global DNA methylation”.

(Remarks on code availability)
The code is easily accessible and well documented on the Anonymous GitHub website.

Version 2:

Reviewer comments:

Reviewer #2

(Remarks to the Author)
The authors have responded the reviewers’ comments and significantly improved the manuscript.

Minor typo.:
454 studies in larger, multicenter cohorts are needed TO improve resolution for these tumours.

(Remarks on code availability)

Reviewer #5

(Remarks to the Author)
The authors have corrected the concerns raised in my previous review.

(Remarks on code availability)
The code is easily accessible and well documented on the Anonymous GitHub website.

Reviewer #6

(Remarks to the Author)
We reviewed the revised manuscript with a focus on the issues previously raised by Reviewer #4. Overall, the authors have addressed most of prior concerns. We only have a few technical comments.

1. Statistical test reporting for Fig. 3c remains inconsistent

Reviewer #4 recommended using Kruskal-Wallis tests consistently for the relevant multi-group comparisons. The authors state in the rebuttal that Kruskal-Wallis tests were applied to all comparisons in Fig. 3c, and the corresponding code appears to use Kruskal-Wallis tests. However, the revised Fig. 3 legend still states that “P values are derived from one-way ANOVA with Dunnett multiple comparison tests.” The legend should be corrected to match the revised statistical analysis

2. Data availability and code reproducibility remain only partially resolved

The README and repository have been improved, including dataset descriptions, accession information, package versions, and script descriptions. However, the workflow is still not sufficiently clear for reproducibility.

First, the authors state in the response letter and README that required datasets are available through Figshare. However, we were unable to download the data from the provided Figshare private link, which prevented us from verifying whether the required files are complete and sufficient for reproducing the analyses. The authors should ensure that the final public Figshare link made available upon acceptance is fully accessible and downloadable, and that each Figshare data folder is clearly mapped to the scripts that use it.

Second, several scripts still rely on hard-coded local paths (e.g., /Volumes/..., /Users/..., or ~/Desktop/...), and many input files are read from local working directories. We recommend organizing the repository as a project-based structure with a data

directory downloaded from Figshare, and revising scripts to use relative paths rather than the hard-coded local paths.

(Remarks on code availability)
Please see my comments above.

Reviewer #7

(Remarks to the Author)
I co-reviewed this manuscript with one of the reviewers who provided the listed reports. This is part of the Nature Communications initiative to facilitate training in peer review and to provide appropriate recognition for Early Career Researchers who co-review manuscripts.

(Remarks on code availability)

Open Access This Peer Review File is licensed under a Creative Commons Attribution 4.0 International License, which permits use, sharing, adaptation, distribution and reproduction in any medium or format, as long as you give appropriate credit to the original author(s) and the source, provide a link to the Creative Commons license, and indicate if changes were made.
In cases where reviewers are anonymous, credit should be given to 'Anonymous Referee' and the source.
The images or other third party material in this Peer Review File are included in the article's Creative Commons license, unless indicated otherwise in a credit line to the material. If material is not included in the article's Creative Commons license and your intended use is not permitted by statutory regulation or exceeds the permitted use, you will need to obtain permission directly from the copyright holder.
To view a copy of this license, visit https://creativecommons.org/licenses/by/4.0/

Point-by-point response letter for manuscript NCOMMS-25-62380-T

"DNA methylation profiling identifies long-range epigenetic silencing of clustered protocadherins as a key determinant of meningioma progression"

General remarks: We want to thank the editorial team and all reviewers for their effort and suggestions to improve our manuscript. We have addressed all reviewer's comments, added new data and re-evaluated the text as outlined below. For enhanced readability, whenever feasible, we show figure panels that have been newly generated or adjusted in response the reviewer's comments within this letter at the adequate position. Please note that we have adjusted our bioinformatic pre-processing pipeline for DNA methylation data in response to the comments here, which has led to changes in our underlying dataset. These changes have been incorporated in all downstream analyses of our study and have thus led to minor modifications on the majority of figure panels throughout the manuscript (e.g. slightly different methylation cluster assignment), but are not necessarily shown here. We want to emphasize that all major claims from our initial submission still hold true.

Reviewer #1, expertise in brain tumour omics, meningioma, DNA methylation and precision medicine (Remarks to the Author):

The authors present a coherent and clinically relevant risk axis for meningioma based on a DNA hypermethylation program centered on clustered protocadherins. The simple two-state label (METHlow / METHhigh) may clarify prognosis across existing taxonomies. To achieve practice-changing impact, the authors may illustrate direct consequences for treatment decisions, and align explicitly with the recent cIMPACT-NOW update.

Response: We thank this reviewer for their acknowledgement of the clinical relevance of our findings.

Major comments

- The authors showed that METH clusters cut across published groups and improve prognostication, with particular value in intermediate-risk meningiomas. Please indicate when testing is recommended.

Response: We acknowledge the superiority of molecular features over morphological criteria alone in predicting recurrence risk in meningiomas as shown previously by several groups[1-3]. In line with these findings, our stratification based on methylation clustering was most apparent in improving risk estimation as compared to WHO grading, mostly in CNS WHO grade 2, but also rare cases of CNS WHO grade 1. However, even when compared to purely molecular-based or integrated classification systems, we find more accurate prognostication for intermediate-risk meningiomas. One of the prominent methods to recapitulate molecular-based groups are DNA methylation arrays, which also have the advantage to

provide CNV profiles. Additionally, to overcome shortcomings of grading based on morphological features alone, the cIMPACT-NOW update 8[4] now provides guidance for scenarios in which molecular testing is recommended:

- Morphological features borderline between CNS WHO grade 1 and 2
- BIOB meningioma
- CNS WHO grade 1 with unexpected rapid growth
- CNS WHO grade 2 confirmation
- clarification for chordoid, clear cell, rhabdoid, and papillary histology

We recommend testing for methylation clustering along the same guidelines. When DNA methylation arrays are employed, methylation groups, methylation clusters, as well as CNVs can be assigned. In this way, patients with tumours fitting above mentioned criteria will benefit from more accurate risk estimation according to molecular-based grading, with finer estimation for intermediate-cases according to methylation clustering. These recommendations are summarized at the end of the Discussion section on page 20 as follow:

"Within the framework of previous recommendations from the cIMPACT-NOW consortium[10], the methylation cluster classification should be seen as a complementary dichotomized molecular risk label providing additional prognostic resolution. Accordingly, we recommend extended molecular testing using DNA methylation arrays for meningiomas with unclear prognostication, including morphologically borderline CNS WHO grade 1 and 2, CNS WHO grade 2 confirmation, unexpected rapid growth of low-grade tumours, brain-invasive meningioma otherwise benign, and tumours with known high-risk histology."

- Please translate the METHhigh/METHlow label into a decision aid. The authors are encouraged to illustrate when and how METH status would alter management in terms of surveillance intervals, timing of adjuvant RT. etc.. A simple decision tree (inputs: EOR, 1p status, integrated risk score/methylation class/molecular subgroup, METH label: "observe", "discuss early RT", "adjuvant RT recommended") would help clinicians operationalize your findings.

Response: We thank this reviewer for this helpful and thoughtful suggestion. We have accordingly generated a decision tree diagram that summarizes recommendations for clinical management of meningioma patients according to CNS WHO grade, EOR, and extended molecular diagnostics including CNVs, methylation-based groups/clusters and integrated classification schemes. Note that methylation clustering might override management decisions derived from WHO grading or methylation-based grouping alone (CNS WHO grade 1/Benign-NF2-Wildtype/METH^{high} suggested for shorter surveillance intervals, while CNS WHO grade 2/Intermediate-Hypermetabolic/METH^{low} suggested for observation only). This decision diagram is shown in Supplementary Fig. 7 and is referred to in the Results section on page 8 as follows:

"Stratification according to methylation clusters seems to be of particular value for clinically-challenging meningiomas with intermediate risk profiles, and might thus have direct clinical implications for the management of meningioma patients (Supplementary Fig. 7)."

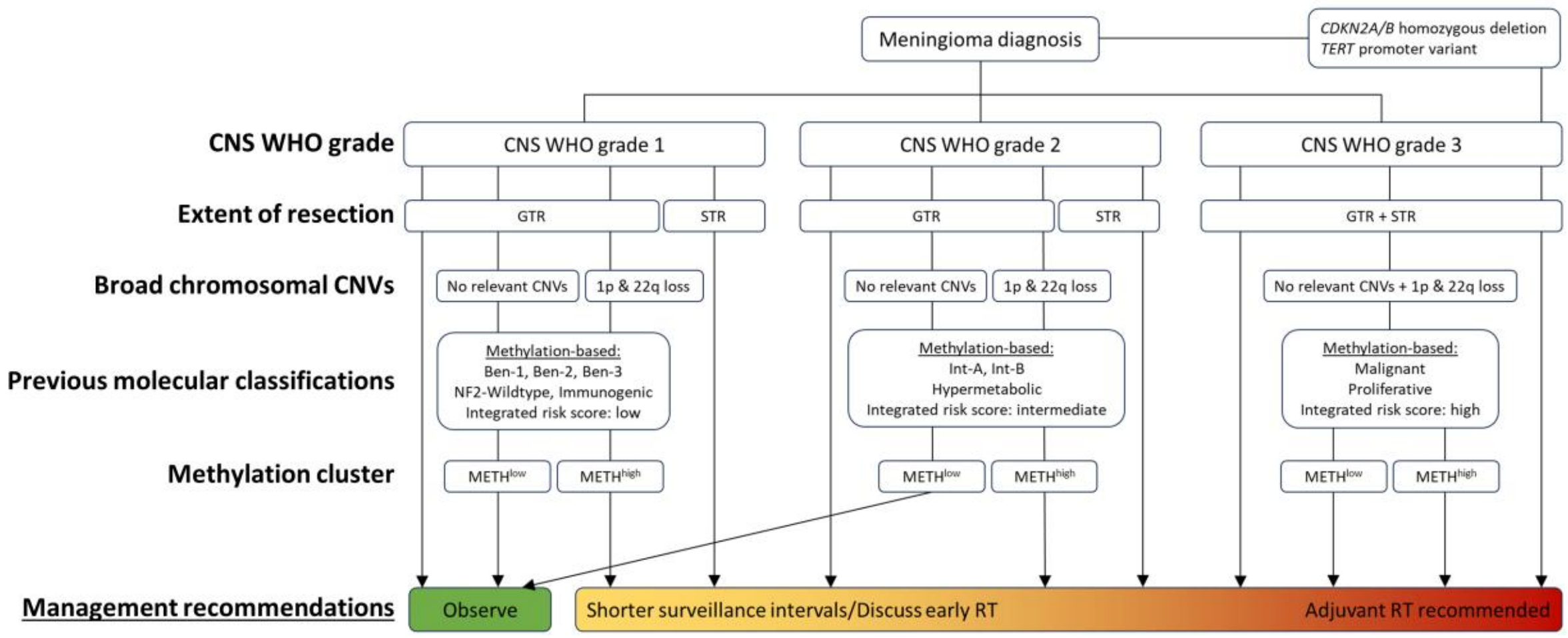

Supplementary Fig. 7: Decision tree for clinical follow-up management. *Recommendations for clinical management of meningioma patients according to CNS WHO grade, EOR, and extended molecular diagnostics. METH^{high} tumors otherwise classified as CNS WHO grade 1 should be subjected to shorter surveillance time frames, while METH^{low} tumors with CNS WHO grade 2 and no relevant CNVs might be recommended for observation only.*

The bioinformatic pipeline needed to derive methylation clustering from DNA methylation IDAT files is difficult to reproduce in a clinical setting. In order to allow for fast clinical real-world translation of our findings, we now provide an easy-to-use web application that predicts meningioma methylation clusters from raw IDAT files from both EPIC and EPICv2 arrays. Seeing the prognostic value of CNVs in meningioma, our web application also generates genome-wide copy number events to provide further prognostic information. We highlight this application in the Discussion section on page 20 as follows:

"In order to facilitate fast clinical translational in a real-world setting, we provide a ready-to-use web application that predicts both meningioma methylation cluster assignment and performs CNV analysis from Illumina DNA methylation arrays (https://mmcc.neurologie.uni-tuebingen.de)."

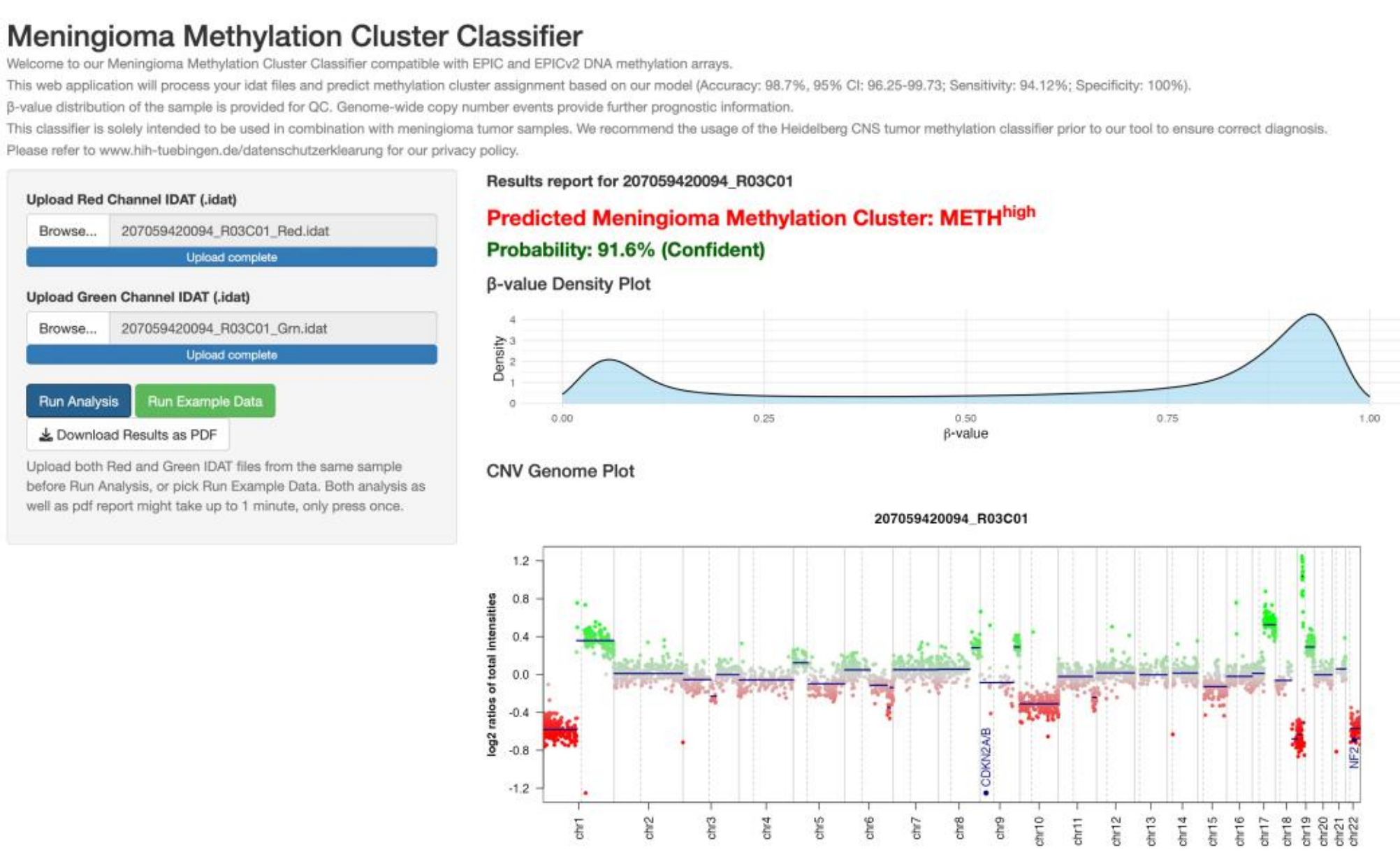

Example picture to illustrate the Meningioma Methylation Cluster Classifier available under https://mmcc.neurologie.uni-tuebingen.de.

- Please separate outcomes by pattern of failure (local bed vs. distant intracranial or spinal) and prior RT exposure. Methylation programs that predict local regrowth may not predict distant seeding, and prior RT can reshape both CNVs and genomic profiles. Provide additional information to contextualize molecular changes in recurrent meningiomas. Tie these dynamics to time-to-recurrence and interval therapies.

Response: We thank this reviewer for this insightful suggestion. We have now explicitly analysed clinical data of our cohort to provide an overview of patterns of failure. For 111 patients within the discovery cohort that experienced disease progression, the overwhelming majority (105/111, 94.6%) presented local regrowth within the surgical bed. Four patients (3.6%) demonstrated both local and distant intracranial recurrence at the time of progression, and only two patients (1.8%) had exclusive distant intracranial recurrence. Our cohort does not include any spinal recurrences. Given the small number of distant events, formal statistical comparisons by patterns of failure were not performed. However, we now provide these data as part of Supplementary Table 1 and within the Methods section on page 21 as follow:

"Across the Discovery cohort, 111 patients experienced disease progression during follow-up either restricted to the local surgical bed (94.6%), both local and intracranially distant (3.6%), or only intracranially distant (1.8%)."

We now provide full information on both prior and adjuvant radiotherapy in Supplementary Table 1, and have corrected a mistake in condition assignment for adjuvant RT present in the original manuscript. To identify a potential effect of prior RT, we have first stratified cases based on prior RT exposure and methylation cluster assignment and modeled PFS accordingly. In both $METH^{low}$ and $METH^{high}$ meningiomas, prior RT exposure was not significantly associated with PFS. Since all meningiomas that received prior RT were recurrences, i.e. a subset of tumours that inherently have a higher risk of disease progression, we also compared only recurrent meningiomas stratified by prior RT. These data further suggested that prior RT exposure does not affect PFS in our cohort. These data are now shown in Extended Data Fig. 4e, and have been mentioned in the Results section of the manuscript on page 8 as follows:

"Since radiotherapy (RT) is commonly applied to meningioma patients, we also assessed the effect of both prior RT and adjuvant RT on PFS in the context of methylation clusters. Prior RT was not associated with differences in PFS within either $METH^{low}$ or $METH^{high}$ tumours, including when analyses were restricted to recurrent cases only (Extended Data Fig. 4e)."

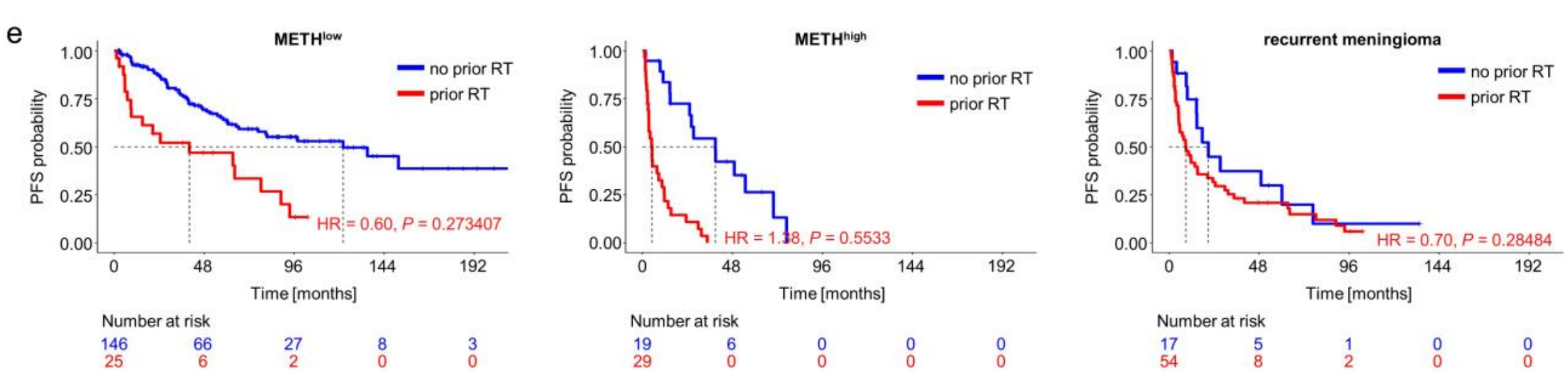

***Extended Data Fig. 4: Survival analyses within the Discovery cohort.** (...) **e**, Survival analyses for $METH^{low}$ and $METH^{high}$ meningiomas, stratified by whether patients received radiotherapy before the follow-up start or not. Since only recurrent cases had received prior RT, this analysis was also performed in recurrent-only cases (right).*

Additionally, we have analysed a potential effect of prior RT on CNVs in our cohort. We find that previously irradiated meningiomas present significantly higher levels of genome disruption and unfavourable CNVs across the entire Discovery cohort. However, since only recurrent cases had prior RT in our cohort, we next analysed only recurrent cases from our cohort. In fact, differences in CNV burden between irradiated and treatment naïve tumours were much smaller within recurrent meningiomas. Furthermore, since we found profound differences in CNV burden in distinct methylation clusters, we further stratified cases by cluster assignment, and find no significant effect anymore of prior RT on CNV burden in both methylation clusters. Together, these data are in line with our analyses described above on the effect of prior RT on PFS that accounted for these covariates. These new data are now shown in Extended Data Fig. 5b and have been incorporated in the Results section on page 9 as follows:

"When accounting for tumour setting and methylation cluster assignment, we did not observe a significant effect of prior radiotherapy on overall CNV burden (Extended Data Fig. 5b)."

b

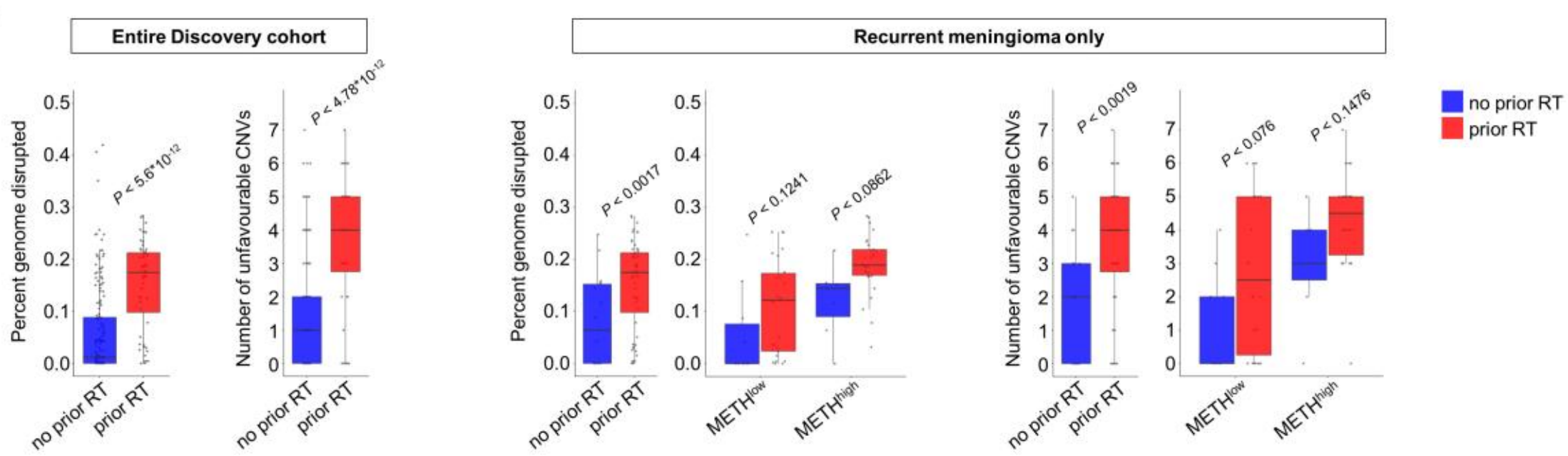

Extended Data Fig. 5: Validation of broad chromosomal CNVs in the Discovery and UCSF cohort. *(…)* ***b,*** *Effect of radiotherapy prior to the follow-up time on CNVs within the entire cohort (left) or only in recurrent meningiomas (right). Percentage of genome disrupted or number of unfavourable CNVs were used as metrics to define CNV burden. P values are derived from a Kruskal-Wallis test followed by Dunn's post hoc test (more than 2 groups) or Mann-Whitney test (2 groups).*

- TERT promoter mutations are a recognized high-risk marker in meningioma. Please report TERT status.

Response: We thank this reviewer for this comment and certainly recognize the effect of *TERT* promoter mutations on meningioma outcome and their recognition as independent diagnostic criteria for CNS WHO grade 3[5-7]. We have therefore performed custom NGS panel sequencing to analyze *TERT* hotspot mutations. While for some cases there was no genomic DNA available anymore, we have sequenced a total of 204 out of 231 tumour samples from the discovery cohort. These covered 98.8% and 94.6% of CNS WHO grade 1 and grade 2 cases, respectively, from our original submission in which *TERT* promoter mutations would ultimately change WHO grading. In total, we have detected *TERT* hotspot mutations in five samples, two in CNS WHO grade 1 (2x C228T), and three (1x C228T, 2x C250T) in CNS WHO grade 2 cases. All of these cases were correspondingly changed to CNS WHO grade 3. These changes have been implemented in all downstream analyses involving WHO grading and can now be seen in Supplementary Table 1. Information on our methodological approach can be found within the Methods section on page 27:

"To analyze TERT promoter hotspots (c.-124C>T (C228T), c.-146C>T (C250T)), targeted multigene mutation screening was performed by Next Generation Sequencing (Ion GeneStudio S5, Thermo Fisher Scientific,

Waltham, MA, USA) using an AmpliSeq Custom panel (hotspot regions in BRAF, CTNNB1, GNA11, GNAQ, KIT, KRAS, MAP2K1, NRAS, PDGFRA, and TERT (promoter region)). To analyze solitarily TERT hotspots, only pool 1 of the panel war used. Amplicon library preparation and semiconductor sequencing was done according to the manufacturers' manuals using the Ion AmpliSeq Library Kit v2.0, the Ion Library TaqMan Quantitation Kit on the QuantStudio 5 (Thermo Fisher Scientific), the Ion 540 Kit – Chef on the Ion Chef and the Ion 540 Chip Kit (Thermo Fisher Scientific). Output files were generated with Torrent Suite 5.18. Variants were visualized using the Integrative Genomics Viewer (IGV; Broad Institute, Cambridge, MA; Version 2.19.7) to detect TERT mutations."

- The data suggest METHhigh enriches for early progression. Please provide exploratory analyses of RT benefit by METH status and whether adjuvant RT preferentially benefits METHhigh.

Response: As adjuvant RT is currently the only adjunctive standard-of-care treatment aside from surgery for meningiomas not related to Neurofibromatosis, we appreciate this important point raised by the reviewer. When stratified based on methylation cluster assignment, adjuvant RT significantly prolonged PFS for METH^{low} meningiomas, while METH^{high} meningiomas only showed moderate improvement of PFS upon RT. However, when constructing Cox regression models with and without interaction term of cluster*adjuvant RT, likelihood ratio testing did not show any significant differences (χ2=0.1831, P=0.6687), indicating that the association between adjuvant RT and the hazard of the event did not differ between clusters. When cases from both methylation clusters were independently stratified based on EOR (GTR vs STR) and receipt of adjuvant RT (RT versus observation), we found significant improvement of PFS for METH^{low} meningiomas after STR (HR=0.25, P=0.00508), and moderate not statistically significant improvement for GTR (HR=0.31, P=0.138). For METH^{high} meningiomas, only marginal effects could be seen after STR (HR=0.38, P=0.115), while none of GTR cases received adjuvant RT. While these results have to be taken cautiously due to the small number of events in our cohort in particular for METH^{high} meningiomas that received adjuvant RT, our findings are largely in line with previous observations indicating that aggressive, Proliferative meningiomas are largely RT resistant[8]. Thus, our data suggest that METH^{low} meningiomas primarily benefit from adjuvant RT. These findings have been added to Extended Data Fig. 4f,g and are elaborated within the Results section on page 8 as follows:

"In contrast, adjuvant RT was associated with a significant PFS benefit in METH^{low} meningiomas, and this effect was mostly driven by tumours after subtotal resection (Extended Data Fig. 4f,g). This benefit appeared more profound than in METH^{high} tumours, but a likelihood ratio test for an interaction of adjuvant RT and methylation cluster did not support a clear sensitivity difference towards adjuvant RT. While these data have to be seen in the context of the limited cohort size, in particular for METH^{high} meningiomas, they are in line with previous reports demonstrating reduced sensitivity to adjuvant RT in biologically aggressive meningiomas[26]."

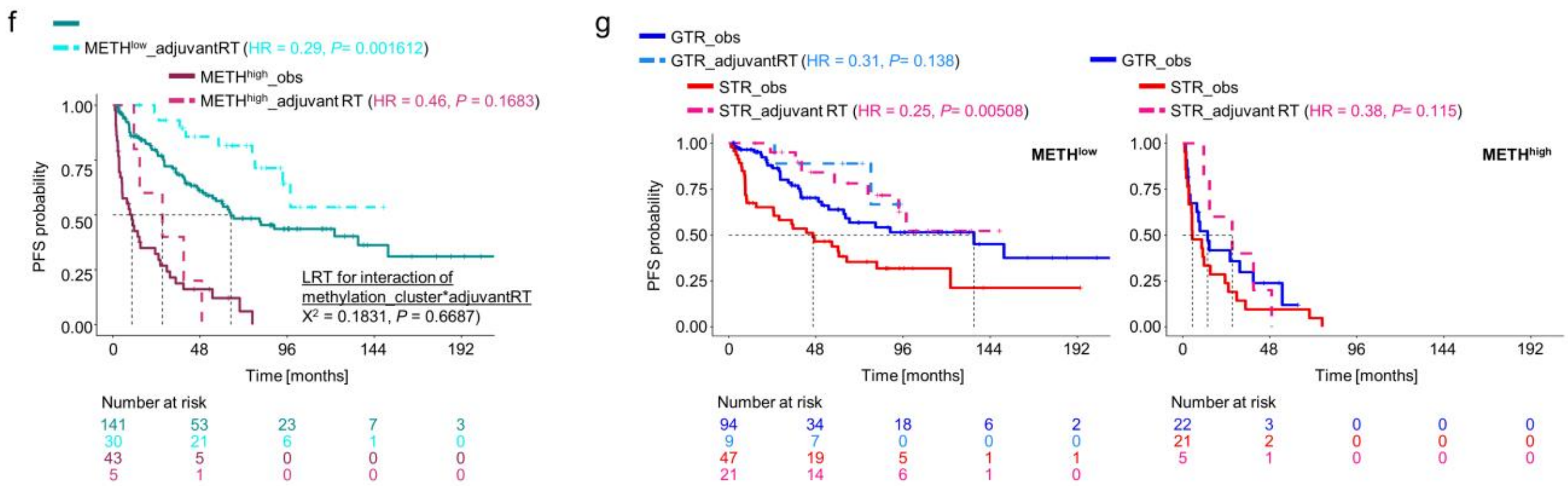

Extended Data Fig. 4: Survival analyses within the Discovery cohort. *(…)* ***f****, PFS outcomes within the Discovery cohort stratified by methylation cluster and adjuvant treatment (receipt of adjuvant RT). In this scenario, only METH^{low} meningiomas seem to significantly benefit from adjuvant RT, however, a likelihood ratio test for interaction of methylation cluster and adjuvant RT is not significant.* ***g****, PFS outcomes for either METH^{low} or METH^{high} meningiomas stratified by treatment modality (EOR and receipt of adjuvant RT).*

- Please contextualize the METH label within cIMPACT-NOW update 8 recommendations for meningioma grading and risk parameters.

Response: We want to emphasize that the METH label does not replace established grading criteria but augments cIMPACT-NOW-guided molecular risk stratification by identifying distinct subsets with differing recurrence risk. In this way, the METH label should be seen as a complementary, easily applicable molecular risk label. Specifically, the distinction between METH^{low} and METH^{high} tumours aligns with the cIMPACT-NOW-recommended scenarios for molecular testing, providing additional prognostic resolution. This comment essentially touches upon the same topic of this reviewer's comment above on recommendations for testing. We are therefore convinced that the following text within the Discussion section on page 20 provides a full presentation of our findings in the light of cIMPACT-NOW recommendations:

"Within the framework of previous recommendations from the cIMPACT-NOW consortium[12], the methylation cluster classification should be seen as a complementary dichotomized molecular risk label providing additional prognostic resolution. Accordingly, we recommend extended molecular testing using DNA methylation arrays for meningiomas with unclear prognostication, including histomorphologically borderline CNS WHO grade 1 and 2, CNS WHO grade 2 confirmation, unexpected rapid growth of low-grade tumours, brain-invasive meningioma otherwise benign, and tumours with known high-risk histology. While sparing patients at low risk of progression from adverse side effects of therapy, additional testing will also identify potentially underdiagnosed high-risk meningiomas which need closed-meshed surveillance time frames."

Minor comments

- Extended Figure 2D (hypermetabolic subgroup): Adapt the placement of its heading.

Response: We have corrected this mistake in what is now Extended Data Fig. 3a.

Reviewer #2, expertise in meningioma biomarkers, protocadherins and neuro-oncology (Remarks to the Author):

Review

The authors present a very interesting integrative study on meningioma progression, with a particular focus on global DNA methylation profiling. They provide original findings regarding the trajectory of meningioma progression, and the suppressive role of protocadherins, which are involved in the β-catenin/WNT signaling pathway. They also demonstrate the significant prognostic value of global methylation status in relevant subgroups, such as the intermediate subgroup and 1p-deleted meningiomas. In addition to methylome analyses, their approach highlights the prognostic relevance of β-catenin immunohistochemistry.

The results appear robust, being supported by a discovery cohort, a longitudinal cohort, and validation in a publicly available cohort. Moreover, the authors performed in-cell experiments to further investigate the role of protocadherins.

Response: We thank this reviewer for acknowledging the scientific rigor of our study.

Minor concerns

1- Was the proportion of METH^high cases higher in the proliferative group? Was the METH^low vs. METH^high signature significantly associated with survival in this proliferative group? This should be clarified in the Results.

Response: We thank this reviewer for this important point on the most aggressive molecular group of Proliferative meningiomas. In fact, 91% of all Proliferative meningiomas in our cohort were assigned to the $METH^{high}$ cluster, representing 82% of all tumours within that cluster. Data on the relative distribution of methylation clusters among previously described methylation groups can be seen from the stacked barplots in Fig. 1d. These plots display both the fraction within each methylation cluster represented by a given condition (y-axis, "What percentage of $METH^{low}$ or $METH^{high}$ clusters is associated with a given condition?"), as well as the partition of each classification condition to each methylation cluster in percent (percentages in boxes, "What percentage of a given condition is associated with each methylation cluster?"). In this sense, the previous figure legend for this panel was misleading, and we have now adjusted the text of the corresponding figure legend on page 36 as follow:

"Percentages on the y-axis show the fraction within each methylation cluster associated with a given condition, while percentages in boxes represent the partition of each condition to each methylation cluster."

In order to enhance and clarify readability of our study, we have added the following text sequence to the Results section on page 6 as follows:

"For example, while all NF2-Wildtype/Immunogenic meningiomas formed part of the $METH^{low}$ cluster (48% and 29% of that cluster, respectively), 91% of Proliferative meningiomas were associated with the $METH^{high}$ cluster (82% of that cluster)."

Within our Discovery cohort, we only have four Proliferative meningiomas that belong to the $METH^{low}$ cluster, and only three of those have accompanying follow-up data on PFS. Throughout the manuscript,

we refrained from analyzing PFS for conditions that have less than five subjects in one group. This being said, two out of those three meningiomas progressed during follow-up time only at around 64 months, a time when the vast majority of $METH^{high}$ Proliferative meningiomas in our cohort have already progressed (please see below for visual representation of that data that we did not include in the manuscript). While we are convinced that our cohort is too small to investigate the prognostic effect of variables that show a very strong shift in distribution among $METH^{low}$ and $METH^{high}$ cluster, such as Proliferative meningiomas, we certainly acknowledge that these are important questions for follow-up investigations of our findings in multi-centre studies which will add higher statistical power.

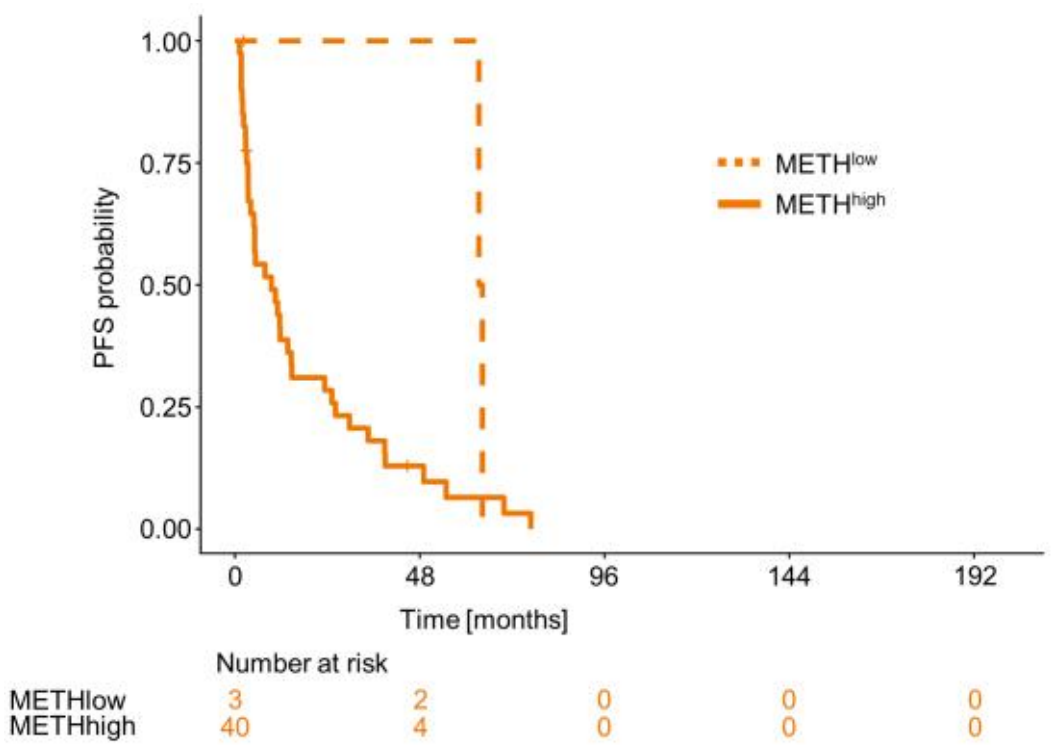

Graph showing PFS outcome of Proliferative meningiomas stratified by methylation cluster assignment.

2- Was there a correlation between the Ki-67 labeling index (a well-established prognostic marker) and the hypermethylation signature?

Response: This is an important point, and we have followed this reviewer's advice to analyze a potential correlation of the hypermethylation signature and the proliferative activity of corresponding tumours (as measured by Ki67 immunoreactivity). In fact, we found a moderate correlation (r=0.51), further supporting our findings of the association between clinical behaviour and our DNA hypermethylation signature. This finding is now shown in Extended Data Fig. 2f, and has been added to the results section on page 6 a follows:

"In line with this, we found a moderate correlation of the average methylation of cluster probes and the Ki67-positive cell fraction in meningioma tissue (Extended Data Fig. 2f)."

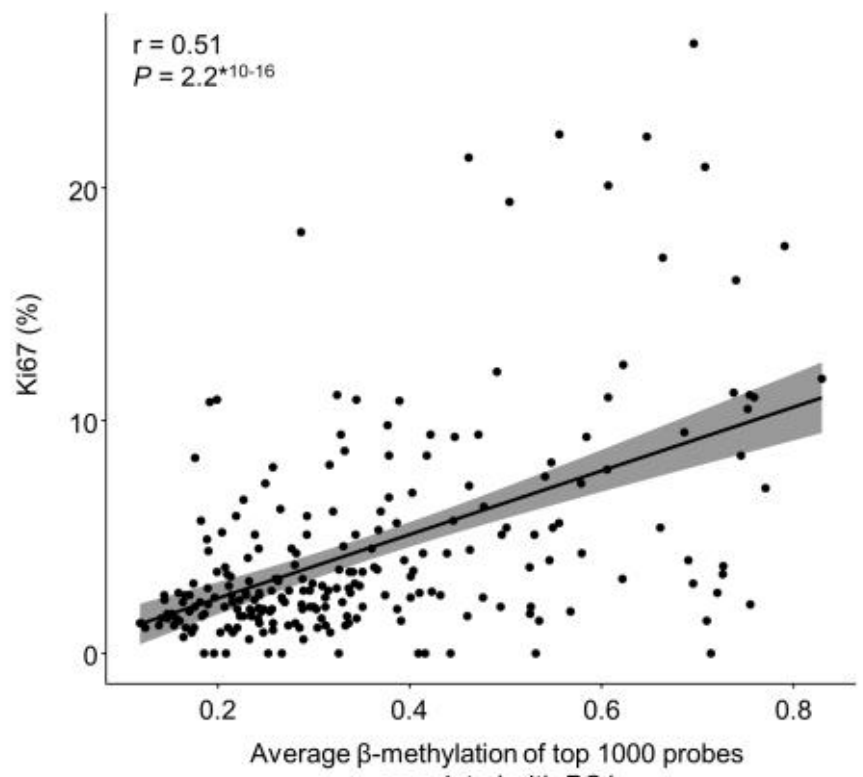

Extended Data Fig. 2: Cluster consistency for hypermethylation signature separating benign from aggressive meningiomas. *(…)* ***f,*** *Correlation of methylation cluster signature and fraction of Ki67-positive cells in tumour tissue. Correlation coefficient r and a regression line with 95% CI are shown.*

3- A few words about the prognostic value of β-catenin (IHC) should be added to the Abstract.

Response: We followed this reviewer's advice and added the following sentence in the Abstract on page 1:

"Systematic analysis of DNA hypermethylation in high-grade meningiomas unravels a tumour suppressive role of clustered protocadherins by restricting β-catenin nuclear localisation, consistent with the association between nuclear β-catenin staining and meningioma progression."

4- In the Introduction, first sentence: should this refer to arachnoid cap cells or to the subdural inner layer (see studies from Kalamarides et al.)?

Response: This reviewer raises an important point, and we are sorry for this inaccuracy in our primary submission. The cell of origin of meningioma is commonly reported to be the arachnoid cap cells. However, this view has been challenged by the discovery that prostaglandin D2 synthase-positive meningeal precursor cells in mice are susceptible to Nf2 inactivation and might thus be the cell of origin for meningioma[9]. We have adjusted the first sentence within the Introduction to reflect these findings and state the appropriate literature on page 2 of the manuscript:

"Meningiomas might arise from prostaglandin D2 synthase-positive meningeal precursors cells and account for approximately 40% of all primary intracranial tumours[1,2]."

5- Fig 6i, magnification of IHC picture should be higher (focusing on nuclear staining, which is not evident on the illustration).

Response: We have adjusted the pictures in what is now Fig. 7i, showing both low and high magnification for meningiomas at corresponding quartiles of β-catenin nuclear immunoreactivity. Please note that due to our new bioinformatic pipeline and the resulting new methylation cluster assignment during revision, these are not the same tumour samples as in the initial submission.

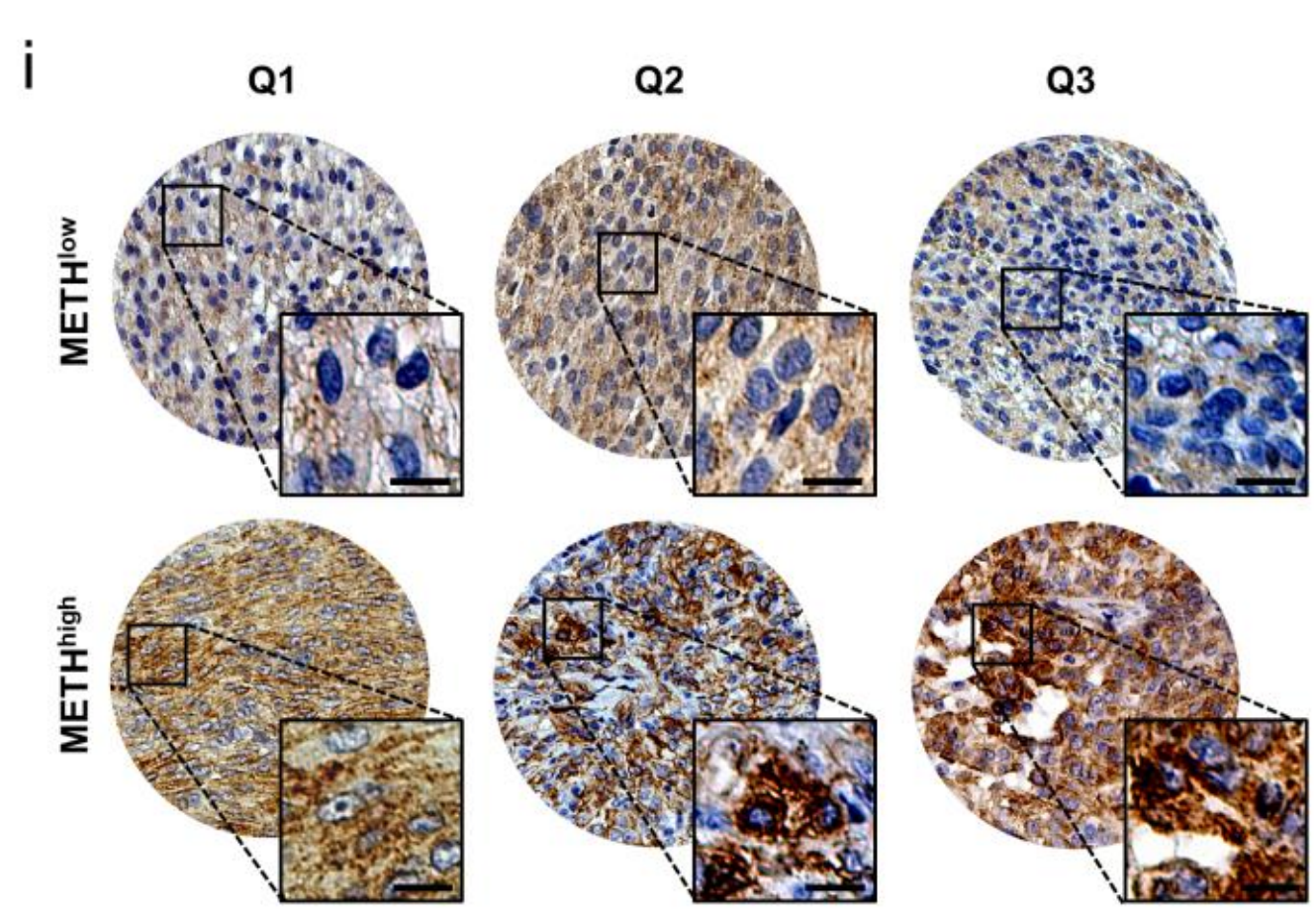

***Fig. 7**: **Tumour-suppressive functions of protocadherins associate with modulation of sub-cellular localization of β-catenin.** (…) **i,** Immunohistochemistry was performed on 80 METH^{low} and 49 METH^{high} human meningiomas on tissue microarrays (TMAs). Exemplary pictures show cases at the 25th, 50th, and 75th percentile with respect to β-catenin immunoreactivity ranking within each cluster. Scale bars: 25 µm.*

Style

In the Results section, the use of present vs. past tense should be standardized.

Response: We now ensure that past tense is used throughout the entire Results section.

Reviewer #3, expertise in protocadherins and brain tumours (Remarks to the Author):

The manuscript with a title „DNA methylation profiling identifies long-range epigenetic silencing of clustered protocadherins as a key determinant of meningioma progression" provides data on DNA methylation profiles from 231 meningioma samples. It identifies clustered protocadherins as determinants of meningioma progression. Although the role of clustered protocadherins in the progression of many different cancers have been demonstrated and their methylation has been demonstrated in meningiomas before, the current study adds additional value due to its complexity and the large number of clinical samples analysed. This research could thus contribute to the establishment of new biomarkers for the classification of meningiomas, the guidance of therapy and the development of new treatment options for affected patients. Significant differences in methylation patterns between normal meninges and those observed during meningioma development are convincingly demonstrated and evaluated using various methods. In addition to the methylation patterns and their comprehensive analysis, the authors present gene expression data from cancers with high and low methylation and demonstrate a correlation between these two data sets. They provide valuable experimental data supporting the overall hypothesis. The overexpressing one of the clustered protocadherins, PCDHGC3, in the IOMM-Lee cell line confirmed its strong effects on the regulation of Wnt-signaling. They provide evidence that epigenic silencing and the PCDHGC3-mediated regulation of b-catenin activity, known from the literature, contribute to poor clinical outcomes in meningioma patients.

Response: We thank this reviewer for acknowledging the additional value our findings provide for the role of protocadherins in meningiomas.

I have just a few comments:

It would be interesting to describe in the introduction the current classification schemes used clinically for meningiomas and to describe the potential of the presented data for defining new clinical classification markers either in the introduction or in the discussion

Response: We agree with this reviewer that a short introduction to currently applied classification schemes will help the reader understand the progress presented in this paper better, and have thus adjusted the Introduction section on page 2 as follows:

"In current clinical practice, meningiomas are primarily classified based on histopathological features assigned to one of three WHO grades (grades 1-3) which are intended to reflect biological aggressiveness and risk of recurrence. The 2021 World Health Organization (WHO) Classification of Tumours of the Central Nervous System integrates histological subtype, other histological atypia features such as brain invasion, mitotic activity or necrosis, and selected molecular alterations into this grading framework[6]. CNS Who grade 1 meningiomas are considered benign, grade 2 tumours are classified as atypical, and grade 3 tumours as anaplastic or malignant. Importantly, recent updates to the WHO classification incorporate molecular criteria such as homozygous deletion of CDKN2A/B or oncogenic TERT promoter mutations, which can independently define CNS WHO grade 3 disease irrespective of histological appearance."

Additionally, also in response to Reviewer #1, we now provide a clear integration of our findings in the light of current knowledge of molecular meningiomas groups and their relation to risk assessment within the Discussion section of our paper on page 20 as follows:

“Within the framework of previous recommendations from the cIMPACT-NOW consortium[12], the methylation cluster classification should be seen as a complementary dichotomized molecular risk label providing additional prognostic resolution. Accordingly, we recommend extended molecular testing using DNA methylation arrays for meningiomas with unclear prognostication, including histomorphologically borderline CNS WHO grade 1 and 2, CNS WHO grade 2 confirmation, unexpected rapid growth of low-grade tumours, brain-invasive meningioma otherwise benign, and tumours with known high-risk histology. While sparing patients at low risk of progression from adverse side effects of therapy, additional testing will also identify potentially underdiagnosed high-risk meningiomas which need closed-meshed surveillance time frames.”

Additional analysis of PCDHGC3 and / or other clustered protocadherins levels in published meningioma mRNA sequencing datasets and their correlation with clinical parameters and overall survival would contribute to the establishment of PCDH genes as novel biomarkers in meningiomas.

Response: This is indeed an important point and we want to thank this reviewer for their comment. In order to respond to this comment, we made use of the UCSF cohort[3] again. While there is no additional clinical parameters or survival data available there, we were able use the publicly available gene expression data from that study (n = 185) for which we knew the methylation cluster assignment derived from global DNA methylation profiles (n = 162 $METH^{low}$ and n = 23 $METH^{high}$ meningiomas). When analyzing this cohort for gene expression changes associated with methylation cluster, we found various genes to be differentially expressed. Many of those were from the HOX gene clusters. Furthermore, many of the HOX genes showed upregulation in $METH^{high}$ tumors, again supporting our previous data that hypermethylation is not necessarily associated with downregulation of gene expression. Of note, this analysis also showed the homeobox transcription factor *PITX1* as one of the most prominently upregulated genes in $METH^{high}$ tumors, thus supporting RNAseq data from our samples shown in the previous submission.

When further focusing on genes from protocadherin clusters, we found only a small number to be differentially expressed at a statistically significant level. However, one of the genes significantly downregulated in $METH^{high}$ meningiomas was *PCDHGC3* that we had previously found in our RNAseq as well, and which we used for further phenotypical analysis in human meningioma cell lines (Fig. 7). As for individual protocadherin clusters, we also provide correlation analyses for all members, illustrating again that changes in gene expression are restricted to a smaller number of genes, and these might be either up- or downregulated. Since our analysis for the UCSF cohort is substantially bigger than our own RNAseq data in terms of sample size, we now opted to show our RNAseq data as a preliminary investigation in Extended Data 8d, and show the results from the UCSF cohort in Fig. 6g, Extended Data Fig. 8d and Supplementary Table 8.

These new findings have been added to the Results section on pages 13 and 14 as follows:

“Thus, to further investigate the relationship of gene cluster methylation and expression of corresponding genes, we first carried out RNA sequencing of a small cohort of $METH^{high}$ and $METH^{low}$ meningiomas (n = 10; Supplementary Table 7). While the majority of hypermethylated genes did not show gene expression changes between $METH^{high}$ and $METH^{low}$ meningiomas (Extended Data Fig. 8d), we found distinct genes with a hypermethylation phenotype to be differentially expressed, including the homeobox transcription factor PITX1 and several protocadherins such as PCDHGC3. Validating these initial findings in meningioma samples from the larger UCSF cohort with available expression and DNA methylation data (n = 185), we

found a preferential upregulation of homeobox transcription factors including several HOX genes and PITX1, as well as robust downregulation of protocadherins such as PCDHB12 and PCDHGC3 in METH^{high} (n = 23) as compared to METH^{low} meningiomas (n = 162) (Fig. 6g, Extended Data Fig. 8e and Supplementary Table 8)."

Additionally, this is covered in the Methods section on page 28 as follows:

"For validation of gene expression findings, meningioma samples from the UCSF cohort[20] with available gene expression data and methylation data to predict methylation cluster assignment (n = 185) were used and analysed using DESeq2."

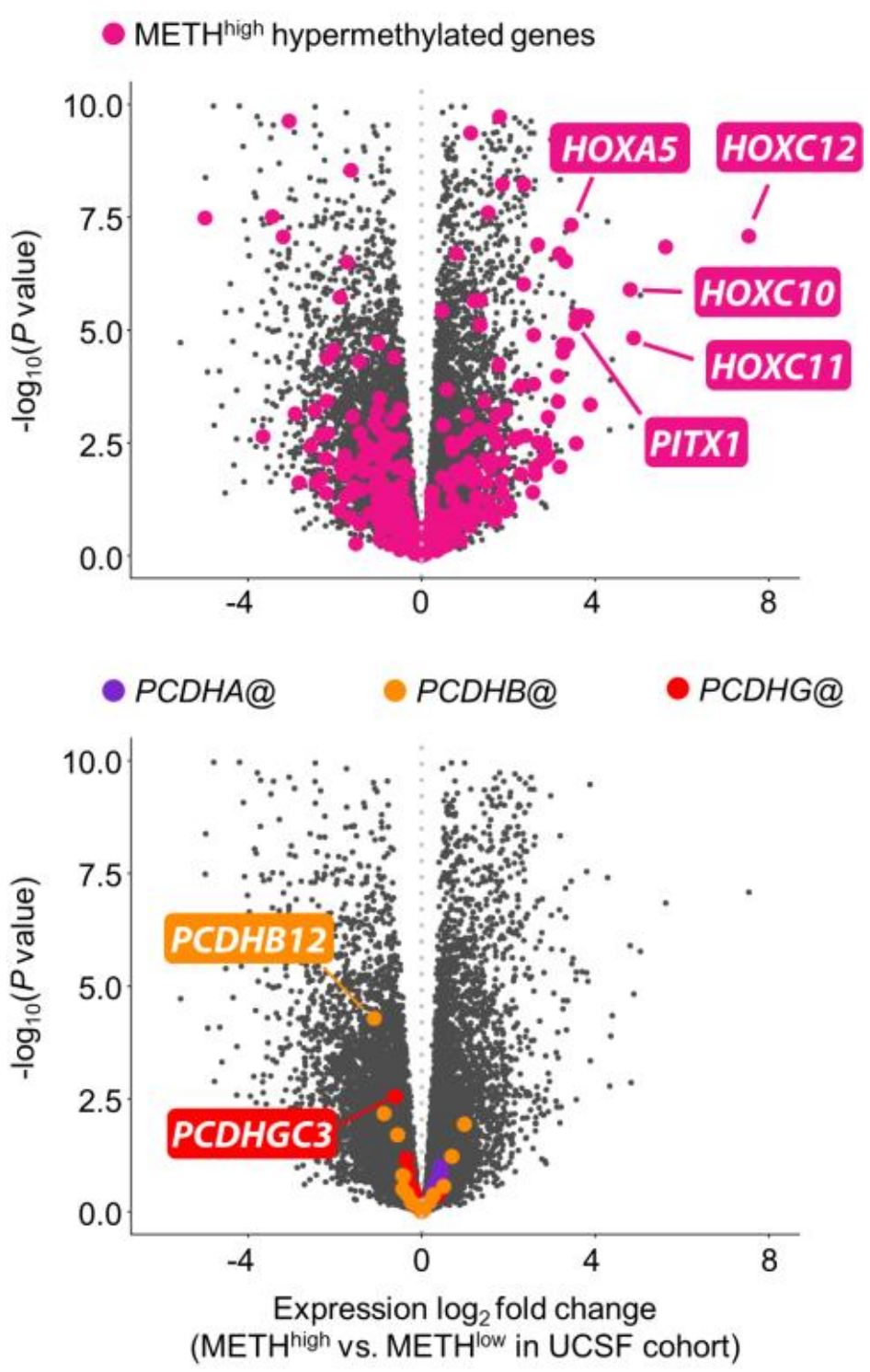

***Fig. 6**: **Meningioma progression is associated with long-range epigenetic silencing of protocadherin genes.** (…) **g**, Volcano plots illustrating the log$_2$ fold changes in gene expression between METH^{high} (n = 23) versus METH^{low} (n = 162) tumour samples in the UCSF cohort. Coloured genes have been found to be hypermethylated in METH^{high} samples from the Discovery cohort (top), or denote clustered protocadherins (bottom).*

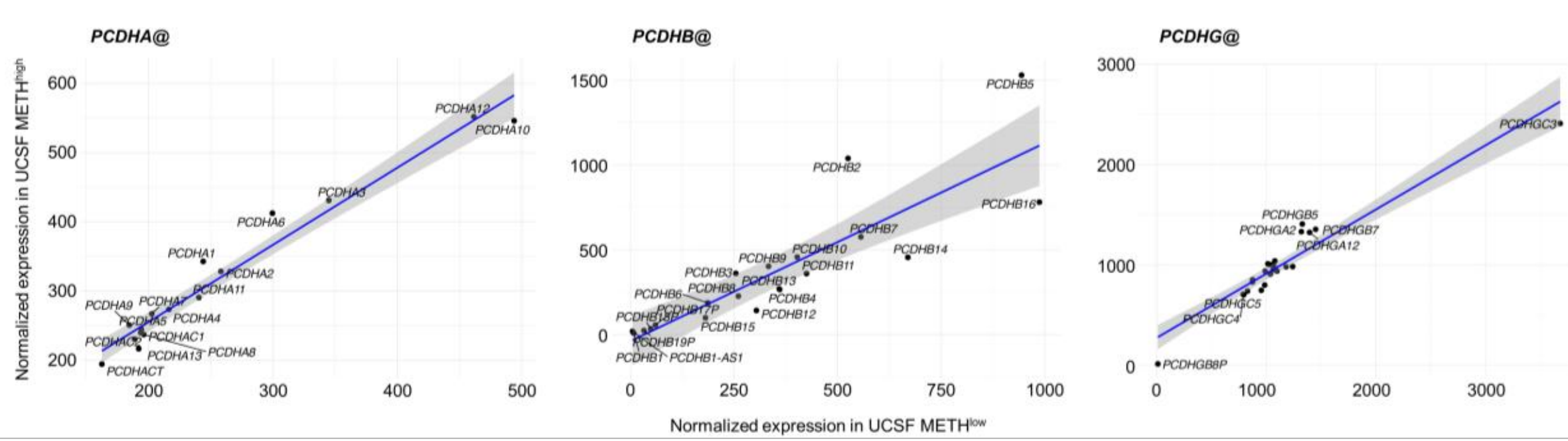

***Extended Data Fig. 8**: **Genes predicted to be affected by differential DNA methylation in METH^{high} meningiomas and their validation in gene expression data.** (…) **e**, Correlation of normalized gene expression values for individual protocadherin clusters in METH^{low} and METH^{high} meningiomas from the UCSF cohort. A regression line and the corresponding 95% confidence interval are shown.*

Additionally, in order to strengthen our previous data on phenotypical changes of protocadherin overexpression on β-catenin localization, we also performed *PCDHGC3* overexpression in a benign meningioma cell line (BEN-MEN-1), and contrasted these results with our previous findings from the malignant IOMM-Lee cell lines. Of note, in BEN-MEN-1 cells, which show preferential cytoplasmic β-catenin localization as opposed to nuclear localization in IOMM-Lee cells on baseline (Fig. 7b,c), overexpression of *PCDHGC3* did not shift β-catenin expression towards a more cytoplasmic localization, a feature that was apparent for IOMM-Lee cells. These new data are now shown in Extended Data Fig. 10, and are mentioned within the Results section on page 15 as follows:

"In contrast, we did not detect any changes in the nuclear-to-cytoplasmic ratio of β-catenin when overexpressing PCDHGC3 in the benign cell line BEN-MEN-1 (Extended Data Fig. 10; Mann Whitney test, P = 0.9976)."

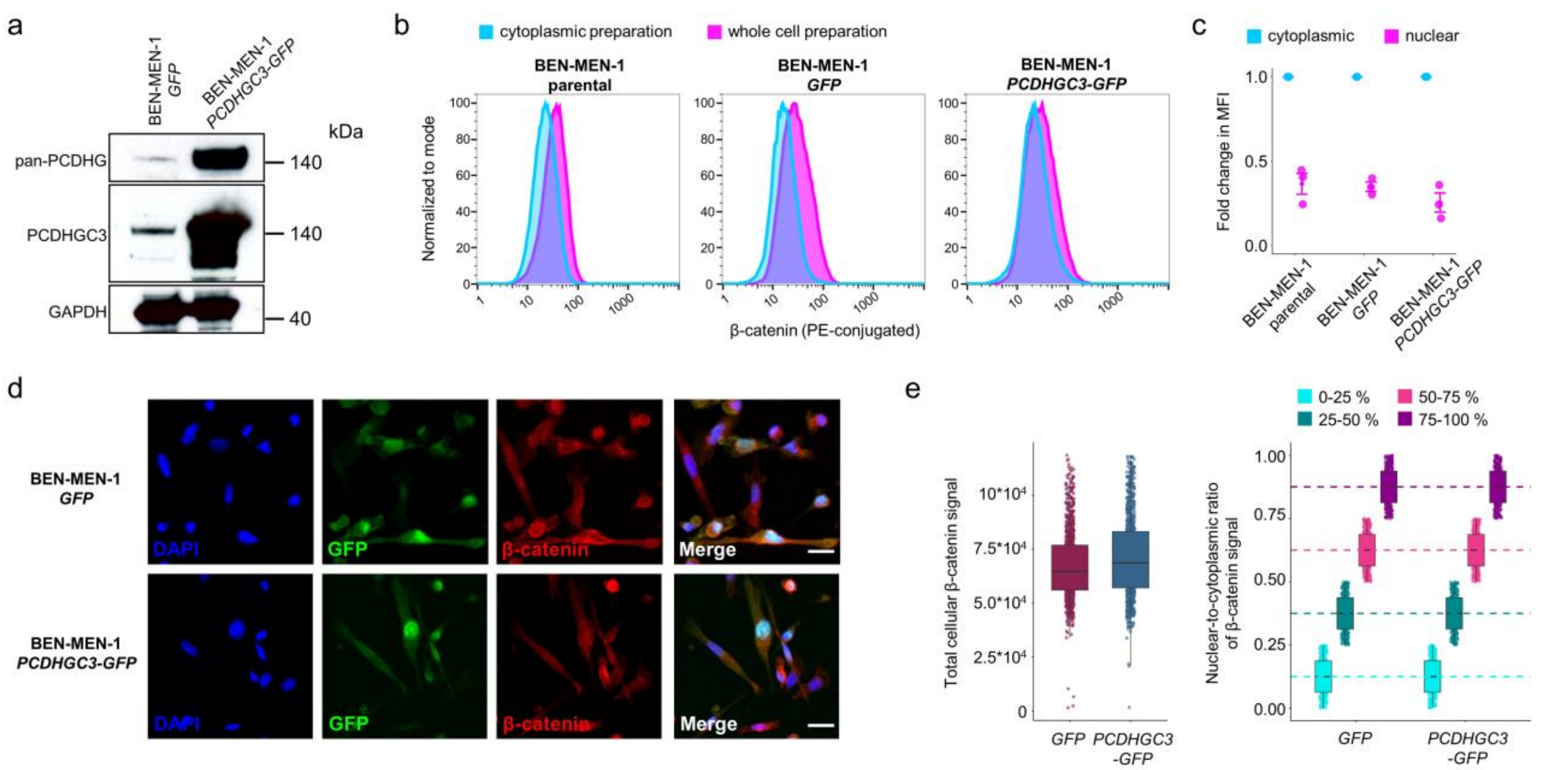

Extended Data Fig. 10: Effect of PCDHGC3 overexpression on β-catenin localization in BEN-MNE-1 cells. ***a***, *Western blot analysis validating overexpression of PCDHGC3 in BEN-MEN-1 meningioma cells.* ***b***, *Normalized density distributions of β-catenin signal from cytoplasmic or whole cell preparations of parental, control, and PCDHGC3-GFP-expressing BEN-MEN-1 cells.* ***c***, *Quantification of cytoplasmic and nuclear β-catenin signal in parental, control, and PCDHGC3-GFP-expressing BEN-MEN-1 cells. Error bars represent mean ± SE.* ***d***, *Representative images from confocal microscopy of BEN-MEN-1 cells (GFP or PCDHGC3-GFP) illustrating expression of GFP and PCDHGC3 together with DAPI stain. Scale bars: 25 µm.* ***e***, *Quantification of total levels of β-catenin in control and PCDHGC3-overexpressing BEN-MEN-1 cells (left). Comparison of the nuclear-to-cytoplasmic ratio of β-catenin signal binned by quartile (right).*

Reviewer #4, expertise in biostatistics and epigenomics (Remarks to the Author):

Key results and significance

This paper presents high-methylation and low-methylation subgroups for meningioma that help to stratify the mid-risk class from currently available classifiers. It proceeds to seek biological function underlying these subgroups that may relate to the differential outcome. While further understanding the prognosis of these challenging cases is of value, there is sufficient concern with the analytical methods to reduce my confidence in the presented results.

Data, methodology, analytical approach, and validity

1. Technical factors that may impact the analysis outcomes are not addressed.

Response: We want to thank this reviewer for stressing the importance of addressing potential technical factors that might affect data analysis. We have investigated the impact of several technical factors now in more detail as outlined below.

a. Explain how the tumour content was verified in the FFPE tissue samples before DNA and RNA extraction (lines 476-7, 552).

Response: For all samples analysed in our study, tumour content was estimated histologically in H&E stains by an experienced neuropathologist in increments of 10% (e.g. 70%, 80%, 90%). We have now added the estimation of tumour content in FFPE tissue samples to Supplementary Table 1. Overall, as expected for a well-circumscribed, non-infiltrative tumour such as meningioma, we have seen rather high tumour content in tissue samples. This has been specified within the Methods section on page 21 as follows:

"Analysis of tissue histology including estimation of tumour content as well as grading based on morphological features has been performed by an experienced neuropathologist."

b. Examine the extent to which PCA is impacted by technical artifacts – % tumour content in tissue sample, batch or run-order, age of the FFPE block.

Response: We appreciate this important point, and want to apologize for not accounting for potential confounding technical artifacts in the initial submission. We now include a detailed analysis of technical artifacts in our discovery cohort, which indeed has revealed several technical batch effects. To systemically evaluate whether technical factors influenced global DNA methylation patterns, we explicitly assessed multiple potential sources of unwanted variation, as suggested by this reviewer, including (i) time of DNA isolation, (ii) array run/batch on the Illumina methylation platform, (iii) tumour cell content on tissue sections, and (iv) age of FFPE block.

First, we quantified their contribution to variation in the dataset using PERMDISP (to assess differences in dispersion) followed by PERMANOVA (to test for differences in centroids). These analyses indicated that DNA isolation time, array batch, and tissue age were associated with statistically significant batch effects, whereas tumour content was not.

To correct for these technical confounders, we applied the limma removeBatchEffect function, incorporating the identified variables with significant effects. Following batch correction, we reassessed the data structure using PERMANOVA. As expected, and consistent with effective removal of technical variation, none of the previously detected batch effects remained statistically significant after correction. This confirms that the adjustment procedure successfully mitigated the influence of technical artifacts while preserving the biological structure of the dataset (as can be seen by downstream analysis of the data). This being said, it should be noted that the batch effect correction performed here, together with the harmonization of preprocessing steps for our discovery cohort throughout the manuscript described further below, slightly changed the selection of probes and their β values for all downstream analyses.

We provide the full code used for batch correction which can be found in the section "01_QC_preselect" on Anonymous Github. Furthermore, we have updated the Methods section on page 23 as follows:

"We considered time of genomic DNA isolation, EPICv2 array runs, tumour content on tissue sections and age of the paraffin block as potentially confounding technical artifacts. These potential technical confounders were evaluated using PERMANOVA on multivariate methylation distances, with PERMDISP used to assess homogeneity of dispersion. All factors identified to represent a real confounding factor were corrected using the removeBatchEffect function from the limma package in R. "

The Results section as been updated on page 5 as follows:

"The initial dataset was analysed for technical batch effects and corrected accordingly using linear modelling (Supplementary Fig. 2)."

The main results and validation of batch effect correction are now shown in Supplementary Figure 2.

a

PERMANOVA results before correction

Factor	Df	SumOfSqs	R^2	F	P value
DNA isolation	19	12,250,436	0.26891	4.0848	0.001
Array run	34	17,268,996	0.37908	3.5194	0.001
Tumor content	8	1,390,016	0.03051	0.8734	0.612
FFPE age	1	4,164,800	0.09142	23.043	0.001

b

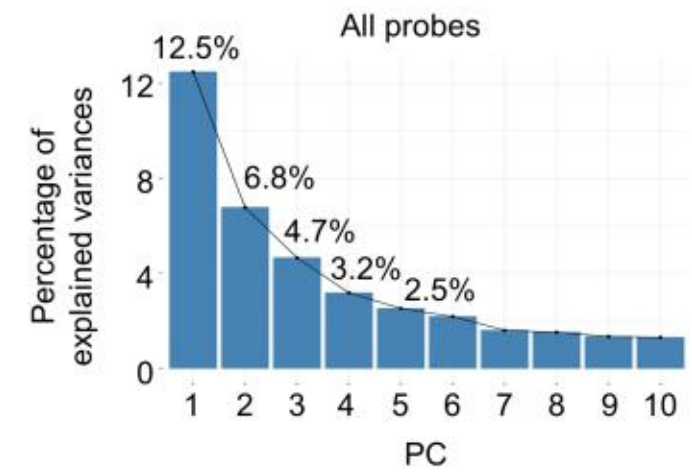

c

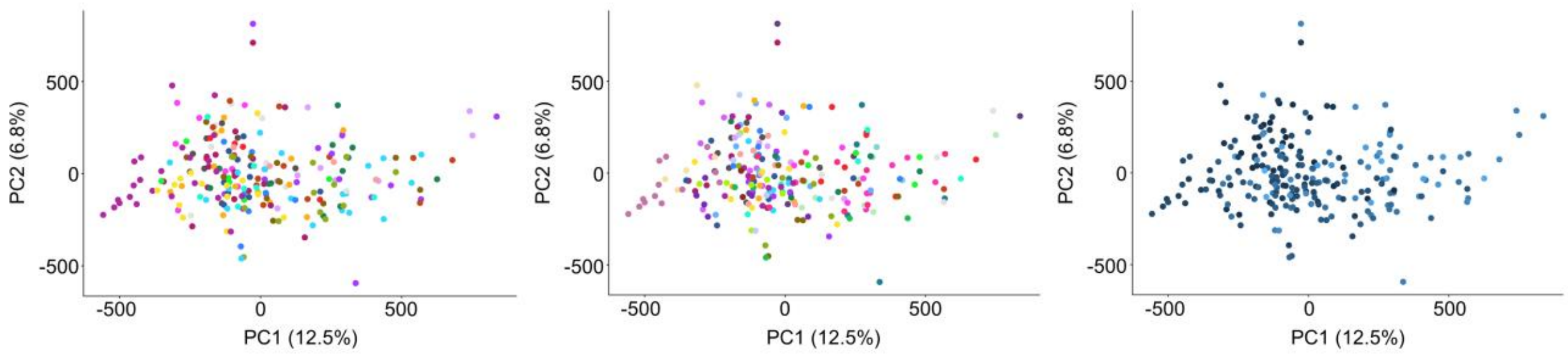

d

PERMANOVA results after limma::removeBatchEffect

Factor	Df	SumOfSqs	R^2	F	P value
DNA isolation	19	2,077,732	0.05	0.5845	1
Array run	34	2,795,265	0.06727	0.4158	1
Tumor content	8	1,673,111	0.04026	1.1642	0.192
FFPE age	1	76,520	0.00184	0.4225	0.896

e

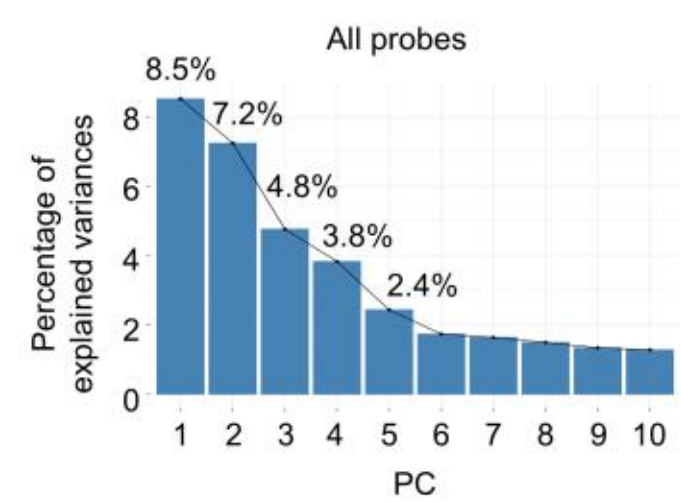

f

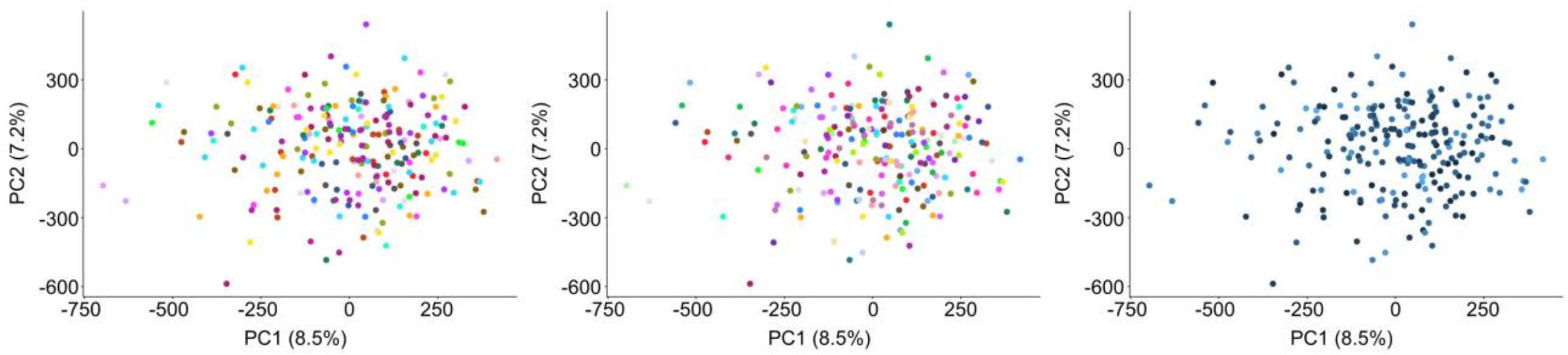

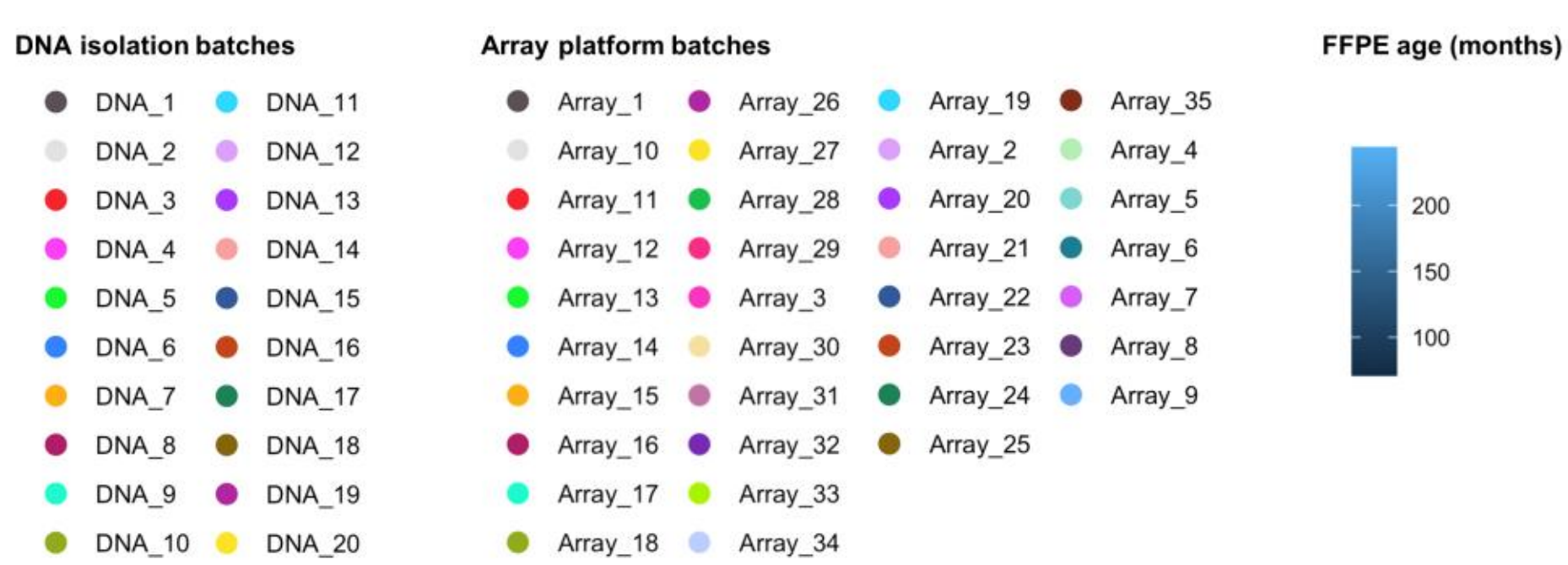

***Supplementary Fig. 2: Batch effect correction in the Discovery cohort**. **a-c**, Results from permutational multivariate analysis of variance on all data from the Discovery cohort (**a**). Scree plot illustrating results from principal component analysis on all data before correction (**b**). Scatter plots associating principal component 1 and 2 in terms of individual tumor sample position, color-coding by DNA isolation batches,*

*array platform batches, and age of the FFPE material (**c**). (**d-f**) Same data illustrations as in a-c, but after correction using the limma::removeBatchEffect function.*

2. Survival modeling has temporality issues that may impact conclusions

Response: We have re-analysed our survival data as outlined below.

a. Define the PFS outcome for primary and for recurrent cases.

Response: Within our study, the definition of PFS is identical for both primary and recurrent cases, i.e. the period between surgical resection and the time of progression as verified by MRI imaging. Patients without progression were censored at last follow-up. In this way, PFS reflects the same clinical processes in both primary and recurrent tumours. We have specified our definition of time zero and endpoint for PFS within the Methods section on page 21 as follows:

"Progression-free survival (PFS) was defined as the period between surgical resection and the time of progression (date of MRI indicating progression). The same definition of PFS was applied to both primary and recurrent tumours. Patients without progression were censored at last follow-up."

b. Primary and recurrent cases likely need to be modeled separately. Simple covariate adjustment makes strong assumptions that primary and recurrent tumours have the same baseline hazard and association with predictors.

Response: We agree that primary and recurrent tumours may differ in baseline hazard. To address this concern, we refit Cox proportional hazards models stratified by tumour setting (primary vs recurrent), thereby allowing for distinct baseline hazard functions while estimating common covariate effects. This approach relaxes the assumption of a shared baseline hazard while keeping the statistical power of our entire cohort. The results were qualitatively similar and did not alter the conclusions. The entire code used for survival analyses can be found at Anonymous Github under section "06_survival", which will be released on regular Github upon acceptance of this manuscript. This is outlined in the Methods section of our manuscript on page 29 as follows:

"To account for potential differences in baseline hazard between primary and recurrent tumours, Cox proportional hazards models were stratified by tumour status (primary vs recurrent), allowing for separate baseline hazard functions while estimating shared covariate effects."

c. Adjuvant RT is not a baseline factor for primary diagnoses, and it is not clear if primary or post-recurrence adjuvant RT is modeled for recurrent diagnoses. To prevent immortal time bias, adjuvant RT must be included as a time-varying covariate. Caution should also be used since receipt of adjuvant RT is tied to grade and EOR which are in the model.

Response: We thank this reviewer for stressing this important point, and want to apologize for not modeling adjuvant RT correctly in the initial manuscript. Within our manuscript, adjuvant RT is defined as radiotherapy applied after initial surgical resection (i.e. time zero of our follow-up time). As such, adjuvant RT is in fact not a baseline factor, i.e. a covariate measured after follow-up time begins, and it is well know that Cox regression is biased in favor of responders in this scenario[10]. In order to prevent immortal time bias, we have now used the *tmerge* function of the *survival* package to generate a counting process format of our survival data that treats adjuvant RT as a time-varying covariate, taking into account the time between time zero and the start of RT. The full code can be found at section "06_survival" at Anonymous Github, which will be released on regular Github upon acceptance of this manuscript. This has been clarified in the Methods section of the manuscript on page 29 as follows:

"Adjuvant RT was treated as a time-varying covariate using a counting process format in order to prevent immortal time bias."

For enhanced readability of our manuscript, the majority of survival analyses are now shown independently in Fig. 2.

d. Caution is needed when reporting and visualizing time-to-event data, so that censoring time is addressed. Figure ED1d does not account for censoring time and cannot be interpreted.

Response: We certainly acknowledge that this panel from the initial submission did not account for censoring time, and this might have given the impression of a true event time. While re-labelling the no-progression group as "censored" would be an option, we are confident that our detailed censoring time-aware survival analyses within the manuscript provides full support for a clear separation of high-risk and low-risk cases and associated molecular groups according to $METH^{low/high}$ classification that is essentially derived from a gradient alongside principal component 1 of our PCA plot. We have thus removed this panel from our manuscript for clarity.

e. Mean and SD are not appropriate summaries for survival data in this study (e.g., lines 207-208). The KM and Reverse KM methods will give appropriate median and IQR estimates, as possible.

Response: We agree that mean-based summaries are inappropriate for censored survival data. The initially identified long-term progression-free cases were therefore redefined using a time-based landmark criterion (> 10 years progression-free, currently applied threshold to stop MRT surveillance for meningioma patients at our hospital upon unremarkable findings), which preserves the originally selected cases while providing a Kaplan-Meier-compatible definition. This has been added to the Results section of our manuscript on page 10 as follow:

"Furthermore, to serve as a control population for bona fide benign meningiomas, we included primary meningiomas from the Discovery cohort with a landmark threshold of 10 years of PFS (n = 13), which substantially exceeded the cohort median PFS of 58.2 months (95% CI 40.1-78.3)."

3. Pseudoreplication in longitudinal analysis may lead to Type 1 error

Response: We now account for tumour samples coming from the same patients as outline below.

a. Simple one-way ANOVA cannot be used when the groups include the repeated measure categories (e.g. Fig 3b, line 522)

Response: We thank the reviewer for raising this important point. We have revised the analysis to explicitly account for repeated measurements from the same patient. Patient identity was added to the dataset, and statistical comparisons between conditions were performed using a linear mixed-effects model for continuous data types with condition as a fixed effect and patient ID as a random effect. This approach accounts for within-patient dependence arising from multiple tumour samples per patient and avoids pseudoreplication. Pairwise contrasts were computed using estimated marginal means with Tukey adjustment. For categorical data such as WHO grades involving repeated measures, we used an ordinal mixed-effects model and used a likelihood ratio test to test significance. The corresponding code is provided. The Methods section has been updated accordingly on pages 32 and 33 as follows:

"For comparisons involving repeated measures, differences between conditions were tested using a linear mixed-effects model with condition as a fixed effect and subject as a random effect, using a Type III F-test to assess the statistical significance of the condition effect. Pairwise comparisons between conditions were performed using estimated marginal means with Tukey adjustment for multiple testing. For categorical data such as WHO grades involving repeated measures, we used an ordinal mixed-effects model and used a likelihood ratio test to test significance."

b. The "nested t-test" (e.g., line 237) is not a precise description and may indicate pseudoreplication. Please clarify.

Response: In analogy to our answer above, we here also use a linear mixed-effect model to account for repeated measures, and provide the Type III F-test result at this position in the revised version of the manuscript on page 11:

"We found profound hypomethylation of methylation cluster-associated CpG sites in normal meninges and benign meningiomas, whereas malignant meningiomas were characterized by significant hypermethylation (Fig. 5b; Type III F-test with Satterthwaite's approximation: $F(5, 35.25) = 12.72$, $P = 4.27 \times 10^{-7}$)."

c. Clustering of primary-recurrent samples within case was not passed to the trajectory analysis algorithm.

Response: We appreciate this important point from the reviewer. However, Slingshot operates on a low-dimensional embedding and cluster labels, and does not accept any blocking or random-effect variables[11]. Therefore, patient-level dependence cannot be directly modeled inside Slingshot. In order to address this point, we have thus performed a patient-aware sensitivity analysis by employing a pseudo-bulk/centroid approach. We have computed patient-level centroids for all samples from the same individual and tested whether trajectory structure is preserved. We have found that lineage topology and pseudotime ordering were highly concordant with the full-sample analysis. This indicates that the inferred trajectory is not driven by within-patient similarity. This approach is now mentioned within the Methods section on page 26 as follows:

"As an additional sensitivity analysis, we computed patient-level centroids of samples in PCA space and used those for trajectory inference to account for non-independence of samples from the same patient."

The corresponding data is now shown Extended Data Fig. 7d,e:

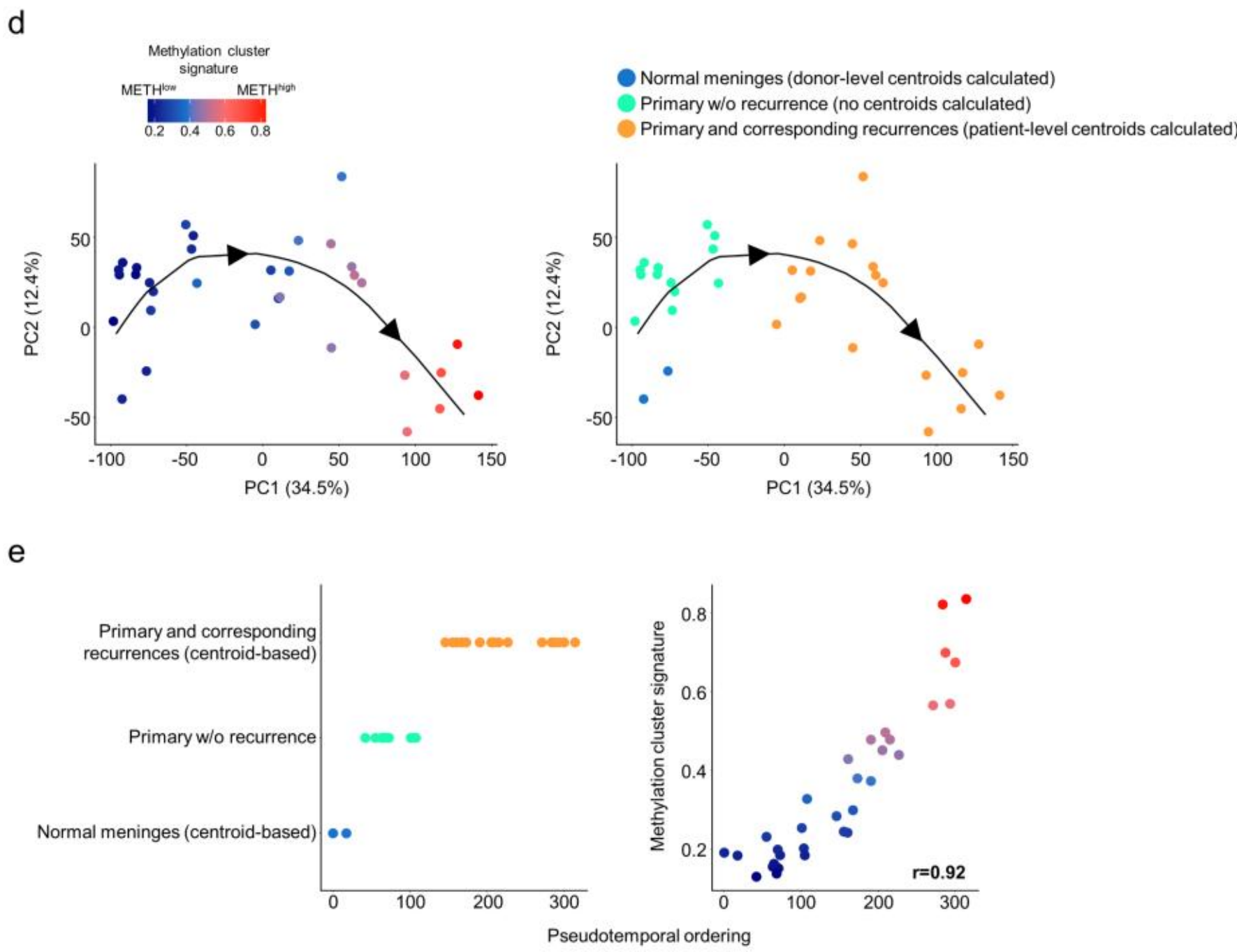

Extended Data Fig. 7: Trajectory analysis for normal meninges and meningiomas based on DNA methylation profiles. *(…)* ***d****, Trajectory analysis was repeated based on donor-level (normal meninges) and patient-level centroids (recurrent meningioma) to account for non-independence of samples from the same donor/patient.* ***e****, Pseudotime analysis for centroid-based trajectory inference.*

4. Methods are not fully aligned with presented text or submitted R-code.

Response: We now ensure that our Methods section is fully aligned with presented text and our R code.

a. Sample-level QC evaluation is described (lines 483-485) but not present in the code. Please verify.

Response: We want to apologize for not including this code within the initial submission. The full code has now been made available at Anonymous Github in section "01_QC_preselect", and will be published on regular Github upon the acceptance of this manuscript.

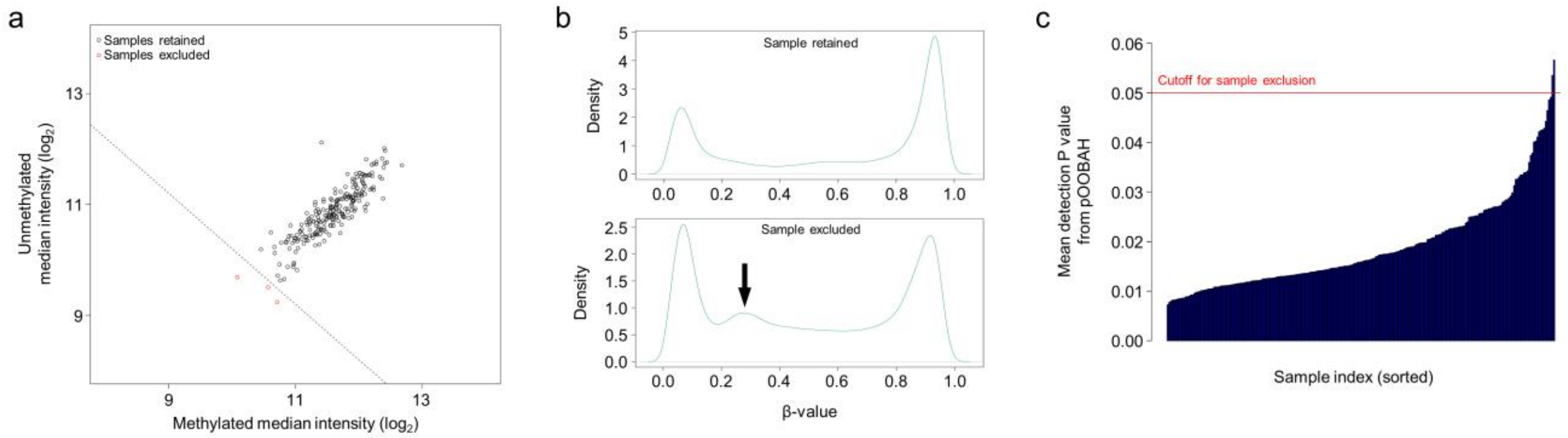

Supplementary Fig. 1: Quality control of DNA methylation arrays. a-c*, Sample-level quality assurance of methylation arrays from meningioma samples of the Discovery cohort. Samples were excluded based on abnormal median methylation intensities (****a****), unusual β methylation value distribution (****b****), or insufficient signal quality as judged by detection P values (****c****). The final Discovery cohort contains a total of n=231 meningioma samples.*

b. Clarify in the methods that p-value filtering excludes probes with any missing data, creating a complete case analysis (lines 485-488)

Response: Indeed, this should have been mentioned in the previous manuscript, and a sentence detailing this has been added to the Methods section on page 23 as follows:

"All probes with missing data in any sample after probe masking were excluded from further analysis, generating a complete case dataset."

c. Exploration of PC1 – PC3 was conducted to select PC1, including checking association with clinical factors. This is not described in the methods.

Response: We now provide detailed explanation that we used PCA in order to identify probes most associated with adverse clinical outcome within the Methods section of our manuscript. Similar to the initial submission, we have performed these analyses on subsets of the top 10.000, 50.000, and 200.000 most variable methylation probes in our dataset. As this implicated the first four principal components to be of potential interest, we now feature separation of tumour samples alongside the first four components. As predictive markers for adverse clinical behaviour, we now employ CNS WHO grading[7], methylation-based molecular groups as suggested by colleagues from Toronto[2], and integrative risk scores[12]. As a quantitative measure of discrimination of marker distributions along individual principal components, we now calculate the weighted pairwise mean overlap for each component. These new data are now shown in Fig. 1b, Extended Data Fig. 1, and Supplementary Fig. 6. The description of our workflow within the Methods section of our manuscript on pages 23/24 reads as follows:

"Principal component analysis (PCA) was performed using the prcomp function in R with parameters center = TRUE, and scale. = TRUE. We found that the total number of most meaningful components was not majorly affected by the number of top variable DNA methylation probes. In order to select for probes most associated with adverse clinical outcome, we investigated which principal component was best able to discriminate meningioma samples based on previous classification schemes predictive of clinical outcome including CNS WHO grading[6], molecular groups as defined by colleagues from Toronto[19], and integrated

risk score[25]. As a measure of discrimination alongside a given principal component, we calculated the weighted pairwise mean overlap of distributions of distinct grades, groups, and risk scores. This was done for the top 10,000, 50,000, and 200,000 most variable probes. The best separation of clinically relevant subgroups was seen alongside PC1 in the 10,000 probes dataset, which was used for all downstream analyses."

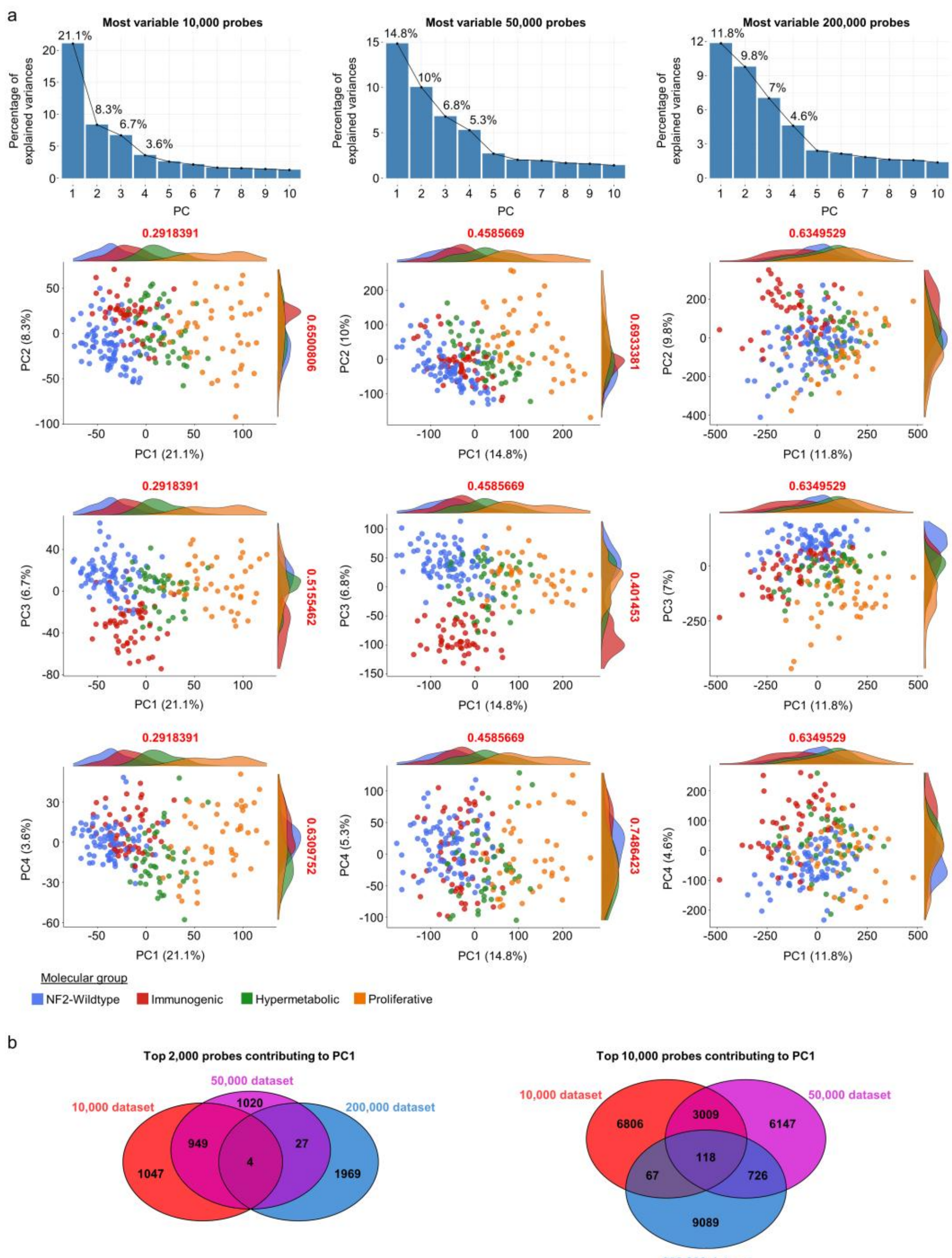

***Extended Data Fig. 1: Principal component analysis identifies recurrence-associated methylation probes based on molecular group classification. a**, Scree plots for the first 10 principal components (PCs) based on varying subsets of most variable methylation probes within the Discovery cohort (top). The first three to four components explain a major part of variance in the dataset. Plots illustrating clustering of*

meningiomas according to the first four components with molecular groups indicated (bottom). Density margins illustrate the distribution of corresponding subclassifications along individual components. Weighted pairwise mean overlaps indicated in red were calculated as a measure of separation. Best separation of low grade/good prognosis cases (NF2-Wildtype/Immunogenic groups) and higher grade/intermediate/bad prognosis cases (Hypermetabolic/Proliferative groups) is seen on PC1, but also exists to some degree on PC2. PC3 separates immunogenic meningiomas from all other cases. ***b**, Venn diagrams illustrate the overlap of probes contributing most to PC1 (top 2,000 and 10,000) in distinct subsets of most variable probe datasets.*

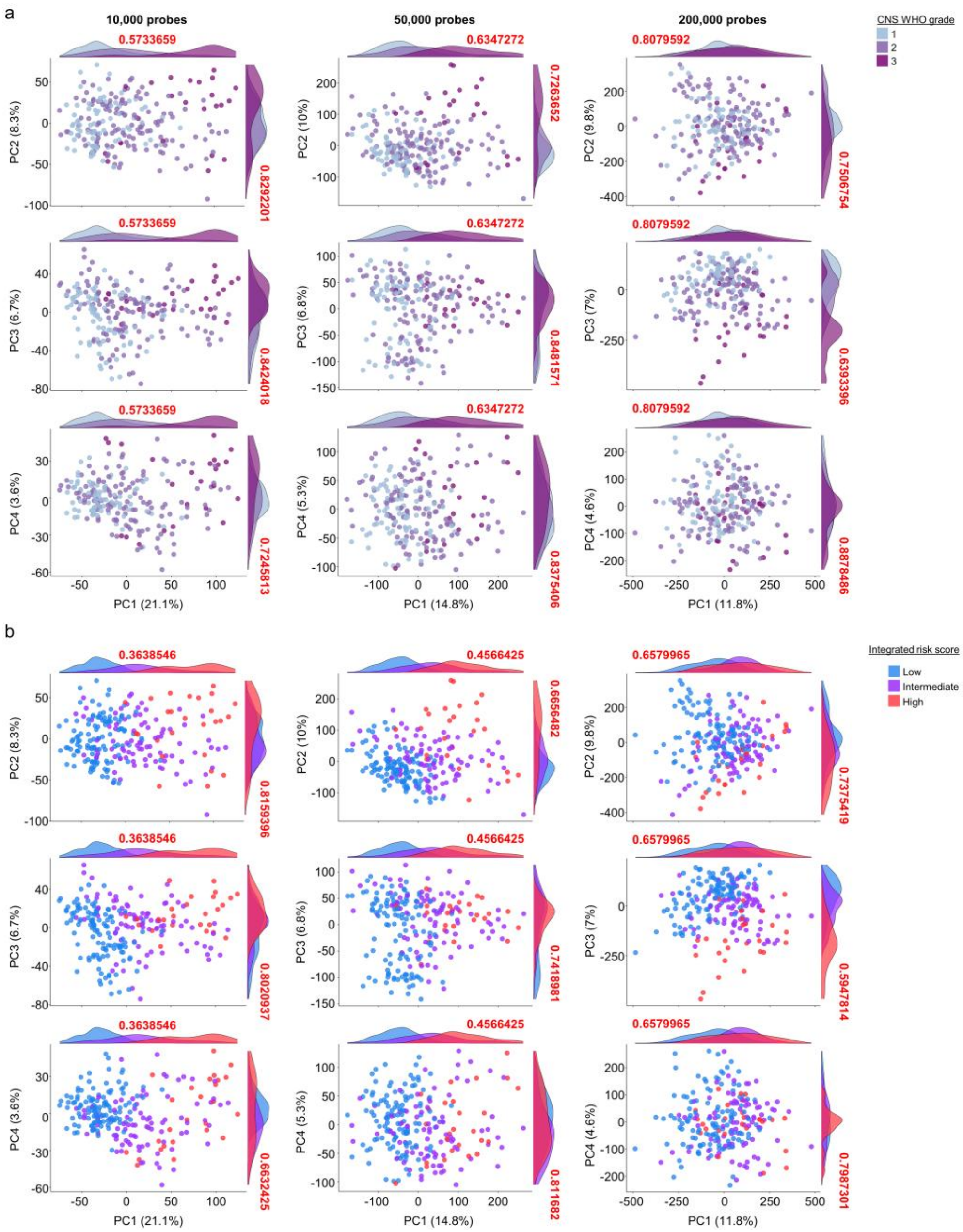

***Supplementary Fig. 6: Principal component analysis using additional classification schemes. a-b**, Plots illustrating clustering of meningiomas according to the first four components with CNS WHO grade (**a**) or integrated risk score (**b**) indicated. Density margins illustrate the distribution of corresponding*

subclassifications along individual components. Weighted pairwise mean overlaps indicated in red were calculated as a measure of separation.

d. The use of multiple clustering models to apply a prior classifier (lines 508-516) is unclear and is not included among the submitted code. The results indicate that gradient boosting (lines 104-5) is used for this classification task, but this method is not described and no code is provided.

Response: We want to apologize for not providing a clearer explanation of our approach to use machine learning for prediction problems. While DKZF meningiomas groups were predicted using an established web application (now featured on https://epignostics.com), a similar prediction tool was not available for other molecular meningioma classifications. We therefore generated models to predict either molecular meningioma groups based on a previous publication with available raw data[2], or methylation cluster assignment based on our own Discovery cohort. As a default, we selected the top 10,000 methylation probes to set up a classifier with the following algorithms available within the caret R package (v7.0-1): random forest, support vector machine, k-nearest neighbors, nearest shrunken centroids and stochastic gradient boosting. The final model was selected based on highest accuracy. In the final analysis, the best performing model was a support vector machine, and this has been stated within the text. PCA to visualize the overall discriminative potential of modeled contrasts, accuracy and Cohen's kappa, as well as ROC curve analysis for predicting molecular groups and methylation clusters are now shown in Supplementary Fig. 3. The full code used for machine learning-based predictions within our study can be found at Anonymous Github in section "04_machine_learning_prediction". The text within the Methods section of our manuscript on pages 24 and 25 now reads as follows:

"Global DNA methylation profiles were used for the prediction of molecular-based classification of meningiomas as defined by previous groups. DKFZ groups[18] were assigned using the Heidelberg CNS Tumour Methylation classifier (v12.8) previously available at www.MolecularNeuropathology.org. DKFZ groups and other classification schemes derived from DNA methylation data alone or from an integration of various data levels are referred to as "molecular classifications" within our manuscript[11]. When referring to "molecular groups" derived from DNA methylation patterns within our manuscript, we stick to the proposed nomenclature employed by colleagues from Toronto unless otherwise stated. We used a machine learning approach to assign meningiomas from our cohorts to Immunogenic, NF2-Wildtype, Hypermetabolic, and Proliferative molecular groups based on a previous cohort[2]. As a default, we selected the top 10,000 methylation probes to set up a classifier with the following algorithms available within the caret R package (v7.0-1): random forest, support vector machine, k-nearest neighbors, nearest shrunken centroids and stochastic gradient boosting. The final model was selected based on the highest accuracy. The selected classifier was then applied to our human meningiomas. Methylation cluster assignment for meningiomas samples outside of our Discovery cohort was assigned accordingly. Models were trained and evaluated using 10-fold cross-validation implemented in the caret package, with accuracy used as the primary tuning metric and Cohen's kappa reported as a complementary measure of agreement. For binary outcomes, discriminative performance was further assessed using standard receiver operating characteristic (ROC) analysis. For multiclass outcomes, ROC analysis was performed using a one-vs-rest strategy with micro-averaging, based on cross-validated hold-out predictions. Integrated risk scores were calculated as previously described[25]."

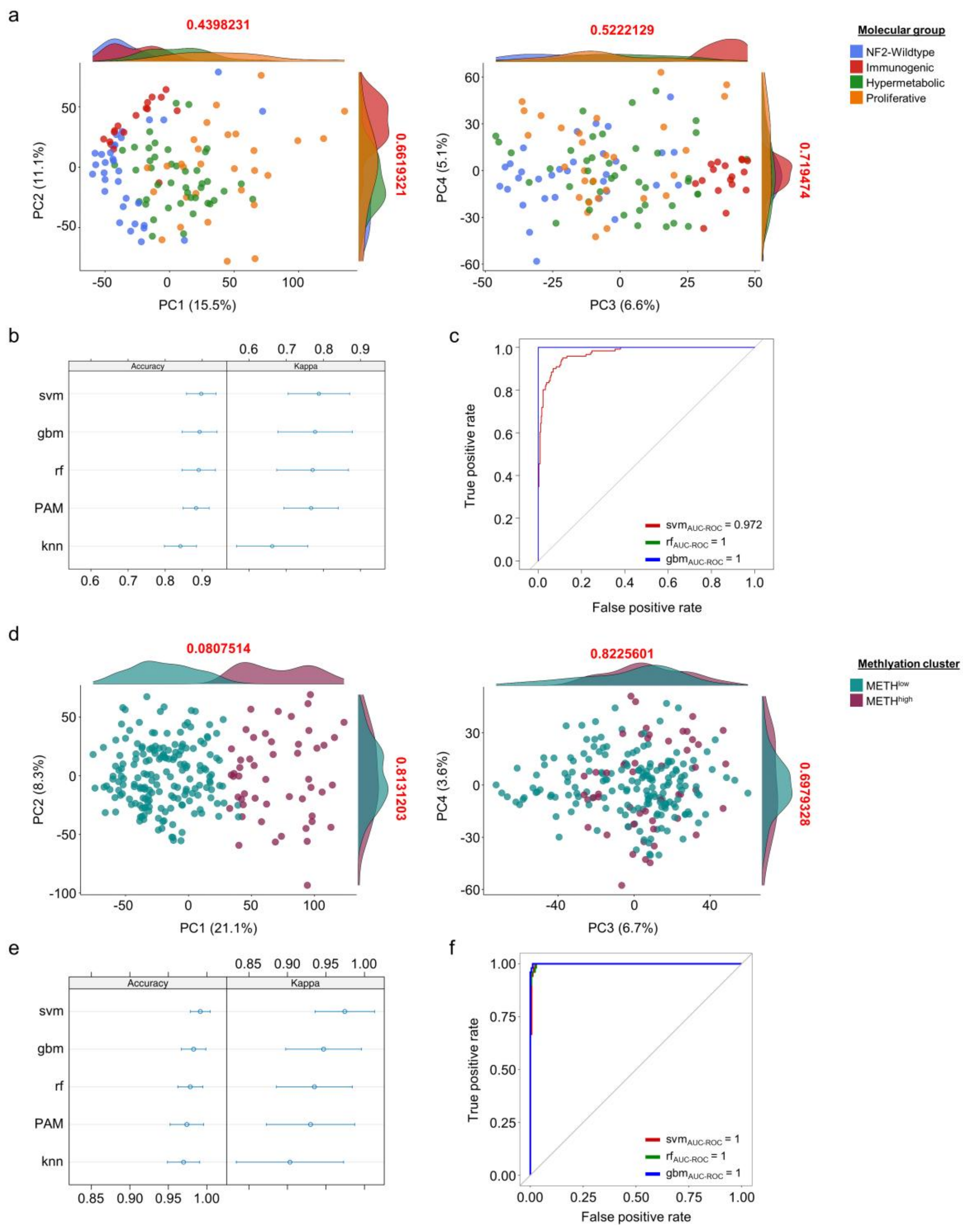

***Supplementary Fig. 3: Data structure and classification performance metrics for classification models. a**, Data structure in the meningioma cohort from Nassiri et al. 2021 (n = 121) with respect to molecular groups in form of a PCA plot. **b**, Performance of models to predict molecular groups as judged by accuracy and Cohen's kappa. Employed algorithms were: support vector machine (svm), stochastic gradient boosting (gbm), random forest (rf), nearest shrunken centroids (PAM), and k-nearest neighbors (knn). **c**, Micro-averaged ROC curve for multiclass classification performance regarding molecular groups for selected models. **d**, PCA plots for the Discovery cohort separating METH^{low} and METH^{high} meningiomas. **e**, Accuracy*

and Cohen's kappa for several models trained on the contrast of METH^{low} and METH^{high} meningiomas. ***f****, ROC curve for binary classification of METH^{low} and METH^{high} meningiomas for selected models.*

e. The work conducted in the submitted code file for differential methylation analysis is not well described in the methods. Also, the data pre-processing and filtering steps in this code file are different than the steps in the code file with PCA and clustering. Please ensure that the analysis-ready data for the Discovery set is consistent or explain the rationale for the difference.

Response: We have revised the Methods section to more clearly described the differential methylation analysis, including the level of analysis, multiple-testing correction, and cutoffs for significance calling. The full code used differential methylation analyses within our study can be found at Anonymous Github in section "05_differential_methylation". This section on page 26 now read as follows:

"Differential DNA methylation in meningioma methylation clusters, or in between meningiomas as compared to normal meninges, was assessed using the DML function implemented in the SeSAMe package. These analyses were conducted at the single-CpG level using previously calculated β-values. Statistical significance was assessed with P values adjusted for multiple testing using the Benjamini-Hochberg false discovery rate. CpG sites with an absolute estimated β-value difference of 0.2 for a given contrast and adjusted P value of < 0.05 were considered differentially methylated."

As already stated in the general remarks in the beginning of this response letter, we have profoundly harmonized our pre-processing pipeline for the generation of β-values to uniformly rely on the sesame R package. This resulted in slight changes in the number of methylation probes kept within the Discovery cohort after pre-processing and filtering (initial submission: 538,104 probes kept; revised manuscript: 527, 352 probes kept). Additionally, as described above, we now also include batch correction for significantly confounding factors in the revised manuscript. This new underlying initial dataset has been used for all subsequent analyses.

f. Multiple sample sets are introduced in the code but not described in the methods – e.g., normal meninges, characteristics of the UCSF dataset, which and how many cases were profiled with RNA seq.

Response: We apologize for not giving more information on these datasets within the initial submission. We have added this information now in Supplementary Table 7 for the RNAseq data, and give further specifics within the Methods section of the paper on pages 24, 25, and 28 as follows:

"Findings on methylation clusters from the Discovery cohort were validated in a larger meningioma cohort (n = 565) from UCSF[20], comprising 456 primary and 109 recurrent tumours, with 388, 142, and 35 cases being CNS WHO grade 1, 2, or 3, respectively. … For integrated analyses of normal meninges and meningiomas, data from meningeal tissue of both dura and leptomeninges covering five different intracranial locations from two healthy donors was retrieved[30]. … Gene expression profiling was performed for a total of 10 tumour meningioma samples, five METH^{low} and five METH^{high} cases."

5. Generation of the Meth-low vs Meth-high groups

a. PC 1 captures more variance with 10K probes, but the 50K probe subset is selected, please clarify

Response: We want to point out at this stage that PCA is used in the manuscript as an exploratory tool to define probes associated with clinical meningioma parameters. Initially, we opted to use the 50K probe subset after scree plot analysis in order to balance the number of probes and the percentage of explained variances for not only PC1, but all meaningful principal components (PC1-PC3/4). This being said, however, we certainly agree with this reviewer that PC1 (i.e. the component we determine to be most associated with molecular groups and risk parameters) captures more variance with the reduced 10K dataset, and this stays true for our revised data after adjusting preprocessing steps and batch correction (Extended data Fig. 1a). In the revised manuscript, we additionally calculate the weighted pairwise mean overlap of distributions of distinct WHO grades, molecular groups and integrated risk score categories as a measure of group discrimination alongside a given principal component, which also identified the 10k dataset to more accurately separate clinically relevant subpopulations of meningioma than larger subsets on PC1. These new data are now shown in Fig. 1b, Extended Data Fig. 1, and Supplementary Fig. 6 (please see above). We have thus decided to perform clustering based on PC1-associated probes identified using the 10k dataset in the revised version of our manuscript.

b. The elbow of the scree plot for the hierarchical clustering is not striking and a 3rd mid-methylation cluster appears reasonable.

Response: We agree with this reviewer that the Elbow method alone does not appear decisive to call for a two-cluster solution. Furthermore, we certainly acknowledge a potential sub-separation of $METH^{low}$ meningiomas within our clustering approach in Fig. 1c Therefore, we now strengthen our initial data by adding the Silhouette method as a complementary method that also calls for a two-cluster solution based on probes associated with PC1. Of note, we employ both of these methods on several subsets of most variable, PC1-associated methylation probes (1000-6000 probes; Extended Data Fig. 2a). Additionally, we now also analyze a potential difference in outcome regarding PFS for low-level ($METH^{low}$-a) and mid-level ($METH^{low}$-b) splits of $METH^{low}$ meningiomas. While we note a moderately higher risk for $METH^{low}$-b as compared to $METH^{low}$-a meningiomas (HR=1.29, P = 0.299946; Extended Data Fig. 4b,c), this was far from statistical significance, being in line most tumours in both subsets being NF2-Wildtype/Immunogenic tumours. While these data suggest a reasonable gradient of methylation status of marker CpG sites between $METH^{low}$ and $METH^{high}$ meningiomas, we are confident that these additional data also support our previous findings of a two-cluster solution with clear relevance for clinical behaviour of meningiomas. These new data are now shown in Extended Data Fig. 2a and Extended Data Fig. 4b,c. This is now stated within the Results section on pages 6 and 7 as follows:

"Clustering based on the top methylation probes contributing to PC1 robustly suggested the presence of two divergent clusters, which we termed $METH^{low}$ and $METH^{high}$ based on their disparate methylation patterns (Fig. 1c and Extended Data Fig. 2a-d) …. Separating $METH^{low}$ meningiomas to a low-level and mid-level methylation group did not show any significant prognostic differences within this methylation cluster, further supporting the clinical relevance of our previous separation into two clusters (Extended Data Fig. 4b,c)."

Please note that we now also employ ConsensusClusterPlus[13], a resampling-based framework, to calculate both within-cluster as well as between-cluster stability for all probe subsets. Overall, this further supported strong cluster consistency for a two-cluster solution, and this is now shown in Extended Data Fig. 2b,c and has been described within the Methods section on page 24 as follows:

"Following cluster number selection, consensus clustering was applied to each probe subset using a resampling-based framework to evaluate clustering stability. ConsensusClusterPlus[51] was used to generate consensus matrices and final cluster assignments, from which consensus cluster prediction (CCP) scores were calculated to quantify within-cluster stability. In addition, concordance of tumour cluster assignments across different probes subsets was assessed using the adjusted rand index."

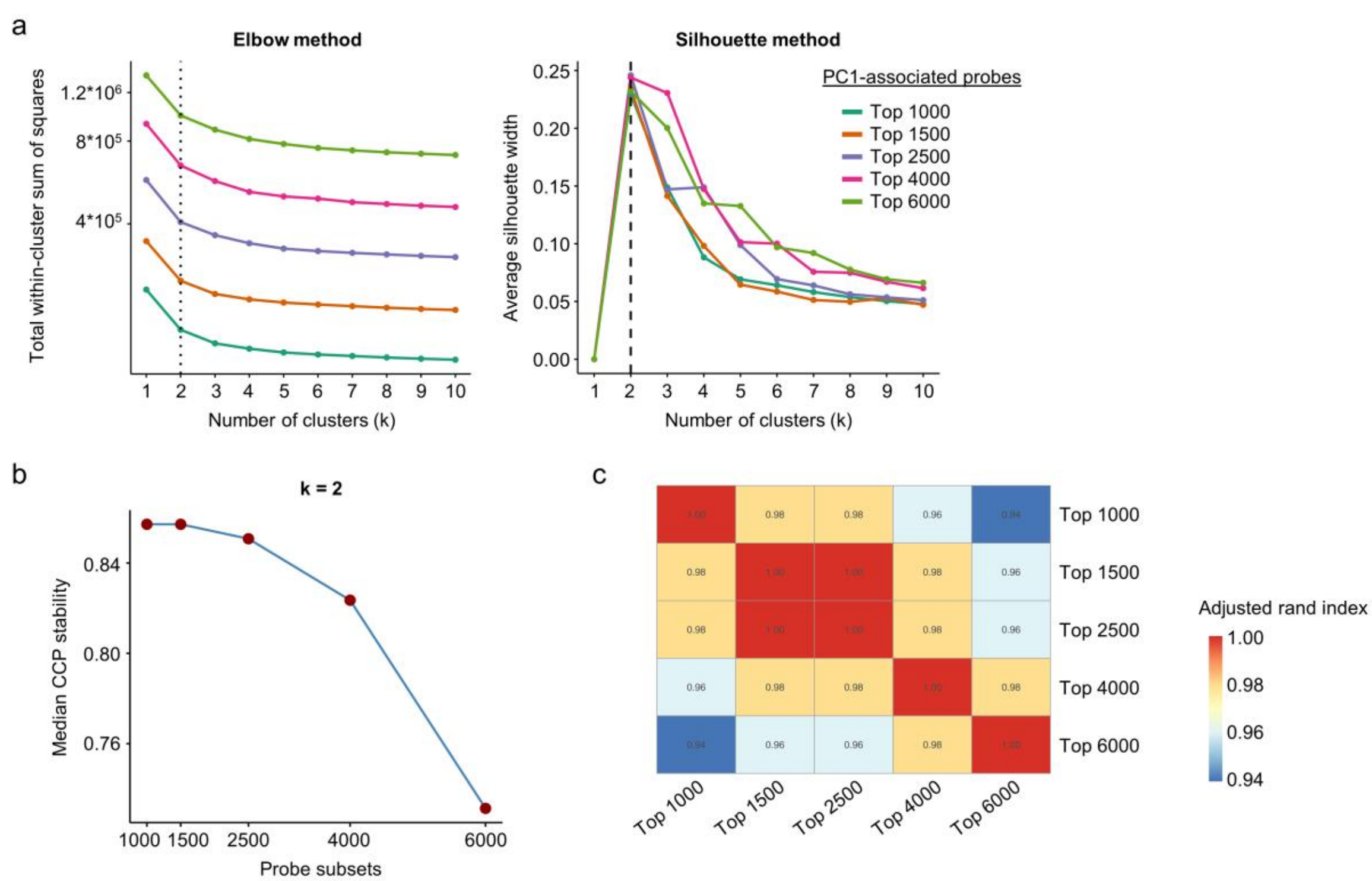

Extended Data Fig. 2: Cluster consistency for hypermethylation signature separating benign from aggressive meningiomas. ***a***, *Elbow and Silhouette methods to determine optimal number of clusters for top PC1-asssociated methylation probes. Analyses were iteratively performed for several subsets of top associated probes.* ***b***, *Consensus clustering prediction (CCP) scores were defined as the mean consensus value between a given sample and all other samples assigned to the same cluster. Consensus clustering only assumed the presence of two clusters. The median CCP scores across samples were used as a summary measure of cluster stability for each probe subset.* ***c***, *The adjusted rand index was used to quantify the concordance of cluster assignments for different probe subsets according to consensus clustering.*

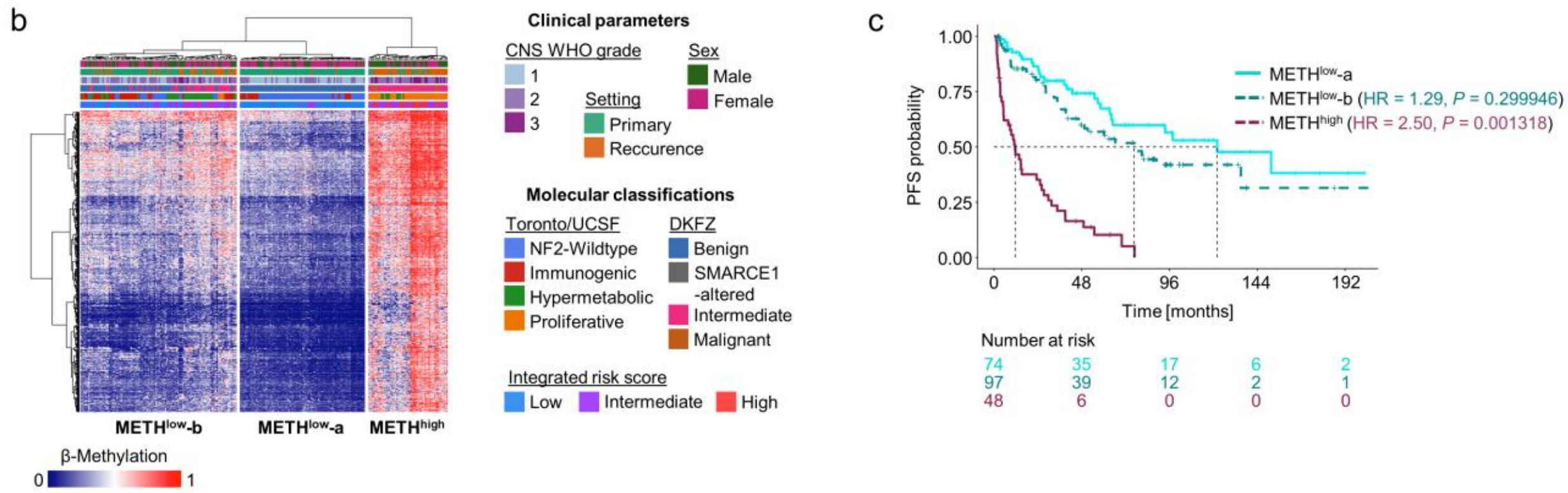

Extended Data Fig. 4: Survival analyses within the Discovery cohort. *(…)* ***b****, Clustering of the discovery cohort by the top 1000 PC1-asssociated probes into three clusters splits METH^{low} meningiomas into low-level (METH^{low}-a) and mid-level (METH^{low}-b) groups.* ***c****, Survival analysis shows a moderate, but not significant difference in PFS for METH^{low}-a and METH^{low}-b tumours.*

Suggested improvements

It is suggested that the authors consider the impact of analyzing primary and recurrent tumours separately. Recurrent tumours tend to be molecularly distinct due to prior treatment and the mix of primary and recurrent tumours may be impacting outcomes. It is also suggested that a three-cluster solution for the generated methylation groups be considered. Figure 1c is suggestive of a mid-level methylation group that separates a fairly distinct NF2-wt subset for the Meth-low group.

Response: We want to thank this reviewer for their effort to improve the scientific approach of our study. As outlined above, we acknowledge that primary and recurrent tumour might present distinct baseline hazards, and we now account for that by stratifying on tumour setting in all of our survival analyses. Furthermore, also outlined above at the corresponding comments, we now provide a series of new analyses supporting our approach to chose a two-cluster solution for our discovery cohort. We acknowledge that several tumours in METH^{low} present somewhat higher methylation levels, potentially suggesting a gradient of methylation rather than a two- or three-cluster solution. However, we are convinced that our new analyses call for a two-cluster solution at current cohort size.

Clarity and context

Figure panels are densely packed and annotation is difficult to read. There is heavy reliance on color to distinguish groups. It appears that “Molecular Group” is used to refer to the Toronto clustering, however, the DKFZ clustering is also under the header of molecular group in figure 1 and elsewhere.

Response: We have improved our figure presentations in order to enhance efficient readability for all readers. Overall, we have adjusted panel sizes in order to loosen up individual panels from figures. Furthermore, we have split Figure 1 to now represent data for cluster assignment and survival analyses in two separate figures. While we don’t see a way to improve dependency on color coding at this point, we have made sure that color coding is consistent across the entire study. Furthermore, we have now adjusted the wording to “Molecular classifications” when referring to several distinct grouping classifications, and

have outlined specifically within the Methods section that "Molecular group" throughout the manuscript strictly refers to the clustering nomenclature put forward by colleagues from Toronto.

Your expertise

While I read the cell experimentation, this bench work is outside of my area of comfort to review in depth.

References

1. Sahm, F., *et al.* DNA methylation-based classification and grading system for meningioma: a multicentre, retrospective analysis. *Lancet Oncol* **18**, 682-694 (2017).
2. Nassiri, F., *et al.* A clinically applicable integrative molecular classification of meningiomas. *Nature* **597**, 119-125 (2021).
3. Choudhury, A., *et al.* Meningioma DNA methylation groups identify biological drivers and therapeutic vulnerabilities. *Nat Genet* **54**, 649-659 (2022).
4. Sahm, F., *et al.* cIMPACT-NOW update 8: Clarifications on molecular risk parameters and recommendations for WHO grading of meningiomas. *Neuro Oncol* **27**, 319-330 (2025).
5. Groff, K.J., *et al.* The effect of TERT promoter mutation on predicting meningioma outcomes: a multi-institutional cohort analysis. *Lancet Oncol* **26**, 1178-1190 (2025).
6. Gui, C., *et al.* Analysis of TERT association with clinical outcome in meningiomas: a multi-institutional cohort study. *Lancet Oncol* **26**, 1191-1203 (2025).
7. Louis, D.N., *et al.* The 2021 WHO Classification of Tumors of the Central Nervous System: a summary. *Neuro Oncol* **23**, 1231-1251 (2021).
8. Wang, J.Z., *et al.* Molecular classification to refine surgical and radiotherapeutic decision-making in meningioma. *Nat Med* **30**, 3173-3183 (2024).
9. Kalamarides, M., *et al.* Identification of a progenitor cell of origin capable of generating diverse meningioma histological subtypes. *Oncogene* **30**, 2333-2344 (2011).
10. Anderson, J.R., Cain, K.C. & Gelber, R.D. Analysis of survival by tumor response. *J Clin Oncol* **1**, 710-719 (1983).
11. Street, K., *et al.* Slingshot: cell lineage and pseudotime inference for single-cell transcriptomics. *BMC Genomics* **19**, 477 (2018).
12. Maas, S.L.N., *et al.* Integrated Molecular-Morphologic Meningioma Classification: A Multicenter Retrospective Analysis, Retrospectively and Prospectively Validated. *J Clin Oncol* **39**, 3839-3852 (2021).
13. Wilkerson, M.D. & Hayes, D.N. ConsensusClusterPlus: a class discovery tool with confidence assessments and item tracking. *Bioinformatics* **26**, 1572-1573 (2010).

Second revision for manuscript NCOMMS-25-62380-A

"DNA methylation profiling identifies long-range epigenetic silencing of clustered protocadherins as a key determinant of meningioma progression"

Point-by-point response letter

REVIEWER COMMENTS

Reviewer #2 (Remarks to the Author):

The authors have responded to each of the reviewers' comments and significantly improved the manuscript.

Response: We thank this reviewer for acknowledging our work.

Minor concerns:

Supplementary Fig. 7: Decision tree. There are some ambiguities in different parts of this decision tree; e.g.: GTR in grade 2: the direct pathway to "shorter surveillance time frames/discuss early RT" should be deleted if methylation clustering is recommended. Grades 1 and 2: if 1p and 22q are present, this should probably lead directly to "shorter surveillance time frames/discuss early RT".

Response: We thank for these detailed comments on the decision tree incorporated into the manuscript during first revision. We want to stress that categories given in our decision tree (e.g. CNS WHO grade, CNVs) reflect several layers of information that might be available to treating physicians rather than recommendations for diagnostic methods. Assuming that CNS WHO grade and extent of resection are available for any resected meningioma, our decision tree aims to provide management recommendations for these cases without molecular information as well. This is why we kept the direct pathway for GTR in CNS WHO grade 2 to "shorter surveillance" to highlight the higher likelihood of recurrence as compared to GTR cases with CNS WHO grade 1[1]. Furthermore, we do acknowledge that CNV information might be available without accompanying methylation data (e.g. by using FISH or sequenced based methods). Thus, we have now included arrows recommending "shorter surveillance" for 1p/22q cases for CNS WHO grade 1 and 2. This has been incorporated in Supplementary Figure 7 accordingly.

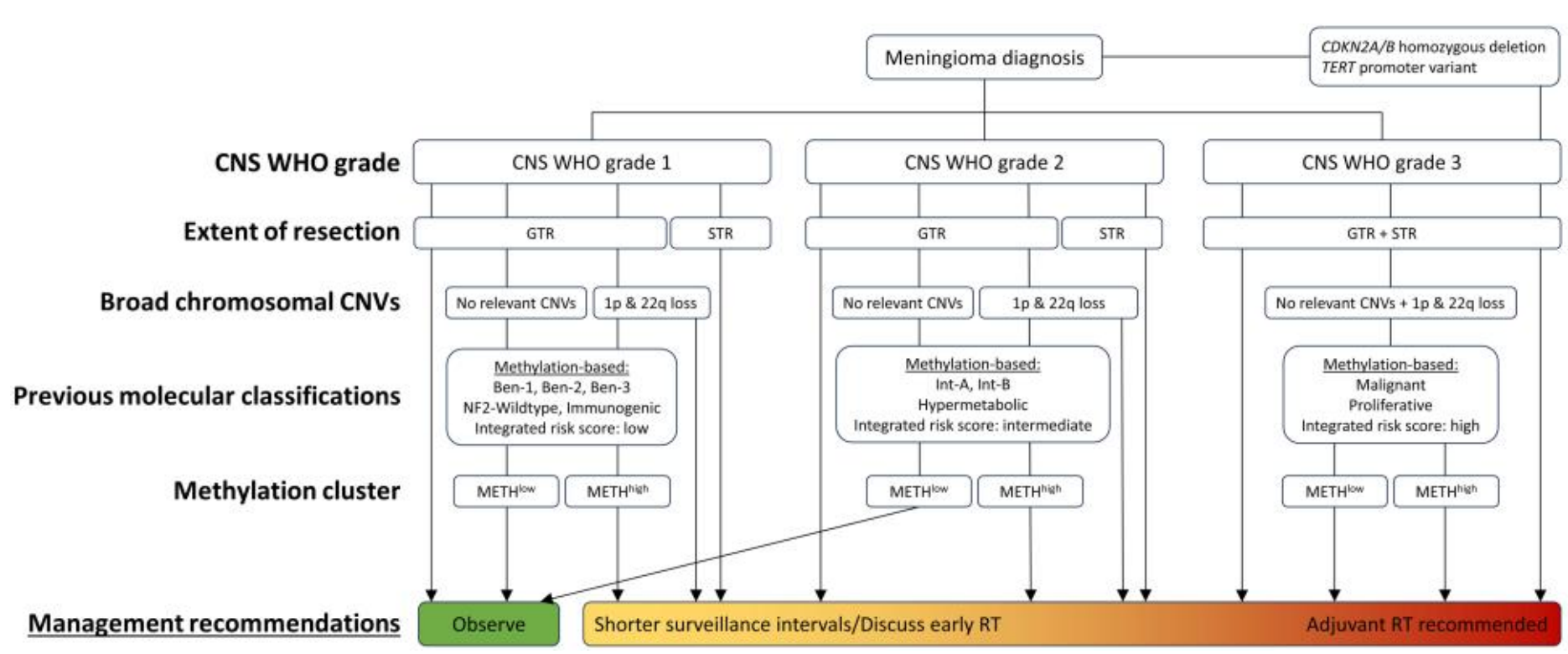

Supplementary Fig. 7: Decision tree for clinical follow-up management. *Recommendations for clinical management of meningioma patients according to CNS WHO grade, EOR, and extended molecular diagnostics. METH^{high} tumors otherwise classified as CNS WHO grade 1 should be subjected to shorter*

surveillance time frames, while METH^{low} tumors with CNS WHO grade 2 and without relevant CNVs might be recommended for observation only.

Extended Data Fig. 4. The HR ratio and p-values seem to be in contradiction with the well-separated survival curves. Is it a log-rank test or Cox proportional hazards models stratified by tumour setting (primary vs. recurrent)? This should be added to the legend.

Response: For all survival analyses throughout our study, we have employed Cox proportional hazards modeling to calculate Hazard ratios and corresponding p values while accounting for competing variables that may affect progression, and this is also the case for Extended Data Fig. 4. The entire code is referenced under "Code availability" and can be retrieved at Github. In response to comments from reviewer #4 during first revision, we stratify all Cox models by tumour setting to account for potential differences in baseline hazard between primary and recurrent tumours, and this was also performed for gender as it has been shown to violate the proportional hazards assumption (Extended Data Fig. a). We agree with this reviewer that there is an obvious contradiction of HR ratios and survival curves, most notably for the investigation of prior RT in METH^{low} cases in Extended Data Fig. 4e. However, in this particular case, this model accounts for adjuvant RT, CNS WHO grade, and extent of resection while stratifying by tumour setting and gender. To highlight this more directly, we have added the following sentence to the figure legend of Extended Data Fig. 4:

"For all survival analyses, Cox proportional hazard models were generated while accounting for competing variables of meningioma behaviour."

Extended Data Fig. 5. Replace "p <" with "p =".

Response: This has been corrected accordingly.

Fig. 7. Nuclear staining is still not evident. I mainly observe, in METHhigh cases, cytoplasmic perinuclear staining. Is it possible to illustrate this with more evident nuclear relocalization?

Response: We agree with this reviewer's opinion that nuclear staining was questionable in the previous pictures. We have re-evaluated the TMAs from the three tumors lying at Q1, Q2, and Q3 with respect to β-catenin immunoreactivity in the METH^{high} cluster. The tumor at Q1 does not show any more nuclear reactivity than what is currently shown in Fig. 7i, and we have kept the original picture as a representative example. We agree with this reviewer that the tumor at Q2 shows strong perinuclear staining at a level that we otherwise rarely see, in particular not in METH^{low} cases. While this staining was abundant across the entire section, we did not see any specific increase in clear nuclear staining in this section, and now keep the original pictures of this case in order to provide a clear representative example. However, when evaluating the tumor at Q3, we noted abundant, clearly nuclear staining across the section, and now provide these new pictures for this case in Fig. 7i.

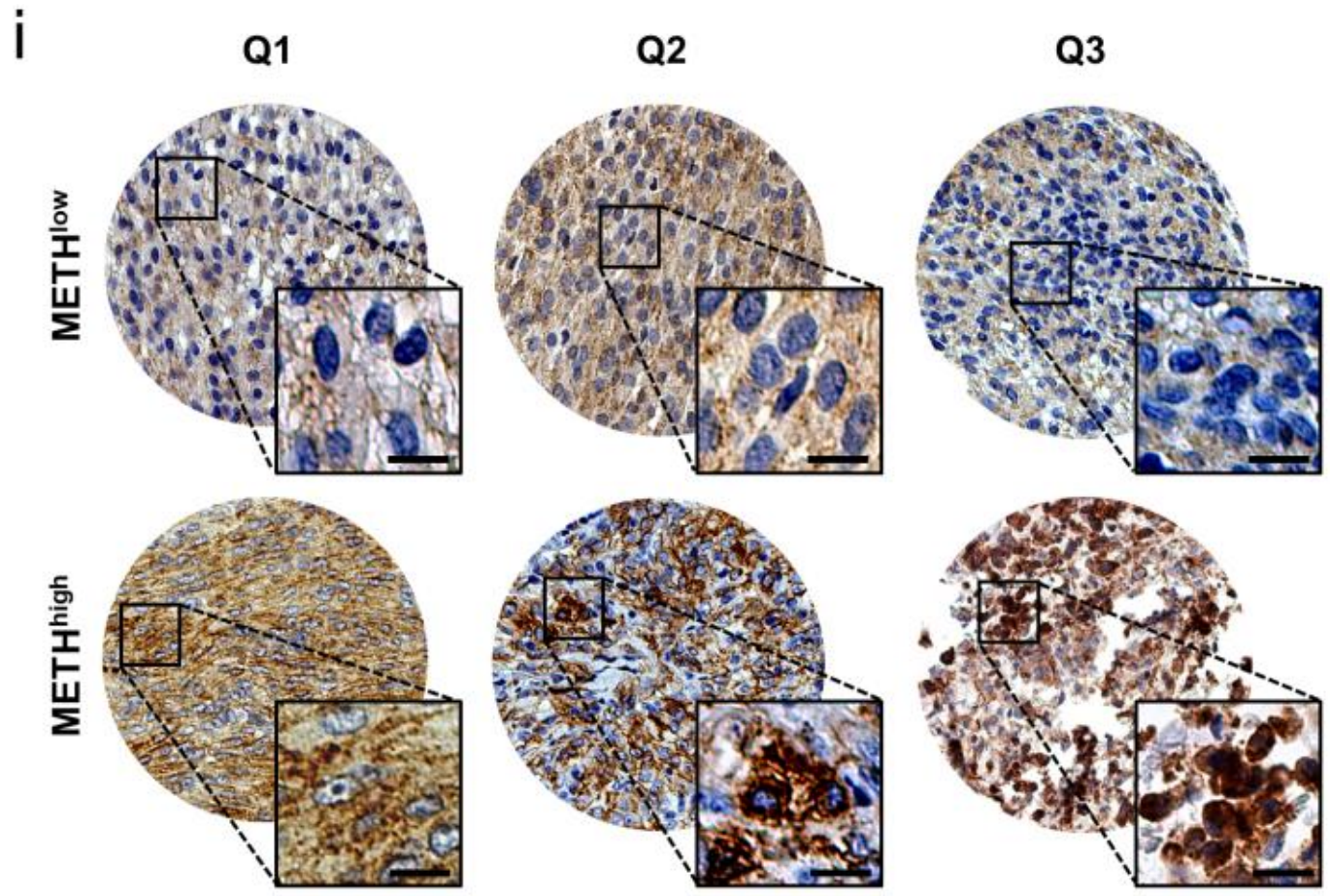

Fig. 7: Tumour-suppressive functions of protocadherins associate with modulation of sub-cellular localization of β-catenin.

***i**, Immunohistochemistry was performed on 80 METH^{low} and 49 METH^{high} human meningiomas on tissue microarrays (TMAs). Exemplary pictures show cases at the 25th, 50th, and 75th percentile with respect to β-catenin immunoreactivity ranking within each cluster. Scale bars: 25 µm.*

Methods, line 732: Was there a specific tool in QuPath to quantify cytoplasmic and nuclear staining separately?

Response: We have used QuPath's built-in "positive cell detection" mode to quantify cytoplasmic and nuclear staining intensities. This tool performs nucleus detection using hematoxylin stain, cell expansion from nucleus to estimate whole cell, and compartment measurement to quantify nuclear and cytoplasmic staining intensities. We have adjusted the text in the Methods section on page 31 to now read as follows:

"DAB signal was further quantified using default settings of QuPath's "positive cell detection" mode. This includes nucleus detection using hematoxylin counterstain, cell expansion from the nucleus to estimate whole cell area, and compartment measurement to quantify nuclear and cytoplasmic intensities."

Radiotherapy and PFS, lines 176–188. In this retrospective study, because of uncontrollable biases, the term "effect" of RT is not appropriate; it should be replaced with "correlation [of both prior and adjuvant RT] with (…)".

Response: This has been adjusted as suggested on page 7.

Typo: In the Extended Data Fig. 10 title: BEN-MEN-1 instead of "BEN-MNE-1".

Response: This typo has been corrected in what is now the title for Extended Data Fig. 11.

Line 394: "for an additional 529 meningiomas"; suggestion to replace with "for 529 additional meningiomas" or "for an additional cohort of 529 meningiomas".

Response: This has been adjusted accordingly on page 17.

Reviewer #3 (Remarks to the Author):

The authors have revised the manuscript in accordance with the reviewers' comments and added valuable new data and analyses. I have no further concerns.

Response: We thank this reviewer for his encouraging words acknowledging our work.

Reviewer #4 (Remarks to the Author):

Thank you for the thoughtful and complete address of the prior feedback. The following comments are minor edits for clarity.

• Line 129-130 - progression is not directly measured please change "most associated with meningioma progression" to "most associated with meningioma progression risk groups"

Response: We have adjusted this part of the text on page 6 as suggested by this reviewer.

• Line 671 - typo: please change "war" to "was"

Response: This type on page 30 was corrected accordingly.

• Line 784-785 - The indicated R code cannot be found in the repository. Please investigate why Figure 7c and 7f do not have error for the cytoplasmic fold change values? If these are group means, then variability would be expected around the mean. If these are 1.0 by design from a paired assessment, then consider a reference line since the cytoplasmic value is built into the fold change.

Response: We apologize for this confusion and agree with this reviewer that data presentation might be misleading. Indeed, for analyses investigating fold changes of mean fluorescence intensities, cytoplasmic values are 1.0 by design from a paired assessment, and thus do not have any error estimate. We thus followed this reviewer's advice to omit data points for cytoplasmic readings and include a horizontal dashed line to illustrate the cytoplasmic reference level. For analyses investigating percentage of cytoplasmic or nuclear β-catenin positive cells, we keep showing data for the cytoplasmic fraction as these have error estimates. All code for the analyses of cancer cell line-associated work can now be found in code file "08_cell_lines.R". These changes have been incorporated in Fig. 7c and f, as well as Extended Data Fig. 11c as follows:

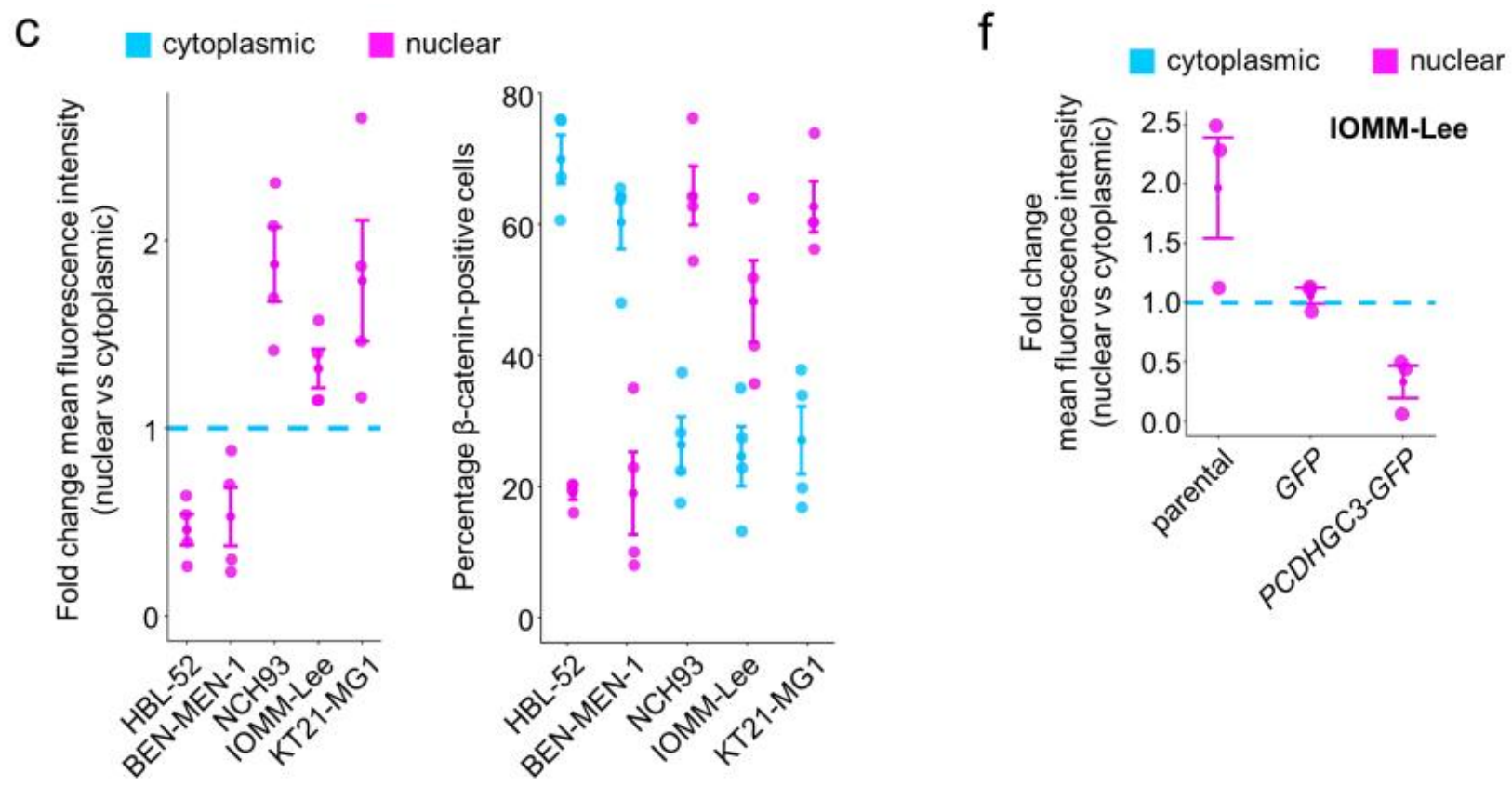

Fig. 7: Tumor suppressive functions of protocadherins associate with modulation of sub-cellular localization of β-catenin.

***C**, Quantification of sub-cellular -catenin signal (cytoplasmic or nuclear), measured as mean fluorescence intensity or expressed as percentage of β-catenin-positive cells. Dashed blue line illustrates the cytoplasmic reference intensity for fold change calculation. Error bars represent mean ± SE.*

***f**, Quantification of cytoplasmic and nuclear β-catenin signal in parental, control, and PCDHGC3-GFP-expressing IOMM-Lee cells. Dashed blue line illustrates the cytoplasmic reference intensity for fold change calculation. Error bars represent mean ± SE.*

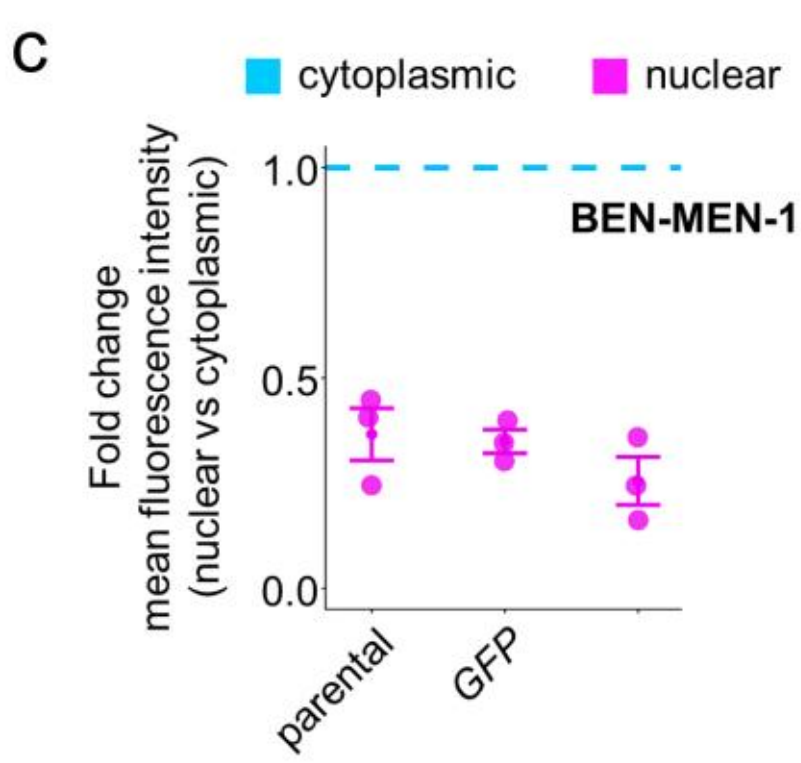

Extended Data Fig.11: Effect of PCDHGC3 overexpression on β-catenin localization in BEN-MEN-1 cells.
***c**, Quantification of cytoplasmic and nuclear β-catenin signal in parental, control, and PCDHGC3-GFP-expressing BEN-MEN-1 cells. Data is shown as fold change in mean fluorescence intensity in the nuclear vs cytoplasmic compartment. Dashed blue line illustrates the cytoplasmic reference intensity. Error bars represent mean ± SE.*

• Extended figure 4 f-g -- The KM plot uses 4 groups that do not account for the immortal time bias of adjuvant RT and other factors that impact decision to add RT are missed (see code file 06_survival beginning line 850). Survival curves for RT groups are likely optimistic. However, an appropriate model was used to estimate the HR. Please indicate in the legend that HR estimates are from an adjusted model.

Response: To highlight this more directly, also in reference to the comment above, we have added the following sentence to the figure legend of Extended Data Fig. 4:

"For all survival analyses, Cox proportional hazard models were generated while accounting for competing variables of meningioma behaviour."

• Since the hypotheses for Kruskal-Wallis and ANOVA multi-group testing differ, when a set of tests are being conducted it is best practice to be consistent so that the results can be meaningfully interpreted between tests. Please consider converting the few ANOVA assessments to KW. (see code file 09_discovery_cnv, beginning line 367)

Response: We have followed the reviewer's suggestion and have applied Kruskal-Wallis test for all comparisons in Fig. 3c, with no major influence on data interpretation.

Reviewer #4 (Remarks on code availability):

Code submission - In reference to the Nature Research code submission check list, this code submission is not for a standalone product, but provides 12 scripts that accomplish most of the analysis in the manuscript. The readme file is a simple abstract for the project, but does provide the operating system and the version numbers for some of the packages that are used. The readme file references a dataset that will be available in GEO and that metadata is in the manuscript. I assume this is in reference to the 231 cases provided by the author as the discovery set and that the manuscript in question is the one under review. This would need clarification once the study is published and unblinded. GEO archive numbers, or other access information, should also be added for all datasets used in the analysis, including the UCSF, Nassiri, normal meninges, and cell line methylation datasets and the RNA seq dataset. The readme file does not directly refer to any of the script files regarding their function or expected output. If this is not in the readme, then each of the 12 files should have a header connecting the code to the manuscript and with references for the called datasets. While I did not access the data to run each script, I was able to load the packages, and I read through the scripts. The readme or script headers would be further improved by indicating which packages are found in CRAN vs Bioconductor. While some of the packages may be obvious to an experienced reader, a novice reader may struggle.

Response: We thank this reviewer for their detailed look at our code description deposited at Github, and agree that further details will enhance reproducibility of our work. We have extensively modified the readme file at Github for this repository, which included: I) addition of all repository numbers for own and external data, II) addition of a description header for each of the individual code files with main tasks and output, and III) indication from where packages can be installed. Furthermore, all archive numbers or alternative ways of data access are now indicated in the Data availability section on page 34 which reads as follows:

"All raw data generated during this study can be found at GEO under accession number GSE304097. Publicly available datasets used in this study include DNA methylation array (GSE180061 and GSE183656) and RNAseq datasets (GSE183656). Due to legal restrictions, data access to DNA methylation arrays from normal meninges will have to be assessed on a case-by-case basis and will be fulfilled by Dr. Tiit Mathiesen (tiit.illimar.mathiesen@regionh.dk), pending positive evaluation of the request by the Legal Department of

Rigshospitalet and Ethical Committee of Region Hovedstaden. Additional preprocessed data files will be made available on Figshare upon acceptance of the manuscript."

Above what is stated, there are no concerns for files 01-05. In files 6-12, the library calls have not been consolidated to the top of the script. If the later load, or reload, is needed (e.g., masking of functions) please clarify.

06_Survival.R - line 729 - introduces a new library (stringr)

07_trajectory_analysis.R - Line 229 reloads the library (ggstatsplot); Line 715 introduces a new library (RColorBrewer); Line 784 introduces a new library (ggplot2)

Response: We thank this reviewer for this important comment. In fact, the later load of several libraries did not serve any purpose (e.g. masking of functions), but rather reflects the process of code development over a longer period of time. We have now made all code files comparable to call all used libraries within the given code at the beginning of the text file.

09_Discovery_CNV.R

Library installation and loading needs clean-up

• Bioconductor is indicated, however, there are redundant calls to install "BiocManager"

• Line 1-4 - the installation call for 'conumee' is missing

• Line 21 - "ComplexHeatMap" is installed, but not loaded until line 258

• Line 23 - "ggbeeswarm" is installed but not loaded

• Line 26 - Install of 'conumee2' package appears to be proprietary from a local library so this cannot be replicated

• Libraries repeatedly added - line 42 (stringr), 217 (stringr)

• Line 539 - ggplot2 reloaded

Multiple txt and csv files are loaded throughout from local directories - please indicate which other script generated these files, provide an external source for these files, or describe the contents so the user can generate a comparable file.

Response: Again, redundant calls are a reflection of computer code generated over a longer period of time, and we are sorry for not deleting redundant calls within the code. All code files now call all used libraries at the beginning, and only call libraries used within the code. While installation of most commonly used packages within our code should be straight forward, the readme file on Github now specifies the most relevant R packages and from where they have been installed, including the version numbers. Please see below our response in reference to the conumee2 R package as well.

At times, data manipulation has been performed in Excel for the generation of .txt or .csv files. We are sorry for not including these intermediate datasets in the initial submission. For all code files, we have now

made available dedicated data folders with all necessary intermediate datasets, as well as helpful intermediate .RDS files such as preprocessed beta values, on Figshare (https://figshare.com/s/ea7852e5723c1972329d) in order to make our analyses fully reproducible.

10_longitudinal_CNV.R

Library installation and loading needs clean-up

• Conumee2 is loaded then removed then installed from a proprietary local library - it is reloaded in line 734

• Libraries are reloaded - Conumee (line 393), sesame (lines 417, 735), ggplot2 (line 1221)

• Libraries added

○ the package 'stringr' is repeatedly loaded on lines 35, 102, 152, 201, 245, 454, 560

○ lines 498 (lme4), 499 (lmerTest), line 500 (emmeans), line 604 (pheatmap), line 631 (data.table), 667 (dplyr), 767 (biomaRt), line 1316 (ComplexHeatmap)

• Etc.

Response: We have substantially re-organized all code files to now call all used R packages at the beginning of the code, and this includes the 10_longitudinal_CNV.R code file.

Clean up unused attempts - code references using old and new conumee

• Line 394 - "important to use conumee, not conumee2, does not work"

• Line 414 - "try with old conumee package"

• Line 416 - "this is a try with the new package"

Issues with coding seem to persist –

• Line 758 - #try using CNV.define_detail for cancer census genes (should however be covered by sig_cgenes below, but somehow is not)

• Line 766 - #does not seem to work, problem with seqnames which are not in seqinfo, means that by default anno function uses seqinfo other than what is given in seqnames

• Line 1458 - ###new try

Provide a header and comments for the function created starting on line 925.

Response: During the course of this study, the conumee R package was repeatedly updated and corrected for bugs, leading from the “conumee” package to the “conumee2.0” and finally the “conumee2” release. This has created several package incompatibilities and errors over the course of the study, and we are sorry for not having removed previous unsuccessful attempts. The code file now includes only the employed final code. All final analyses have been performed with the “conumee2” package that has been installed from Github (https://github.com/hovestadtlab/conumee2) according to maintainer’s

recommendations. All analyses have been performed using the v2.1 realease from Sep 21, 2024, which is available on Github, and should therefore be fully reproducible. We now provide an explanatory header for the custom genomeplot function.

11_RNAseq.R

Lines 19, 22 - Origin of the data is not clear

New library calls - line 149-150

Response: All count data and corresponding meta data are available as text files in a dedicated data folder on Figshare (https://figshare.com/s/ea7852e5723c1972329d), and corresponding raw data repository links to GEO are indicated in the readme file on Github as well as in the Data Availability section of the manuscript. All libraries are called at the beginning of the code.

12_Classifier.R

At the top, indicate that the webapp is being built - beginning with line 108

Response: We now provide specifications on model generation and the code that is used to built the webapp, which now starts with line 114. Additionally, we provide a dedicated data folder on Figshare (https://figshare.com/s/ea7852e5723c1972329d) (data_12_Classifier) that provides necessary data to reproduce the model used in the classifier, and the model itself as a .RDS file.

Reviewer #5 (Remarks to the Author):

In this manuscript the authors perform EPIC methylation profiling on a cohort of 231 meningiomas, then propose a binary classification scheme based on 1000 probes with strongest association to PC1. They find that this scheme recapitulates previously described paradigms (Fig 1d). Intermediate risk (WHO grade 2, Hypermetabolic) were balanced between METH low/high; and Meth low/high could stratify outcomes of WHO grade 2 tumors (but not hypermetabolic or Intermediate integrated risk; Fig 2c). Analysis of longitudinal samples showed expected differences between progressive and benign cases (WHO grade, number of CNVs, etc), and between meningiomas and a small set of meninges samples previously published. They examine genes that show statistical hypo-methylation (23) or hyper-methylation (744) in the METH high subgroup, and find enrichment of HOX genes and protocadherins, which also showed differential expression in some cases. As shown in previous studies of other cancers, the authors suggest that silencing of protocadherin in high-grade meningiomas may result in more nuclear localization of B-catenin, which is known to play a role in meningioma biology. Indeed, overexpression of PCDHGC3 in IOMM-LEE (but not BEN-MEN-1) caused increased cytoplasmic localization of B-catenin, as well as decreased overall abundance of B-catenin.

The authors have addressed the comments provided by Reviewer #1. However, included in the rebuttal is the statement “However, even when compared to purely molecular-based or integrated classification systems, we find more accurate prognostication for intermediate-risk meningiomas”, which does not seem to be supported by Fig 2c (see further discussion below).

Response: We thank this reviewer for their critical view of our data. We have performed additional analyses with respect to our DNA methylation signature and its prediction performance in comparison the previously described prognostication schemes. Please find our comments and new data below.

Comments:

While the authors have demonstrated that their binary methylation classification provides additional prognostic benefit on top of WHO grading, this has now been demonstrated in many large-cohort publications (PMIDs 28314689, 34433969, 34618539, 35534562 among others). To be of added clinical value, this manuscript would need to show a direct prognostic comparison of this proposed classification to these other systems. If the claim is that this new system is only helpful for “intermediate” cases, more evidence is needed to support this (currently figure 2c seems to show that it does not provide prognostic benefit).

Line 127: "Furthermore, within classification groups with difficult estimation of prognosis commonly referred to as intermediate-risk meningiomas, methylation clustering stratified into groups with different clinical behaviour, with METHhigh cluster patients having a significantly or moderately worse prognosis (Fig. 2c and Extended Data Fig. 4d)."

Fig 2c and Extended Data Fig 4d show that Meth Low/High can stratify outcomes of WHO grade 2 tumors, while Fig 2c seems to show that Meth Low/High is unable to stratify outcomes of Hypermetabolic or Intermediate Risk (Maas) meningiomas ($p > 0.05$). This needs to be clearly and explicitly stated in the main text, as it suggests that the Meth Low/High classification does not provide any further prognostic benefit over currently proposed schemes. Unless I have mis-understood, this is in direct contradiction to how the text is currently worded.

Response: We agree with this reviewer’s opinion that our initial claim of prognostic superiority of our signature particular in intermediate-risk cases, more specifically those groups defined by molecular biology (Hypermetabolic/Integrated intermediate risk), was premature and not backed by our own data in full. While we noted the only “moderate” stratification of molecularly defined intermediate risk groups in the previous version of our manuscript, we should have addressed this issue more carefully.

We now provide further details on our methylation signature and perform several levels of direct comparisons to directly compare the prognostic performance of our signature as opposed to previous classifications. We compared more directly our signature with previous classifications on the DNA methylation level. These analyses further showed that our signature strongly aligned with clinically well-defined molecular/risk groups of meningioma, with a predominant significant downregulation of methylation in low-risk tumors (NF2-Wildtype/Immunogenic/low-risk) and upregulation of those sites in high-risk cases (proliferative/high-risk), conserving the diametrically opposed methylation patterns across all groups. Intermediate-risk tumors (Hypermetabolic/intermediate-risk) only showed a minor upregulation of a subset of probes know to be upregulated in clinically aggressive meningioma, and failed to show a clear diametrically opposed methylation pattern. Of note, while CNS WHO grade 3 tumours

showed a clear pattern of preferential upregulation, both CNS WHO grade 2, and in particular CNS WHO grade 1 tumours did not show a clear opposing methylation pattern indicative of low-risk methylation pattern. This suggests that prognostic superiority of molecular-defined groups in prognostication might in large part be derived by better identification of bona fide low-risk meningiomas. These additional results have been added to Extended Data Fig. 9a and are discussed within the Results section on page 13 as follows:

"Additionally, we found that this opposing methylation pattern was well recapitulated in individual molecular groups with clear low-risk or high-risk profiles, while in particular CNS WHO grade 1 tumours did not present the typical low-risk profile of our signature (Extended Data Fig. 9a)."

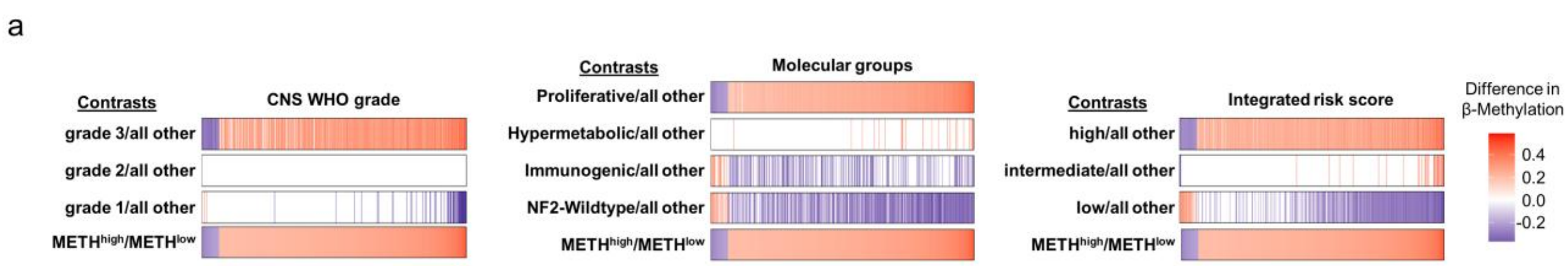

Extended Data Fig. 9: Genes predicted to be affected by differential DNA methylation in METHhigh meningiomas and their validation in gene expression data.

a, *Illustration of shared DNA methylation changes between $METH^{high}$/$METH^{low}$ clusters and individual molecular groups of meningioma. Bottom bar presents all methylation probes significantly different in $METH^{high}$ vs $METH^{low}$ tumors. Top bars illustrate the methylation change of those probes within probes determined to significantly define individual molecular groups (e.g. NF2-Wildtype) when compared to all other meningiomas.*

Despite clear separation by binary stratification, restricted cubic spline modeling[2] of the continuous methylation cluster signature was further used to assess whether risk followed a nonlinear gradient rather than a simple threshold effect. Here, we see a continuous, nonlinear relationship between DNA methylation and risk of progression, showing that increasing methylation is associated with a higher risk of progression over time. Classification schemes show a strong trend to be ordered along this gradient according to their expected clinical behaviour. In particular, methylation clusters capture a biologically meaningful transition along this continuous risk gradient, highlighting the prognostic value of the methylation signature. These results are now shown in Fig. 2d with an adjusted figure title and are discussed in the Results section on page 8 as follows:

"To capture potential nonlinear risk gradients beyond binary stratification, we modeled the continuous methylation cluster signature – defined as the mean methylation of cluster-defining probes – using restricted cubic splines. These analyses revealed a continuous, nonlinear relationship between methylation and risk of progression (Fig. 2d), showing that increasing methylation of cluster-related CpG sites is associated with a higher risk of progression over time."

d

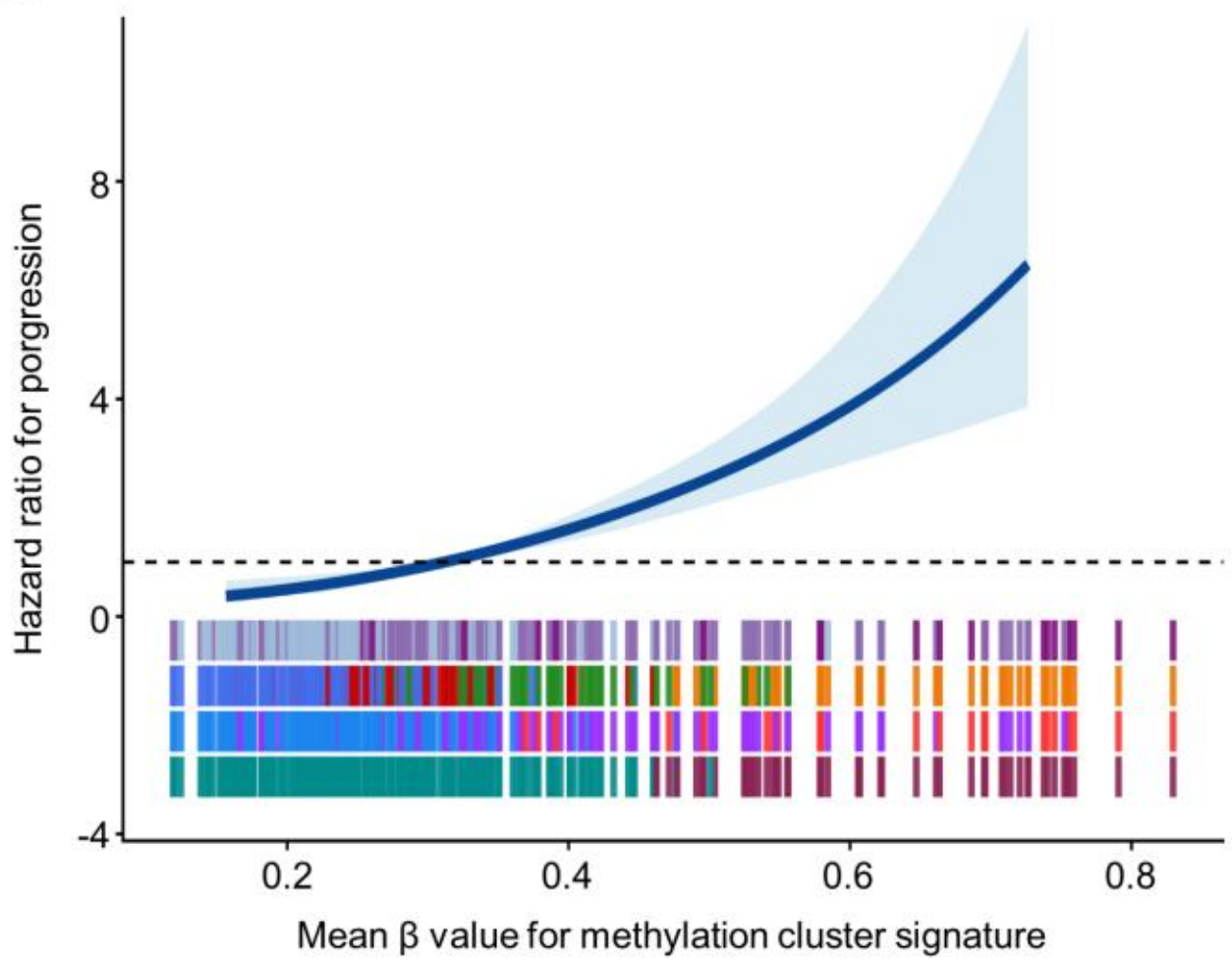

Fig. 2: The methylation cluster signature improves risk stratification across histopathological grades and molecular groups.

d, *Restricted cubic spline modeling showing the nonlinear association between a continuous cluster methylation signature (sample-wise mean β values for cluster probes) and progression risk, with rugs indicating CNS WHO grade, molecular group, integrated risk scores, and methylation cluster affiliation. The tumor sample at the median of the continuous rank was assigned a hazard ratio of 1.*

Additionally, we now provide a more detailed analysis in order to directly compare the ability of distinct classification schemes to predict risk. We now assess differences in performance for discrimination (using c-index), balancing of sensitivity and specificity of predicted risks (integrated discrimination improvement, IDI)[3], reclassification capacity (net reclassification improvement, NRI)[3], and the accuracy of predicted probabilities (Brier score). Since we have seen a nonlinear continuous risk gradient associated with the average methylation level of cluster-defining probes, we have here included our methylation cluster signature both as a binary as well as a continuous variable in survival models. Overall, as expected, we find that methylation cluster signature, both as binary and continuous variable, strongly improves risk prediction of CNS WHO grading alone across the entire cohort, and stratification of CNS WHO grade 2 tumors into low- and high-risk subsets is supported by significant improvements of performance indicators in this cohort as well. While we had initially claimed that methylation clusters improve molecularly-defined intermediate-risk cases, as stratification called for a strong trend and/or borderline statistical significance, these additional performance metrics do not support an additional benefit of the methylation cluster signature in intermediate-risk meningiomas. In contrast, for the entire cohort used here, all employed metrics provide significant support for an improvement in risk prediction when adding the methylation cluster signature as a continuous variable. Thus, while our initial claim does not hold true, we now provide solid data showing that our methylation signature captures a nonlinear risk gradient in meningioma that refines risk prediction in meningioma beyond currently employed classification schemes. These new data are now shown in Fig. 2e,f and Extended Data Fig. 5. Furthermore, this is discussed within the Results section on pages 8 and 9, while “clearly and explicitly stating” (see comment above) that stratification of Hypermetabolic and Intermediate integrated risk meningiomas alone does not meet statistical significance, as follows:

*"Furthermore, methylation clusters successfully stratified CNS WHO grade 2 cases into groups with distinct risk profiles, and similar stratification was seen for molecularly-defined intermediate-risk meningiomas, although this did not reach statistical significance (Fig. 2c and Extended Data Fig. 4d). … Overall, the methylation cluster signature demonstrated improved discrimination and reclassification over current CNS WHO grading, both in univariate as well as multivariate analyses, and this was particular evident when defining the methylation signature as a continuous variable (Fig. 2e and Extended Data Fig. 5a,b). Furthermore, the continuous signature significantly improved performance beyond molecular groups and clinical covariates across the entire cohort, while this was not the case in Hypermetabolic meningioma. Accordingly, we found improved calibration and more accurate absolute risk estimation (Brier score) within the entire cohort in particular for the continuous signature as compared to molecular groups (Fig. 2f; ΔInt_Brier = 0.008, P = 5.15*10^{-9}, Wilcoxon signed-rank test), while this effect was less pronounced in CNS WHO grade 2 meningioma (ΔInt_Brier = 0.004, P = 0.002627, Wilcoxon signed-rank test)."*

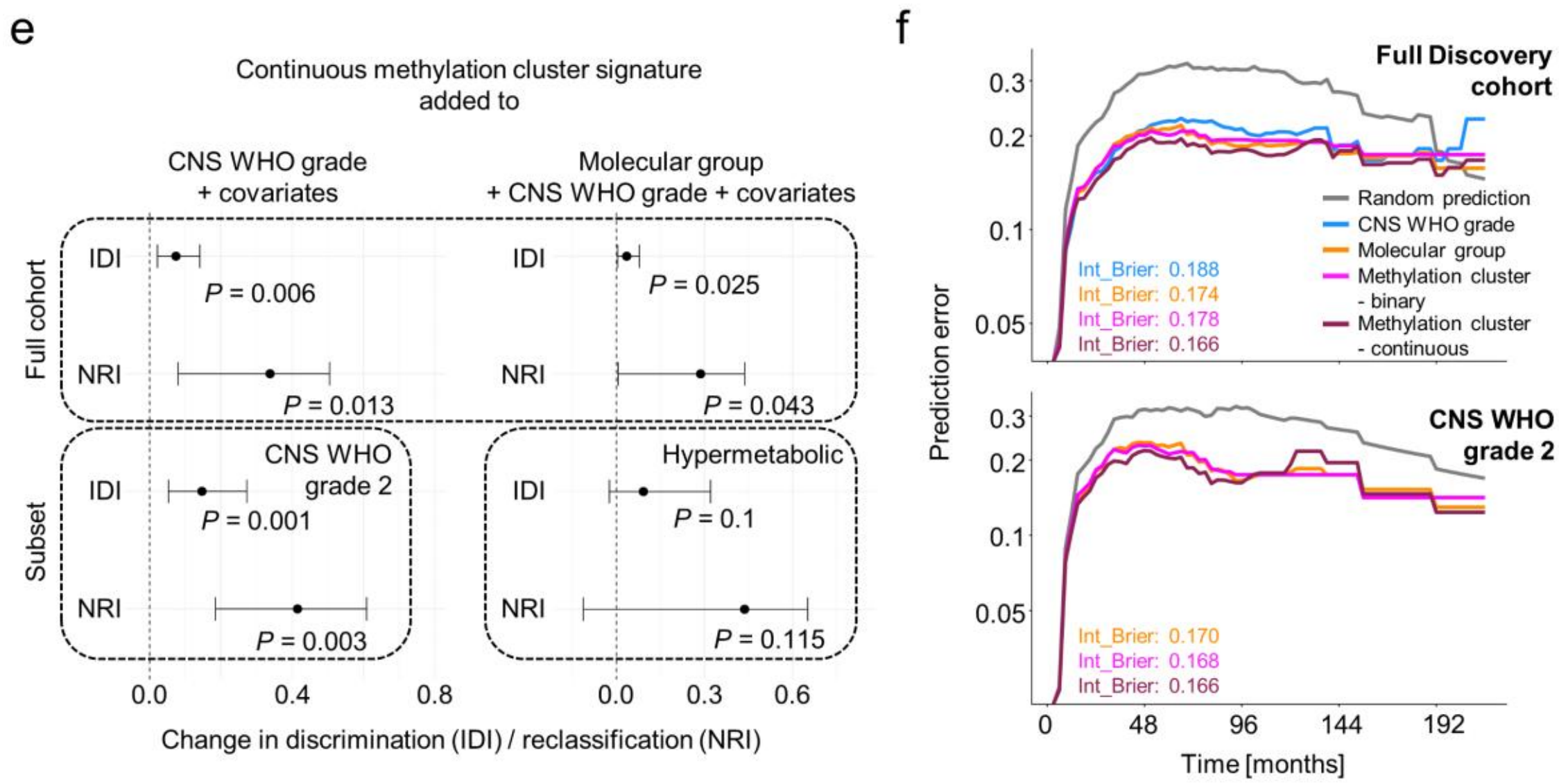

Fig. 2: The methylation cluster signature improves risk stratification across histopathological grades and molecular groups. e, *Point estimates and 95% confidence intervals for integrated discrimination improvement (IDI) and net reclassification improvement (NRI) illustrating the change in discrimination and reclassification associated with inclusion of the continuous methylation cluster signature.* ***f****, Brier prediction plots for indicated models using the full Discovery cohort (top) or only CNS WHO grade 2 meningiomas (bottom). Integrated Brier scores for the entire follow-up time are indicated (Int-Brier).*

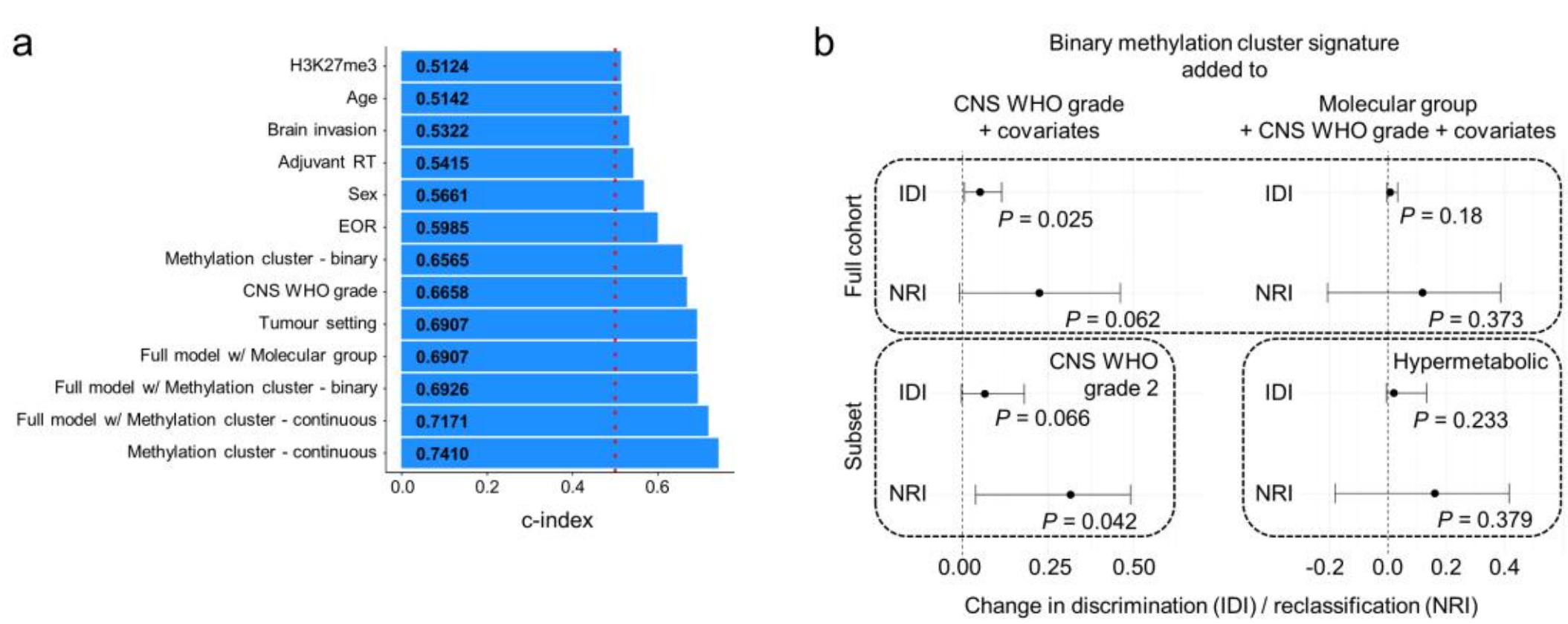

Extended Data Fig. 5: Performance metrics to compare survival models. a, *Bar graph illustrating concordance indices (c-index) for indicated univariate and multivariate models.* ***b****, Point estimates and 95% confidence intervals for integrated discrimination improvement (IDI) and net reclassification improvement (NRI) illustrating the change in discrimination and reclassification associated with inclusion of the binary methylation cluster signature.*

Code for additional statistical comparisons can be found in the 06_survival.R code file and have been added to the Methods section on page 24 and 25 as follows:

"Nonlinear associations between the continuous DNA methylation cluster signature and risk of progression were modeled using restricted cubic splines with three knots, allowing flexible modeling of potential nonlinear relationships while avoiding overfitting. To assess the prediction performance of individual models, we employed both the concordance index (c-index) and the Brier score as implemented in the survival package. Additionally, we assessed the integrated discrimination improvement (IDI) and net reclassification improvement (NRI) using the survIDINRI R package (v1.1-2)[62]."

We are convinced that our dataset of the entire cohort has a sufficient sample size to allow for robust statistical analyses. However, due to low abundance of $METH^{high}$ cases overall and the resulting limited sample size in selected subsets of the survival data (e.g. $METH^{high}$ vs $METH^{low}$ in Hypermetabolic, only n = 8 for $METH^{high}$), statistical analyses in those substes may be less stable and should be interpreted with caution. Thus, future multicentric studies are needed to validate these results and perform further investigations on pre-stratified sub-cohorts. These considerations and adjusted parts of the Discussion section incorporating these new findings on the comparison of methylation clusters and previous classifications models can be found on pages 18, 19, and 21 as follows:

"Our study provides evidence for a DNA methylation signature that strongly correlates with poor clinical outcomes in meningioma patients and separates meningiomas in $METH^{low}$ and $METH^{high}$ cases using a binary cluster solution. Accordingly, methylation cluster assignment was largely in agreement with assignments to CNS WHO grades or molecular groups associated with bona fide low-risk or high-risk meningiomas. Furthermore, our data show that the binary cluster classification represents a surrogate for an underlying methylation gradient that is directly associated with a continuous, nonlinear risk gradient. In light of these findings, it is worth noting that the paradigm of previous assignments to distinct meningioma molecular groups has been recently challenged toward a tumour microenvironment-determined risk continuum[46], and our data might present evidence for a tumour-intrinsic contribution to this biologically relevant gradient. As such, models including the continuous methylation signature significantly improved risk prediction beyond both CNS WHO grade and molecular group across our entire cohort. In contrast to clinically well-defined low-risk or high-risk meningiomas, we show that classifications with a considerable heterogeneity in their clinical course (CNS WHO grade 2, Hypermetabolic, intermediate integrated risk)[19,25,47] showed a more balanced assignment to either $METH^{low}$ and $METH^{high}$ clusters. While we found that methylation cluster assignment successfully stratified outcomes of CNS WHO grade 2 tumors, it was unable to stratify Hypermetabolic or intermediate integrated risk meningiomas into groups with different clinical behaviour. However, these findings have to be seen in light of limited sample sizes in our cohort, in particular for $METH^{high}$ tumors in these intermediate-risk meningiomas. Future studies in larger, multicenter cohorts are needed improve resolution for these tumours. … Our study reveals a prognostically relevant DNA methylation signature that defines a continuous, nonlinear risk gradient in meningioma. While methylation clusters defined by this signature are largely in line with previous

classifications for bona fide low-grade and high-grade tumours, it may be particularly useful to predict clinical outcome for meningiomas currently classified as CNS WHO grade 2 and thereby inform clinical observation strategies in these patients accordingly."

Line 227: "our data suggest that DNA hypermethylation in METHhigh tumours is an independent mediator of meningioma progression, allowing for further prognostic sub-stratification of 1p-altered meningiomas."

Do other methylation predictors also stratify 1p loss cases, or is this a unique feature of the binary classification proposed here?

Response: We have accordingly modeled survival for molecular meningioma groups in relation to chr1p status in the Discovery cohort, excluding Proliferative 1p intact cases as there was only one such case in the cohort. Within molecular groups, 1p status only significantly stratified NF2-Wildtype meningioma to low- and high-risk tumors, while this was not the case for Immunogenic and Hypermetabolic meningiomas. Within 1p deleted meningioma, we did not detect any statistically significant differences in PFS between any of the molecular groups (only pairwise contrasts to NF2-Wildtype 1p loss are shown). Again, due to limited sample numbers, these results might not be statistically reliable. This has been added to the Results section on page 10 as follows:

"We did not observe a comparable stratification of 1p-deleted meningiomas by molecular groups, even though these analyses have to be seen with caution due to limited sample numbers (Extended Data Fig. 6f)."

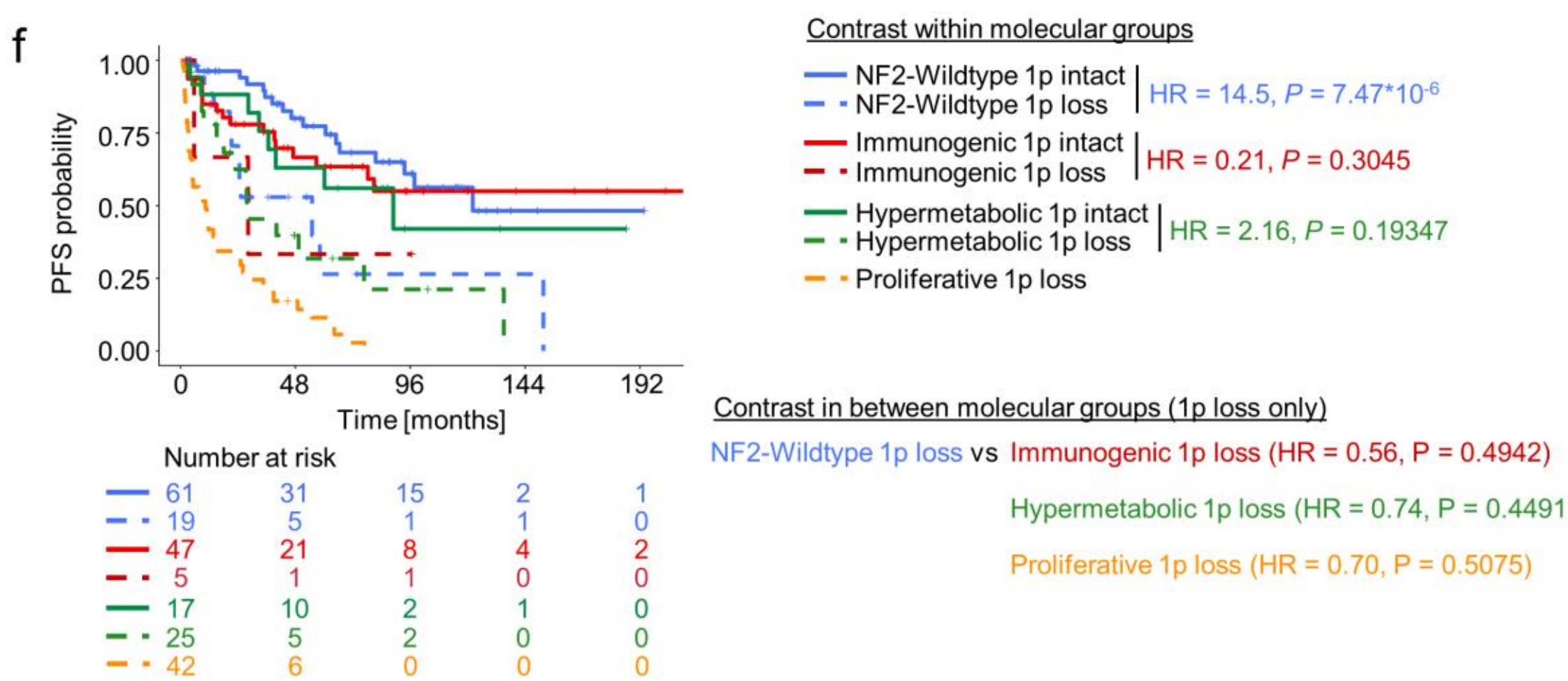

Extended Data Fig. 6: Validation of broad chromosomal CNVs in the Discovery and UCSF cohort.

f, *Kaplan-Meier analyses for the Discovery cohort, stratifying samples both by molecular group and chromosome 1p status. Hazard ratios from Cox proportional hazards modeling with adjustment for competing variables are shown for within molecular group comparisons, as well as for within 1p-deleted meningiomas, using NF2-Wildtype cases as a reference.*

Line 258: "we identified several candidates to exclusively occur in high-grade tumours including gene amplifications of MDM2, a negative regulator of p53 signalling, and gene deletion events for tumour suppressors such as VHL and CDKN2A/B (Fig. 4d,e)."

Is there statistical testing that supports enrichment of these genes in high-grade cases? How were these specific genes selected for the figure, there are no p values shown. Contrary to the statement in the main text ("exclusively occur in high-grade"), both MDM2 amplification and VHL deletion appears to occur in a WHO grade 1 tumor.

Response: We have performed a Fisher's exact test of over-enrichment for called focal CNVs in our longitudinal analysis, and the corresponding code can be found in the "10_longitudinal_CNV.R" code file. However, none of the comparisons came up to be significant among any of the contrasts employed (**1:** Primary_w/o_recurrence vs. Primary_w/_recurrence vs. Recurrences; **2:** Primary_w/o_recurrence vs. all other; **3:** all_Primaries vs. Recurrences). Since we saw several CNVs such as VHL deletion only in Primaries w/ recurrence and their recurrent counterparts (commonly referred to progressive meningiomas in the manuscript), we assumed that small cohort size precluded meaningful statistics. In this sense, we here arbitrarily selected several cancer candidate genes, focusing mostly on focal CNVs that exclusively occurred in progressive meningiomas. We agree that this should have been mentioned in the corresponding figure legend, and this has been adjusted. Additionally, we agree that the term "high-grade" was misleading in the respect, when we were referring to the group of progressive meningiomas (primaries and their recurrent counterparts). We have now adjusted that within the Results section on page 11 as follows:

"Screening for divergent CNVs between benign and progressive meningiomas, we identified several candidates to exclusively occur in recurrent tumours or their primary counterparts, but not in primaries that did not progress. These included gene amplifications of MDM2, a negative regulator of p53 signalling, and gene deletion events for tumour suppressors such as VHL and CDKN2A/B (Fig. 4d,e)."

Line 312: "We noted that a substantial number of genes associated with DNA hypermethylation formed part of several gene clusters, including HOX genes and protocadherin clusters (Fig. 6d and Extended Data Fig. 8c)."

Methylation changes in HOX genes have been well reported previously in meningiomas (PMIDs: 22735029, 23349797, 22102699, 31069492; and 30202034 as noted in the discussion); and to a lesser extent for protocadherins (PMIDs: 36551712). The authors should comment on these papers and if there are any unique findings from this new analysis.

Response: We certainly acknowledge the work shown in previous studies on HOX genes in meningioma. We gladly cite the four remaining publications together with the initial one on page 20 within the Discussion section. However, since HOX genes was not the focus of our work and we did not have a more detailed look at these genes in our cohort, we refrain from further discussing this class of genes at this point. We also gladly cite the publication on protocadherin hypermethylation in meningioma, and go on to discuss our new findings on a potential downstream mechanism of protocadherin downregulation involving β-catenin localization shown in our study. This has been added to the Discussion section on page 20 as follows:

“Of note, hypermethylation of all three protocadherin gene clusters has already been implicated as a potential proliferative biomarker associated with meningioma progression57, and we expand on these findings using a larger cohort. In particular, we now provide mechanistic insights showing that protocadherins from the PCDHG@ gene cluster can regulate the subcellular localization of β-catenin in meningioma cells. Furthermore, high levels of nuclear β-catenin staining correlated with poor clinical outcome in primary meningiomas. Thus, our data suggest a model where protocadherin expression conveys tumour suppressive functions by preventing translocation of β-catenin to the nucleus, and this mechanism can be attenuated by DNA hypermethylation and subsequent downregulation of protocadherin gene expression.”

Line 377: "In contrast, we did not detect any changes in the nuclear-to-cytoplasmic ratio of B-catenin when overexpressing PCDHGC3 in the benign cell line BEN-MEN-1."

How do you explain this? It is not clear to me why protocadherins would regulate B-catenin localization (and possibly production) only in the context of aggressive meningiomas. More broadly, I am concerned that this experiment has only shown an effect in a single meningioma cell line. It is possible that the observed effect on B-catenin localization is not due to benign vs. aggressive meningiomas (n = 1 each), but rather an effect of IOMM-LEE vs. BEN-MEN, which have many differences. To be more convincing, the authors should replicate this finding in the other cell lines that they have available (HBL-52, NCH93, KT21-MG1 are mentioned), in addition to primary cells if possible. This is particularly needed given the known hyper-aggressive phenotype associated with IOMM-LEE, which may poorly replicate meningioma biology.

Response: We thank this reviewer for bringing up this important point. In the primary submission, we had included only data on IOMM-Lee cells. While there had been no comment on the analyses regarding overexpression studies of protocadherins from the reviewers, we added the benign BEN-MEN-1 cell line data during first revision as a reference line to test our hypothesis. For a total of five cell models (two low-grade, three high-grade), we assessed β-catenin localization, and found that β-catenin is primarily localized in the nucleus in high-grade models, while localization is enriched in the cytoplasm in low-grade models (Fig. 7b,c). Based on previous literature showing evidence for both direct as well as indirect inhibitory interaction of protocadherins with β-catenin, in particular gamma protocadherin C3[4,5], we hypothesized that the predicted downregulation of protocadherins by hypermethylation in aggressive meningiomas (which we also validate by RNAseq in Fig. 6g and Extended Data Fig. 9e) might lead to less retention at the plasma membrane and/or less degradation of β-catenin altogether, thus potentially increasing nuclear levels in aggressive models. In this sense, overexpression studies were a rescue experiment to test whether re-expressing protocadherins might shift β-catenin levels back to the cytoplasm, which is where β-catenin is potentially degraded by a complex with APC and Axin1. In fact, overexpression of *PCDHGC3* in IOMM-Lee cells followed exactly our hypothesis by shifting catenin levels to the cytoplasm and presenting slightly lower total levels of β-catenin (Fig. 7e,f). Since the majority of β-catenin is already in the cytoplasm in BEN-MEN-1 cells (Fig. 7c), we did not expect a major shift of β-catenin back to the cytoplasm, and thus no decrease in total levels, and this is what we observed (Extended Data Fig. 11). In this sense, the effect is only visible in the context of aggressive meningioma models because only in those cells β-catenin levels in the nucleus are high enough to generate a detectable shift.

Our current experimental design was intended to provide a mechanistic proof-of-concept rather than a comprehensive survey across multiple models. This being said, we had initially tried to transduce all meningioma model at our hands with lentiviral particles coding for *PCDHGC3*, but were only successful in

two lines. While we agree that extension to additional models would be valuable, the current data already provide internally consistent and biologically plausible evidence supporting our proposed mechanism, which aligns well with our downstream analyses of TMA of primary meningiomas. We have added the publication showing indirect regulation of β-catenin by PCDHGC3 in the Results section on page 15 as follows:

"Protocadherins interact with β-catenin via their intracellular protein domain and might regulate β-catenin indirectly as well[33,34]."

Furthermore, we have mentioned plausible follow-up studies for further validation within the Discussion section on page 21 as follows:

"Thus, further research is needed to comprehensively dissect the role of protocadherins in meningioma development, potentially including further validation studies in suitable model system."

Line 57: "no recurrent genetic aberrations other than NF2 have been identified thus far in high-grade meningiomas,"

This does not seem accurate, I think you mean "genetic coding mutations". TERTp mutations and CDKN2A/B loss is sufficient for high-grade classification by the WHO, and CNVs such as 1p loss (among others) are well associated with aggressiveness. Alterations in BAP1 have also been proposed to be enriched in high-grade cases.

Response: We agree with this reviewer, and have adjusted this sentence within the Main section on page 3 as follows:

"While high-grade meningiomas have a higher mutational burden than low-grade meningiomas, no recurrent genetic coding mutations other than NF2 have been identified thus far in high-grade meningiomas, and numbers of targetable alterations in high-grade tumours tend to be lower than in low-grade meningiomas[10]."

Line 415: "In contrast, CNS WHO grade 2 meningiomas, encompassing the majority of hypermetabolic/integrated intermediate-risk tumours that are characterized by a considerable heterogeneity in their clinical course, showed a more balanced assignment to either METHlow and METHhigh clusters."

However, you show that there is not a statistical difference conferred by METH high/low for hypermetabolic/integrated intermediate-risk. This should be acknowledged explicitly here. Similar changes should be made to the statement "it may be particularly useful to predict clinical outcome for meningiomas currently classified as intermediate-risk and thereby inform clinical..", which should explicitly say "Grade 2" instead of "intermediate risk", in order to match your results.

Response: We agree with this reviewer and have now adjusted these two sentences in the Discussion section on pages 18, 19, and 21 to more accurately match our results:

"In contrast to clinically well-defined low-risk or high-risk meningiomas, we show that classifications with a considerable heterogeneity in their clinical course (CNS WHO grade 2, Hypermetabolic, intermediate

integrated risk)[19,25,46] showed a more balanced assignment to either METHlow and METHhigh clusters. While we found that methylation cluster assignment successfully stratified outcomes of CNS WHO grade 2 tumors, it was unable to stratify Hypermetabolic or intermediate integrated risk meningiomas into groups with different clinical behaviour. However, these findings have to be seen in light of limited sample sizes in our cohort, in particular for METH^{high} tumors in these intermediate-risk meningiomas. Future studies in larger, multicenter cohorts are needed improve resolution for these tumours. … While methylation clusters defined by this signature are largely in line with previous classifications for bona fide low-grade and high-grade tumours, it may be particularly useful to predict clinical outcome for meningiomas currently classified as CNS WHO grade 2 and thereby inform clinical observation strategies in these patients accordingly."

Line 427: "Together, our data reveal a hypermethylation signature directly associated with meningioma progression, and this association is independent of previously described molecular groups defined mainly based on DNA methylation."

From my understanding this statement is not accurate, there is in fact a strong association of previously described molecular groups and your hyper-methylation signature (Fig 1d). The sentence should be clarified. Similar adjustments should be made to line 476 "Our study reveals a prognostically relevant DNA hypermethylation signature that acts independently of molecular groups defined by global DNA methylation".

Response: We apologize for our confusing wording. Indeed, "independent" or "independently" was misleading in this case, as in fact we provide several lines of evidence now showing the strong association of our signature and previously described molecular groups (e.g. Fig. 1d, Extended Data Fig. 9a). Strengthened by our new data, we now show that the cluster signature overall adds additional prognostic value beyond CNS WHO grading and molecular group classifications, which however does not mean that it is "independent" altogether from previously described groups. During the course of rigorous adjustment of the Discussion section, these two sentences have either been deleted from the manuscript, or adjusted as follows on page 21:

"Our study reveals a prognostically relevant DNA methylation signature that defines a continuous, nonlinear risk gradient in meningioma."

Reviewer #5 (Remarks on code availability):

The code is easily accessible and well documented on the Anonymous GitHub website.

Response: We thank this reviewer for acknowledging the integrity of our code repository.

1. Mansouri, A., *et al.* Surgically resected skull base meningiomas demonstrate a divergent postoperative recurrence pattern compared with non-skull base meningiomas. *J Neurosurg* **125**, 431-440 (2016).
2. Gauthier, J., Wu, Q.V. & Gooley, T.A. Cubic splines to model relationships between continuous variables and outcomes: a guide for clinicians. *Bone Marrow Transplant* **55**, 675-680 (2020).
3. Pencina, M.J., D'Agostino, R.B., Sr., D'Agostino, R.B., Jr. & Vasan, R.S. Evaluating the added predictive ability of a new marker: from area under the ROC curve to reclassification and beyond. *Stat Med* **27**, 157-172; discussion 207-112 (2008).
4. Mah, K.M., Houston, D.W. & Weiner, J.A. The gamma-Protocadherin-C3 isoform inhibits canonical Wnt signalling by binding to and stabilizing Axin1 at the membrane. *Sci Rep* **6**, 31665 (2016).
5. Han, M.H., Lin, C., Meng, S. & Wang, X. Proteomics analysis reveals overlapping functions of clustered protocadherins. *Mol Cell Proteomics* **9**, 71-83 (2010).

Third revision for manuscript NCOMMS-25-62380B

“DNA methylation profiling identifies long-range epigenetic silencing of clustered protocadherins as a key determinant of meningioma progression”

Point-by-point response letter

REVIEWER COMMENTS

Reviewer #2 (Remarks to the Author): The authors have responded the reviewers' comments and significantly improved the manuscript.

Response: We thank this reviewer for acknowledging our work.

Minor typo: line 454 studies in larger, multicenter cohorts are needed TO improve resolution for these tumours.

Response: We have corrected this typo.

Reviewer #5 (Remarks to the Author): The authors have corrected the concerns raised in my previous review.

Response: We thank this reviewer for acknowledging our work.

Reviewer #5 (Remarks on code availability): The code is easily accessible and well documented on the Anonymous GitHub website.

Response: We thank this reviewer for acknowledging our efforts to make our study fully reproducible.

Reviewer #6 (Remarks to the Author): We reviewed the revised manuscript with a focus on the issues previously raised by Reviewer #4. Overall, the authors have addressed most of prior concerns. We only have a few technical comments.

Response: We thank this reviewer for acknowledging our work.

1. Statistical test reporting for Fig. 3c remains inconsistent

Reviewer #4 recommended using Kruskal-Wallis tests consistently for the relevant multi-group comparisons. The authors state in the rebuttal that Kruskal-Wallis tests were applied to all comparisons in Fig. 3c, and the corresponding code appears to use Kruskal-Wallis tests. However, the revised Fig. 3 legend still states that “P values are derived from one-way ANOVA with Dunnett multiple comparison tests.” The legend should be corrected to match the revised statistical analysis

Response: We have accordingly corrected this mistake.

2. Data availability and code reproducibility remain only partially resolved

The README and repository have been improved, including dataset descriptions, accession information, package versions, and script descriptions. However, the workflow is still not sufficiently clear for reproducibility.

First, the authors state in the response letter and README that required datasets are available through Figshare. However, we were unable to download the data from the provided Figshare private link, which prevented us from verifying whether the required files are complete and sufficient for reproducing the analyses. The authors should ensure that the final public Figshare link made available upon acceptance is fully accessible and downloadable, and that each Figshare data folder is clearly mapped to the scripts that use it.

Response: We thank this reviewer for this important point. We have made sure that all Figshare entries are now publicly available (https://doi.org/10.6084/m9.figshare.32758710 and https://doi.org/10.6084/m9.figshare.32043249). Each data folder is clearly mapped to the corresponding scripts from GitHub that use it (see comments below).

Second, several scripts still rely on hard-coded local paths (e.g., /Volumes/..., /Users/..., or ~/Desktop/...), and many input files are read from local working directories. We recommend organizing the repository as a project-based structure with a data directory downloaded from Figshare, and revising scripts to use relative paths rather than the hard-coded local paths.

Response: We thank this reviewer for their comment. We have now organized the Figshare repository in a clear project-based structure. Additionally, we have revised all code deposited at GitHub to now use relative paths using the R library *here*. The README file on GitHub now contains detailed information on how to clone the Figshare repository to make the code fully reproducible.

Reviewer #6 (Remarks on code availability): Please see my comments above.

Response: We have responded accordingly above.

Reviewer #7 (Remarks to the Author):

I co-reviewed this manuscript with one of the reviewers who provided the listed reports. This is part of the Nature Communications initiative to facilitate training in peer review and to provide appropriate recognition for Early Career Researchers who co-review manuscripts.

Response: We thank this reviewer for their efforts in the review process.
